# Synthesis of the polyketide section of seragamide A and related cyclodepsipeptides via Negishi cross coupling

**DOI:** 10.3762/bjoc.15.53

**Published:** 2019-02-28

**Authors:** Jan Hendrik Lang, Thomas Lindel

**Affiliations:** 1 TU Braunschweig, Institute of Organic Chemistry, Hagenring 30, 38106 Braunschweig, Germany

**Keywords:** jasplakinolide, marine natural products, Negishi coupling, polyketides, stereoselective synthesis

## Abstract

The synthesis of the polyketide section present in the potently cytotoxic marine cyclodepsipeptide jasplakinolide and related natural products, geodiamolides and seragamides, is reported. The key step is a Negishi cross coupling of (*R*)-(3-methoxy-2-methyl-3-oxopropyl)zinc(II) bromide and an (*E*)-iodoalkene that was synthesized via an aluminium ester enolate attack at (*R*)-propylene oxide. The overall synthesis comprises nine steps with an overall yield of 21%. It proved to be possible to liberate the free 8-hydroxynonenoic acid and to couple it with a protected tripeptide composed of L-alanine, *N,O*-dimethyl-D-iodotyrosine, and TIPS-protected L-threonine, which occurs as partial structure of seragamide A. The tripeptide section of seragamide A was assembled by solution-phase synthesis and an open-chain analogue of the natural product was obtained.

## Introduction

**Scheme 1:**
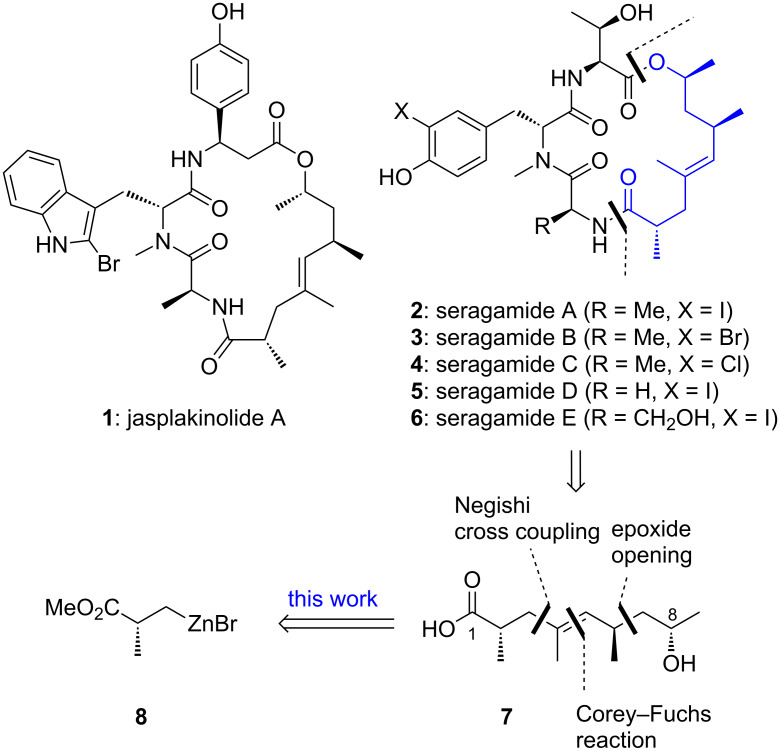
Actin-binding cyclodepsipeptides, photo amino acids, retrosynthetic cuts of polyketide **7** leading to organozinc compound **8**.

Our program on the synthesis of biologically active natural products with peptide partial structures is driven by an interest in the chemistry of photoreactive amino acids and heterocycles that may find application in photoaffinity labelling. Knowing the binding of a natural product to a biological target at atomic resolution, as it is the case for the cyclodepsipeptide jasplakinolide A (**1**, Scheme 1) [1–2], is an ideal situation for the validation of the chemoselectivity and efficiency of photoaffinity labelling. Recently, it has been determined by cryo-electron microscopy how jasplakinolide A (**1**) binds to F-actin and alters the actin skeleton in vivo, resulting in pronounced cytotoxicity [3]. A photoreactive derivative of the cytotoxic jasplakinolides, geodiamolides [4–5], or seragamides (**2**–**6**, seragamides A–E) [6] could also enable the search for additional targets in the cell, including proteins involved in transport or even membrane components. For this purpose, the 2-bromoabrine unit of **1** could be replaced by phototryptophan [7], whereas photo β-phenylalanine [8] could replace the β-tyrosine moiety. For photoaffinity labelling studies with seragamides and geodiamolides, D-photophenylalanine could be incorporated.

In this paper we describe, as the first step of such an endeavour, a new synthesis of the polyketide section present in seragamides and most of jasplakinolides and geodiamolides. We also include the synthesis of the protected tripeptide section of seragamide A (**2**), even if the macrocycle still remains to be assembled. Seragamide A (**2**) from the marine sponge *Suberites japonicus* (Thiele) has been synthesized only once, with relay ring-closing metathesis being the key step [9]. Characteristically, seragamides A–E exhibit a L-threonine unit at the C-terminus of the peptide moiety.

There is a considerable body of work on the synthesis of the C_12_ polyketide section. In most cases, the C2–C3 bond was formed by Ireland-, Johnson-, or Eschenmoser–Claisen rearrangement [10–17]. Alternatively, Evans methodology has been used [18–20]. The central C4–C5 double bond has also been constructed by Wittig reaction, cross metathesis, and ring-closing metathesis [9,21–22]. The C3–C4 bond has been assembled by 1,2-cuprate rearrangement [23]. Iterative construction of the polyketide from smaller building blocks has been achieved by Matteson homologation [24], that was also applied to the synthesis of the related polyketide section of lagunamide A [25]. In addition, the shorter C_11_ polyketide section of the closely related chondramides has been assembled in several ways [26–29].

## Results and Discussion

**Scheme 2:**
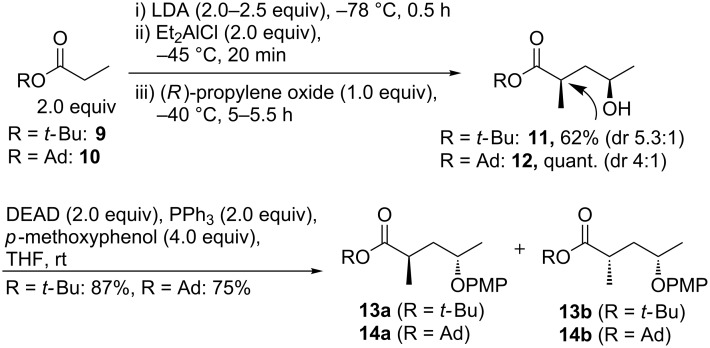
Synthesis of γ-hydroxy esters **11** and **12**, followed by Mitsunobu inversion.

In our approach the polyketide section (**7**, Scheme 1) of seragamide A (**2**) was to be assembled via construction of the C3–C4 bond by sp^3^–sp^2^ Negishi cross coupling. The required organozinc reagent **8** has been applied occasionally and is accessible from the corresponding commercially available chiral bromide [30–34]. The coupling partner would be an (*E*)-iodoalkene that was to be constructed from enantiomerically pure (*R*)-propylene oxide. The C4–C5 double bond would be formed by Corey–Fuchs reaction. Reaction of the aluminium enolate of *tert*-butyl propionate (**9**, Scheme 2) with (*S*)-propylene oxide had provided Taylor et al. with a mixture of diastereomers of α-methylated γ-hydroxyester **11** favouring the (2*S*,4*S*)-*syn* over the (2*R*,4*S*)-*anti* isomer [35–36]. As the configuration at C4 was expected to be invertible in a later step, we aimed for the (2*R,*4*R*) configuration by choosing (*R*)-propylene oxide as electrophile. We obtained an acceptable ratio of diastereomers of **11** (84:16 in favour of (2*R*,4*R*), 62% combined) after having treated *tert*-butyl propionate (**9**) with LDA/Et_2_AlCl (Scheme 2). Performing the reaction at −78 °C instead of −40 °C unexpectedly diminished the dr, which is in agreement with results by Liu, Petersen and co-workers [37]. The analogous reaction of adamantyl ester **10** (obtained from adamantan-1-ol and propionyl chloride/pyridine, 85%) instead of **9** gave a quantitative yield of the hydroxy ester **12**, but a lower dr (4:1). For both cases, we inverted the carbinol centre by Mitsunobu reaction employing *p*-methoxyphenol. DEAD was used instead of DIAD because of facilitated purification. It was possible to separate the diastereomers of product **13** by column chromatography. The use of DMEAD (di-2-methoxyethyl azodicarboxylate) [38] was inferior (33%). The PMP protecting group was envisioned to be stable during the following steps and to be selectively cleavable with ceric ammonium nitrate (CAN).

**Scheme 3:**
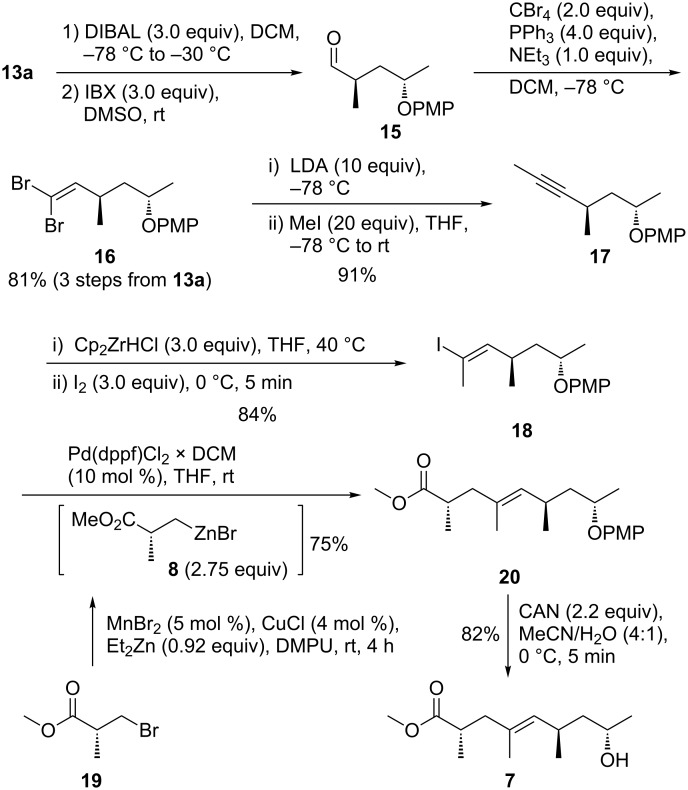
Synthesis of the polyketide section **7**.

Reduction of *tert*-butyl ester **13a** to the alcohol by portionwise addition of DIBAL and careful temperature control (slow warm-up from −78 to −30 °C) afforded the alcohol without α-epimerization, which was oxidized to aldehyde **15** (IBX, Scheme 3). Adamantyl ester **14** resisted reduction under the same conditions and was abandoned.

Dibromoalkene **16** was accessed from aldehyde **15** under Corey–Fuchs conditions and treated with *n*-hexane to separate triphenylphosphane oxide. For the conversion of **16** to the internal alkyne **17**, the use of a large excess of freshly prepared LDA and iodomethane with a slow warm-up from −78 °C to rt [39] proved to be superior to the more commonly employed *n*-BuLi/MeI reagent combination that led to the formation of a series of side products. We also attempted a Bestmann–Ohira reaction with in situ formation of the diazo phosphonate [40] that gave a slightly inferior yield after methylation of the alkyne terminus. Hydrozirconation/iodination of alkyne **17** required freshly prepared Schwartz reagent (3 equiv) and iodine (3 equiv) to obtain yields of (*E*)-olefin **18** above 80% with perfect stereo- and regioselectivity. For comparison, as part of a study towards kendomycin, Arimoto and co-workers had published a route to a 1:1 mixture of diastereomers of a TBS-protected analogue of building block **18** [41].

Negishi cross coupling of sp^3^ organozinc homoenolate **8** (2.75 equiv) and iodoalkene **18** (10 mol % [Pd(dppf)Cl_2_ × DCM] in DCM) afforded a satisfying 75% yield of the protected nonenoic acid **20** (Scheme 3). For the synthesis of organozinc homoenolate **8**, the MnBr_2_/CuCl-catalyzed reaction of diethylzinc with β-bromopropionic acid ester **19** in DMPU proved to be the best choice [42]. Racemization was avoided.

The PMP protecting group was removed (CAN) affording methyl ester **7** (82%, Scheme 3). For comparison with literature data we converted **7** to the free carboxylic acid carrying a TBS-protected hydroxy group at C8 by silylation with TBSOTf/2,6-lutidine, followed by saponification with LiOH.

**Scheme 4:**
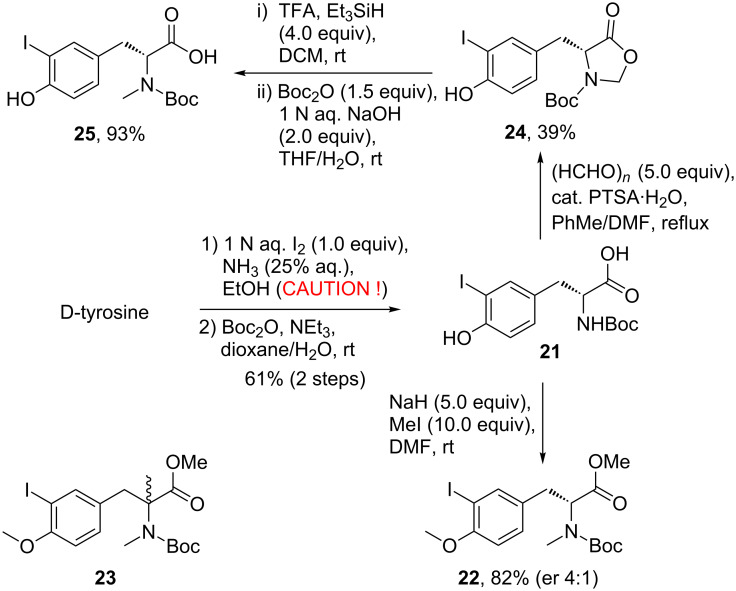
Access to methylated D-iodotyrosine derivatives **22**, **23**, and **25**.

To investigate whether polyketide **7** could be coupled in an unprotected form with the peptide fragment of seragamide A (**2**), we synthesized the tripeptide section. The assembly of the iodotyrosine moiety commenced with D-3-iodotyrosine that was obtained in 63% yield from D-tyrosine upon treatment with iodine (1 equiv, added via syringe pump over 90 min to avoid diiodination) in aqueous ammonia [43] (Scheme 4). This setup also served to avoid the formation of excess amounts of the explosive iodinating reagent NI_3_. On work-up, the pH had to be adjusted to exactly 4.5 to destroy remaining NI_3_ and to precipitate the product. Other iodination conditions such as use of NaI/NaOCl [44–45] or I_2_/CAN [46] did not convince. Boc protection yielded *N*-Boc-D-3-iodotyrosine (**21**), which underwent three-fold methylation to *N*-methyl-*O*-methyliodotyrosine methyl ester **22** (82% yield, er 4:1, CHIRALPAK^®^ IA, hexane/iPrOH 9:1). Partial racemization of **21** could not be suppressed since a slight excess of base was always necessary, whereas the use of exactly stoichiometric amounts resulted in incomplete *N*-methylation. Treating the Boc-protected methyl ester of iodotyrosine **21** with a larger excess of base and methyl iodide (10 equiv each) led to the formation of the α-quaternary amino acid **23** (Scheme 4). An alternative route to the *N*-methylated product via oxazolidinone **24** was hampered by low yield on reaction with paraformaldehyde [47–48]. Although subsequent reduction of **24** with Et_3_SiH/TFA and reprotection of the free *N*-methyl amino acid with Boc_2_O proceeded smoothly and delivered iodotyrosine **25**, the overall yield via **24** was not satisfying.

**Scheme 5:**
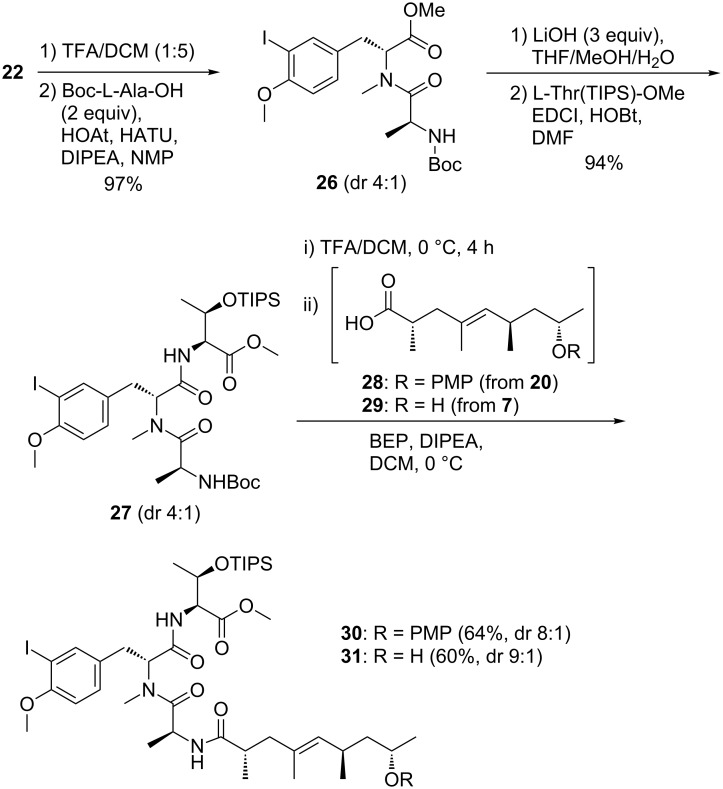
Synthesis of the doubly protected open chain peptide-polyketide **31**.

After Boc removal at **22**, HATU/HOAt-mediated coupling with Boc-protected L-alanine afforded dipeptide **26**, which was saponified and coupled with TIPS-protected threonine methyl ester (obtained with TIPSCl/DBU/MeCN) to provide tripeptide **27** (Scheme 5). Peptides **26** and **27** were obtained in nearly quantitative yield, albeit as mixtures of inseparable diastereomers (4:1), as consequence of the optical impurity of **22**. Only when following the coupling order shown in Scheme 5, high yields could be obtained.

The labile carboxylic acid **29** was formed by saponification of methyl ester **7** (LiOH, H_2_O/MeOH) and underwent BEP-mediated coupling with tripeptide **27** (Scheme 5). Product **31** (60%) was obtained with a higher dr of 9:1 than tripeptide **27** (dr 4:1). We have evidence that the major side product of **31** was a desilylated analogue, for which the ^1^H NMR spectrum indicated a lower dr of about 7:3. The analogous product **30** carrying an 8-OPMP group was obtained from PMP-protected polyketide **28**.

The endgame of a total synthesis of seragamide A (**2**) will have to address the macrocyclisation, desilylation of the threonine moiety, and *O*-demethylation of the D-iodotyrosine unit of **31**. In orienting reactions on small scale, desilylation of **31** (TBAF) was a spot-to-spot conversion. Saponification of both the silylated and desilylated methyl esters with LiOH was possible, as long as very mild acidic conditions were applied on work-up. However, macrocyclisation of the TIPS-protected or the desilylated acid did not occur. Under Yamaguchi conditions, we observed complete decomposition in both cases. Shiina conditions gave a complex product mixture, if the TIPS group was present, whereas the desilylated acid provided a product that apparently had undergone dehydration, as judged by TLC–MS coupling. In course of their recent synthesis of plusbacin A_3_, Ichikawa et al. experienced similar difficulties when attempting esterification of a bulky β-OTIPS-substituted amino acid with a secondary alcohol under a variety of conditions [49].

## Conclusion

We developed a short synthesis of the polyketide building block present in most of the jasplakinolides, geodiamolides, and seragamides, potently cytotoxic peptide-polyketide cyclodepsipeptides stabilizing filamentous actin. The key step is a Negishi cross coupling, and the carbon atoms stem from (*R*)-propylene oxide (C7–C9), *tert*-butyl propionate (**9**, C5–C6, 6-Me), CBr_4_ (C4), MeI (4-Me), and (*R*)-(3-methoxy-2-methyl-3-oxopropyl)zinc(II) bromide (**8**, C1–C3, 2-Me). We obtained 8-hydroxyester **7** within nine steps and an overall yield of 21% starting from (*R*)-propylene oxide. The efficiency of our route to polyketide **7** compares well to earlier approaches. It proved to be possible to liberate the free carboxylic acid **29** and to couple it with the protected tripeptide **27** that was obtained by solution phase synthesis. We will now aim at the macrocyclisation of analogues of compound **31**, which will probably require replacement of the bulky TIPS group installed at the threonine unit. The synthesis of photoreactive tripeptide analogues incorporating photo amino acids **4**–**6** will be explored thereafter.

## Supporting Information

File 1 Experimental procedures and NMR spectra.
