# Supplementary material for: Synthesis of the polyketide section of seragamide A and related cyclodepsipeptides via Negishi cross coupling
Source: Beilstein J Org Chem. 2019 Feb 28;15:577–83. doi: 10.3762/bjoc.15.53 (PMC6404460; doi:10.3762/bjoc.15.53)
Supplement: File 1 — Experimental procedures and NMR spectra. [file Beilstein_J_Org_Chem-15-577-s001.pdf]

BEILSTEIN JOURNAL OF ORGANIC CHEMISTRY

Supporting Information

for

Synthesis of the polyketide section of seragamide A and related cyclodepsipeptides via Negishi cross coupling

Jan Hendrik Lang and Thomas Lindel

Beilstein J. Org. Chem. **2019,** *15,* 577–583. doi:10.3762/bjoc.15.53

Experimental procedures and NMR spectra

License and Terms: This is a supporting information file under the terms of the Creative Commons Attribution License (http://creativecommons.org/licenses/by/4.0). Please note that the reuse, redistribution and reproduction in particular requires that the authors and source are credited. The license is subject to the *Beilstein Journal of Organic Chemistry* terms and conditions: (https://www.beilstein-journals.org/bjoc)

Table of contents

1 Analytical data, reagents and working techniques S3

2 List of used abbreviations S4

3 Syntheses S5

3.1 Synthesis of the polyketide section S5

3.2 Synthesis of amino acids for the tripeptide section S17

3.3 Synthesis of tripeptides S25

3.4 Synthesis of “open-chain seragamide A” S33

4 ^{1}H and ^{13}C NMR spectra S37

1 Analytical data, reagents and working techniques

Chemicals and synthesis

Chemicals were purchased from Aldrich, Acros, ABCR, TCI, BASF, Merck and Carbolution and, if not stated otherwise, used without further purification. If synthesis was conducted under an argon atmosphere (Schlenk conditions), it is stated in the procedures, otherwise, reactions were carried out without argon. The solvents for reactions under air were distilled prior to use or purchased. For reactions under an argon atmosphere, solvents were dried and distilled under argon using a Solvent Purification System. Yields that are given for each step refer to purified compounds.

Optical rotatory power was recorded with a Dr. Kernchen Propol automatic polarimeter at the given concentration [$[\alpha]_D$: deg·cm^3·g^{-1}·dm^{-1}, *c*: g/100 cm^3]. Solvents are given in the procedures. **NMR spectra** were recorded with a Bruker AVII-300 (300 MHz for ^{1}H, 75.5 MHz for ^{13}C, *T* = 297 K), a Bruker AVIIIHD-300N (300 MHz for ^{1}H, 75.5 MHz for ^{13}C, *T* = 298 K), a Bruker DRX400 (400 MHz for ^{1}H, 100.6 MHz for ^{13}C, *T* = 298 K), a Bruker AVIII-400 (400 MHz for ^{1}H, 100.6 MHz for ^{13}C, *T* = 297 K), a Bruker AVIII-HD500, (500 MHz for ^{1}H, 125.8 MHz for ^{13}C, *T* = 298 K) and a Bruker AVII-600 (600 MHz for ^{1}H, 150.9 MHz for ^{13}C, 60.8 MHz for ^{15}N, *T* = 303 K). Chemical shifts (δ) are given in ppm as referenced to tetramethylsilane (TMS). Signals were assigned with the aid of ^{1}H,^{1}H-COSY, ^{1}H,^{13}C-HSQC, ^{1}H,^{13}C-HMBC, ^{1}H,^{15}N-HMBC, ^{1}H,^{1}H-NOESY and ^{1}H,^{1}H-TOCSY experiments. ^{15}N NMR spectra were referenced to the external standard CH_3NO_2[1] and chemical shifts are given as observed in the ^{1}H,^{15}N-HMBC experiments. **Mass spectra** were recorded with EI and ESI ionization methodology. For EI measurements, a Finnigan MAT 95 XL (Thermo Finnigan MAT) or a GC-EIMS system consisting of an Agilent 6890 gas chromatograph (column: Phenomenex ZB5-MS, 30 m length × 0.25 mm internal diameter, 0.25 µm phase density) and a JMST100GC (GCAccuTOF, JEOL, Japan) mass spectrometer were used. For ESI measurements, a LTQ Orbitrap Velos (ThermoFisher Scientific) was employed. **IR spectra** were recorded with a Bruker Tensor 27 spectrometer. **UV–vis spectra** were recorded with a Varian Cary 100 Bio UV–vis-spectrometer. Solvents are given in the procedures. **Melting points** were determined with a Büchi 530 or a Büchi M-560 device and are uncorrected. **Thin layer chromatography (TLC)** was performed on Merck silica 60 F_{254} aluminum sheets. **Column chromatography** was performed using Merck Geduran® silica 40–63 µm with elevated pressure (“flash” chromatography[2]). Eluents are given in the procedures (PE: petroleum ether (bp 40 to 60 °C), EA: ethyl acetate, MTBE: *tert*-butyl methyl ether).

---

[1] Harris, R. K.; Becker, E. D.; Cabral De Menezes, S. M.; Granger, P.; Hoffman, R. E.; Zilm, K. W. *Magn. Reson. Chem.* **2008**, *46*, 582-598.

[2] Still, W. C.; Kahn, M.; Mitra, A. *J. Org. Chem.* **1978**, *43*, 2923-2925.

2 List of used abbreviations

Protecting groups

Boc	*tert*-butoxycarbonyl
PMP	*p*-methoxyphenyl
TBS	*tert*-butyldimethylsilyl
TIPS	triisopropylsilyl

Solvents

DCM	dichloromethane
DIPEA	diisopropylethylamine (Hünig's base)
DMF	*N*,*N*-dimethylformamide
DMPU	*N*,*N*'-dimethylpropylene urea
DMSO	dimethyl sulfoxide
EA / EtOAc	ethyl acetate
MeCN	acetonitrile
MTBE	*tert*-butyl methyl ether
PE	petroleum ether (bp 40 to 60 °C)
TFA	trifluoroacetic acid
THF	tetrahydrofuran

Peptide coupling reagents and additives

BEP	2-bromo-1-ethyl-pyridiniumtetrafluoroborate
DEPBT	3-(diethoxyphosphoryloxy)-1,2,3-benzotriazin-4(3*H*)-one
EDC(I)	*N*-(3-dimethylaminopropyl)-*N*′-ethylcarbodiimide(-hydrochloride)
HATU	1-[bis(dimethylamino)methylene]-1*H*-1,2,3-triazolo[4,5-*b*]pyridinium-3-oxide hexafluorophosphate
HBTU	*O*-(benzotriazol-1-yl)-*N*,*N*,*N*′,*N*′-tetramethyluronium hexafluorophosphate
HOAt	1-hydroxy-7-azabenzotriazole
HOBt	1-hydroxybenzotriazole
PyAOP	(7-azabenzotriazol-1-yloxy)tripyrrolidinophosphonium hexafluorophosphate

3 Syntheses

3.1 Synthesis of the polyketide section

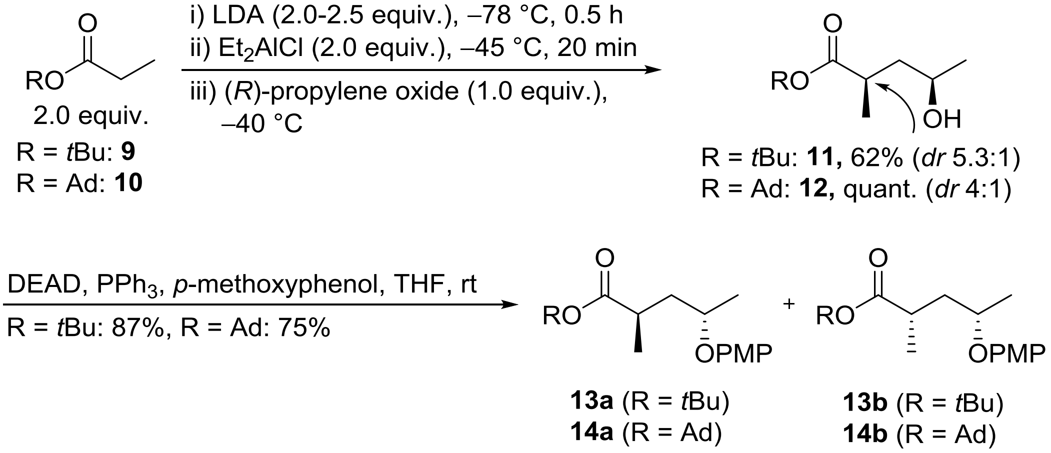

3.1.1 Adamantan-1-yl propionate (10)

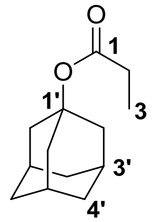

10

At room temperature, 1-adamantanol (8.000 g, 52.55 mmol, 1.0 equiv) was dissolved in DCM (170 mL). Propionyl chloride (10.10 mL, 115.62 mmol, 2.2 equiv) and pyridine (26.78 mL, 331.08 mmol, 6.3 equiv) were added and the mixture was stirred for 19 h. Then, saturated aqueous $NaHCO_3$ (100 mL) was added, the phases were separated and the aqueous phase was further extracted with MTBE (3 × 75 mL). The combined organic phases were dried over $MgSO_4$ and the solvents were removed in vacuo. After column chromatography [silica, PE/EA (10:1)], the ester **10** (9.358 g, 44.93 mmol, 85%) was obtained as a colorless oil.

TLC [PE/EA (10:1)]: R_f = 0.56. **^{1}H NMR** (300 MHz, $CDCl_3$): δ = 2.23 (q, J = 7.6 Hz, 2H, C(2)H_2), 2.18-2.03 (m, 3H, C(3')H), 2.11-2.00 (m, 6H, C(2')H_2), 1.68-1.64 (m, 6H, C(4')H_2), 1.08 (t, J = 7.6 Hz, 3H, C(3)H_3). **^{13}C NMR** (75.5 MHz, $CDCl_3$): δ = 173.7 (1C, C(1)), 80.0 (1C, C(1')), 41.3 (3C, C(2')H_2), 36.2 (3C, C(4')H_2), 30.8 (3C, C(3')H), 28.9 (1C, C(2)H_2), 9.2 (1C, C(3)H_3). **IR** (ATR): $\tilde{\nu}$ = 2910 cm^{-1} (m), 2854 (m), 1727 (s), 1457 (m), 1350 (m), 1271 (m), 1185 (s), 1104 (m), 1079 (m), 1056 (s), 1004 (w), 967 (m), 938 (w), 910 (m), 866 (m), 810 (m), 756 (w), 726 (w). **UV** (CH_2Cl_2): λ_{max} (lg ε) = –. **HRESIMS**: calc. for $C_{13}H_{20}O_2Na$ $[M+Na]^+$ 231.13555; found 231.13559.

3.1.2 *tert*-Butyl (2*R*,4*R*)-4-hydroxy-2-methylpentanoate (11)

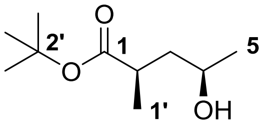

11

Under an argon atmosphere, diisopropylamine (4.83 mL, 34.40 mmol, 2.0 equiv) was dissolved in THF (50 mL) at −78 °C. *n*-BuLi (21.5 mL, 34.40 mmol, 1.6 M in hexanes, 2.0 equiv) was added dropwise and the mixture was stirred for 30 min. *tert*-Butyl propionate (**9**, 5.18 mL, 34.40 mmol, 2.0 equiv) was added, followed by an additional 30 min of stirring at −78 °C. The reaction was warmed to −45 °C and Et_2AlCl (34.4 mL, 34.40 mmol, 1 M in hexanes, 2.0 equiv) was added over a period of 5 min. After 15 min of continued stirring, (*R*)-propylene oxide (1.21 mL, 17.20 mmol, 1.0 equiv) was added and the mixture was stirred for 5.5 h with the temperature being kept at roughly −40 °C. After quenching with saturated aqueous NH_4Cl (20 mL), the suspension was carefully poured into a mixture of

aqueous HCl (6 M, 50 mL) and ice (50 g). After fuming had ceased, the phases were separated and the aqueous phase was further extracted with Et_2O (2 × 100 mL). The combined organic phases were washed with saturated aqueous $NaHCO_3$ (2 × 50 mL) and saturated aqueous NaCl (75 mL), dried over $MgSO_4$ and the solvents were removed in vacuo. Purification of the crude product was achieved by vacuum distillation (10 cm Vigreux column, bp: 77 °C/1.7 mbar). The hydroxy ester **11** (1.999 g, 10.62 mmol, 62%) was obtained as a colorless oil and as a mixture of diastereomers at C2, which could not be separated at this stage (*dr* 5.3:1).

TLC [PE/EA (5:1)]: R_f = 0.22. $[\alpha]_D^{21}$ = −9.2 (*c* = 1.20, $CHCl_3$). **^{1}H NMR** (400 MHz, $CDCl_3$, major diastereomer): δ = 3.90-3.82 (m, 1H, C(4)*H*), 2.59 (dqd, *J* = 9.8 Hz, *J* = 7.0 Hz, *J* = 4.5 Hz, 1H, C(2)*H*), 2.01 (br s, 1H, O*H*), 1.74 (ddd, *J* = 13.9 Hz, *J* = 9.8 Hz, *J* = 3.6 Hz, 1H, C(3)H_2), 1.50 (ddd, *J* = 13.8 Hz, *J* = 9.1 Hz, *J* = 4.5 Hz, 1H, C(3)H_2), 1.46 (s, 9H, C(3')H_3), 1.19 (d, *J* = 6.2 Hz, 3H, C(5)H_3), 1.15 (d, *J* = 7.0 Hz, 3H, C(1')H_3). **^{13}C NMR** (100.6 MHz, $CDCl_3$, major diastereomer): δ = 176.3 (1C, *C*(1)O), 80.4 (1C, *C*(2')), 65.8 (1C, *C*(4)H), 43.1 (1C, *C*(3)H_2), 37.3 (1C, *C*(2)H), 28.1 (3C, *C*(3')H_3), 23.6 (1C, *C*(5)H_3), 17.7 (1C, *C*(1')H_3). **^{1}H NMR** (400 MHz, $CDCl_3$, minor diastereomer): δ = 3.90-3.82 (m, 1H, C(4)*H*), 2.54-2.45 (m, 1H, C(2)*H*), 2.01 (br s, 1H, O*H*), 1.84 (ddd, *J* = 14.1 Hz, *J* = 8.7 Hz, *J* = 7.9 Hz, 1H, C(3)H_2), 1.49-1.41 (m, 1H, C(3)H_2), 1.45 (s, 9H, C(3')H_3), 1.21 (d, *J* = 6.2 Hz, 3H, C(5)H_3), 1.18 (d, *J* = 7.1 Hz, 3H, C(1')H_3). **^{13}C NMR** (100.6 MHz, $CDCl_3$, minor diastereomer): δ = 176.6 (1C, *C*(1)), 80.2 (1C, *C*(2')), 66.6 (1C, *C*(4)H), 43.0 (1C, *C*(3)H_2), 38.1 (1C, *C*(2)H), 28.0 (3C, *C*(3')H_3), 24.0 (1C, *C*(5)H_3), 17.7 (1C, *C*(1')H_3). **IR** (ATR): $\tilde{\nu}$ = 3431 cm^{-1} (w, br), 2972 (m), 2934 (m), 2879 (w), 1725 (m), 1458 (m), 1367 (m), 1255 (m), 1231 (m), 1149 (s), 1082 (m), 1037 (m), 1009 (w), 955 (w), 928 (w), 848 (m), 749 (w). **UV** (MeOH): λ_{max} (lg ε) = −. **HRESIMS**: calc. for $C_{10}H_{20}O_3Na$ $[M+Na]^+$ 211.13047; found 211.13049.

3.1.3 Adamantan-1-yl (2*R*,4*R*)-4-hydroxy-2-methylpentanoate (12)

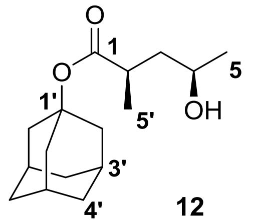

12

Under an argon atmosphere, diisopropylamine (2.98 mL, 21.183 mmol, 2.5 equiv) was dissolved in THF (30 mL) at −78 °C. *n*-BuLi (13.24 mL, 21.183 mmol, 1.6 M in hexane, 2.5 equiv) was added dropwise and the mixture was stirred for 30 min. The adamantyl ester **10** (3.530 g, 16.947 mmol, 2.0 equiv) was added, followed by an additional 30 min of stirring at −78 °C. The reaction was warmed to −45 °C and Et_2AlCl (16.95 mL, 16.947 mmol, 1 M in hexanes, 2.0 equiv) was added dropwise over 5 min. After 15 min of stirring, (*R*)-propylene oxide (594 µL, 8.473 mmol, 1.0 equiv) was added and the reaction was stirred at −40 °C for 5 h. Then, it was quenched by adding saturated aqueous NH_4Cl (10 mL) and the suspension was carefully poured into a mixture of aqueous HCl (6 M, 25 mL) and ice (25 g). After fuming had ceased, the phases were separated and the aqueous phase was further extracted with MTBE (2 × 50 mL). The combined organic phases were washed with saturated aqueous $NaHCO_3$ (2 × 30 mL) and saturated aqueous NaCl (40 mL), dried over $MgSO_4$ and the solvents were removed in vacuo. After column chromatography [silica, PE/EA (5:1)], hydroxy ester **12** (2.255 g, 8.465 mmol, quantitative) was obtained as a pale yellow oil. The product was a mixture of diastereomers at C2, which could not be separated at this stage (dr 4:1).

TLC [PE/EA (5:1)]: R_f = 0.43. $[\alpha]_D^{24}$ = −16.4 (*c* = 0.97, MeOH). **^{1}H NMR** (600 MHz, $CDCl_3$, major diastereomer): δ = 3.89-3.84 (m, 1H, C(4)*H*), 2.61-2.55 (m, 1H, C(2)*H*), 2.18-2.14 (m, 3H, C(3')*H*), 2.12-2.10 (m, 6H, C(2')H_2), 1.72 (ddd, *J* = 13.9 Hz, *J* = 10.0 Hz, *J* = 3.5 Hz, 1H, C(3)H_2), 1.69-1.63 (m, 6H, C(4')H_2), 1.50 (ddd, *J* = 13.9 Hz, *J* = 9.2 Hz, *J* = 4.5 Hz, 1H, C(3)H_2), 1.19 (d, *J* = 6.2 Hz, 3H, C(5)H_3), 1.14 (d, *J* = 7.0 Hz, 3H, C(5')H_3). **^{13}C NMR** (150.9 MHz, $CDCl_3$, major diastereomer): δ = 176.1 (1C, *C*(1)), 80.5 (1C, *C*(1')), 65.8 (1C, *C*(4)H), 43.2 (1C, *C*(3)H_2), 41.3 (3C, *C*(2')H_2), 37.3 (1C, *C*(2)H), 36.1 (3C, *C*(4')H_2), 30.8 (3C, *C*(3')H), 23.6 (1C, *C*(5)H_3), 17.8 (1C, *C*(5')H_3). **^{1}H NMR** (600 MHz, $CDCl_3$, minor diastereomer): δ = 3.88-3.82 (m, 1H, C(4)*H*), 2.48 (dqd, *J* = 7.8 Hz, *J* = 7.0 Hz, *J* = 6.1 Hz, 1H, C(2)*H*), 2.18-2.14 (m, 3H, C(3')*H*), 2.12-2.10 (m, 6H, C(2')H_2), 1.83 (ddd, *J* = 14.0 Hz, *J* = 8.6 Hz, *J* = 8.1 Hz, 1H, C(3)H_2), 1.69-1.63 (m, 6H, C(4')H_2), 1.44 (ddd, *J* = 14.0 Hz, *J* = 6.1 Hz, *J* = 4.3 Hz, 1H, C(3)H_2), 1.21 (d, *J* = 6.2 Hz, 3H, C(5)H_3), 1.14 (d, *J* = 7.0 Hz, 3H, C(5')H_3). **^{13}C NMR** (150.9 MHz, $CDCl_3$, minor diastereomer): δ = 176.4 (1C, *C*(1)), 80.3 (1C, *C*(1')), 66.6 (1C, *C*(4)H), 43.1 (1C, *C*(3)H_2), 41.2 (3C, *C*(2')H_2), 38.2 (1C, *C*(2)H), 36.0 (3C, *C*(4')H_2), 30.8 (3C, *C*(3')H), 24.0 (1C, *C*(5)H_3), 17.8 (1C, *C*(5')H_3). **IR** (ATR): $\tilde{\nu}$ = 3408 cm^{-1} (w, br), 2966 (m), 2908 (m), 2853 (m), 1722 (m), 1706 (m), 1455 (m), 1374 (w), 1351 (w), 1278 (w), 1255 (w), 1221 (w), 1174 (m), 1147 (w), 1120 (w), 1103 (m), 1084 (m), 1053 (m), 1010 (w), 965 (m), 930 (m), 892 (m), 855 (m), 835 (w), 813 (w), 727 (w), 554 (w). **HRESIMS**: calc. for $C_{16}H_{26}O_3Na$ $[M+Na]^+$ 289.17742; found 289.17764.

3.1.4 ***tert*-Butyl (2*R*,4*S*)-4-(4-methoxyphenoxy)-2-methylpentanoate (13a)**

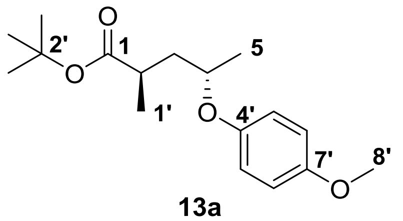

13a

Under an argon atmosphere, hydroxy ester **11** (1.693 g, 8.993 mmol, mixture of diastereomers at C2 with *dr* 3.3:1, 1.0 equiv) was dissolved in degassed THF (125 mL) at room temperature. PPh_3 (4.718 g, 17.986 mmol, 2.0 equiv) and *p*-methoxyphenol (4.466 g, 35.972 mmol, 4.0 equiv) were added and the mixture was cooled to 0 °C. After the dropwise addition of diethyl azodicarboxylate (8.19 mL, 17.986 mmol, 40% in toluene, 2.0 equiv), the mixture was stirred at room temperature for 4 d. The reaction was quenched by the addition of H_2O (50 mL), MTBE (75 mL) was added and the phases were separated. The aqueous phase was further extracted with MTBE (2x 75 mL) and the combined organic phases were washed with H_2O and saturated aqueous NaCl (50 mL each). The combined organic phases were dried over $MgSO_4$ and the solvents were removed in vacuo. Column chromatography [silica, PE/MTBE (20:1)] allowed partial separation of diastereomers. The desired (2*R*,4*S*) diastereomer **13a** (1.332 g, 4.525 mmol, 50%), the minor (2*S*,4*S*) diastereomer **13b** (0.114 g, 0.387 mmol, 4%) and a mixed fraction of both diastereomers (0.851 g, 2.891 mmol, 32%, *dr* 1:1) were each obtained as colorless oils. The total yield added up to 87%.

Major diastereomer **13a**: **TLC** [PE/MTBE (20:1)]: R_f = 0.26. $[\alpha]_D^{29}$ = +8.8 (*c* = 1.30, $CHCl_3$). **^{1}H NMR** (400 MHz, $CDCl_3$): δ = 6.86-6.80 (m, 4H, C(5')*H*, C(6')*H*), 4.27 (ap. dquint, *J* = 7.7 Hz, *J* = 6.1 Hz, 1H, C(4)*H*), 3.76 (s, 3H, C(8')H_3), 2.61-2.52 (m, 1H, C(2)*H*), 2.15 (ddd, *J* = 13.9 Hz, *J* = 7.6 Hz, *J* = 7.6 Hz, 1H, C(3)H_2), 1.52 (ddd, *J* = 13.9 Hz, *J* = 6.8 Hz, *J* = 5.5 Hz, 1H, C(3)H_2), 1.44 (s, 9H, C(3')H_3), 1.27 (d, *J* = 6.1 Hz, 3H, C(5)H_3), 1.14 (d, *J* = 7.0 Hz, 3H, C(1')H_3). **^{13}C NMR** (100.6 MHz, $CDCl_3$): δ = 175.9 (1C, *C*(1)), 154.0 (1C,

C(7')), 152.0 (1C, C(4')), 117.5 + 114.6 (4C, C(5')H, C(6')H), 80.0 (1C, C(2')), 72.9 (1C, C(4)H), 55.7 (1C, C(8')H$_3$), 40.2 (1C, C(3)H$_2$), 37.3 (1C, C(2)H), 28.1 (3C, C(3')H$_3$), 19.8 (1C, C(5)H$_3$), 17.5 (1C, C(1')H$_3$). Minor diastereomer **13b**: **TLC** [PE/MTBE (20:1)]: R_f = 0.30. $[\alpha]_D^{29}$ = +35.3 (*c* = 1.20, $CHCl_3$). **^{1}H NMR** (400 MHz, $CDCl_3$): δ = 6.85-6.79 (m, 4H, C(5')*H*, C(6')*H*), 4.26-4.19 (m, 1H, C(4)*H*), 3.76 (s, 3H, C(8')*H*$_3$), 2.72-2.63 (m, 1H, C(2)*H*), 1.89 (ddd, *J* = 13.6 Hz, *J* = 9.8 Hz, *J* = 3.7 Hz, 1H, C(3)*H*$_2$), 1.71 (ddd, *J* = 13.9 Hz, *J* = 9.2 Hz, *J* = 4.5 Hz, 1H, C(3)*H*$_2$), 1.39 (s, 9H, C(3')*H*$_3$), 1.24 (d, *J* = 6.1 Hz, 3H, C(5)*H*$_3$), 1.17 (d, *J* = 7.1 Hz, 3H, C(1')*H*$_3$). **^{13}C NMR** (100.6 MHz, $CDCl_3$): δ = 176.1 (1C, C(1)O), 154.0 (1C, C(7')), 152.3 (1C, C(4')), 117.8 + 114.6 (4C, C(5')H, C(6')H), 80.0 (1C, C(2')), 73.9 (1C, C(4)H), 55.7 (1C, C(8')H$_3$), 41.3 (1C, C(3)H$_2$), 37.2 (1C, C(2)H), 28.0 (3C, C(3')H$_3$), 20.2 (1C, C(5)H$_3$), 18.2 (1C, C(1')H$_3$). **IR** (ATR): $\tilde{\nu}$ = 2974 cm^{-1} (m), 2935 (m), 2834 (w), 1723 (m), 1504 (s), 1459 (m), 1367 (m), 1288 (w), 1225 (s), 1148 (s), 1105 (m), 1083 (m), 1037 (m), 1009 (m), 961 (w), 930 (m), 846 (m), 826 (m), 736 (m). **UV** (CH_2Cl_2): λ_{max} (lg ε) = 290 nm (3.38), 230 (3.87). **HRESIMS**: calc. for $C_{17}H_{26}O_4Na$ [M+Na]$^+$ 317.17233; found 317.17250.

3.1.5 Adamantan-1-yl (2*R*,4*S*)-4-(4-methoxyphenoxy)-2-methylpentanoate (14a)

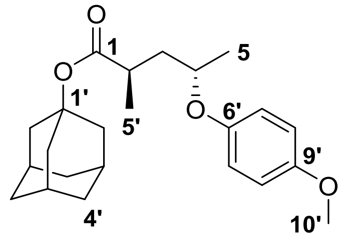

14a

Under an argon atmosphere, hydroxy ester **12** (2.212 g, 8.304 mmol, mixture of diastereomers at C2 with dr 4:1, 1.0 equiv) was dissolved in degassed THF (125 mL) at room temperature. PPh_3 (4.356 g, 16.608 mmol, 2.0 equiv) and *p*-methoxyphenol (4.123 g, 33.216 mmol, 4.0 equiv) were added and the mixture was cooled to 0 °C. After the dropwise addition of diethyl azodicarboxylate (7.56 mL, 16.608 mmol, 40% in toluene, 2.0 equiv), the mixture was stirred at room temperature for 24 h. The reaction was quenched by the addition of H_2O (100 mL). MTBE (125 mL) was added and the phases were separated. The aqueous phase was further extracted with MTBE (3 × 100 mL) and the combined organic phases were washed with H_2O and saturated aqueous NaCl (50 mL each). The combined organic phases were dried over $MgSO_4$ and the solvents were removed in vacuo. Column chromatography [silica, PE/MTBE (20:1)] delivered the phenyl ether **14** (2.312 g, 6.207 mmol, 75%) as a colorless oil. The compound was obtained as a mixture of diastereomers (dr 4:1, **14a**:**14b**). Separation of diastereomers was not attempted at this step.

$[\alpha]_D^{24}$ = +1.1 (*c* = 1.41, *dr* 4:1, MeOH). Major diastereomer **14a**: **TLC** [PE/MTBE (20:1)]: R_f = 0.26. **^{1}H NMR** (600 MHz, $CDCl_3$): δ = 6.86-6.80 (m, 4H, C(7')*H*, C(8')*H*), 4.27 (ap. dquint, *J* = 7.3 Hz, *J* = 6.0 Hz, 1H, C(4)*H*), 3.76 (s, 3H, C(10')*H*$_3$), 2.58-2.52 (m, 1H, C(2)*H*), 2.17-2.12 (m, 3H, C(3')*H*), 2.15 (ddd, *J* = 14.0 Hz, *J* = 7.6 Hz, *J* = 7.6 Hz, 1H, C(3)*H*$_2$), 2.11-2.07 (m, 6H, C(2')*H*$_2$), 1.67-1.64 (m, 6H, C(4')*H*$_2$), 1.51 (ddd, *J* = 13.9 Hz, *J* = 6.6 Hz, *J* = 5.8 Hz, 1H, C(3)*H*$_2$), 1.28 (d, *J* = 6.1 Hz, 3H, C(5)*H*$_3$), 1.14 (d, *J* = 7.0 Hz, 3H, C(5')*H*$_3$). **^{13}C NMR** (150.9 MHz, $CDCl_3$): δ = 175.7 (1C, C(1)), 153.9 (1C, C(9')), 151.9 (1C, C(6')), 117.5 + 114.6 (4C, C(7')H, C(8')H), 80.1 (1C, C(1')), 72.8 (1C, C(4)H), 55.7 (1C, C(10')H$_3$), 41.3 (3C, C(2')H$_2$), 40.2 (1C, C(3)H$_2$), 37.4 (1C, C(2)H), 36.2 (3C, C(4')H$_2$), 30.8 (3C, C(3')H), 19.9 (1C, C(5)H$_3$), 17.6 (1C, C(5')H$_3$). Minor diastereomer **14b**: **TLC** [PE/MTBE (20:1)]: R_f = 0.32. **^{1}H NMR** (600 MHz, $CDCl_3$): δ = 6.86-6.80 (m, 4H, C(7')*H*, C(8')*H*), 4.25-4.20 (m, 1H, C(4)*H*), 3.76 (s, 3H, C(8')*H*$_3$), 2.66 (dqd, *J* = 10.0 Hz, *J* = 7.1 Hz, *J* = 4.4 Hz, 1H, C(2)*H*), 2.17-2.12 (m, 3H, C(3')*H*), 2.11-2.07 (ap. d, *J* = 3.2 Hz, 6H, C(2')*H*$_2$), 1.87 (ddd, *J* = 13.8 Hz,

J = 10.0 Hz, J = 3.6 Hz, 1H, C(3)H_2), 1.70 (ddd, J = 13.8 Hz, J = 9.2 Hz, J = 4.3 Hz, 1H, C(3)H_2), 1.64-1.62 (m, 6H, C(4')H_2), 1.24 (d, J = 6.1 Hz, 3H, C(5)H_3), 1.16 (d, J = 7.1 Hz, 3H, C(5')H_3). **^{13}C NMR** (150.9 MHz, $CDCl_3$): δ = 175.9 (1C, *C*(1)), 154.0 (1C, *C*(9')), 152.4 (1C, *C*(6')), 117.9 + 114.6 (4C, *C*(7')H, *C*(8')H), 80.1 (1C, *C*(1')), 74.1 (1C, *C*(4)H), 55.7 (1C, *C*(10')H_3), 41.4 (1C, *C*(3)H_2), 41.2 (3C, *C*(2')H_2), 37.3 (1C, *C*(2)H), 36.2 (3C, *C*(4')H_2), 30.8 (3C, *C*(3')H),20.3 (1C, *C*(5)H_3), 18.3 (1C, *C*(5')H_3). **IR** (ATR): $\tilde{\nu}$ = 2970 cm^{-1} (w), 2910 (m), 2854 (m), 1723 (m), 1504 (m), 1457 (m), 1376 (w), 1351 (m), 1281 (w), 1224 (m), 1178 (m), 1130 (m), 1103 (m), 1037 (m), 1008 (m), 965 (m), 937 (w), 896 (m), 825 (m), 735 (m). **UV** (CH_2Cl_2): λ_{max} (lg ε) = 291 nm (3.41). **HRESIMS**: calc. for $C_{23}H_{32}O_4Na$ $[M+Na]^+$ 395.21928; found 395.21949.

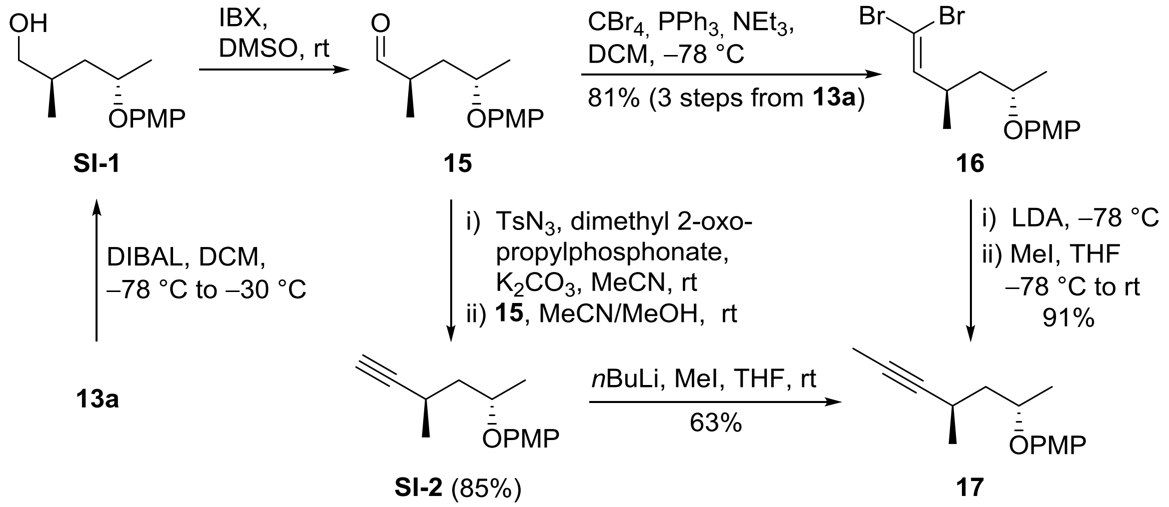

3.1.6 (2*R*,4*S*)-4-(4-Methoxyphenoxy)-2-methylpentan-1-ol (SI-1)

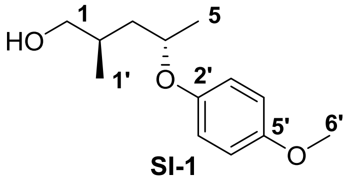

SI-1

Under an argon atmosphere, the protected hydroxy ester **13a** (1.412 g, 4.796 mmol, 1.0 equiv) was dissolved in DCM (40 mL) at room temperature. The solution was cooled to −78 °C and DIBAL-H (11.99 mL, 11.991 mmol, 1 M in toluene, 2.5 equiv) was added. After 3 h of stirring at −78 °C, the mixture was warmed to −50 °C and additional DIBAL-H (2.40 mL, 2.398 mmol, 1 M in toluene, 0.5 equiv) was added. During the following 2 h of stirring, the mixture was warmed to −30 °C. The reaction was then quenched by dropwise addition of H_2O (1 mL) and $MgSO_4$ was added in small portions until drying was visually achieved. The solids were filtered off with a porous frit (porosity 4), the filter cake was further washed with DCM (3 × 5 mL) and the filtrate was concentrated in vacuo. After column chromatography [silica, PE/EA (5:1 → 2:1)], alcohol **SI-1** (0.864 g, 3.852 mmol, 80%) was obtained as a colorless oil. The column chromatography was not mandatory and by direct conversion of the crude material to the dibromoalkene **16**, a higher total yield of **16** could be achieved (see 3.1.8).

TLC [PE/EA (2:1)]: R_f = 0.43. $[\alpha]_D^{26}$ = +48.2 (*c* = 1.24, $CHCl_3$). **^{1}H NMR** (400 MHz, $CDCl_3$): δ = 6.87-6.80 (m, 4H, C(3')*H*, C(4')*H*), 4.38-4.31 (m, 1H, C(4)*H*), 3.77 (s, 3H, C(6')H_3), 3.55-3.48 (m, 2H, C(1)H_2), 1.98-1.87 (m, 1H, C(2)*H*), 1.85 (ddd, J = 14.3 Hz, J = 8.9 Hz, J = 5.8 Hz, 1H, C(3)H_2), 1.78 (br s, 1H, O*H*), 1.39 (ddd, J = 14.1 Hz, J = 7.5 Hz, J = 3.4 Hz,

1H, C(3)H_2), 1.26 (d, J = 6.0 Hz, 3H, C(5)H_3), 0.97 (d, J = 6.7 Hz, 3H, C(1')H_3). **^{13}C NMR** (100.6 MHz, $CDCl_3$): δ = 154.0 (1C, C(5')), 151.8 (1C, C(2')), 117.4 + 114.7 (4C, C(3')H, C(4')H), 73.5 (1C, C(4)H), 68.3 (1C, C(1)H_2), 55.7 (1C, C(6')H_3), 41.0 (1C, C(3)H_2), 33.2 (1C, C(2)H), 20.4 (1C, C(5)H_3), 17.3 (1C, C(1')H_3). **IR** (ATR): $\tilde{\nu}$ = 3378 cm^{-1} (w, br), 2967 (m), 2930 (m), 2874 (m), 2835 (w), 1503 (s), 1463 (m), 1376 (m), 1289 (w), 1223 (s), 1179 (m), 1119 (m), 1033 (s), 957 (m), 914 (m), 883 (w), 825 (m), 734 (m). **UV** (CH_2Cl_2): λ_{max} (lg ε) = 291 nm (3.39), 231 (3.87). **MS** (EI, 70 eV): m/z (%) = 224 (5) $[M]^{+\bullet}$, 151 (3), 124 (100), 109 (40), 95 (3), 81 (5), 55 (5). **HREIMS**: calc. for $C_{13}H_{20}O_3$ $[M]^{+\bullet}$ 224.14070; found 224.14298.

3.1.7 (2*R*,4*S*)-4-(4-Methoxyphenoxy)-2-methylpentanal (15)

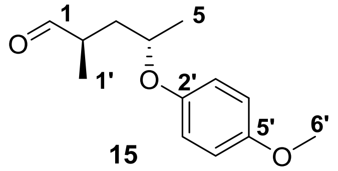

15

At room temperature, the alcohol **SI-1** (0.301 g, 1.342 mmol, 1.0 equiv) was dissolved in DMSO (10 mL). 2-iodoxybenzoic acid (1.127 g, 4.026 mmol, 3.0 equiv) was added and the mixture was stirred for 40 min. The reaction was quenched with H_2O (10 mL) and after 5 min of stirring, the solids were filtered off with a porous frit (porosity 4). The filter cake was washed with H_2O and MTBE (3x 5 mL each). The phases of the filtrate were separated and the aqueous phase was further extracted with MTBE (3 × 20 mL). The combined organic phases were washed with saturated aqueous $NaHCO_3$ and H_2O (2 × 20 mL each), dried over Na_2SO_4 and the solvent was removed in vacuo. After column chromatography [silica, PE/EA (5:1)], the aldehyde **15** (0.266 g, 1.179 mmol, 89%) was obtained as a pale yellow oil. The column chromatography was not mandatory and by direct conversion of the crude material to the dibromoalkene **16**, a higher total yield of **16** could be achieved (see 3.1.8).

TLC [PE/EA (5:1)]: R_f = 0.67. $[\alpha]_D^{26}$ = +30.3 (c = 1.20, $CHCl_3$). **^{1}H NMR** (400 MHz, $CDCl_3$): δ = 9.65 (d, J = 2.1 Hz, 1H, C(1)HO), 6.82 (s, 4H, C(3')H, C(4')H), 4.35-4.27 (m, 1H, C(4)H), 3.76 (s, 3H, C(6')H_3), 2.63 (sextd, J = 7.0 Hz, J = 2.1 Hz, 1H, C(2)H), 2.19 (ddd, J = 14.3 Hz, J = 9.2 Hz, J = 6.7 Hz, 1H, C(3)H_2), 1.54 (ddd, J = 14.3 Hz, J = 7.1 Hz, J = 3.7 Hz, 1H, C(3)H_2), 1.28 (d, J = 6.0 Hz, 3H, C(5)H_3), 1.13 (d, J = 7.1 Hz, 3H, C(1')H_3). **^{13}C NMR** (100.6 MHz, $CDCl_3$): δ = 204.4 (1C, C(1)HO), 154.1 (1C, C(5')), 151.6 (1C, C(2')), 117.4 + 114.7 (4C, C(3')H, C(4')H), 72.7 (1C, C(4)H), 55.7 (1C, C(6')H_3), 43.8 (1C, C(2)H), 38.1 (1C, C(3)H_2), 20.0 (1C, C(5)H_3), 13.8 (1C, C(1')H_3). **IR** (ATR): $\tilde{\nu}$ = 2972 cm^{-1} (w), 2934 (w), 2834 (w), 2718 (w), 1723 (m), 1504 (m), 1461 (m), 1377 (w), 1289 (w), 1225 (m), 1180 (w), 1122 (m), 1105 (m), 1037 (m), 928 (w), 827 (m), 734 (m), 574 (w). **UV** (CH_2Cl_2): λ_{max} (lg ε) = 290 nm (3.37), 230 (3.86). **HRESIMS**: calc. for $C_{13}H_{18}O_3Na$ $[M+Na]^+$ 245.11482; found 245.11473.

3.1.8 1-(((2*S*,4*R*)-6,6-Dibromo-4-methylhex-5-en-2-yl)oxy)-4-methoxybenzene (16)

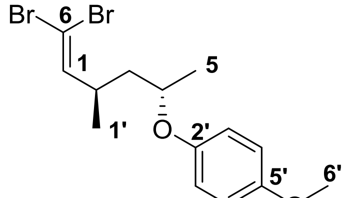

16

At room temperature, PPh_3 (12.631 g, 48.155 mmol, 4.0 equiv) was dissolved in DCM (100 mL). After the addition of CBr_4 (7.985 g, 24.078 mmol, 2.0 equiv), the mixture was cooled to −78 °C and a solution of the aldehyde **15** (2.676 g, crude product resulting from the transformation of 3.940 g (13.384 mmol) of the ester **13a** following 3.1.6 and 3.1.7 without purification steps) and NEt_3 (1.68 mL, 12.039 mmol, 1.0 equiv) in DCM (20 mL) was added

dropwise. After 90 min of stirring at −78 °C, the mixture was warmed to room temperature. *n*-Hexane (150 mL) was added and after 10 min of stirring, the precipitated solid was removed by filtration through a silica-filled, porous frit (porosity 4). The filter cake was washed with a mixture of *n*-hexane/EtOAc (3:1, 3 × 50 mL) and the solvents were removed in vacuo. After column chromatography [silica, PE/EA (20:1)], the dibromoolefin **16** (4.079 g, 10.788 mmol, 81% over 3 steps) was obtained as a yellow oil.

TLC [PE/EA (20:1)]: R_f = 0.47. $[\alpha]_D^{26}$ = +14.4 (*c* = 1.08, $CHCl_3$). **^{1}H NMR** (400 MHz, $CDCl_3$): δ = 6.83 (s, 4H, C(3')*H*, C(4')*H*), 6.25 (d, *J* = 9.6 Hz, 1H, C(1)*H*), 4.26-4.18 (m, 1H, C(4)*H*), 3.77 (s, 3H, C(6')H_3), 2.73-2.62 (m, 1H, C(2)*H*), 1.83 (ddd, *J* = 13.8 Hz, *J* = 8.5 Hz, *J* = 6.7 Hz, 1H, C(3)H_2), 1.53 (ddd, *J* = 13.8 Hz, *J* = 6.1 Hz, *J* = 6.1 Hz, 1H, C(3)H_2), 1.29 (d, *J* = 6.0 Hz, 3H, C(5)H_3), 1.04 (d, *J* = 6.7 Hz, 3H, C(1')H_3). **^{13}C NMR** (100.6 MHz, $CDCl_3$): δ = 153.9 (1C, *C*(5')), 151.8 (1C, *C*(2')), 143.7 (1C, *C*(1)H), 117.3 + 114.7 (4C, *C*(3')H, *C*(4')H), 87.6 (1C, *C*(6)Br_2), 72.8 (1C, *C*(4)H), 55.7 (1C, *C*(6')H_3), 42.8 (1C, *C*(3)H_2), 35.5 (1C, *C*(2)H), 20.1 (1C, *C*(5)H_3), 19.4 (1C, *C*(1')H_3). **IR** (ATR): $\tilde{\nu}$ = 2967 cm^{-1} (m), 2930 (m), 2833 (w), 1614 (w), 1503 (s), 1459 (m), 1377 (m), 1289 (w), 1225 (s), 1179 (m), 1120 (m), 1037 (m), 964 (w), 936 (m), 825 (m), 783 (m), 735 (m), 569 (w). **UV** (CH_2Cl_2): λ_{max} (lg ε) = 291 nm (3.41), 230 (3.93). **HRESIMS**: calc. for $C_{14}H_{18}Br_2O_2Na$ $[M+Na]^+$ 400.95453; found 400.95470.

3.1.9 1-Methoxy-4-(((2*S*,4*R*)-4-methylhept-5-in-2-yl)oxy)benzene (17)

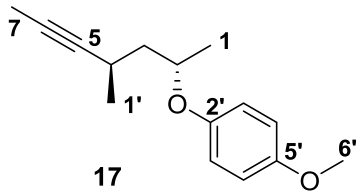

17

Under an argon atmosphere, diisopropylamine (5.72 mL, 40.470 mmol, 10.0 equiv) was dissolved in THF (130 mL) at room temperature. The solution was cooled to −20 °C and *n*-BuLi (25.29 mL, 40.470 mmol, 10.0 equiv) was added, followed by 20 min of stirring at −20 °C. Then, the mixture was cooled to −78 °C and a solution of the dibromoolefin **16** (1.530 g, 4.047 mmol, 1.0 equiv) in THF (20 mL) was added dropwise. After 2 h of stirring at −78 °C, iodomethane (5.04 mL, 80.940 mmol, 20.0 equiv) was added and the mixture was slowly brought to room temperature over 16 h. After quenching with saturated aqueous NH_4Cl (20 mL), MTBE (100 mL) was added, the phases were separated and the aqueous phase was further extracted with MTBE (2 × 75 mL). The combined organic phases were dried over $MgSO_4$ and the solvents were removed in vacuo. Column chromatography [silica, PE/DCM (3:1)] delivered the internal alkyne **17** (0.860 g, 3.702 mmol, 91%) as a colorless oil.

TLC [PE/EA (20:1)]: R_f = 0.51. $[\alpha]_D^{26}$ = +34.7 (*c* = 1.09, $CHCl_3$). **^{1}H NMR** (400 MHz, $CDCl_3$): δ = 6.89-6.80 (m, 4H, C(3')*H*, C(4')*H*), 4.45 (dquint, *J* = 7.3 Hz, *J* = 6.0 Hz, 1H, C(2)*H*), 3.77 (s, 3H, C(6')H_3), 2.58-2.47 (m, 1H, C(4)*H*), 1.95 (ddd, *J* = 13.3 Hz, *J* = 9.1 Hz, *J* = 5.7 Hz, 1H, C(3)H_2), 1.81 (d, *J* = 2.4 Hz, 3H, C(7)H_3), 1.51 (ddd, *J* = 13.4 Hz, *J* = 7.5 Hz, *J* = 6.0 Hz, 1H, C(3)H_2), 1.29 (d, *J* = 6.1 Hz, 3H, C(1)H_3), 1.16 (d, *J* = 6.9 Hz, 3H, C(1')H_3). **^{13}C NMR** (100.6 MHz, $CDCl_3$): δ = 153.8 (1C, *C*(5')), 152.0 (1C, *C*(2')), 117.1 + 114.7 (4C, *C*(3')H, *C*(4')H), 83.0 (1C, *C*(5)), 76.0 (1C, *C*(6)), 72.7 (1C, *C*(2)H), 55.7 (1C, *C*(6')H_3), 43.4 (1C, *C*(3)H_2), 22.7 (1C, *C*(4)H), 21.5 (1C, *C*(1')H_3), 19.6 (1C, *C*(1)H_3), 3.5 (1C, *C*(7)H_3). **IR** (ATR): $\tilde{\nu}$ = 2969 cm^{-1} (w), 2929 (w), 2834 (w), 1505 (m), 1462 (w), 1378 (w), 1289 (w), 1228 (m), 1179 (w), 1108 (m), 1067 (w), 1039 (m), 949 (w), 825 (m), 738 (w). **UV** (CH_2Cl_2): λ_{max} (lg ε) =

292 nm (3.35), 230 (3.85). **MS** (EI, 70 eV): *m/z* (%) = 232 (14) $[M]^{+\bullet}$, 221 (5), 207 (8), 193 (2), 147 (3), 124 (100), 109 (28), 81 (5), 67 (10). **HREIMS**: calc. for $C_{15}H_{20}O_2$ $[M]^{+\bullet}$ 232.14578; found 232.14521.

3.1.10 1-Methoxy-4-(((2*S*,4*R*)-4-methylhex-5-in-2-yl)oxy)benzene (SI-2)

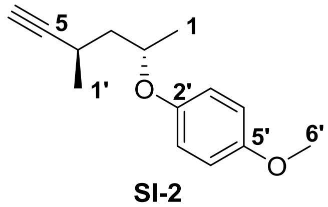

SI-2

At room temperature, tosyl azide (0.166 g, 0.844 mmol, 1.25 equiv) and K_2CO_3 (0.280 g, 2.024 mmol, 3.0 equiv) were dissolved in MeCN (10 mL) and dimethyl 2-oxopropyl phosphonate (112 µL, 0.810 mmol, 1.2 equiv) was added. After 2 h of stirring, a solution of the aldehyde **15** (0.150 g, 0.675 mmol, *dr* 4:1 at C4, 1.0 equiv) in MeOH (2 mL) was added dropwise and the mixture was stirred at room temperature for 20 h. The solvents were removed in vacuo and the residue was dissolved in MTBE and H_2O (20 mL each). The phases were separated and the aqueous phase was further extracted with MTBE (2 × 10 mL). The combined organic phases were washed with H_2O and saturated aqueous NaCl (10 mL each), dried over $MgSO_4$ and the solvents were removed in vacuo. The residue was washed with pentane (5 × 10 mL). The organic phases were collected and filtered. After removal of the solvent in vacuo, the terminal alkyne **SI-2** (0.125 g, 0.573 mmol, dr 4:1 at C4 as in the starting material **15**, 85%) was obtained as a pale yellow oil.

TLC [PE/EA (20:1)]: R_f = 0.47. $[\alpha]_D^{26}$ = +30.7 (*c* = 0.97, $CHCl_3$). **^{1}H NMR** (400 MHz, $CDCl_3$, major diastereomer): δ = 6.91-6.80 (m, 4H, C(3')*H*, C(4')*H*), 4.52-4.42 (m, 1H, C(2)*H*), 3.77 (s, 3H, C(6')H_3), 2.66-2.57 (m, 1H, C(4)*H*), 2.08 (d, *J* = 2.5 Hz, 1H, C(6)*H*), 2.02 (ddd, *J* = 13.5 Hz, *J* = 8.7 Hz, *J* = 6.1 Hz, 1H, C(3)H_2), 1.57 (ddd, *J* = 13.4 Hz, *J* = 7.1 Hz, *J* = 6.4 Hz, 1H, C(3)H_2), 1.30 (d, *J* = 6.1 Hz, 3H, C(1)H_3), 1.22 (d, *J* = 6.9 Hz, 3H, C(1')H_3). **^{13}C NMR** (100.6 MHz, $CDCl_3$, major diastereomer): δ = 153.9 (1C, *C*(5')), 151.8 (1C, *C*(2')), 117.2 + 114.7 (4C, *C*(3')H, *C*(4')H), 88.3 (1C, *C*(5)), 72.5 (1C, *C*(2)H), 68.6 (1C, *C*(6)H), 55.7 (1C, *C*(6')H_3), 42.9 (1C, *C*(3)H_2), 22.4 (1C, *C*(4)H), 21.0 (1C, *C*(1')H_3), 19.6 (1C, *C*(1)H_3). **^{1}H NMR** (400 MHz, $CDCl_3$, minor diastereomer): δ = 6.91-6.80 (m, 4H, C(3')*H*, C(4')*H*), 4.52-4.42 (m, 1H, C(2)*H*), 3.77 (s, 3H, C(6')H_3), 2.85-2.76 (m, 1H, C(4)*H*), 2.06 (d, *J* = 2.4 Hz, 1H, C(6)*H*), 1.79 (ddd, *J* = 13.9 Hz, *J* = 9.8 Hz, *J* = 4.2 Hz, 1H, C(3)H_2), 1.65-1.58 (m, 1H, C(3)H_2), 1.25 (d, *J* = 6.1 Hz, 3H, C(1)H_3), 1.23 (d, *J* = 7.0 Hz, 3H, C(1')H_3). **^{13}C NMR** (100.6 MHz, $CDCl_3$, minor diastereomer): δ = 154.1 (1C, *C*(5')), 152.3 (1C, *C*(2')), 118.1 + 114.6 (4C, *C*(3')H, *C*(4')H), 88.6 (1C, *C*(5)), 73.9 (1C, *C*(2)H), 68.8 (1C, *C*(6)H), 55.7 (1C, *C*(6')H_3), 44.5 (1C, *C*(3)H_2), 22.8 (1C, *C*(4)H), 21.3 (1C, *C*(1')H_3), 20.1 (1C, *C*(1)H_3). **IR** (ATR): $\tilde{\nu}$ = 3292 cm^{-1} (w), 2973 (m), 2934 (m), 2834 (w), 1503 (s), 1460 (m), 1379 (m), 1289 (w), 1217 (s), 1180 (m), 1127 (m), 1090 (m), 1035 (s), 958 (m), 936 (m), 825 (m), 734 (m), 632 (m). **UV** (CH_2Cl_2): λ_{max} (lg ε) = 291 nm (3.39). **MS** (EI, 70 eV): *m/z* (%) = 218 (18) $[M]^{+\bullet}$, 124 (100), 109 (45), 95 (6), 81 (5), 67 (3), 53 (7). **HREIMS**: calc. for $C_{14}H_{18}O_2$ $[M]^{+\bullet}$ 218.13013; found 218.13221.

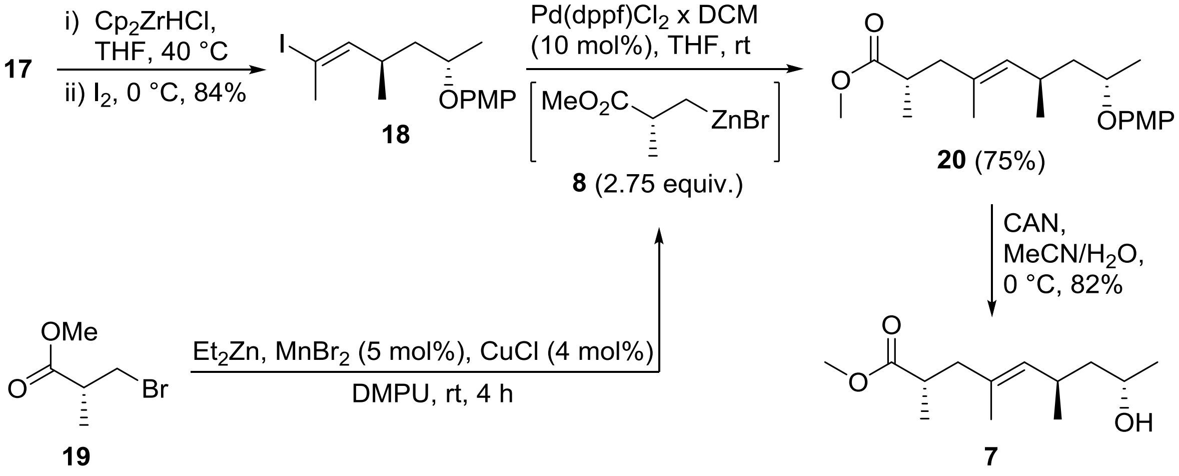

3.1.11 1-(((2*S*,4*R*,*E*)-6-Iodo-4-methylhept-5-en-2-yl)oxy)-4-methoxybenzene (18)

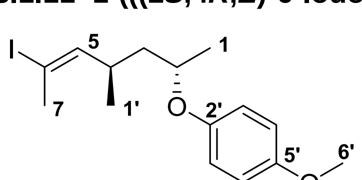

18

At room temperature, the internal alkyne **17** (0.375 g, 1.614 mmol, 1.0 equiv) was dissolved in THF (35 mL) under an argon atmosphere. Cp_2ZrHCl (Schwartz reagent, 1.249 g, 4.842 mmol, 3.0 equiv) was added, the suspension was warmed to 40 °C and stirred for 3 h. Then, the mixture was cooled to 0 °C and a solution of iodine (1.229 g, 4.842 mmol, 3.0 equiv) in THF (15 mL) was added dropwise. After completion of the addition, half-concentrated aqueous $Na_2S_2O_3$ (40 mL) was added to end the reaction. MTBE (50 mL) was added, the phases were separated and the aqueous phase was further extracted with MTBE (3 × 30 mL). The combined organic phases were washed with saturated aqueous NaCl (50 mL), dried over $MgSO_4$ and the solvents were removed in vacuo. Purification by column chromatography [silica, PE/EA (20:1)] delivered the (*E*)-iodoalkene **18** (0.487 g, 1.352 mmol, 84%) as a colorless oil.

TLC [PE/EA (20:1)]: R_f = 0.58. $[\alpha]_D^{29}$ = −4.5 (*c* = 1.30, $CHCl_3$). **^{1}H NMR** (400 MHz, $CDCl_3$): δ = 6.85-6.79 (m, 4H, C(3')*H*, C(4')*H*), 6.02 (dd, *J* = 9.9 Hz, *J* = 1.5 Hz, 1H, C(5)*H*), 4.26-4.18 (m, 1H, C(2)*H*), 3.77 (s, 3H, C(6')H_3), 2.67-2.56 (m, 1H, C(4)*H*), 2.39 (d, *J* = 1.5 Hz, 3H, C(7)H_3), 1.76 (ddd, *J* = 14.0 Hz, *J* = 7.5 Hz, *J* = 7.1 Hz, 1H, C(3)H_2), 1.48-1.41 (m, 1H, C(3)H_2), 1.25 (d, *J* = 6.0 Hz, 3H, C(1)H_3), 0.98 (d, *J* = 6.7 Hz, 3H, C(1')H_3). **^{13}C NMR** (100.6 MHz, $CDCl_3$): δ = 153.9 (1C, *C*(5')), 151.9 (1C, *C*(2')), 146.7 (1C, *C*(5)H), 117.2 + 114.7 (4C, *C*(3')H, *C*(4')H), 92.8 (1C, *C*(6)I), 72.5 (1C, *C*(2)H), 55.7 (1C, *C*(6')H_3), 43.5 (1C, *C*(3)H_2), 32.4 (1C, *C*(4)H), 27.7 (1C, *C*(7)H_3), 20.3 (1C, *C*(1')H_3), 20.0 (1C, *C*(1)H_3). **IR** (ATR): $\tilde{\nu}$ = 2966 cm^{-1} (m), 2928 (m), 2870 (w), 2833 (w), 1634 (w), 1504 (s), 1460 (m), 1377 (m), 1289 (w), 1227 (s), 1179 (m), 1123 (m), 1038 (m), 952 (w), 916 (w), 825 (m), 736 (m), 644 (w). **UV** (CH_2Cl_2): λ_{max} (lg ε) = 291 nm (3.42). **MS** (EI, 70 eV): *m/z* (%) = 360 (32) $[M]^{+\bullet}$, 194 (25), 180 (9), 151 (2), 124 (100), 109 (30), 95 (6), 77 (4), 67 (13). **HREIMS**: calc. for $C_{15}H_{21}O_2I$ $[M]^{+\bullet}$ 360.05862; found 360.06023.

3.1.12 Methyl (2*S*,6*R*,8*S*,*E*)-8-(4-methoxyphenoxy)-2,4,6-trimethylnon-4-enoate (20)

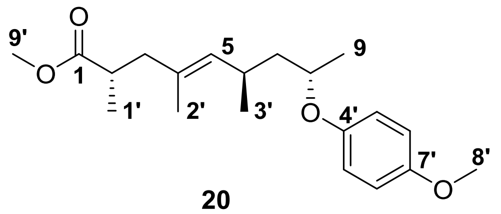

20

a) Generation of the organozinc species **8**

At room temperature, $MnBr_2$ (40.7 mg, 0.189 mmol, 15 mol %) was dissolved in DMPU (3 mL) under an argon atmosphere. CuCl (12.5 mg, 0.126 mmol, 10 mol %), methyl (*R*)-(+)-3-bromo-2-methylpropionate (**19**, 482 µL, 3.787 mmol, 3.0 equiv) and Et_2Zn (3.47 mL, 3.472 mmol, 2.75 equiv) were added in rapid succession. The resulting, dark red suspension was stirred at room temperature for 4 h. Then, it was used for the cross coupling reaction without further purification or filtration.

b) Negishi coupling

At room temperature, the iodoalkene **18** (454.2 mg, 1.261 mmol, 1.0 equiv) and $Pd(dppf)Cl_2$ · DCM (103.1 mg, 0.126 mmol, 10 mol %) were dissolved in THF (20 mL) under an argon atmosphere. Then, the suspension of organozinc species **8** obtained in a) was slowly added and the mixture was stirred at room temperature for 15 h. It was then diluted with MTBE (30 mL) and water (30 mL), the phases were separated and the aqueous phase was further extracted with MTBE (2 × 40 mL). The combined organic phases were washed with H_2O and saturated aqueous NaCl (25 mL each), dried over $MgSO_4$ and the solvents were removed in vacuo. After column chromatography [silica, PE/EA (30:1)], the protected polyketide **20** (314.4 mg, 0.940 mmol, 75%) was obtained as a colorless oil.

TLC [PE/EA (20:1)]: R_f = 0.34. $[\alpha]_D^{22}$ = +15.5 (*c* = 1.19, MeOH). **^{1}H NMR** (600 MHz, $CDCl_3$): δ = 6.83-6.79 (m, 4H, C(5')*H*, C(6')*H*), 4.98 (dq, *J* = 9.6 Hz, *J* = 1.1 Hz, 1H, C(5)*H*), 4.19 (dq, *J* = 7.3 Hz, *J* = 6.0 Hz, 1H, C(8)*H*), 3.77 (s, 3H, C(8')H_3), 3.64 (s, 3H, C(9')H_3), 2.67-2.61 (m, 1H, C(2)*H*), 2.56-2.48 (m, 1H, C(6)*H*), 2.36 (ddd, *J* = 13.5 Hz, *J* = 7.6 Hz, *J* = 1.0 Hz, 1H, C(3)H_2), 2.04 (ddd, *J* = 13.5 Hz, *J* = 7.6 Hz, *J* = 0.9 Hz, 1H, C(3)H_2), 1.72 (ddd, *J* = 13.6 Hz, *J* = 8.7 Hz, *J* = 5.9 Hz, 1H, C(7)H_2), 1.62 (d, *J* = 1.4 Hz, 3H, C(2')H_3), 1.43 (ddd, *J* = 13.4 Hz, *J* = 7.4 Hz, *J* = 6.0 Hz, 1H, C(7)H_2), 1.24 (d, *J* = 6.0 Hz, 3H, C(9)H_3), 1.12 (d, *J* = 6.9 Hz, 3H, C(1')H_3), 0.92 (d, *J* = 6.7 Hz, 3H, C(3')H_3). **^{13}C NMR** (150.9 MHz, $CDCl_3$): δ = 177.0 (1C, *C*(1)O), 153.7 (1C, *C*(7')), 152.0 (1C, *C*(4')), 133.1 (1C, *C*(5)H), 131.0 (1C, *C*(4)), 117.2 + 114.6 (4C, *C*(5')H, *C*(6')H), 73.0 (1C, *C*(8)H), 55.7 (1C, *C*(8')H_3), 51.4 (1C, *C*(9')H_3), 44.1 (1C, *C*(3)H_2), 44.0 (1C, *C*(7)H_2), 38.0 (1C, *C*(2)H), 29.2 (1C, *C*(6)H), 21.3 (1C, *C*(3')H_3), 19.8 (1C, *C*(9)H_3), 16.7 (1C, *C*(1')H_3), 15.9 (1C, *C*(2')H_3). **IR** (ATR): $\tilde{\nu}$ = 2953 cm^{-1} (m), 2930 (m), 2871 (w), 1736 (m), 1504 (s), 1457 (m), 1376 (m), 1287 (w), 1225 (s), 1165 (m), 1107 (m), 1038 (m), 942 (w), 825 (m), 737 (m), 581 (w). **UV** (CH_2Cl_2): λ_{max} (lg ε) = 292 nm (3.34), 230 (3.84). **MS** (EI, 70 eV): *m/z* (%) = 334 (13) $[M]^{+\bullet}$, 211 (3), 179 (12), 151 (8), 124 (100), 109 (26), 95 (11), 81 (8), 55 (5). **HREIMS**: calc. for $C_{20}H_{30}O_4$ $[M]^{+\bullet}$ 334.21386; found 334.21487.

When using the mixed fraction from 3.1.4 for further synthesis, the (2*S*,6*S*,8*S*,*E*) diastereomer **SI-3** could also be obtained and separated from the main diastereomer:

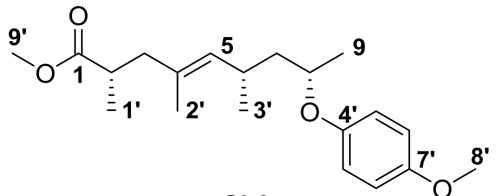

SI-3

TLC [PE/EA (30:1)]: R_f = 0.21. $[\alpha]_D^{22}$ = +35.3 (*c* = 0.57, MeOH). **^{1}H NMR** (600 MHz, $CDCl_3$): δ = 6.81-6.78 (m, 4H, C(5')*H*, C(6')*H*), 4.91 (dq, *J* = 9.7 Hz, *J* = 1.0 Hz, 1H, C(5)*H*), 4.14 (dqd, *J* = 9.2 Hz, *J* = 6 ,.0 Hz, *J* = 3.0 Hz, 1H, C(8)*H*), 3.76 (s, 3H, C(8')*H*$_3$), 3.65 (s, 3H, C(9')*H*$_3$), 2.72-2.64 (m, 1H, C(6)*H*), 2.63-2.57 (m, 1H, C(2)*H*), 2.36 (ddd, *J* = 13.5 Hz, *J* = 7.5 Hz, *J* = 0.7 Hz, 1H, C(3)*H*$_2$), 1.99 (ddd, *J* = 13.5 Hz, *J* = 7.5 Hz, *J* = 0.7 Hz, 1H, C(3)*H*$_2$), 1.73 (ddd, *J* = 13.7 Hz, *J* = 9.5 Hz, *J* = 4.0 Hz, 1H, C(7)*H*$_2$), 1.42 (d, *J* = 1.3 Hz, 3H, C(2')*H*$_3$), 1.31 (ddd, *J* = 13.7 Hz, *J* = 10.2 Hz, *J* = 3.1 Hz, 1H, C(7)*H*$_2$), 1.19 (d, *J* = 6.1 Hz, 3H, C(9)*H*$_3$), 1.10 (d, *J* = 7.0 Hz, 3H, C(1')*H*$_3$), 0.95 (d, *J* = 6.7 Hz, 3H, C(3')*H*$_3$). **^{13}C NMR** (150.9 MHz, $CDCl_3$): δ = 177.0 (1C, *C*(1)), 153.6 (1C, *C*(7')), 152.2 (1C, *C*(4')), 133.3 (1C, *C*(5)H), 131.6 (1C, *C*(4)), 117.2 + 114.5 (4C, *C*(5')H, *C*(6')H), 72.7 (1C, *C*(8)H), 55.7 (1C, *C*(8')H$_3$), 51.5 (1C, *C*(9')H$_3$), 45.0 (1C, *C*(7)H$_2$), 44.0 (1C, *C*(3)H$_2$), 38.0 (1C, *C*(2)H), 29.1 (1C, *C*(6)H), 21.6 (1C, *C*(3')H$_3$), 20.1 (1C, *C*(9)H$_3$), 16.7 (1C, *C*(1')H$_3$), 15.8 (1C, *C*(2')H$_3$).

3.1.13 Methyl (2*S*,6*R*,8*S*,*E*)-8-hydroxy-2,4,6-trimethylnon-4-enoate (7)

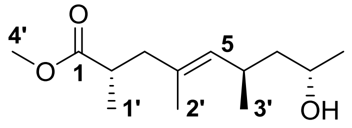

7

At room temperature, the protected polyketide **20** (0.107 g, 0.320 mmol, 1.0 equiv) was dissolved in MeCN (20 mL) and H_2O (5 mL). After cooling to 0 °C, ceric ammonium nitrate (0.386 g, 0.704 mmol, 2.2 equiv) was added in one portion. The mixture was stirred for 10 min at 0 °C. H_2O and MTBE (40 mL each) were added, the phases were separated and the aqueous phase was further extracted with MTBE (3 × 20 mL). The combined organic phases were dried over $MgSO_4$ and the solvents were removed in vacuo. After column chromatography [silica, PE/EA (3:1)], the alcohol **7** (0.059 g, 0.258 mmol, 81%) was obtained as a yellow oil.

TLC [PE/EA (3:1)]: R_f = 0.37. $[\alpha]_D^{24}$ = −13.9 (*c* = 0.23, $CHCl_3$). **^{1}H NMR** (600 MHz, $CDCl_3$): δ = 5.02-5.00 (m, 1H, C(5)*H*), 3.82-3.77 (m, 1H, C(8)*H*), 3.65 (s, 3H, C(4')*H*$_3$), 2.65-2.60 (m, 1H, C(2)*H*), 2.52-2.44 (m, 1H, C(6)*H*), 2.34 (ddd, *J* = 13.6 Hz, *J* = 7.9 Hz, *J* = 1.0 Hz, 1H, C(3)*H*$_2$), 2.04 (ddd, *J* = 13.6 Hz, *J* = 7.2 Hz, *J* = 0.9 Hz, 1H, C(3)*H*$_2$), 1.64 (br s, 1H, O*H*), 1.63 (d, *J* = 1.4 Hz, 3H, C(2')*H*$_3$), 1.46-1.37 (m, 2H, C(7)*H*$_2$), 1.15 (d, *J* = 6.2 Hz, 3H, C(9)*H*$_3$), 1.11 (d, *J* = 6.9 Hz, 3H, C(1')*H*$_3$), 0.92 (d, *J* = 6.7 Hz, 3H, C(3')*H*$_3$). **^{13}C NMR** (150.9 MHz, $CDCl_3$): δ = 176.9 (1C, *C*(1)), 133.5 (1C, *C*(5)H), 131.4 (1C, *C*(4)), 67.2 (1C, *C*(8)H), 51.5 (1C, *C*(4')H$_3$), 47.1 (1C, *C*(7)H$_2$), 44.0 (1C, *C*(3)H$_2$), 38.0 (1C, *C*(2)H), 30.3 (1C, *C*(6)H), 23.5 (1C, *C*(9)H$_3$), 21.4 (1C, *C*(3')H$_3$), 16.7 (1C, *C*(1')H$_3$), 15.9 (1C, *C*(2')H$_3$). **IR** (ATR): $\tilde{\nu}$ = 3412 cm^{-1} (w, br), 2959 (m), 2926 (m), 2872 (w), 1737 (m), 1669 (w), 1457 (m), 1373 (w), 1286 (w), 1258 (w), 1194 (w), 1167 (m), 1115 (m), 1097 (m), 1060 (w), 1032 (w), 937 (w), 862 (w), 826 (w), 803 (w), 760 (w). **UV** (CH_2Cl_2): λ_{max} (lg ε) = −. **HRESIMS**: calc. for $C_{13}H_{24}O_3Na$ [M+Na]$^+$ 251.16177; found 251.16194.

Transformation of tetraketide 7 to known compound SI-5 for data comparison

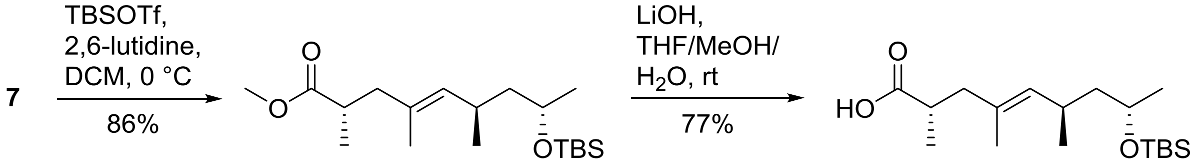

3.1.14 Methyl (2*S*,6*R*,8*S*,*E*)-8-((*tert*-butyldimethylsilyl)oxy)-2,4,6-trimethylnon-4-enoate (SI-4)

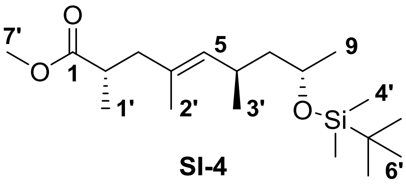

SI-4

At room temperature, the alcohol **7** (15.0 mg, 0.066 mmol, 1.0 equiv) was dissolved in DCM (10 mL) and the solution cooled to 0 °C. 2,6-lutidine (15 µL, 0.131 mmol, 2.0 equiv) and TBSOTf (18 µL, 0.099 mmol, 1.5 equiv) were added and the mixture was stirred at 0 °C for 1 h. Additional 2,6-lutidine (15 µL, 0.131 mmol, 2.0 equiv) and TBSOTf (18 µL, 0.099 mmol, 1.5 equiv) were added and stirring was continued at 0 °C for 20 min. The reaction was quenched by addition of H_2O and saturated aqueous $NaHCO_3$ (5 mL each). The phases were separated and the aqueous phase was further extracted with DCM (3 × 20 mL). The combined organic phases were dried over $MgSO_4$ and the solvent was removed in vacuo. Column chromatography [silica, PE/EA (10:1)] delivered silyl ether **SI-4** (19.4 mg, 0.057 mmol, 86%) as a colorless oil.

TLC [PE/EA (10:1)]: R_f = 0.62. $[\alpha]_D^{24}$ = −7.2 (*c* = 0.65, $CHCl_3$). **^{1}H NMR** (600 MHz, $CDCl_3$): δ = 4.95-4.93 (m, 1H, C(5)*H*), 3.77-3.72 (m, 1H, C(8)*H*), 3.65 (s, 3H, C(7')H_3), 2.64-2.58 (m, 1H, C(2)*H*), 2.46-2.38 (m, 1H, C(6)*H*), 2.34 (ddd, *J* = 13.4 Hz, *J* = 7.2 Hz, *J* = 0.9 Hz, 1H, C(3)H_2), 2.01 (ddd, *J* = 13.4 Hz, *J* = 8.0 Hz, *J* = 0.8 Hz, 1H, C(3)H_2), 1.59 (d, *J* = 1.4 Hz, 3H, C(2')H_3), 1.42 (ddd, *J* = 13.4 Hz, *J* = 7.8 Hz, *J* = 6.4 Hz, 1H, C(7)H_2), 1.29 (ddd, *J* = 13.5 Hz, *J* = 6.8 Hz, *J* = 6.8 Hz, 1H, C(7)H_2), 1.09 (d, *J* = 7.1 Hz, 3H, C(1')H_3), 1.09 (d, *J* = 5.9 Hz, 3H, C(9)H_3), 0.89 (d, *J* = 6.6 Hz, 3H, C(3')H_3), 0.88 (s, 9H, C(6')H_3), 0.04 (s, 3H, C(4')H_3), 0.04 (s, 3H, C(4')H_3). **^{13}C NMR** (150.9 MHz, $CDCl_3$): δ = 177.1 (1C, *C*(1)O), 134.0 (1C, *C*(5)H), 130.2 (1C, *C*(4)), 66.7 (1C, *C*(8)H), 51.4 (1C, *C*(7')H_3), 47.6 (1C, *C*(7)H_2), 44.2 (1C, *C*(3)H_2), 37.9 (1C, *C*(2)H), 29.0 (1C, *C*(6)H), 25.9 (3C, *C*(6')H_3), 23.7 (1C, *C*(9)H_3), 21.0 (1C, *C*(3')H_3), 18.2 (1C, *C*(5')), 16.5 (1C, *C*(1')H_3), 15.6 (1C, *C*(2')H_3), −4.4 (1C, *C*(4')H_3), −4.8 (1C, *C*(4')H_3). **IR** (ATR): $\tilde{\nu}$ = 2955 cm^{-1} (m), 2928 (m), 2856 (m), 1741 (m), 1460 (m), 1374 (m), 1361 (m), 1253 (m), 1193 (w), 1164 (m), 1132 (w), 1100 (m), 1051 (m), 1023 (m), 987 (m), 956 (w), 919 (w), 832 (m), 804 (m), 773 (m), 720 (w), 662 (w). **UV** (CH_2Cl_2): λ_{max} (lg ε) = −. **HRESIMS**: calc. for $C_{19}H_{38}O_3SiNa$ $[M+Na]^+$ 365.24824; found 365.24843.

3.1.15 (2*S*,6*R*,8*S*,*E*)-8-((*tert*-Butyldimethylsilyl)oxy)-2,4,6-trimethylnon-4-enoic acid (SI-5)

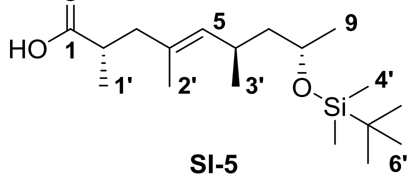

SI-5

At room temperature, the silyl ether **SI-4** (10.9 mg, 0.032 mmol, 1.0 equiv) was dissolved in THF (750 µL), MeOH (500 µL) and H_2O (250 µL). LiOH (6.1 mg, 0.255 mmol, 8.0 equiv) was added and the solution was stirred at room temperature for 2 h. The solvents were removed in vacuo and the residue was dissolved in a

mixture of saturated aqueous NH_4Cl and MTBE (5 mL each). The phases were separated and the aqueous phase was further extracted with MTBE (2 × 5 mL). The combined organic phases were dried over $MgSO_4$ and the solvent was removed in vacuo. After column chromatography [silica, PE/EA (3:1)], the hydroxynonenoic acid **SI-5** (8.1 mg, 0.025 mmol, 77%) was obtained as a colorless oil. All spectral data matched literature records.[3]

TLC [PE/EA (3:1)]: R_f = 0.70. $[\alpha]_D^{29}$ = −6.4 (*c* = 0.77, $CHCl_3$). **^{1}H NMR** (600 MHz, $CDCl_3$): δ = 11.03 (br s, 1H, COO*H*), 4.98-4.96 (m, 1H, C(5)*H*), 3.78-3.72 (m, 1H, C(8)*H*), 2.65-2.59 (m, 1H, C(2)*H*), 2.47-2.40 (m, 1H, C(6)*H*), 2.39 (dd, *J* = 13.2 Hz, *J* = 6.6 Hz, 1H, C(3)H_2), 2.03 (dd, *J* = 13.6 Hz, *J* = 8.1 Hz, 1H, C(3)H_2), 1.60 (d, *J* = 1.3 Hz, 3H, C(2')H_3), 1.43 (ddd, *J* = 13.5 Hz, *J* = 7.8 Hz, *J* = 6.4 Hz, 1H, C(7)H_2), 1.29 (ddd, *J* = 13.4 Hz, *J* = 6.8 Hz, *J* = 6.8 Hz, 1H, C(7)H_2), 1.12 (d, *J* = 6.9 Hz, 3H, C(1')H_3), 1.10 (d, *J* = 6.0 Hz, 3H, C(9)H_3), 0.89 (d, *J* = 7.3 Hz, 3H, C(3')H_3), 0.88 (s, 9H, C(6')H_3), 0.04 (s, 3H, C(4')H_3), 0.04 (s, 3H, C(4')H_3). **^{13}C NMR** (150.9 MHz, $CDCl_3$): δ = 182.1 (1C, *C*(1)OOH), 134.3 (1C, *C*(5)H), 129.9 (1C, *C*(4)), 66.7 (1C, *C*(8)H), 47.5 (1C, *C*(7)H_2), 43.8 (1C, *C*(3)H_2), 37.6 (1C, *C*(2)H), 29.1 (1C, *C*(6)H), 25.9 (3C, *C*(6')H_3), 23.7 (1C, *C*(9)H_3), 20.9 (1C, *C*(3')H_3), 18.2 (1C, *C*(5')), 16.2 (1C, *C*(1')H_3), 15.6 (1C, *C*(2')H_3), −4.4 (1C, *C*(4')H_3), −4.8 (1C, *C*(4')H_3). **IR** (ATR): $\tilde{\nu}$ = 3400-2500 cm^{-1} (w, br), 2956 (m), 2928 (m), 2857 (m), 1708 (m), 1462 (w), 1416 (w), 1376 (w), 1292 (w), 1252 (m), 1132 (w), 1102 (w), 1051 (m), 1024 (w), 987 (w), 956 (w), 834 (m), 806 (w), 774 (m), 662 (w). **UV** (CH_2Cl_2): λ_{max} (lg ε) = −. **HRESIMS**: calc. for $C_{18}H_{36}O_3SiNa$ $[M+Na]^+$ 351.23259; found 351.23282.

3.2 Synthesis of amino acids for the tripeptide section

3.2.1 General procedures for amino acid synthesis

a) Boc protection

At room temperature, the amino acid (1.0 equiv, with a free C terminus or methyl ester function) was dissolved in a mixture of dioxane and H_2O or dioxane and 1 M NaOH (as a 1:1 mixture each, 10 mL/mmol). If dioxane/H_2O was employed, the mixture was cooled to 0 °C and NEt_3 (1.5 equiv) was added. In both cases, Boc_2O (1.1-1.65 equiv) was now added and the mixture was stirred at room temperature for the specified time. The reaction was quenched by adding citric acid (10% aqueous), HCl (2 M aqueous) or $KHSO_4$ (1 M aqueous) until pH 2 was reached. The aqueous phase was extracted with EtOAc (3 × 10 mL/mmol). The combined organic phases were dried over $MgSO_4$ and the solvents were removed in vacuo. The Boc-protected amino acids were either used without further purification or purified by column chromatography.

b) *N*- and *O*-methylation with sodium hydride and iodomethane

At room temperature, the Boc-protected amino acid (1.0 equiv) was dissolved in DMF (10 mL/mmol) and NaH (3.0–10.0 equiv) was added in one portion. The suspension was stirred at room temperature for 20 min and then, iodomethane (6.0–10.0 equiv) was added. After stirring at room temperature for the specified time, the reaction was quenched by adding saturated aqueous NH_4Cl (15 mL/mmol) and the aqueous phase was extracted with EtOAc (3 × 20 mL/mmol). The combined organic phases were washed with saturated

[3] Wattanasereekul, S.; Maier, M. E. *Adv. Synth. Catal.* **2004**, *346*, 855-861.

aqueous NH_4Cl (2 × 10 mL/mmol), dried over $MgSO_4$ and the solvent was removed in vacuo. After column chromatography, the methylated amino acids were obtained.

Synthesis of N-Boc-protected iodotyrosines and oxazolidinone pathway

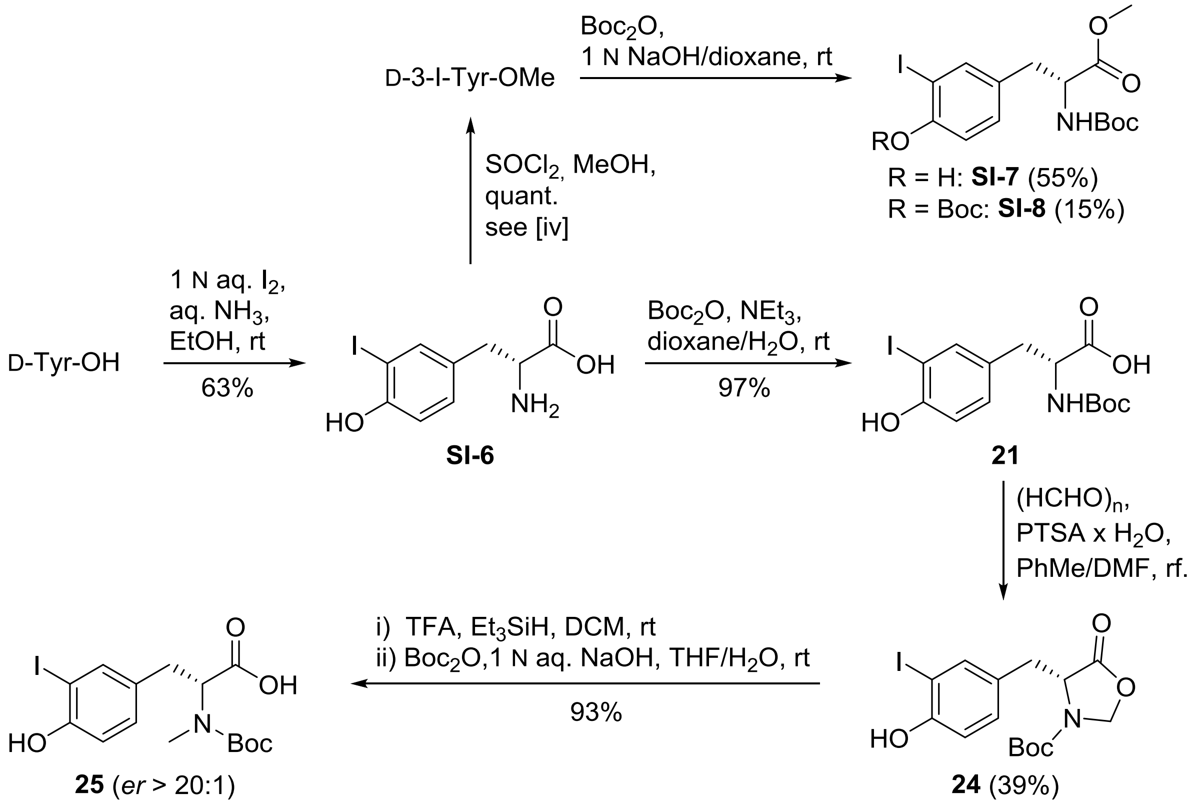

3.2.2 (*R*)-2-Amino-3-(4-hydroxy-3-iodophenyl)propanoic acid (SI-6)

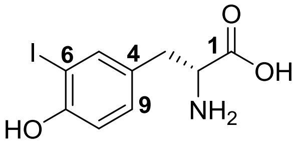

SI-6

At room temperature, D-tyrosine (2.718 g, 15.000 mmol, 1.0 equiv) was dissolved in an ammonium hydroxide solution (25% aqueous, 350 mL). An aqueous solution of iodine (30.00 mL, 15.000 mmol, 1 N, 1.0 equiv) was added over a peroid of 90 min using a syringe pump (risk of explosion!), while any purple solid that crystallized was immediately washed into the solution with EtOH (50 mL in total). After the end of addition, stirring was continued for 1 h. Then, the solution was concentrated to a total volume of 75 mL under reduced pressure. The pH of the mixture was carefully brought to 4.5 by adding HCl (2 M aqueous) and the suspension was left in a fridge (4 °C) over night. The crystallized product was collected by suction filtration. Recrystallization from H_2O (75 mL) delivered D-3-iodotyrosine (**SI-6**, 2.883 g, 9.383 mmol, 63%) as a colorless solid.

mp: 203-204 °C (decomp.). $[\alpha]_D^{21}$ = +9.1 (*c* = 1.10, 2 M HCl). **^{1}H NMR** (400 MHz, MeOD): δ = 7.65 (d, *J* = 1.3 Hz, 1H, C(5)*H*), 7.14 (dd, *J* = 8.0 Hz, *J* = 1.3 Hz, 1H, C(9)*H*), 6.81 (d, *J* = 8.2 Hz, 1H, C(8)*H*), 3.70 (dd, *J* = 8.1 Hz, *J* = 4.0 Hz, 1H, C(2)*H*), 3.17 (dd, *J* = 14.8 Hz, *J* = 3.7 Hz, 1H, C(3)H_2), 2.90 (dd, *J* = 14.6 Hz, *J* = 8.4 Hz, 1H, C(3)H_2). **^{13}C NMR** (100.6 MHz, MeOD): δ = 173.6 (1C, *C*(1)), 157.5 (1C, *C*(7)), 141.1 (1C, *C*(5)H), 131.6 (1C, *C*(9)H), 129.9 (1C, *C*(4)), 116.1 (1C, *C*(8)H), 85.1 (1C, *C*(6)), 57.6 (1C, *C*(2)H), 36.8 (1C, *C*(3)H_2). **IR** (ATR): $\tilde{\nu}$ = 3507 cm^{-1} (w, br), 3236-2465 (m, br), 1585 (m), 1522 (m), 1504 (m), 1434 (m), 1412 (m), 1353 (m), 1327 (m), 1298 (m), 1277 (m), 1226 (m), 1143 (w), 1116 (m), 1078 (w), 1033 (m),

980 (w), 902 (w), 877 (m), 831 (m), 798 (w), 744 (m), 714 (m), 663 (w), 633 (m), 606 (m), 538 (s). **UV** (MeOH): λ_{max} (lg ε) = 285 nm (3.45), 227 (4.01), 206 (4.46). **MS** (EI, 70 eV): *m/z* (%) = 307 (6) $[M]^{+\bullet}$, 261 (11), 234 (19), 233 (100), 135 (7), 128 (11), 107 (30), 106 (14), 44 (19).

3.2.3 (*R*)-2-((*tert*-Butoxycarbonyl)amino)-3-(4-hydroxy-3-iodophenyl)propanoic acid (21)

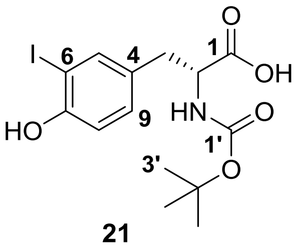

21

The Boc-amino acid **21** was synthesized according to the general procedure for Boc protection (3.2.1) from the iodotyrosine **SI-6** (1.059 g, 3.449 mmol, 1.0 equiv) with dioxane/H_2O, NEt_3 (1.5 equiv) and Boc_2O (1.1 equiv, 24 h). After column chromatography [silica, EA (100%)], the Boc-amino acid **21** (1.363 g, 3.347 mmol, 97%) was obtained as a pale yellow foam.

Melting range: 52-125 °C. **TLC** [EA (100%)]: R_f = 0.47. $[\alpha]_D^{21}$ = −18.1 (*c* = 3.16, MeOH). **^{1}H NMR** (400 MHz, MeOD): δ = 7.54 (d, *J* = 1.7 Hz, 1H, C(5)*H*), 7.04 (dd, *J* = 8.3 Hz, *J* = 2.2 Hz, 1H, C(9)*H*), 6.75 (d, *J* = 8.2 Hz, 1H, C(8)*H*), 4.26 (dd, *J* = 8.8 Hz, *J* = 5.0 Hz, 1H, C(2)*H*), 3.04 (dd, *J* = 13.9 Hz, *J* = 5.0 Hz, 1H, C(3)H_2), 2.78 (dd, *J* = 13.9 Hz, *J* = 9.0 Hz, 1H, C(3)H_2), 1.39 (s, 9H, C(3')H_3). **^{13}C NMR** (100.6 MHz, MeOD): δ = 175.3 (1C, *C*(1)), 157.8 (1C, *C*(1')), 156.8 (1C, *C*(7)), 141.0 (1C, *C*(5)H), 131.5 (1C, *C*(4)), 131.4 (1C, *C*(9)H), 115.6 (1C, *C*(8)H), 84.4 (1C, *C*(6)), 80.6 (1C, *C*(2')), 56.4 (1C, *C*(2)H), 37.4 (1C, *C*(3)H_2), 28.7 (3C, *C*(3')H_3). **IR** (ATR): $\tilde{\nu}$ = 3650-2400 cm^{-1} (w, br), 3332 (w, br), 2978 (w), 2932 (w), 1683 (m), 1504 (w), 1412 (w), 1395 (w), 1368 (w), 1250 (w), 1221 (w), 1158 (m), 1054 (w), 821 (w), 790 (w), 662 (w), 563 (w). **UV** (CH_2Cl_2): λ_{max} (lg ε) = 289 nm (3.30), 282 (3.33). **HRESIMS**: calc. for $C_{14}H_{18}INO_5Na$ $[M+Na]^+$ 430.01219, found 430.01229.

3.2.4 Methyl (*R*)-2-((*tert*-butoxycarbonyl)amino)-3-(4-hydroxy-3-iodophenyl)propanoate (SI-7)

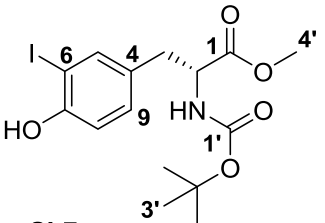

SI-7

The Boc-amino acid **SI-7** was synthesized according to the general procedure for Boc protection (3.2.1) from D-3-iodotyrosine methyl ester[4] (2.600 g, 7.271 mmol, 1.0 equiv) with dioxane/1 M NaOH and Boc_2O (1.48 equiv, 2 h). After column chromatography [silica, PE/EA (3:1)], the Boc-amino acid **SI-7** (1.670 g, 3.965 mmol, 55%) was obtained as a colorless solid.

mp: 109 °C. **TLC** [PE/EA (3:1)]: R_f = 0.20. $[\alpha]_D^{22}$ = −9.8 (*c* = 1.85, MeOH). **^{1}H NMR** (400 MHz, $CDCl_3$): δ = 7.42 (d, *J* = 1.7 Hz, 1H, C(5)*H*), 6.99 (dd, *J* = 8.3 Hz, *J* = 2.1 Hz, 1H, C(9)*H*), 6.88 (d, *J* = 8.3 Hz, 1H, C(8)*H*), 5.52 (br s, 1H, O*H*), 5.01 (d, *J* = 7.9 Hz, 1H, N*H*), 4.52 (dt, *J* = 7.9 Hz, *J* = 6.0 Hz, 1H, C(2)*H*), 3.73 (s, 3H, C(4')H_3), 3.04 (dd, *J* = 14.0 Hz, *J* = 5.7 Hz, 1H, C(3)H_2), 2.93 (dd, *J* = 14.0 Hz, *J* = 6.0 Hz, 1H, C(3)H_2), 1.43 (s, 9H, C(3')H_3). **^{13}C NMR** (100.6 MHz, $CDCl_3$): δ = 172.1 (1C, *C*(1)), 155.0 (1C, *C*(1')), 154.0 (1C, *C*(7)), 138.9 (1C, *C*(5)H), 131.0 (1C, *C*(9)H), 130.1 (1C, *C*(4)), 115.0 (1C, *C*(8)H), 85.5 (1C, *C*(6)), 80.1 (1C, *C*(2')), 54.5 (1C, *C*(2)H), 52.3 (1C, *C*(4')H_3), 37.0 (1C, *C*(3)H_2), 28.3 (3C, *C*(3')H_3). **IR** (ATR): $\tilde{\nu}$ = 3355 cm^{-1} (m, br), 2978 (m), 1681 (s), 1603 (w), 1573 (w), 1503 (m), 1439 (m), 1414 (m),

[4] White, J. D.; Amedio, Jr., J. C. *J. Org. Chem.* **1989**, *54*, 738-743.

1392 (m), 1365 (m), 1289 (m), 1250 (m), 1214 (m), 1158 (s), 1057 (m), 1016 (m), 864 (m), 822 (m), 805 (m), 753 (m), 664 (m). **UV** (CH_2Cl_2): λ_{max} (lg ε) = 289 nm (3.38), 282 (3.39). **HRESIMS**: calc. for $C_{15}H_{20}INO_5Na$ $[M+Na]^+$ 444.02784, found 444.02818.

As a byproduct, the *N*,*O*-Di-Boc derivate **SI-8** (0.570 g, 1.093 mmol, 15%) was isolated as a colorless, solidified oil.

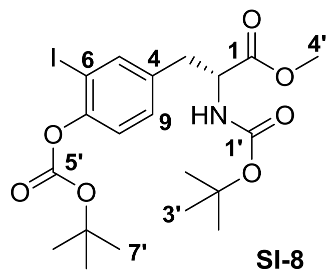

SI-8

TLC [PE/EA (3:1)]: R_f = 0.43. $[\alpha]_D^{22}$ = −4.3 (*c* = 2.19, MeOH). **^{1}H NMR** (400 MHz, $CDCl_3$): δ = 7.59-7.58 (m, 1H, C(5)*H*), 7.13 (dd, *J* = 8.3 Hz, *J* = 1.7 Hz, 1H, C(9)*H*), 7.09 (d, *J* = 8.2 Hz, 1H, C(8)*H*), 5.01 (d, *J* = 7.5 Hz, 1H, N*H*), 4.58-4.53 (m, 1H, C(2)*H*), 3.72 (s, 3H, C(4')*H*$_3$), 3.10 (dd, *J* = 13.9 Hz, *J* = 5.7 Hz, 1H, C(3)*H*$_2$), 3.00 (dd, *J* = 13.9 Hz, *J* = 6.2 Hz, 1H, C(3)*H*$_2$), 1.57 (s, 9H, C(7')*H*$_3$), 1.43 (br s, 9H, C(3')*H*$_3$). **^{13}C NMR** (100.6 MHz, $CDCl_3$): δ = 171.9 (1C, *C*(1)), 155.0 (1C, *C*(1')), 150.9 (1C, *C*(5')), 150.4 (1C, *C*(7)), 140.2 (1C, *C*(5)H), 136.0 (1C, *C*(4)), 130.4 (1C, *C*(9)H), 122.6 (1C, *C*(8)H), 90.5 (1C, *C*(6)), 84.2 (1C, *C*(6')), 80.2 (1C, *C*(2')), 54.2 (1C, *C*(2)H), 52.4 (1C, *C*(4')H$_3$), 37.1 (1C, *C*(3)H$_2$), 28.3 (3C, *C*(3')H$_3$), 27.7 (3C, *C*(7')H$_3$). **IR** (ATR): $\tilde{\nu}$ = 3371 cm^{-1} (w, br), 2979 (m), 2933 (w), 1759 (m), 1710 (m), 1502 (m), 1485 (m), 1455 (m), 1393 (w), 1367 (m), 1277 (m), 1253 (m), 1226 (m), 1144 (s), 1052 (m), 1016 (m), 897 (m), 859 (m), 775 (m), 756 (m), 663 (w). **UV** (CH_2Cl_2): λ_{max} (lg ε) = 295 nm (2.72), 278 (3.00), 271 (3.00). **HRESIMS**: calc. for $C_{20}H_{28}INO_7Na$ $[M+Na]^+$ 544.08027, found 544.07999.

3.2.5 *tert*-Butyl (*R*)-4-(4-hydroxy-3-iodobenzyl)-5-oxooxazolidine-3-carboxylate (24)

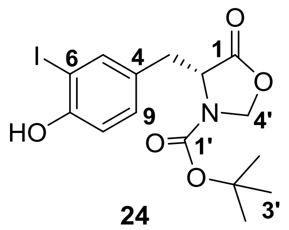

24

At room temperature, the Boc-protected amino acid **21** (1.301 g, 3.195 mmol, 1.0 equiv) was dissolved in a mixture of DMF (1 mL) and toluene (60 mL). Paraformaldehyde (0.480 g, 15.975 mmol, 5.0 equiv) and *para*-toluenesulfonic acid hydrate (0.320 mmol, 0.1 equiv) were added and the mixture was heated under reflux for 2 h, using a Dean-Stark apparatus. After cooling, most of the solvent was removed in vacuo and the oily residue was taken up in EtOAc (60 mL). The organic solution was washed with saturated aqueous $NaHCO_3$ (3 × 20 mL), dried over $MgSO_4$ and the solvent was removed in vacuo. After column chromatography [silica, PE/EA (2:1)], the oxazolidinone **24** (0.517 g, 1.233 mmol, 39%) was obtained as a colorless foam. In the ^{1}H and ^{13}C NMR spectra, strong peak broadening occurred due to the existence of rotamers (ratio in $CDCl_3$ could not be determined because of poor signal separation).

mp: 58 °C. **TLC** [PE/EA (1:1)]: R_f = 0.67. $[\alpha]_D^{21}$ = −83.7 (*c* = 4.25, $CHCl_3$). **^{1}H NMR** (400 MHz, $CDCl_3$, mixture of rotamers): δ = 7.46 (d, *J* = 2.1 Hz, 1H, C(5)*H*), 7.04 (dd, *J* = 8.3 Hz, *J* = 2.0 Hz, 1H, C(9)*H*), 6.91 (d, *J* = 8.3 Hz, 1H, C(8)*H*), 5.45 (s, 1H, O*H*), 5.38-5.16 (m, 1H, C(4')*H*$_2$), 4.51-4.37 (m, 2H, C(2)*H*, C(4')*H*$_2$), 3.39-3.16 (m, 1H, C(3)*H*$_2$), 3.08-3.03 (m, 1H, C(3)*H*$_2$), 1.52 (s, 9H, C(3')*H*$_3$). **^{13}C NMR** (100.6 MHz, $CDCl_3$, mixture of rotamers): δ = 172.1 (1C, *C*(1)), 154.4 (1C, *C*(7)), 151.4 (1C, *C*(1')),139.3 (1C, *C*(5)H), 131.4 (1C, *C*(9)H), 129.0 (1C, *C*(4)), 115.2 (1C, *C*(8)H), 85.7 (1C, *C*(6)), 82.2 (1C, *C*(2')), 78.1 (1C, *C*(4')H$_2$), 56.2 (1C, *C*(2)H), 56.1 (1C, *C*(2)H), 34.9 (1C, *C*(3)H$_2$), 33.8 (1C, *C*(3)H$_2$), 28.3 (3C, *C*(3')H$_3$). **IR** (ATR):

$\tilde{\nu}$ = 3352 cm^{-1} (w, br), 2977 (w), 2929 (w), 1796 (m), 1680 (m), 1603 (w), 1503 (m), 1396 (m), 1368 (m), 1316 (w), 1291 (m), 1256 (m), 1212 (m), 1132 (m), 1042 (m), 996 (m), 968 (m), 916 (w), 852 (m), 822 (m), 765 (m), 735 (m), 708 (w), 663 (m), 608 (m), 576 (m). **UV** (CH_2Cl_2): λ_{max} (lg ε) = 288 nm (3.32), 281 (3.35). **HRESIMS**: calc. for $C_{16}H_{22}INO_6Na$ $[M+MeOH+Na]^+$ 474.03840, found 474.03896.

3.2.6 (*R*)-2-((*tert*-Butoxycarbonyl)(methyl)amino)-3-(4-hydroxy-3-iodophenyl)-propanoic acid (25)

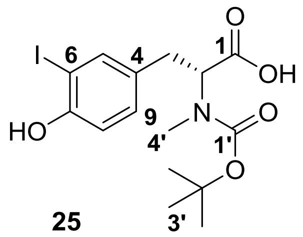

25

At 0 °C, the oxazolidinone **24** (0.555 g, 1.324 mmol, 1.0 equiv) was dissolved in TFA and DCM (1:1, 15 mL each). After the addition of triethylsilane (846 µL, 5.296 mmol, 4.0 equiv), the solution was brought to room temperature and stirred for 20 h. The solvents were removed in vacuo and the residue was dissolved in H_2O (15 mL). The pH of the solution was adjusted to 7 by adding solid $NaHCO_3$. The mixture was recooled to 0 °C and NaOH (2.65 mL, 2.648 mmol, 1 M aqueous, 2.0 equiv) as well as a solution of Boc_2O (0.433 g, 1.986 mmol, 1.5 equiv) in THF (15 mL) were added. The mixture was stirred at room temperature for 4 h and afterwards, THF was removed in vacuo. The aqueous phase was extracted with hexane (2 × 30 mL) and the organic phases were discarded. The pH of the aqueous phase was adjusted to 2 by adding HCl (6 M aqueous) and it was then extracted with DCM (3 × 50 mL). The combined organic phases were washed with saturated aqueous NaCl (30 mL), dried over $MgSO_4$ and the solvents were removed in vacuo. After column chromatography [silica, EA (100%)], the *N*-methylated amino acid **25** (0.521 g, 1.237 mmol, 93%) was obtained as a slightly tan foam. The compound produced a double NMR signal set due to the occurence of rotamers (ratio 1.3:1 in $CDCl_3$).

mp: 129-135 °C. **TLC** [EA (100%)]: R_f = 0.34. $[\alpha]_D^{21}$ = +52.2 (c = 1.57, $CHCl_3$). **^{1}H NMR** (600 MHz, $CDCl_3$, major rotamer): δ = 7.50-7.48 (m, 1H, C(5)*H*), 7.09 (d, *J* = 8.3 Hz, 1H, C(9)*H*), 6.90 (d, *J* = 8.3 Hz, 1H, C(8)*H*), 5.54 (br s, 1H, O*H*), 4.67 (dd, *J* = 10.6 Hz, *J* = 5.1 Hz, 1H, C(2)*H*), 3.21 (dd, *J* = 14.5 Hz, *J* = 4.9 Hz, 1H, C(3)H_2), 3.05 (dd, *J* = 14.3 Hz, *J* = 11.1 Hz, 1H, C(3)H_2), 2.69 (s, 3H, C(4')H_3), 1.44 (s, 9H, C(3')H_3). **^{13}C NMR** (150.9 MHz, $CDCl_3$, major rotamer): δ = 174.9 (1C, *C*(1)), 156.6 (1C, *C*(1')), 153.9 (1C, *C*(7)), 138.5 (1C, *C*(5)H), 131.2 (1C, *C*(4)), 130.8 (1C, *C*(9)H), 115.1 (1C, *C*(8)H), 85.5 (1C, *C*(6)), 81.3 (1C, *C*(2')), 61.3 (1C, *C*(2)H), 33.6 (1C, *C*(4')H_3), 33.4 (1C, *C*(3)H_2), 28.4 (3C, *C*(3')H_3). **^{1}H NMR** (600 MHz, $CDCl_3$, minor rotamer): δ = 7.51-7.49 (m, 1H, C(5)*H*), 7.03 (d, *J* = 8.0 Hz, 1H, C(9)*H*), 6.90 (d, *J* = 8.3 Hz, 1H, C(8)*H*), 5.54 (br s, 1H, O*H*), 4.60 (dd, *J* = 10.6 Hz, *J* = 5.1 Hz, 1H, C(2)*H*), 3.21 (dd, *J* = 14.5 Hz, *J* = 4.9 Hz, 1H, C(3)H_2), 2.92 (dd, *J* = 14.1 Hz, *J* = 11.3 Hz, 1H, C(3)H_2), 2.76 (s, 3H, C(4')H_3), 1.37 (s, 9H, C(3')H_3). **^{13}C NMR** (150.9 MHz, $CDCl_3$, minor rotamer): δ = 175.5 (1C, *C*(1)), 155.1 (1C, *C*(1')), 154.0 (1C, *C*(7)), 138.5 (1C, *C*(5)H), 131.5 (1C, *C*(4)), 130.9 (1C, *C*(9)H), 115.1 (1C, *C*(8)H), 85.6 (1C, *C*(6)), 81.1 (1C, *C*(2')), 61.2 (1C, *C*(2)H), 33.9 (1C, *C*(3)H_2), 32.5 (1C, *C*(4')H_3), 28.3 (3C, *C*(3')H_3). **IR** (ATR): $\tilde{\nu}$ = 3600-2400 cm^{-1} (w, br), 3219 (w, br), 2976 (m), 2933 (w), 1714 (m), 1661 (m), 1504 (w), 1486 (m), 1446 (w), 1393 (m), 1368 (m), 1331 (m), 1286 (w), 1252 (w), 1212 (m), 1145 (m), 1073 (w), 1035 (w), 963 (w), 941 (w), 859 (w), 814 (m), 769 (m), 661 (m), 584 (w), 541 (w).

UV (CH_2Cl_2): λ_{max} (lg ε) = 289 nm (3.14), 282 (3.16). **HRESIMS**: calc. for $C_{15}H_{20}INO_5Na$ $[M+Na]^+$ 444.02784, found 444.02811.

Methylation of iodotyrosine

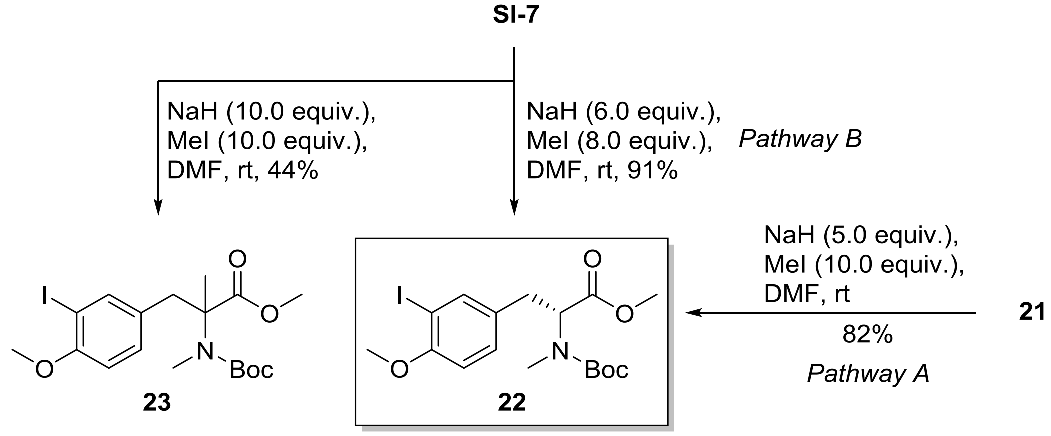

3.2.7 Methyl (*R*)-2-((*tert*-butoxycarbonyl)(methyl)amino)-3-(3-iodo-4-methoxyphenyl)propanoate (22)

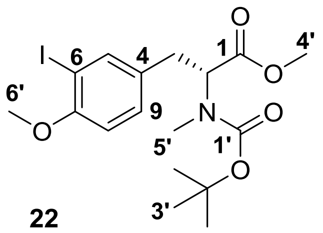

The fully protected amino acid **22** could be prepared following two different pathways.

Pathway A: Direct methylation of *N*-Boc-D-3-iodotyrosine (**21**)
The amino acid **22** was synthesized according to the general procedure for methylation reactions detailed in 3.2.1, employing *N*-Boc-D-3-iodotyrosine (**21**, 1.085 g, 2.665 mmol, 1.0 equiv), sodium hydride (5.0 equiv) and iodomethane (10.0 equiv, 24 h). After column chromatography [silica, PE/EA (3:1)], the protected amino acid **22** (0.980 g, 2.181 mmol, 82%) was obtained as a pale yellow oil. The compound was obtained as an enantiomeric mixture (er 4:1).

Pathway B: Methylation of the Boc-protected methyl ester **SI-7**
The general procedure for methylation reactions as detailed in 3.2.1 coud also be applied to the Boc-protected methyl ester **SI-7** (0.100 g, 0.237 mmol, 1.0 equiv). By employing sodium hydride (6.0 equiv) and iodomethane (8.0 equiv) with a reaction time of 20 h and a column chromatographic separation [silica, PE/EA (3:1)], the fully protected amino acid **22** (0.097 g, 0.216 mmol, 91%) was obtained. The er was worse than for pathway A.

In both cases, the compound **22** produced a double NMR signal set due to the occurence of rotamers (ratio 1.2:1 in $CDCl_3$).

TLC [PE/MTBE (2:1)]: R_f = 0.28. $[\alpha]_D^{24}$ = +49.7 (*c* = 2.87, MeOH). **^{1}H NMR** (600 MHz, $CDCl_3$, major rotamer): δ = 7.62 (d, *J* = 2.1 Hz, 1H, C(5)*H*), 7.08 (ap. d, *J* = 7.7 Hz, 1H, C(9)*H*), 6.74 (d, *J* = 8.4 Hz, 1H, C(8)*H*), 4.53 (dd, *J* = 10.6 Hz, *J* = 4.5 Hz, 1H, C(2)*H*), 3.85 (s, 3H, C(6')H_3), 3.75 (s, 3H, C(4')H_3), 3.19 (dd, *J* = 11.5 Hz, *J* = 4.8 Hz, 1H, C(3)H_2), 2.95-2.88 (m, 1H, C(3)H_2), 2.73 (s, 3H, C(5')H_3), 1.36 (s, 9H, C(3')H_3). **^{13}C NMR** (150.9 MHz, $CDCl_3$, major rotamer): δ = 171.4 (1C, *C*(1)), 157.0 (1C, *C*(7)), 154.9 (1C, *C*(1')), 139.7 (1C, *C*(5)H), 131.8 (1C, *C*(4)), 130.2 (1C, *C*(9)H), 110.8 (1C, *C*(8)H), 86.0 (1C, *C*(6)), 80.4 (1C, *C*(2')), 61.3 (1C,

C(2)H), 56.4 (1C, C(6')H_3), 52.2 (1C, C(4')H_3), 34.1 (1C, C(3)H_2), 32.4 (1C, C(5')H_3), 28.2 (3C, C(3')H_3). **^{1}H NMR** (600 MHz, $CDCl_3$, minor rotamer): δ = 7.59 (d, J = 2.0 Hz, 1H, C(5)H), 7.17 (ap. d, J = 8.0 Hz, 1H, C(9)H), 6.74 (d, J = 8.4 Hz, 1H, C(8)H), 4.82 (dd, J = 10.5 Hz, J = 5.4 Hz, 1H, C(2)H), 3.85 (s, 3H, C(6')H_3), 3.73 (s, 3H, C(4')H_3), 3.21 (dd, J = 12.4 Hz, J = 5.4 Hz, 1H, C(3)H_2), 2.95-2.88 (m, 1H, C(3)H_2), 2.71 (s, 3H, C(5')H_3), 1.40 (s, 9H, C(3')H_3). **^{13}C NMR** (150.9 MHz, $CDCl_3$, minor rotamer): δ = 171.7 (1C, C(1)), 156.9 (1C, C(7)), 155.8 (1C, C(1')), 139.8 (1C, C(5)H), 131.6 (1C, C(4)), 130.0 (1C, C(9)H), 110.8 (1C, C(8)H), 85.7 (1C, C(6)), 80.2 (1C, C(2')), 59.6 (1C, C(2)H), 56.4 (1C, C(6')H_3), 52.2 (1C, C(4')H_3), 33.6 (1C, C(3)H_2), 32.1 (1C, C(5')H_3), 28.3 (3C, C(3')H_3). **IR** (ATR): $\tilde{\nu}$ = 2928 cm^{-1} (m), 2852 (w), 1741 (m), 1691 (m), 1599 (w), 1490 (m), 1438 (m), 1392 (m), 1366 (m), 1325 (m), 1280 (m), 1252 (m), 1223 (m), 1144 (m), 1047 (m), 1015 (m), 959 (w), 864 (m), 808 (m), 791 (m), 771 (m), 663 (m), 557 (m). **UV** (CH_2Cl_2): λ_{max} (lg ε) = 291 nm (3.42), 283 (3.43), 231 (3.99). **HRESIMS**: calc. for $C_{17}H_{24}INO_5Na$ $[M+Na]^+$ 472.05914, found 472.05989.

If a large excess of reagents was used in pathway B (10.0 equiv of sodium hydride and iodomethane), reaction with the amino acid **SI-7** (0.200 g, 0.491 mmol, 1.0 equiv) led to the isolation of the α-methylated amino acid **23** (0.100 g, 0.216 mmol, 44%) after 18 h of reaction time as the single product. The compound was a colorless oil and produced a double NMR signal set due to the occurence of rotamers (ratio 1.4:1 in $CDCl_3$).

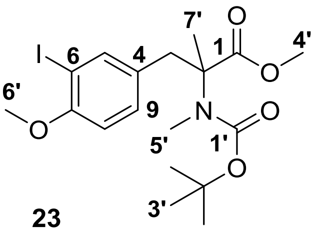

23

TLC [PE/MTBE (2:1)]: R_f = 0.33. $[\alpha]_D^{22}$ = −4.0 (c = 1.21, MeOH). **^{1}H NMR** (600 MHz, $CDCl_3$, major rotamer): δ = 7.55-7.50 (m, 1H, C(5)H), 7.08-6.99 (m, 1H, C(9)H), 6.74 (d, J = 8.4 Hz, 1H, C(8)H), 3.86 (s, 3H, C(6')H_3), 3.72 (s, 3H, C(4')H_3), 3.65-3.43 (m, 1H, C(3)H_2), 2.83 (d, J = 11.8 Hz, 1H, C(3)H_2), 2.54-2.39 (m, 3H, C(5')H_3), 1.50 (br s, 9H, C(3')H_3), 1.36 (s, 3H, C(7')H_3). **^{13}C NMR** (150.9 MHz, $CDCl_3$, major rotamer): δ = 175.3 (1C, C(1)), 157.0 (1C, C(7)), 155.3 (1C, C(1')), 141.4 (1C, C(5)H), 131.6 (1C, C(9)H), 131.2 (1C, C(4)), 110.5 (1C, C(8)H), 85.5 (1C, C(6)), 80.2 (1C, C(2')), 63.0 (1C, C(2)), 56.3 (1C, C(6')H_3), 52.2 (1C, C(4')H_3), 39.1 (1C, C(3)H_2), 31.0 (1C, C(5')H_3), 28.4 (3C, C(3')H_3), 22.0 (1C, C(7')H_3). **^{1}H NMR** (600 MHz, $CDCl_3$, minor rotamer): δ = 7.55-7.50 (m, 1H, C(5)H), 7.08-6.99 (m, 1H, C(9)H), 6.74 (d, J = 8.4 Hz, 1H, C(8)H), 3.86 (s, 3H, C(6')H_3), 3.72 (s, 3H, C(4')H_3), 3.65-3.43 (m, 1H, C(3)H_2), 2.83 (d, J = 11.8 Hz, 1H, C(3)H_2), 2.54-2.39 (m, 3H, C(5')H_3), 1.50 (br s, 9H, C(3')H_3), 1.36 (s, 3H, C(7')H_3). **^{13}C NMR** (150.9 MHz, $CDCl_3$, minor rotamer): δ = 175.3 (1C, C(1)), 157.0 (1C, C(7)), 154.9 (1C, C(1')), 141.4 (1C, C(5)H), 131.6 (1C, C(9)H), 130.8 (1C, C(4)), 110.5 (1C, C(8)H), 85.5 (1C, C(6)), 81.1 (1C, C(2')), 63.0 (1C, C(2)), 56.3 (1C, C(6')H_3), 52.2 (1C, C(4')H_3), 40.4 (1C, C(3)H_2), 31.0 (1C, C(5')H_3), 28.4 (3C, C(3')H_3), 22.7 (1C, C(7')H_3). **IR** (ATR): $\tilde{\nu}$ = 2976 cm^{-1} (m), 2948 (m), 2840 (w), 1740 (m), 1685 (s), 1598 (w), 1490 (m), 1459 (m), 1439 (m), 1366 (s), 1319 (m), 1278 (m), 1250 (s), 1142 (s), 1101 (m), 1071 (m), 1047 (m), 1016 (m), 990 (m), 928 (w), 867 (m), 815 (m), 772 (m), 757 (m), 721 (m), 663 (m), 629 (w), 589 (m), 561 (m), 541 (m). **UV** (CH_2Cl_2): λ_{max} (lg ε) = 290 nm (3.42), 283 (3.43), 232 (4.03). **HRESIMS**: calc. for $C_{18}H_{26}INO_5Na$ $[M+Na]^+$ 486.07479, found 486.07443.

Side-chain protected threonines for tripeptide synthesis

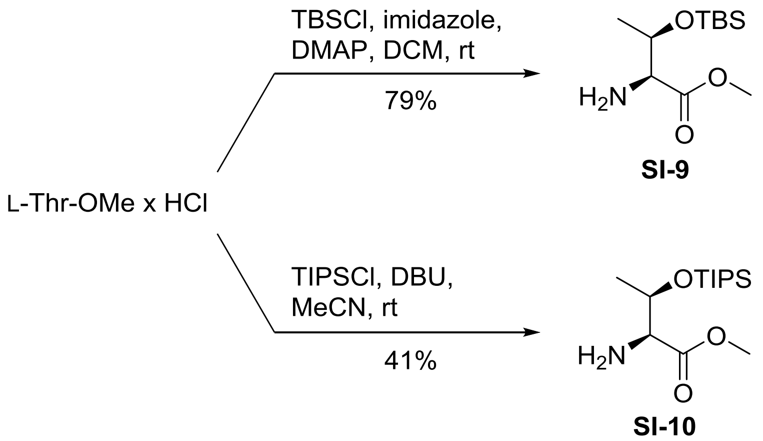

3.2.8 Methyl *O*-(*tert*-butyldimethylsilyl)-L-threoninate (SI-9)

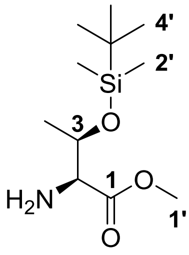

SI-9

At room temperature, L-Thr-OMe · HCl (1.458 g, 8.596 mmol, 1.0 equiv) was dissolved in DCM (50 mL). Imidazole (1.170 g, 17.192 mmol, 2.0 equiv), TBSCl (1.425 g, 9.456 mmol, 1.1 equiv) and 4-dimethylaminopyridine (0.011 g, 0.086 mmol, 1 mol %) were added and the mixture was stirred at room temperature for 19 h. The precipitated solid was removed by filtration through a porous frit (porosity 4) and the filter cake was further washed with DCM (3 × 20 mL). The organic solution was washed with saturated aqueous $NaHCO_3$ (5% aqueous) and H_2O (50 mL each), dried over $MgSO_4$ and the solvent was removed in vacuo. (OTBS)-L-Thr-OMe (**SI-9**, 1.674 g, 6.766 mmol, 79%) was obtained as a slightly yellow oil without further purification.

TLC [EA (100%)]: R_f = 0.36. $[\alpha]_D^{21}$ = −20.6 (*c* = 2.08, $CHCl_3$). **^{1}H NMR** (400 MHz, $CDCl_3$): δ = 4.29 (qd, *J* = 6.3 Hz, *J* = 2.7 Hz, 1H, C(3)*H*), 3.70 (s, 3H, C(1')H_3), 3.27 (d, *J* = 2.7 Hz, 1H, C(2)*H*), 1.56 (br s, 2H, NH_2), 1.24 (d, *J* = 6.3 Hz, 3H, C(4)H_3), 0.84 (s, 9H, C(4')H_3), 0.04 (s, 3H, C(2')H_3), −0.02 (s, 3H, C(2')H_3). **^{13}C NMR** (100.6 MHz, $CDCl_3$): δ = 174.9 (1C, *C*(1)), 69.5 (1C, *C*(3)H), 60.8 (1C, *C*(2)H), 51.9 (1C, *C*(1')H_3), 25.6 (3C, *C*(4')H_3), 20.9 (1C, *C*(4)H_3), 17.9 (1C, *C*(3')), −4.4 (1C, *C*(2')), −5.3 (1C, *C*(2')). **IR** (ATR): $\tilde{\nu}$ = 2954 cm^{-1} (m), 2931 (m), 2890 (w), 2857 (m), 1745 (m), 1469 (m), 1437 (w), 1375 (w), 1362 (w), 1253 (m), 1158 (m), 1075 (m), 1040 (m), 1003 (m), 967 (m), 886 (m), 834 (s), 807 (m), 772 (s), 738 (m), 667 (m), 560 (w), 544 (w). **UV** (MeOH): λ_{max} (lg ε) = −. **HRESIMS**: calc. for $C_{11}H_{25}NO_3SiNa$ $[M+Na]^+$ 270.14959, found 270.14966.

3.2.9 Methyl *O*-(triisopropylsilyl)-L-threoninate (SI-10)

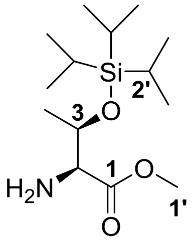

SI-10

At room temperature, L-Thr-OMe · HCl (1.194 g, 7.040 mmol, 1.0 equiv) was dissolved in MeCN (20 mL). After cooling to 0 °C, 1,8-diazabicyclo[5.4.0]undec-7-ene (4.74 mL, 31.679 mmol, 4.5 equiv) and TIPSCl (1.81 mL, 8.448 mmol, 1.2 equiv) were added and the mixture was stirred at room temperature for 20 h. The reaction was quenched by adding saturated aqueous NH_4Cl (30 mL) and the aqueous phase was extracted with DCM (3 × 100 mL). The combined organic phases were washed with

saturated aqueous NaCl (50 mL), dried over $MgSO_4$ and the solvents were removed in vacuo. After column chromatography [silica, EA (100%)], (OTIPS)-L-Thr-OMe (**SI-10**, 0.840 g, 2.902 mmol, 41%) was obtained as a pale yellow oil.

TLC [EA (100%)]: R_f = 0.52. $[\alpha]_D^{27}$ = +0.5 (*c* = 0.84, MeOH). **^{1}H NMR** (400 MHz, $CDCl_3$): δ = 4.49 (qd, *J* = 6.3 Hz, *J* = 2.7 Hz, 1H, C(3)*H*), 3.72 (s, 3H, C(1')H_3), 3.31 (d, *J* = 2.7 Hz, 1H, C(2)*H*), 1.63 (br s, 2H, NH_2), 1.32 (d, *J* = 6.3 Hz, 3H, C(4)H_3), 1.04 (s, 21H, C(2')*H*, C(3')H_3). **^{13}C NMR** (100.6 MHz, $CDCl_3$): δ = 175.0 (1C, *C*(1)), 69.9 (1C, *C*(3)H), 60.9 (1C, *C*(2)H), 51.8 (1C, *C*(1')H_3), 21.1 (1C, *C*(4)H_3), 18.0 (3C, *C*(3')H_3), 18.0 (3C, *C*(3')H_3), 12.6 (1C, *C*(2')H). **IR** (ATR): $\tilde{\nu}$ = 3392 cm^{-1} (w), 2944 (m), 2894 (m), 2867 (m), 1744 (m), 1601 (w), 1464 (m), 1438 (m), 1375 (w), 1290 (w), 1228 (m), 1159 (m), 1086 (m), 1068 (m), 1040 (m), 997 (m), 964 (m), 921 (m), 881 (m), 842 (m), 734 (m), 675 (m), 653 (m). **UV** (CH_2Cl_2): λ_{max} (lg ε) = −. **HRESIMS**: calc. for $C_{14}H_{31}NO_3SiNa$ $[M+Na]^+$ 312.19654, found 312.19700.

3.3 Synthesis of tripeptides

3.3.1 General procedures for peptide synthesis

a) Boc removal with TFA

At 0 °C, the Boc-protected amino acids or peptides (1.0 equiv) were treated with a solution of TFA in DCM (1:5 *v*/*v*, 15-30 mL/mmol) for the specified reaction time. TFA and DCM were then removed in vacuo and the residue was azeotroped with DCM (3 × 10 mL/mmol) to remove residual TFA. After drying under high vacuum for 2–4 h, the Boc-deprotected amines were directly used in the coupling steps.

b) Saponification of methyl esters with lithium hydroxide monohydrate

At room temperature, the methyl esters were dissolved in a mixture of THF, MeOH and H_2O (3:1:1) or MeOH and H_2O (3:1) and LiOH · H_2O (3.0–10.0 equiv) was added. After stirring at room temperature for the specified time, the pH of the solution was adjusted to 2 by adding HCl (2 M aqueous) and the aqueous phase was extracted with EtOAc (3 × 75 mL/mmol). The combined organic phases were washed with saturated aqueous NaCl (20 mL/mmol), dried over $MgSO_4$ and the solvents were removed in vacuo. The free carboxylic acids were directly used in the next steps without further purification.

c) Peptide coupling with EDCI and HOBt

At room temperature, the amino component (1.3–1.4 equiv) and the carboxylic acid component (1.0 equiv) were dissolved in DMF (10–20 mL/mmol) and the mixture was cooled to 0 °C. HOBt (2.5 equiv) and EDC · HCl (2.5 equiv) were added, the mixture was warmed to room temperature and then stirred for the specified time. H_2O and EtOAc (50 mL/mmol each) were added, the phases were separated and the aqueous phase was further extracted with EtOAc (2 × 30 mL/mmol). The combined organic phases were washed with saturated aqueous NH_4Cl (3 × 20 mL/mmol) and saturated aqueous NaCl (20 mL/mmol), dried over $MgSO_4$ and the solvent was removed in vacuo. Column chromatographic separation led to isolation of the peptides.

Non-preferred coupling order

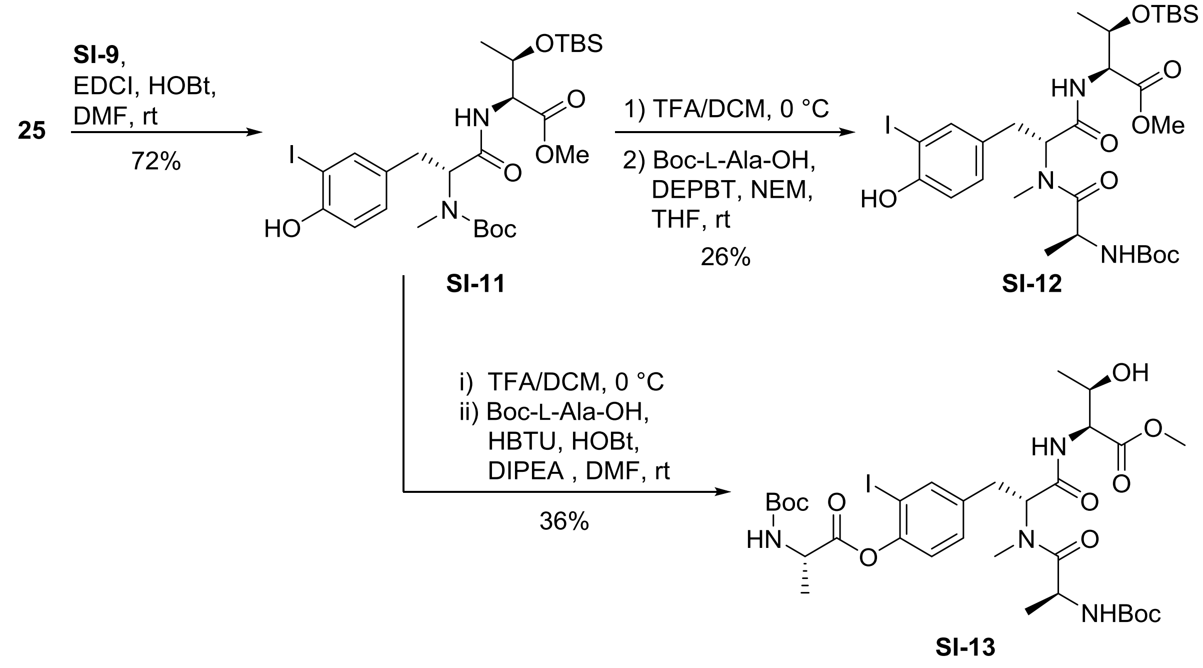

3.3.2 Methyl *N*-((*R*)-2-((Boc)(methyl)amino)-3-(4-hydroxy-3-iodophenyl)propanoyl)-*O*-(TBS)-L-threoninate (SI-11)

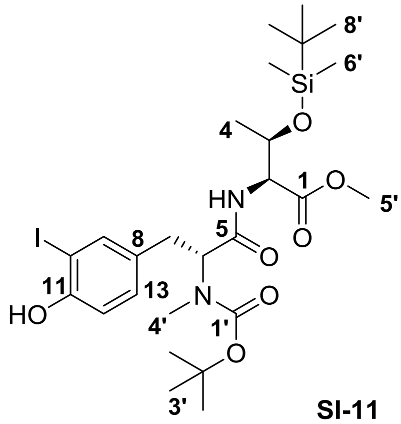

SI-11

The dipeptide **SI-11** was synthesized according to the general procedure for peptide coupling with EDCI and HOBt as detailed under 3.3.1. As the carboxylic acid component, tyrosine **25** (0.167 g, 0.396 mmol, 1.0 equiv) was used, while the TBS-protected threonine **SI-9** (0.137 g, 0.555 mmol, 1.4 equiv) was used as the amino component (5 h). After column chromatography [silica, PE/EA (1:1)], the dipeptide **SI-11** (0.185 g, 0.284 mmol, 72%) was obtained as a colorless foam. The compound produced a double NMR signal set due to the occurrence of rotamers (ratio 1.2:1 in $CDCl_3$).

mp: 55-56 °C. **TLC** [EA (100%)]: R_f = 0.89. $[\alpha]_D^{21}$ = +40.6 (*c* = 0.82, $CHCl_3$). **^{1}H NMR** (600 MHz, $CDCl_3$, major rotamer): δ = 7.51 (s, 1H, C(9)*H*), 7.11 (d, *J* = 8.1 Hz, C(13)*H*), 6.89-6.87 (m, 1H, C(12)H), 6.78 (d, *J* = 9.3 Hz, N*H*), 5.35 (br s, 1H, O*H*), 5.10 (dd, *J* = 9.7 Hz, *J* = 6.1 Hz, 1H, C(6)*H*), 4.50-4.45 (m, 2H, C(2)*H*, C(3)*H*), 3.71 (s, 3H, C(5')H_3), 3.38-3.29 (m, 1H, C(7)H_2), 2.87-2.71 (m, 1H, C(7)H_2), 2.79 (s, 3H, C(4')H_3), 1.39 (s, 9H, C(3')H_3), 1.13 (d, *J* = 6.2 Hz, 3H, C(4)H_3), 0.83 (s, 9H, C(8')H_3), 0.03 (s, 3H, C(6')H_3), −0.03 (s, 3H, C(6')H_3). **^{13}C NMR** (150.9 MHz, $CDCl_3$, major rotamer): δ = 171.2 (1C, *C*(5)), 170.9 (1C, *C*(1)), 156.5 (1C, *C*(1')), 153.4 (1C, *C*(11)), 138.3 (1C, *C*(9)H), 131.8 (1C, *C*(8)), 130.9 (1C, *C*(13)H), 114.8 (1C, *C*(12)H), 85.3 (1C, *C*(10)), 80.5 (1C, *C*(2')), 68.4 (1C, *C*(3)H), 58.9 (1C, *C*(6)H), 57.9 (1C, *C*(2)H), 52.2 (1C, *C*(5')H_3), 32.4 (1C, *C*(7)H_2), 30.5 (1C, *C*(4')H_3), 28.2 (3C, *C*(3')H_3), 25.6 (3C, *C*(8')H_3), 21.0 (1C, *C*(4)H_3), 17.8 (1C, *C*(7')), −4.4 (1C, *C*(6')H_3), −5.5 (1C, *C*(6')H_3). **^{1}H NMR** (600 MHz, $CDCl_3$, minor rotamer): δ = 7.51 (s, 1H, C(9)*H*), 7.05 (d, *J* = 7.8 Hz, C(13)*H*), 6.89-6.87 (m, 1H, C(12)H), 6.63 (d, *J* = 8.9 Hz, N*H*), 5.46 (br s, 1H, O*H*),

4.91 (dd, J = 10.6 Hz, J = 3.9 Hz, 1H, C(6)H), 4.50-4.45 (m, 2H, C(2)H, C(3)H), 3.73 (s, 3H, C(5')H_3), 3.38-3.29 (m, 1H, C(7)H_2), 2.87-2.71 (m, 1H, C(7)H_2), 2.86 (s, 3H, C(4')H_3), 1.32 (s, 9H, C(3')H_3), 1.14 (d, J = 6.1 Hz, 3H, C(4)H_3), 0.82 (s, 9H, C(8')H_3), 0.03 (s, 3H, C(6')H_3), −0.03 (s, 3H, C(6')H_3). **^{13}C NMR** (150.9 MHz, $CDCl_3$, minor rotamer): δ = 170.8 (1C, C(5)), 170.8 (1C, C(1)), 155.0 (1C, C(1')), 153.7 (1C, C(11)), 138.2 (1C, C(9)H), 132.1 (1C, C(8)), 130.8 (1C, C(13)H), 114.9 (1C, C(12)H), 85.6 (1C, C(10)), 80.7 (1C, C(2')), 68.4 (1C, C(3)H), 61.1 (1C, C(6)H), 57.9 (1C, C(2)H), 52.4 (1C, C(5')H_3), 32.4 (1C, C(7)H_2), 29.9 (1C, C(4')H_3), 28.1 (3C, C(3')H_3), 25.5 (3C, C(8')H_3), 21.0 (1C, C(4)H_3), 17.8 (1C, C(7')), −4.5 (1C, C(6')H_3), −5.4 (1C, C(6')H_3). **IR** (ATR): $\tilde{\nu}$ = 3423 cm^{-1} (w), 3308 (w, br), 2954 (m), 2931 (m), 2857 (w), 1750 (m), 1668 (m), 1604 (w), 1506 (m), 1448 (w), 1414 (w), 1389 (m), 1366 (m), 1319 (m), 1253 (m), 1209 (m), 1129 (m), 1093 (m), 1035 (m), 962 (m), 939 (m), 828 (m), 809 (m), 776 (m), 664 (m), 567 (w), 542 (m). **UV** (CH_2Cl_2): λ_{max} (lg ε) = 289 nm (3.27), 283 (3.32). **HRESIMS**: calc. for $C_{26}H_{43}IN_2O_7SiNa$ $[M+Na]^+$ 673.17764, found 673.17790.

3.3.3 Methyl *N*-((*R*)-2-((*S*)-2-((*tert*-butoxycarbonyl)amino)-*N*-methylpropanamido)-3-(4-hydroxy-3-iodophenyl)propanoyl)-*O*-(*tert*-butyldimethylsilyl)-L-threoninate (SI-12)

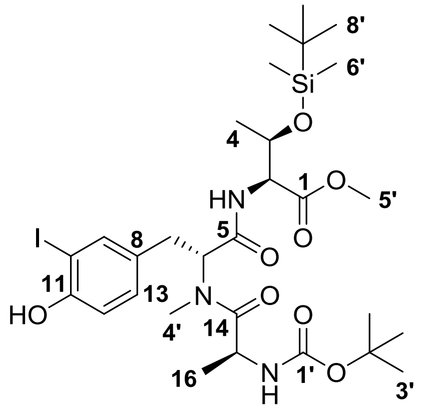

SI-12

The dipeptide **SI-11** (27.0 mg, 0.041 mmol, 1.0 equiv) was first Boc-deprotected with TFA/DCM following the general procedure detailed in 3.3.1 (3.5 h). Then, the free amine was dissolved in THF (2 mL) and the mixture was cooled to 0 °C. Boc-L-alanine (8.6 mg, 0.046 mmol, 1.1 equiv), DEPBT (14.9 mg, 0.050 mmol, 1.2 equiv) and *N*-ethylmorpholine (16 µL, 0.124 mmol, 3.0 equiv) were added in rapid succession. The reaction was brought to room temperature and stirred for 19 h. Then, H_2O (2 mL) was added and the organic solvent was removed in vacuo. The aqueous phase was extracted with EtOAc (3 × 10 mL). The combined organic phases were washed with saturated aqueous $NaHCO_3$, citric acid (10% aqueous) and saturated aqueous NaCl (5 mL each), dried over Na_2SO_4 and the solvent was removed in vacuo. After column chromatography [silica, PE/EA (1:1)], the tripeptide **SI-12** (7.7 mg, 0.011 mmol, 26%) was obtained as a yellow, solidified oil.

TLC [PE/EA (1:1)]: R_f = 0.56. $[\alpha]_D^{21}$ = +61.7 (c = 0.36, $CHCl_3$). **^{1}H NMR** (600 MHz, $CDCl_3$): δ = 7.48 (d, J = 1.8 Hz, 1H, C(9)H), 7.08 (dd, J = 8.2 Hz, J = 1.6 Hz, 1H, C(13)H), 6.87 (d, J = 8.3 Hz, 1H, C(12)H), 6.78 (d, J = 9.3 Hz, 1H, NH_{Thr}), 5.57 (d, J = 7.2 Hz, 1H, NH_{Ala}), 5.53 (dd, J = 10.5 Hz, J = 6.1 Hz, 1H, C(6)H), 5.41 (br s, 1H, OH), 4.54 (ap. quint, J = 6.8 Hz, 1H, C(15)H), 4.46 (q, J = 5.9 Hz, 1H, C(3)H), 4.40 (d, J = 9.3 Hz, 1H, C(2)H), 3.67 (s, 3H, C(5')H_3), 3.22 (dd, J = 15.2 Hz, J = 6.1 Hz, 1H, C(7)H_2), 2.94 (dd, J = 15.4 Hz, J = 10.6 Hz, 1H, C(7)H_2), 2.92 (s, 3H, C(4')H_3), 1.42 (s, 9H, C(3')H_3), 1.17 (d, J = 6.3 Hz, 3H, C(4)H_3), 0.99 (d, J = 6.7 Hz, 3H, C(16)H_3), 0.84 (s, 9H, C(8')H_3), 0.04 (s, 3H, C(6')H_3), −0.05 (s, 3H, C(6')H_3). **^{13}C NMR** (150.9 MHz, $CDCl_3$): δ = 174.5 (1C, C(14)), 170.8 (1C, C(1)), 170.3 (1C, C(5)), 154.7 (1C, C(1')), 153.7 (1C, C(11)), 138.1 (1C, C(9)H), 130.8 (1C, C(8)), 130.7 (1C, C(13)H), 114.9 (1C, C(12)H), 85.4 (1C, C(10)), 79.4 (1C, C(2')), 68.1 (1C, C(3)H), 58.1 (1C, C(2)H), 56.4 (1C, C(6)H), 52.3 (1C, C(5')H_3), 46.8 (1C, C(15)H), 31.7 (1C, C(7)H_2), 30.5 (1C,

C(4')H$_3$), 28.4 (3C, C(3')H$_3$), 25.4 (3C, C(8')H$_3$), 21.1 (1C, C(4)H$_3$), 18.9 (1C, C(16)H$_3$), 17.7 (1C, C(7')), −4.4 (1C, C(6')H$_3$), −5.5 (1C, C(6')H$_3$). **IR** (ATR): $\tilde{\nu}$ = 3424 cm^{-1} (w), 3339 (w, br), 2954 (m), 2928 (m), 2856 (m), 1748 (m), 1682 (m), 1642 (m), 1504 (m), 1411 (m), 1365 (m), 1318 (w), 1253 (m), 1211 (m), 1163 (m), 1129 (m), 1093 (m), 1051 (m), 1032 (m), 1005 (m), 965 (m), 938 (w), 837 (m), 809 (m), 777 (m), 755 (m), 665 (m), 538 (m). **UV** (CH_2Cl_2): λ_{max} (lg ε) = 289 nm (3.28), 282 (3.30). **HRESIMS**: calc. for $C_{29}H_{48}IN_3O_8SiNa$ [M+Na]$^+$ 744.21476, found 744.21507.

3.3.4 Methyl ((*R*)-3-(4-(((*tert*-butoxycarbonyl)-L-alanyl)oxy)-3-iodophenyl)-2-((*S*)-2-((*tert*-butoxycarbonyl)amino)-*N*-methylpropanamido)propanoyl)-L-threoninate (SI-13)

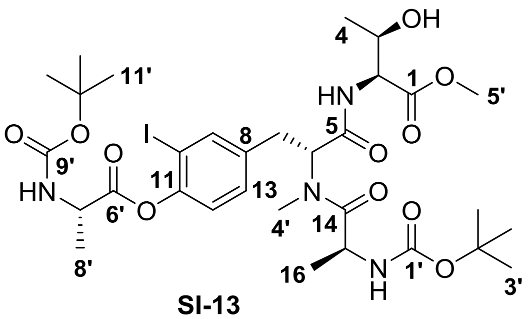

SI-13

The dipeptide **SI-11** (0.039 g, 0.060 mmol, 1.0 equiv) was first Boc-deprotected with TFA/DCM following the general procedure detailed in 3.3.1 (1 h). Then, the free amine was dissolved in DMF (2 mL) and the mixture was cooled to 0 °C. Boc-L-alanine (0.023 g, 0.120 mmol, 2.0 equiv), DIPEA (42 µL, 0.240 mmol, 4.0 equiv), HOBt (0.018 g, 0.120 mmol, 2.0 equiv) and HBTU (0.045 g, 0.120 mmol, 2.0 equiv) were added in rapid succession. The reaction was brought to room temperature and stirred for 19 h before it was quenched by addition of H_2O (10 mL). After 5 min of stirring, H_2O and EtOAc (10 mL each) were added, the phases were separated and the aqueous phase was further extracted with EtOAc (2 × 10 mL). The combined organic phases were washed with saturated aqueous NH_4Cl (3 × 10 mL) and saturated aqueous NaCl (10 mL), dried over $MgSO_4$ and the solvent was removed in vacuo. After column chromatography [silica, PE/EA (1:1)], the depsipeptide **SI-13** (0.017 g, 0.022 mmol, 36%) was isolated as a colorless oil.

TLC [PE/EA (1:1)]: R_f = 0.29. $[\alpha]_D^{21}$ = +9.3 (c = 0.85, $CHCl_3$). **^{1}H NMR** (600 MHz, $CDCl_3$): δ = 7.66 (d, J = 1.9 Hz, 1H, C(9)H), 7.23 (dd, J = 8.3 Hz, J = 1.7 Hz, 1H, C(13)H), 7.01 (d, J = 8.2 Hz, 1H, C(12)H), 6.85 (d, J = 9.0 Hz, 1H, NH_{Thr}), 5.77 (dd, J = 11.1 Hz, J = 5.6 Hz, 1H, C(6)H), 5.09 (d, J = 5.9 Hz, 2H, NH_{Ala}), 4.62-4.56 (m, 1H, C(7')H), 4.52 (dd, J = 9.0 Hz, J = 2.6 Hz, 1H, C(2)H), 4.41-4.34 (m, 2H, C(3)H, C(15)H), 3.77 (s, 3H, C(5')H_3), 3.50 (dd, J = 15.3 Hz, J = 5.6 Hz, 1H, C(7)H_2), 3.25 (d, J = 8.4 Hz, 1H, OH), 3.01 (s, 3H, C(4')H_3), 2.91 (dd, J = 15.3 Hz, J = 11.2 Hz, 1H, C(7)H_2), 1.63 (d, J = 7.3 Hz, 3H, C(8')H_3), 1.46 (s, 9H, C(11')H_3), 1.40 (s, 9H, C(3')H_3), 1.22 (d, J = 6.5 Hz, 3H, C(4)H_3), 1.00 (d, J = 7.0 Hz, 3H, C(16)H_3). **^{13}C NMR** (150.9 MHz, $CDCl_3$): δ = 174.1 (1C, C(14)), 171.1 (1C, C(1)), 171.0 (1C, C(6')), 170.2 (1C, C(5)), 156.0 (1C, C(1')), 155.1 (1C, C(9')), 149.5 (1C, C(11)), 139.4 (1C, C(9)H), 137.2 (1C, C(8)), 130.0 (1C, C(13)H), 122.6 (1C, C(12)H), 89.6 (1C, C(10)), 80.5 (1C, C(2')), 80.1 (1C, C(10')), 67.9 (1C, C(3)H), 58.0 (1C, C(2)H), 56.4 (1C, C(6)H), 52.5 (1C, C(5')H$_3$), 49.6 (1C, C(7')H), 46.7 (1C, C(15)H), 32.4 (1C, C(7)H$_2$), 30.7 (1C, C(4')H$_3$), 28.3 (6C, C(3')H$_3$, C(11')H$_3$), 20.2 (1C, C(4)H$_3$), 18.6 (1C, C(8')H$_3$), 16.5 (1C, C(16)H$_3$). **IR** (ATR): $\tilde{\nu}$ = 3337 cm^{-1} (w, br), 2977 (m), 2928 (m), 2854 (w), 1747 (m), 1684 (m), 1516 (m), 1484 (m), 1454 (m), 1392 (w), 1367 (m), 1250 (m), 1214 (m), 1162 (m), 1107 (m), 1058 (m), 1021

(m), 888 (m), 862 (m), 752 (s), 664 (m). **UV** (CH_2Cl_2): λ_{max} (lg ε) = 278 nm (2.85), 271 (2.91). **HRESIMS**: calc. for $C_{31}H_{47}IN_4O_{11}Na$ $[M+Na]^+$ 801.21782, found 801.21802.

Preferred coupling order

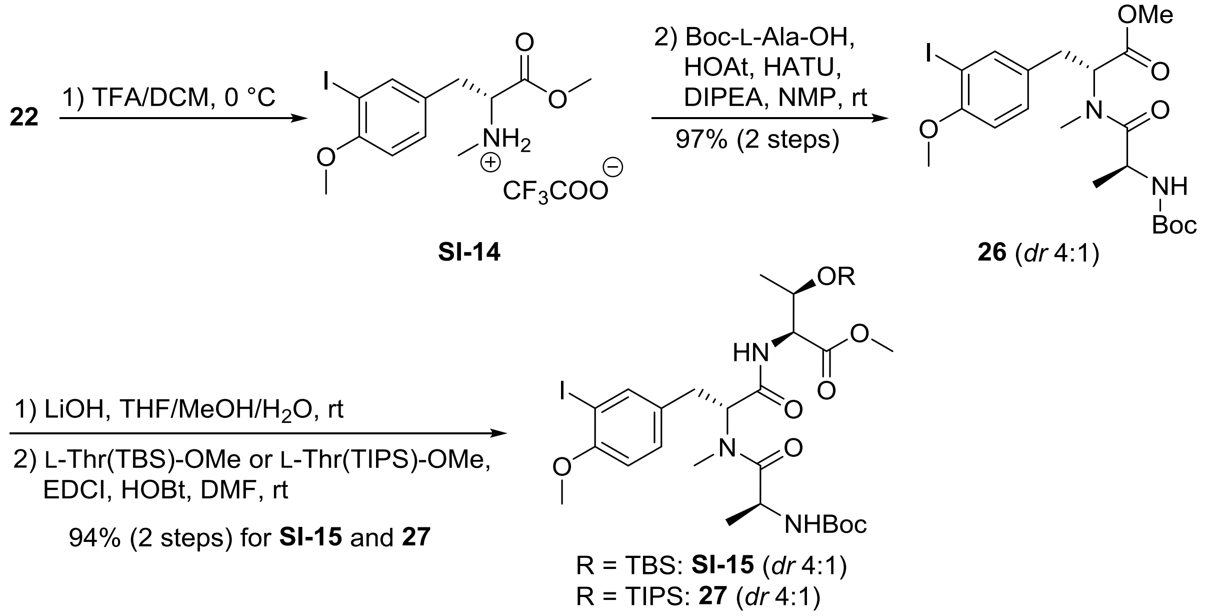

3.3.5 Methyl (*R*)-2-((*S*)-2-((*tert*-butoxycarbonyl)amino)-*N*-methylpropanamido)-3-(3-iodo-4-methoxyphenyl)propanoate (26)

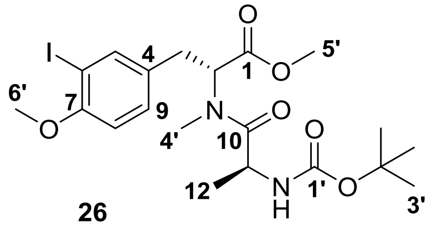

Synthesis of the dipeptide **26** was performed following a two-step procedure. First, Boc deprotection according to the general procedure as detailed in 3.3.1 was carried out starting from tyrosine **22** (60.9 mg, 0.136 mmol, 1.5 h). For coupling of the free amine **SI-14** with Boc-L-alanine, the amine (1.0 equiv) was dissolved in *N*-methyl-2-pyrrolidone (1.5 mL) at 0 °C. HOAt (18.5 mg, 0.163 mmol, 1.2 equiv), HATU (61.8 mg, 0.163 mmol, 1.2 equiv), Boc-L-Ala-OH (51.3 mg, 0.271 mmol, 2.0 equiv) and DIPEA (115 µL, 0.678 mmol, 5.0 equiv) were added and the mixture was stirred at room temperature for 5 h. The reaction was quenched by adding H_2O (1.5 mL). After 5 min of stirring, H_2O and EtOAc (10 mL each) were added, the phases were separated and the aqueous phase was further extracted with EtOAc (2 × 10 mL). The combined organic phases were washed with saturated aqueous NH_4Cl (3 × 5 mL) and saturated aqueous NaCl (5 mL), dried over $MgSO_4$ and the solvent was removed in vacuo. After column chromatography [silica, PE/EA (1:1)], the dipeptide **26** (68.1 mg, 0.131 mmol, 97% over 2 steps) was obtained as a colorless, solidified oil. The compound produced a double NMR signal set due to the occurence of diastereomers at C2 (ratio depending on the enantiomeric purity of the tyrosine **22**).

Boc deprotected amino acid **SI-14**:

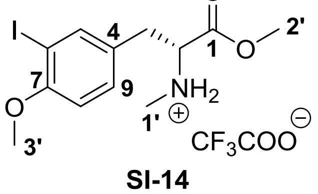

SI-14

TLC [PE/EA (3:1)]: R_f = 0.06. **^{1}H NMR** (600 MHz, $CDCl_3$): δ = 8.80 (br s, 2H, NH_2^+), 7.56 (d, J = 2.1 Hz, 1H, C(5)H), 7.15 (dd, J = 8.4 Hz, J = 2.1 Hz, 1H, C(9)H), 6.76 (d, J = 8.4 Hz, 1H, C(8)H), 4.08 (dd, J = 7.0 Hz, J = 6.0 Hz, 1H, C(2)H), 3.86 (s, 3H, C(3')H_3),

3.79 (s, 3H, C(2')H_3), 3.26 (dd, J = 14.5 Hz, J = 5.8 Hz, 1H, C(3)H_2), 3.20 (dd, J = 14.5 Hz, J = 7.1 Hz, 1H, C(3)H_2), 2.78 (s, 3H, C(1')H_3). **^{13}C NMR** (150.9 MHz, $CDCl_3$): δ = 168.1 (1C, *C*(1)), 158.1 (1C, *C*(7)), 139.9 (1C, *C*(5)H), 130.4 (1C, *C*(9)H), 126.7 (1C, *C*(4)), 111.1 (1C, *C*(8)H), 86.5 (1C, *C*(6)), 62.4 (1C, *C*(2)H), 56.3 (1C, *C*(3')H_3), 53.4 (1C, *C*(2')H_3), 34.4 (1C, *C*(3)H_2), 32.7 (1C, *C*(1')H_3). **IR** (ATR): $\tilde{\nu}$ = 3150-2350 cm^{-1} (w, br), 3012 (w), 2960 (w), 2841 (w), 2725 (w), 2451 (m), 1747 (m), 1666 (m), 1599 (m), 1492 (m), 1464 (m), 1440 (m), 1282 (m), 1257 (m), 1137 (s), 1048 (m), 1016 (m), 835 (m), 812 (m), 798 (m), 723 (m), 704 (m), 663 (w), 596 (m), 551 (w). **UV** (CH_2Cl_2): λ_{max} (lg ε) = 291 nm (3.42), 284 (3.43), 232 (3.98). **HRESIMS**: calc. for $C_{12}H_{17}INO_3$ $[M+H]^+$ 350.02476, found 350.02530.

Dipeptide **26**:
TLC [PE/EA (1:1)]: R_f = 0.55. $[\alpha]_D^{24}$ = +13.1 (c = 1.79, MeOH). **^{1}H NMR** (600 MHz, $CDCl_3$, major diastereomer): δ = 7.57 (d, J = 2.0 Hz, 1H, C(5)*H*), 7.13 (dd, J = 8.3 Hz, J = 2.5 Hz, 1H, C(9)*H*), 6.73 (d, J = 8.4 Hz, 1H, C(8)*H*), 5.43 (d, J = 7.9 Hz, 1H, N*H*), 5.25 (dd, J = 11.3 Hz, J = 5.1 Hz, 1H, C(2)*H*), 4.55-4.50 (m, 1H, C(11)*H*), 3.84 (s, 3H, C(6')H_3), 3.74 (s, 3H, C(5')H_3), 3.30 (dd, J = 14.7 Hz, J = 5.0 Hz, 1H, C(3)H_2), 2.93 (dd, J = 14.8 Hz, J = 11.4 Hz, 1H, C(3)H_2), 2.88 (s, 3H, C(4')H_3), 1.42 (s, 9H, C(3')H_3), 0.97 (d, J = 6.8 Hz, 3H, C(12)H_3). **^{13}C NMR** (150.9 MHz, $CDCl_3$, major diastereomer): δ = 173.7 (1C, *C*(10)), 170.7 (1C, *C*(1)), 157.1 (1C, *C*(7)), 155.0 (1C, *C*(1')), 139.5 (1C, *C*(5)H), 130.8 (1C, *C*(4)), 129.9 (1C, *C*(9)H), 110.8 (1C, *C*(8)H), 85.7 (1C, *C*(6)), 79.5 (1C, *C*(2')), 58.1 (1C, *C*(2)H), 56.4 (1C, *C*(6')H_3), 52.5 (1C, *C*(5')H_3), 46.6 (1C, *C*(11)H), 33.3 (1C, *C*(3)H_2), 32.5 (1C, *C*(4')H_3), 28.3 (3C, *C*(3')H_3), 18.6 (1C, *C*(12)H_3). **^{1}H NMR** (600 MHz, $CDCl_3$, minor diastereomer): δ = 7.59 (d, J = 2.2 Hz, 1H, C(5)*H*), 7.13 (dd, J = 8.3 Hz, J = 2.3 Hz, 1H, C(9)*H*), 6.73 (d, J = 8.4 Hz, 1H, C(8)*H*), 5.37 (d, J = 8.1 Hz, 1H, N*H*), 4.90 (dd, J = 10.2 Hz, J = 5.6 Hz, 1H, C(2)*H*), 4.55-4.50 (m, 1H, C(11)*H*), 3.85 (s, 3H, C(6')H_3), 3.72 (s, 3H, C(5')H_3), 3.27 (dd, J = 14.3 Hz, J = 5.2 Hz, 1H, C(3)H_2), 3.01 (dd, J = 14.6 Hz, J = 10.4 Hz, 1H, C(3)H_2), 2.87 (s, 3H, C(4')H_3), 1.42 (s, 9H, C(3')H_3), 1.29 (d, J = 6.8 Hz, 3H, C(12)H_3). **^{13}C NMR** (150.9 MHz, $CDCl_3$, minor diastereomer): δ = 173.3 (1C, *C*(10)), 170.7 (1C, *C*(1)), 157.0 (1C, *C*(7)), 155.0 (1C, *C*(1')), 139.8 (1C, *C*(5)H), 131.1 (1C, *C*(4)), 129.8 (1C, *C*(9)H), 110.8 (1C, *C*(8)H), 85.9 (1C, *C*(6)), 79.5 (1C, *C*(2')), 59.9 (1C, *C*(2)H), 56.3 (1C, *C*(6')H_3), 52.4 (1C, *C*(5')H_3), 46.5 (1C, *C*(11)H), 33.8 (1C, *C*(4')H_3), 33.0 (1C, *C*(3)H_2), 28.3 (3C, *C*(3')H_3), 18.8 (1C, *C*(12)H_3). **IR** (ATR): $\tilde{\nu}$ = 3421 cm^{-1} (w), 3325 (w), 2974 (m), 2930 (m), 2851 (w), 1740 (m), 1705 (m), 1646 (m), 1599 (w), 1490 (m), 1442 (m), 1410 (m), 1365 (m), 1279 (m), 1251 (m), 1164 (m), 1047 (m), 1016 (m), 857 (m), 811 (m), 789 (m), 749 (m), 663 (m), 557 (w). **UV** (CH_2Cl_2): λ_{max} (lg ε) = 290 nm (3.48), 283 (3.51), 231 (4.03). **HRESIMS**: calc. for $C_{20}H_{29}IN_2O_6Na$ $[M+Na]^+$ 543.09625, found 543.09666.

3.3.6 Methyl *N*-((*R*)-2-((*S*)-2-((*tert*-butoxycarbonyl)amino)-*N*-methylpropanamido)-3-(3-iodo-4-methoxyphenyl)propanoyl)-*O*-(*tert*-butyldimethylsilyl)-L-threoninate (SI-15)

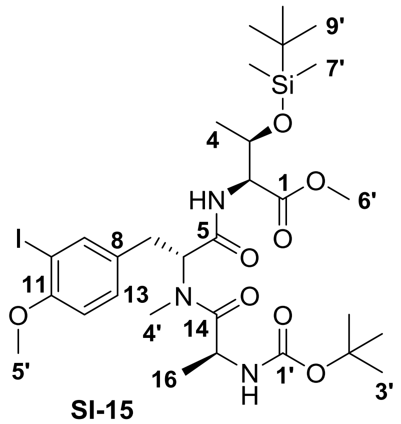

SI-15

Following the general procedures as detailed in 3.3.1, the methyl ester function of dipeptide **26** (38.9 mg, 0.075 mmol, 1.0 equiv) was first saponified with LiOH · H_2O (3.0 equiv) in THF/MeOH/H_2O (2 h). After workup, the free carboxylic acid (41.2 mg) was obtained as a colorless, solidified oil. The acid was then coupled with the TBS-protected threonine **SI-9** (25.9 mg, 0.105 mmol, 1.4 equiv) following the procedure for the EDCI/HOBt coupling (14 h). After column chromatography [silica, PE/EA (1:1)], the tripeptide **SI-15** (51.6 mg, 0.070 mmol, 94% over 2 steps) was obtained as a colorless foam. The compound produced a double NMR signal set due to the occurence of diastereomers at C6 (ratio depending on the *dr* of dipeptide **26**, best dr 4:1).

mp: 60-62 °C. **TLC** [PE/EA (1:1)]: R_f = 0.61. $[\alpha]_D^{23}$ = +16.8 (*c* = 0.75, *dr* 4:1, MeOH). **^{1}H NMR** (600 MHz, $CDCl_3$, major diastereomer): δ = 7.59 (d, *J* = 2.2 Hz, 1H, C(9)*H*), 7.15 (dd, *J* = 8.3 Hz, *J* = 2.2 Hz, 1H, C(13)*H*), 6.77 (br d, *J* = 9.3 Hz, 1H, NH_{Thr}), 6.72 (d, *J* = 8.4 Hz, 1H, C(12)*H*), 5.57 (d, *J* = 7.4 Hz, 1H, NH_{Ala}), 5.54 (dd, *J* = 10.6 Hz, *J* = 6.1 Hz, 1H, C(6)*H*), 4.55-4.51 (m, 1H, C(15)*H*), 4.46 (qd, *J* = 6.2 Hz, *J* = 1.5 Hz, 1H, C(3)*H*), 4.41 (dd, *J* = 9.3 Hz, *J* = 1.5 Hz, 1H, C(2)*H*), 3.83 (s, 3H, C(5')H_3), 3.68 (s, 3H, C(6')H_3), 3.23 (dd, *J* = 15.2 Hz, *J* = 6.1 Hz, 1H, C(7)H_2), 2.96-2.92 (m, 1H, C(7)H_2), 2.92 (s, 3H, C(4')H_3), 1.42 (s, 9H, C(3')H_3), 1.17 (d, *J* = 6.3 Hz, 3H, C(4)H_3), 0.99 (d, *J* = 6.8 Hz, 3H, C(16)H_3), 0.84 (s, 9H, C(9')H_3), 0.04 (s, 3H, C(7')H_3), −0.05 (s, 3H, C(7')H_3). **^{13}C NMR** (150.9 MHz, $CDCl_3$, major diastereomer): δ = 174.5 (1C, *C*(14)), 170.9 (1C, *C*(1)), 170.3 (1C, *C*(5)), 157.0 (1C, *C*(11)), 154.7 (1C, *C*(1')), 139.4 (1C, *C*(9)H), 130.9 (1C, *C*(8)), 129.9 (1C, *C*(13)H), 110.8 (1C, *C*(12)H), 85.8 (1C, *C*(10)), 79.3 (1C, *C*(2')), 68.1 (1C, *C*(3)H), 58.1 (1C, *C*(2)H), 56.4 (1C, *C*(6)H), 56.4 (1C, *C*(5')H_3), 52.3 (1C, *C*(6')H_3), 46.8 (1C, *C*(15)H), 31.7 (1C, *C*(7)H_2), 30.5 (1C, *C*(4')H_3), 28.4 (3C, *C*(3')H_3), 25.4 (3C, *C*(9')H_3), 21.1 (1C, *C*(4)H_3), 18.9 (1C, *C*(16)H_3), 17.7 (1C, *C*(8')), −4.4 (1C, *C*(7')H_3), −5.5 (1C, *C*(7')H_3). **^{1}H NMR** (600 MHz, $CDCl_3$, minor diastereomer): δ = 7.63 (d, *J* = 2.1 Hz, 1H, C(9)*H*), 7.16 (dd, *J* = 8.2 Hz, *J* = 2.4 Hz, 1H, C(13)*H*), 6.72 (d, *J* = 8.4 Hz, 1H, C(12)*H*), 6.61 (br d, *J* = 9.3 Hz, 1H, NH_{Thr}), 5.36-5.32 (m, 1H, C(6)*H*), 5.33 (d, *J* = 8.2 Hz, 1H, NH_{Ala}), 4.55-4.51 (m, 1H, C(15)*H*), 4.51 (dd, *J* = 9.4 Hz, *J* = 1.7 Hz, 1H, C(2)*H*), 4.44 (qd, *J* = 6.2 Hz, *J* = 1.7 Hz, 1H, C(3)*H*), 3.84 (s, 3H, C(5')H_3), 3.69 (s, 3H, C(6')H_3), 3.27 (dd, *J* = 14.9 Hz, *J* = 7.1 Hz, 1H, C(7)H_2), 2.96 (s, 3H, C(4')H_3), 2.96-2.92 (m, 1H, C(7)H_2), 1.43 (s, 9H, C(3')H_3), 1.28 (d, *J* = 6.8 Hz, 3H, C(16)H_3), 1.09 (d, *J* = 6.3 Hz, 3H, C(4)H_3), 0.84 (s, 9H, C(9')H_3), 0.02 (s, 3H, C(7')H_3), −0.03 (s, 3H, C(7')H_3). **^{13}C NMR** (150.9 MHz, $CDCl_3$, minor diastereomer): δ = 173.6 (1C, *C*(14)), 170.6 (1C, *C*(1)), 169.9 (1C, *C*(5)), 156.9 (1C, *C*(11)), 154.9 (1C, *C*(1')), 139.7 (1C, *C*(9)H), 131.0 (1C, *C*(8)), 129.8 (1C, *C*(13)H), 110.8 (1C, *C*(12)H), 85.9 (1C, *C*(10)), 79.6 (1C, *C*(2')), 68.6 (1C, *C*(3)H), 58.1 (1C, *C*(6)H), 57.7 (1C, *C*(2)H), 56.3 (1C, *C*(5')H_3), 52.4 (1C, *C*(6')H_3), 46.6 (1C, *C*(15)H), 32.2 (1C, *C*(7)H_2), 31.5 (1C, *C*(4')H_3), 28.4 (3C, *C*(3')H_3), 25.6 (3C, *C*(9')H_3), 20.9 (1C, *C*(4)H_3), 19.0 (1C, *C*(16)H_3), 17.8 (1C, *C*(8')), −4.4 (1C, *C*(7')H_3), −5.4 (1C, *C*(7')H_3). **IR**

(ATR): $\tilde{\nu}$ = 3430 cm^{-1} (w), 3345 (w, br), 2953 (m), 2932 (m), 2857 (w), 1748 (m), 1709 (m), 1688 (m), 1643 (m), 1491 (m), 1407 (m), 1365 (m), 1316 (w), 1279 (m), 1252 (m), 1205 (w), 1164 (m), 1128 (w), 1094 (m), 1048 (m), 1018 (m), 966 (m), 938 (w), 836 (m), 810 (m), 777 (m), 663 (m), 547 (m). **UV** (CH_2Cl_2): λ_{max} (lg ε) = 291 nm (3.44), 284 (3.45). **HRESIMS**: calc. for $C_{30}H_{50}IN_3O_8SiNa$ $[M+Na]^+$ 758.23041, found 758.23059.

3.3.7 Methyl *N*-((*R*)-2-((*S*)-2-((*tert*-butoxycarbonyl)amino)-*N*-methylpropanamido)-3-(3-iodo-4-methoxyphenyl)propanoyl)-*O*-(triisopropylsilyl)-L-threoninate (27)

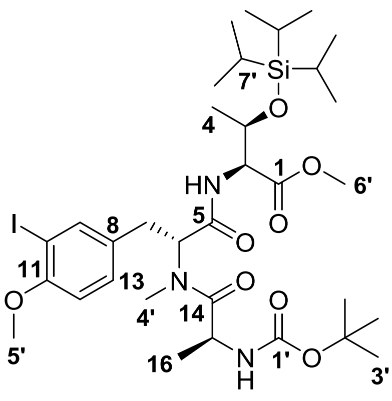

27

Following the general procedures as detailed in 3.3.1, the methyl ester function of dipeptide **26** (31.9 mg, 0.061 mmol, 1.0 equiv) was first saponified with LiOH · H_2O (3.0 equiv) in THF/MeOH/H_2O (2 h). After workup, the carboxylic acid (32.7 mg) was obtained as a colorless, solidified oil. The acid was then coupled with the TIPS-protected threonine **SI-10** (23.1 mg, 0.080 mmol, 1.3 equiv) following the procedure for the EDCI/HOBt coupling (5 h). After column chromatography [silica, PE/EA (3:1→1:1)], the tripeptide **27** (44.9 mg, 0.058 mmol, 94% over 2 steps) was obtained as a colorless, solidified oil. The compound produced a double NMR signal set due to the occurence of diastereomers at C6 (ratio depending on the *dr* of dipeptide **26**, best *dr* 4:1).

TLC [PE/EA (3:1)]: R_f = 0.21. $[\alpha]_D^{24}$ = +18.4 (*c* = 1.07, *dr* 4:1, MeOH). **^{1}H NMR** (600 MHz, $CDCl_3$, major diastereomer): δ = 7.59 (d, *J* = 2.1 Hz, 1H, C(9)*H*), 7.15 (dd, *J* = 8.3 Hz, *J* = 2.2 Hz, 1H, C(13)*H*), 6.74 (d, *J* = 9.0 Hz, 1H, NH_{Thr}), 6.71 (d, *J* = 8.4 Hz, 1H, C(12)*H*), 5.59-5.56 (m, 2H, C(6)*H*, NH_{Ala}), 4.66 (qd, *J* = 6.3 Hz, *J* = 1.5 Hz, 1H, C(3)*H*), 4.56-4.48 (m, 1H, C(15)*H*), 4.43 (dd, *J* = 9.3 Hz, *J* = 1.4 Hz, 1H, C(2)*H*), 3.83 (s, 3H, C(5')H_3), 3.68 (s, 3H, C(6')H_3), 3.27 (dd, *J* = 15.0 Hz, *J* = 5.6 Hz, 1H, C(7)H_2), 2.93 (s, 3H, C(4')H_3), 2.93 (dd, *J* = 14.9 Hz, *J* = 11.0 Hz, 1H, C(7)H_2), 1.42 (s, 9H, C(3')H_3), 1.23 (d, *J* = 6.3 Hz, 3H, C(4)H_3), 1.04-0.99 (m, 21H, C(7')*H*, C(8')H_3), 0.95 (d, *J* = 6.7 Hz, 3H, C(16)H_3). **^{13}C NMR** (150.9 MHz, $CDCl_3$, major diastereomer): δ = 174.3 (1C, *C*(14)), 170.8 (1C, *C*(1)), 170.2 (1C, *C*(5)), 157.0 (1C, *C*(11)), 154.6 (1C, *C*(1')), 139.4 (1C, *C*(9)H), 130.9 (1C, *C*(8)), 129.9 (1C, *C*(13)H), 110.8 (1C, *C*(12)H), 85.8 (1C, *C*(10)), 79.3 (1C, *C*(2')), 68.6 (1C, *C*(3)H), 58.2 (1C, *C*(2)H), 56.5 (1C, *C*(6)H), 56.4 (1C, *C*(5')H_3), 52.2 (1C, *C*(6')H_3), 46.8 (1C, *C*(15)H), 31.8 (1C, *C*(7)H_2), 30.5 (1C, *C*(4')H_3), 28.4 (3C, *C*(3')H_3), 21.4 (1C, *C*(4)H_3), 18.9 (1C, *C*(16)H_3), 17.9 (3C, *C*(8')H_3), 17.9 (3C, *C*(8')H_3), 12.4 (3C, *C*(7')H). **^{1}H NMR** (600 MHz, $CDCl_3$, minor diastereomer): δ = 7.63 (d, *J* = 2.1 Hz, 1H, C(9)*H*), 7.16 (dd, *J* = 8.3 Hz, *J* = 2.1 Hz, 1H, C(13)*H*), 6.72 (d, *J* = 8.2 Hz, 1H, C(12)*H*), 6.62 (d, *J* = 9.2 Hz, 1H, NH_{Thr}), 5.32-5.27 (m, 2H, C(6)*H*, NH_{Ala}), 4.63 (qd, *J* = 6.3 Hz, *J* = 1.7 Hz, 1H, C(3)*H*), 4.56-4.48 (m, 2H, C(2)*H*, C(15)*H*), 3.84 (s, 3H, C(5')H_3), 3.68 (s, 3H, C(6')H_3), 3.27 (dd, *J* = 14.9 Hz, *J* = 7.2 Hz, 1H, C(7)H_2), 2.97 (dd, *J* = 14.8 Hz, *J* = 9.0 Hz, 1H, C(7)H_2), 2.96 (s, 3H, C(4')H_3), 1.43 (s, 9H, C(3')H_3), 1.26 (d, *J* = 6.8 Hz, 3H, C(16)H_3), 1.17 (d, *J* = 6.3 Hz, 3H, C(4)H_3), 1.04-0.99 (m, 21H, C(7')*H*, C(8')H_3). **^{13}C NMR** (150.9 MHz, $CDCl_3$, minor diastereomer): δ = 173.5 (1C, *C*(14)), 170.6 (1C, *C*(1)), 170.0 (1C, *C*(5)), 156.9 (1C, *C*(11)), 154.9 (1C, *C*(1')), 139.8 (1C,

C(9)H), 131.0 (1C, *C*(8)), 129.9 (1C, *C*(13)H), 110.8 (1C, *C*(12)H), 85.9 (1C, *C*(10)), 79.6 (1C, *C*(2‘)), 68.8 (1C, *C*(3)H), 58.6 (1C, *C*(6)H), 58.0 (1C, *C*(2)H), 56.3 (1C, *C*(5')H_3), 52.3 (1C, *C*(6')H_3), 46.6 (1C, *C*(15)H), 32.4 (1C, *C*(7)H_2), 31.8 (1C, *C*(4')H_3), 28.4 (3C, *C*(3‘)H_3), 21.2 (1C, *C*(4)H_3), 18.9 (1C, *C*(16)H_3), 18.0 (3C, *C*(8‘)H_3), 17.9 (3C, *C*(8‘)H_3), 12.4 (3C, *C*(7‘)H). **IR** (ATR): $\tilde{\nu}$ = 3430 cm^{-1} (w), 3348 (w, br), 2942 (m), 2867 (m), 1749 (m), 1710 (m), 1688 (m), 1645 (m), 1492 (m), 1461 (m), 1406 (w), 1366 (m), 1316 (w), 1278 (m), 1253 (m), 1164 (m), 1129 (m), 1097 (m), 1049 (m), 1017 (m), 965 (w), 919 (w), 882 (m), 799 (w), 752 (m), 679 (m), 558 (w), 535 (w). **UV** (CH_2Cl_2): λ_{max} (lg ε) = 291 nm (3.44), 283 (3.46), 231 (4.05). **HRESIMS**: calc. for $C_{33}H_{56}IN_3O_8SiNa$ $[M+Na]^+$ 800.27736, found 800.27751.

3.4 Synthesis of “open-chain seragamide A”

3.4.1 General procedure for BEP amide coupling

At 0 °C, the amino component (1.09–1.2 equiv, TFA or HCl salt) was dissolved in DCM (35 mL/mmol) and DIPEA (5 equiv) was added. After 5 min of stirring to removed excess acid, a solution of the carboxylic acid coupling partner (1.0 equiv) in DCM (15 mL/mmol) as well as BEP (1.05–1.25 equiv) were added, the mixture was brought to room temperature and was stirred for the specified time. Then, the reaction was diluted with EtOAc (200 mL/mmol) and the organic solution was washed with saturated aqueous NH_4Cl, saturated aqueous $NaHCO_3$ and saturated aqueous NaCl (50 mL/mmol each), dried over $MgSO_4$ and the solvents were removed in vacuo. The amides were purified by column chromatography.

Synthesis of open-chain seragamide A

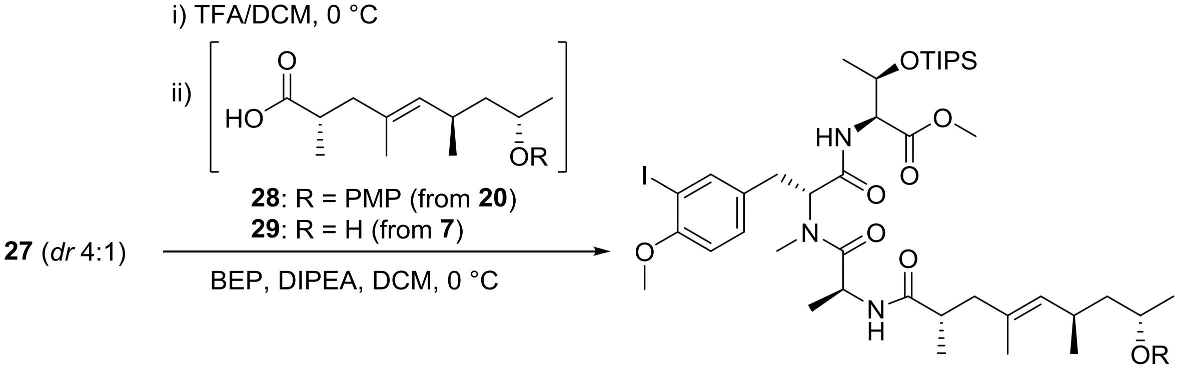

30: R = PMP (64%, *dr* 8:1)
31: R = H (60%, *dr* 9:1)

3.4.2 Methyl (2*S*,3*R*)-2-(((*R*)-3-(3-iodo-4-methoxyphenyl)-2-((*S*)-2-((2*S*,6*R*,8*S*,*E*)-8-(4-methoxyphenoxy)-2,4,6-trimethylnon-4-enamido)-*N*-methylpropanamido)propanoyl)oxy)-3-((triisopropylsilyl)oxy)butanoate (30)

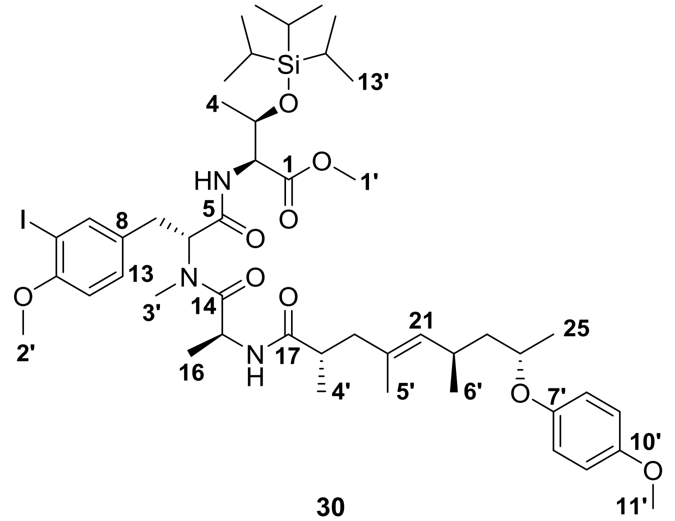

30

The methyl ester function of the polyketide **20** (30.8 mg, 0.092 mmol, 1.0 equiv) was first saponified with LiOH · H_2O (10.0 equiv) in MeOH/H_2O following the general procedure for saponification as detailed in 3.3.1 (19 h). After workup, the crude carboxylic acid **28** was obtained. In parallel, the tripeptide **27** (78.0 mg, 0.100 mmol, 1.09 equiv) was Boc-deprotected with TFA/DCM following the general procedure as detailed in 3.3.1 with a differing TFA/DCM ratio of 1:10 to suppress partial TIPS deprotection (4 h). Both coupling partners were then coupled with BEP (1.25 equiv) and DIPEA (5.0 equiv), following the general procedure as detailed in 3.4.1 (1.5 h). After column chromatography [silica, PE/MTBE (1:3)], the "open-chain seragamide A" **30** (57.6 mg, 0.059 mmol, 64% over 2 linear steps) was obtained as a colorless oil (mixture of two diastereomers at C6, *dr* 8:1).

TLC [PE/MTBE (1:3)]: R_f = 0.51. $[\alpha]_D^{24}$ = +61.9 (*c* = 0.67, MeOH). **^{1}H NMR** (600 MHz, $CDCl_3$): δ = 7.60 (d, *J* = 2.1 Hz, 1H, C(9)*H*), 7.16 (dd, *J* = 8.4 Hz, *J* = 2.0 Hz, 1H, C(13)*H*), 6.82-6.78 (m, 4H, C(8')*H*, C(9')*H*), 6.72 (d, *J* = 8.4 Hz, 1H, C(12)*H*), 6.71 (d, *J* = 8.7 Hz, 1H, NH_{Thr}), 6.60 (d, *J* = 7.0 Hz, 1H, NH_{Ala}), 5.53 (dd, *J* = 10.2 Hz, *J* = 6.2 Hz, 1H, C(6)*H*), 5.01 (ap. d, *J* = 9.4 Hz, 1H, C(21)*H*), 4.75 (ap. quint, *J* = 6.8 Hz, 1H, C(15)*H*), 4.64 (qd, *J* = 6.1 Hz, *J* = 1.3 Hz, 1H, C(3)*H*), 4.46 (dd, *J* = 9.4 Hz, *J* = 1.4 Hz, 1H, C(2)*H*), 4.22-4.17 (m, 1H, C(24)*H*), 3.83 (s, 3H, C(2')H_3), 3.76 (s, 3H, C(11')H_3), 3.68 (s, 3H, C(1')H_3), 3.27 (dd, *J* = 15.0 Hz, *J* = 6.1 Hz, 1H, C(7)H_2), 2.94 (s, 3H, C(3')H_3), 2.92 (dd, *J* = 14.9 Hz, *J* = 10.3 Hz, 1H, C(7)H_2), 2.57-2.50 (m, 1H, C(22)*H*), 2.44-2.38 (m, 1H, C(18)*H*), 2.34 (dd, *J* = 13.5 Hz, *J* = 5.4 Hz, 1H, C(19)H_2), 2.04 (dd, *J* = 13.6 Hz, *J* = 9.7 Hz, 1H, C(19)H_2), 1.72 (ddd, *J* = 13.9 Hz, *J* = 8.3 Hz, *J* = 6.3 Hz, 1H, C(23)H_2), 1.59 (d, *J* = 1.1 Hz, 3H, C(5')H_3), 1.45-1.40 (m, 1H, C(23)H_2), 1.24 (d, *J* = 6.0 Hz, 3H, C(25)H_3), 1.21 (d, *J* = 6.3 Hz, 3H, C(4)H_3), 1.06 (d, *J* = 6.9 Hz, 3H, C(4')H_3), 1.02-0.99 (m, 21H, C(12')*H*, C(13')H_3), 0.98 (d, *J* = 6.7 Hz, 3H, C(16)H_3), 0.92 (d, *J* = 6.7 Hz, 3H, C(6')H_3). **^{13}C NMR** (150.9 MHz, $CDCl_3$): δ = 175.0 (1C, *C*(17)), 174.0 (1C, *C*(14)), 170.9 (1C, *C*(1)), 170.0 (1C, *C*(5)), 157.0 (1C, *C*(11)), 153.7 (1C, *C*(10')), 152.0 (1C, *C*(7')), 139.5 (1C, *C*(9)H), 133.6 (1C, *C*(21)H), 130.9 (1C, *C*(8)), 130.8 (1C, *C*(20)), 129.9 (1C, *C*(13)H), 117.2 + 114.6 (4C, *C*(8')H, *C*(9')H), 110.8 (1C, *C*(12)H), 85.8 (1C, *C*(10)), 73.0 (1C, *C*(24)H), 68.6 (1C, *C*(3)H), 58.1 (1C, *C*(2)H), 56.9 (1C, *C*(6)H), 56.4 (1C, *C*(2')H_3), 55.7 (1C, *C*(11')H_3), 52.3 (1C, *C*(1')H_3), 45.8 (1C, *C*(15)H), 44.1 (1C, *C*(23)H_2), 43.7 (1C, *C*(19)H_2), 38.7 (1C, *C*(18)H), 31.9 (1C, *C*(7)H_2), 30.6 (1C, *C*(3')H_3), 29.2 (1C, *C*(22)H), 21.3 (1C, *C*(4)H_3), 21.1 (1C, *C*(6')H_3), 19.8 (1C, *C*(25)H_3), 18.2 (1C, *C*(16)H_3), 17.9 (3C, *C*(13')H_3), 17.9 (3C, *C*(13')H_3), 16.7 (1C, *C*(4')H_3), 15.7 (1C, *C*(5')H_3), 12.4 (3C, *C*(12')H). **^{15}N NMR** (60.8 MHz, $CDCl_3$): δ = −262.2 (*N*H_{Ala}), −270.0 (N_{Tyr}), −273.7 (*N*H_{Thr}). **IR** (ATR): $\tilde{\nu}$ = 3410 cm^{-1} (w), 3333 (w, br), 2939 (m), 2867 (m), 1747 (w),

1683 (m), 1642 (m), 1504 (m), 1461 (m), 1408 (w), 1378 (w), 1282 (w), 1254 (m), 1227 (m), 1180 (w), 1154 (w), 1130 (w), 1099 (w), 1042 (w), 942 (w), 883 (w), 825 (w), 738 (w), 681 (w). **UV** (CH_2Cl_2): λ_{max} (lg ε) = 291 nm (3.67). **HRESIMS**: calc. for $C_{47}H_{74}IN_3O_9SiNa$ $[M+Na]^+$ 1002.41312; found 1002.41380.

3.4.3 Methyl *N*-((*R*)-2-((*S*)-2-((2*S*,6*R*,8*S*,*E*)-8-hydroxy-2,4,6-trimethylnon-4-enamido)-*N*-methylpropanamido)-3-(3-iodo-4-methoxyphenyl)propanoyl)-*O*-(triisopropylsilyl)-L-threoninate (31)

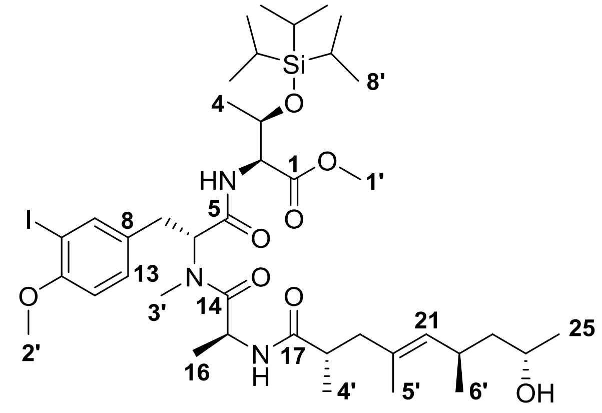

31

At room temperature, the polyketide **20** (75.7 mg, 0.226 mmol, 1.0 equiv) was dissolved in MeCN (8 mL) and H_2O (2 mL) The solution was cooled to 0 °C and cerium ammonium nitrate (272.9 mg, 0.498 mmol, 2.2 equiv) was added. After 10 min of stirring, the mixture was diluted with H_2O (40 mL). The aqueous phase was extracted with MTBE (3 × 25 mL), the combined organic phases were dried over $MgSO_4$ and the solvents were removed in vacuo. The PMP-deprotected polyketide **7** was directly submitted to a saponification with LiOH · H_2O (5.0 equiv) in MeOH/H_2O following the general procedure for saponification as detailed in 3.3.1 (26 h). After workup, the crude carboxylic acid **29** was obtained. In parallel, the tripeptide **27** (176.0 mg, 0.220 mmol, 1.0 equiv) was Boc-deprotected with TFA/DCM following the general procedure as detailed in 3.3.1 with a differing TFA/DCM ratio of 1:10 to suppress partial TIPS deprotection (4 h). Both coupling partners were then coupled with BEP (1.25 equiv) and DIPEA (5.0 equiv), following the general procedure as detailed in 3.4.1 (45 min). After column chromatography [silica, EA (100%)], the PMP-free “open-chain seragamide A” **31** (118.2 mg, 0.135 mmol, 60% over 3 linear steps) was obtained as a colorless, solidified oil (mixture of two diastereomers at C6, dr 9:1).

TLC [EA (100%)]: R_f = 0.63. $[\alpha]_D^{23}$ = +12.2 (c = 0.58, MeOH). **^{1}H NMR** (600 MHz, $CDCl_3$): δ = 7.60 (d, J = 2.2 Hz, 1H, C(9)H), 7.16 (dd, J = 8.4 Hz, J = 2.2 Hz, 1H, C(13)H), 6.77 (d, J = 9.3 Hz, 1H, NH_{Thr}), 6.72 (d, J = 8.5 Hz, 1H, C(12)H), 6.61 (d, J = 7.0 Hz, 1H, NH_{Ala}), 5.52 (dd, J = 10.1 Hz, J = 6.4 Hz, 1H, C(6)H), 5.02 (qd, J = 9.6 Hz, J = 1.0 Hz, 1H, C(21)H), 4.75 (ap. quint, J = 6.7 Hz, 1H, C(15)H), 4.63 (qd, J = 6.3 Hz, J = 1.6 Hz, 1H, C(3)H), 4.47 (dd, J = 9.4 Hz, J = 1.7 Hz, 1H, C(2)H), 3.84 (s, 3H, C(2')H_3), 3.77 (ap. ddd, J = 11.8 Hz, J = 5.9 Hz, J = 1.3 Hz, 1H, C(24)H), 3.69 (s, 3H, C(1')H_3), 3.27 (dd, J = 14.9 Hz, J = 6.3 Hz, 1H, C(7)H_2), 2.94 (s, 3H, C(3')H_3), 2.91 (dd, J = 14.9 Hz, J = 10.1 Hz, 1H, C(7)H_2), 2.52-2.44 (m, 1H, C(22)H), 2.44-2.37 (m, 1H, C(18)H), 2.31 (dd, J = 13.5 Hz, J = 6.4 Hz, 1H, C(19)H_2), 2.04 (ddd, J = 13.8 Hz, J = 8.7 Hz, J = 0.6 Hz, 1H, C(19)H_2), 1.85 (br s, 1H, OH), 1.59 (d, J = 1.2 Hz, 3H, C(5')H_3), 1.46-1.35 (m, 2H, C(23)H_2), 1.20 (d, J = 6.3 Hz, 3H, C(4)H_3), 1.15 (d, J = 6.2 Hz, 3H, C(25)H_3), 1.06 (d, J = 6.9 Hz, 3H, C(4')H_3), 1.03-1.01 (m, 21H, C(7')H, C(8')H_3), 0.99 (d, J = 6.5 Hz, 3H, C(16)H_3), 0.91 (d, J = 6.7 Hz, 3H, C(6')H_3). **^{13}C NMR** (150.9 MHz, $CDCl_3$): δ = 175.0 (1C, C(17)), 173.9 (1C, C(14)), 170.9 (1C, C(1)), 169.9 (1C,

C(5)), 157.0 (1C, *C*(11)), 139.5 (1C, *C*(9)H), 133.6 (1C, *C*(21)H), 131.2 (1C, *C*(20)), 130.8 (1C, *C*(8)), 129.9 (1C, *C*(13)H), 110.8 (1C, *C*(12)H), 85.8 (1C, *C*(10)), 68.6 (1C, *C*(3)H), 66.9 (1C, *C*(24)H), 58.0 (1C, *C*(2)H), 56.9 (1C, *C*(6)H), 56.3 (1C, *C*(2')H_3), 52.2 (1C, *C*(1')H_3), 47.1 (1C, *C*(23)H_2), 45.8 (1C, *C*(15)H), 43.5 (1C, *C*(19)H_2), 39.1 (1C, *C*(18)H), 32.0 (1C, *C*(7)H_2), 30.6 (1C, *C*(3')H_3), 30.1 (1C, *C*(22)H), 23.5 (1C, *C*(25)H_3), 21.2 (1C, *C*(6')H_3), 21.2 (1C, *C*(4)H_3), 18.1 (1C, *C*(16)H_3), 17.9 (3C, *C*(8')H_3), 17.9 (3C, *C*(8')H_3), 17.0 (1C, *C*(4')H_3), 16.0 (1C, *C*(5')H_3), 12.4 (3C, *C*(7')H). **^{15}N NMR** (60.8 MHz, $CDCl_3$): $\delta = -262.0$ (NH_{Ala}), −269.6 (N_{Tyr}), −273.3 (NH_{Thr}). **IR** (ATR): $\tilde{\nu}$ = 3411 cm^{-1} (w, br), 3341 (w, br), 2931 (m), 2867 (m), 1744 (m), 1637 (m), 1492 (m), 1458 (m), 1408 (m), 1376 (m), 1314 (w), 1280 (m), 1255 (m), 1207 (w), 1153 (w), 1127 (m), 1097 (m), 1047 (m), 1015 (m), 962 (w), 935 (w), 882 (m), 804 (m), 750 (m), 680 (m), 586 (w), 549 (w). **UV** (CH_2Cl_2): λ_{max} (lg ε) = 291 nm (3.46), 284 (3.47), 232 (4.08). **HRESIMS**: calc. for $C_{40}H_{68}IN_3O_8SiNa$ $[M+Na]^+$ 896.37126; found 896.37126.

4 ^{1}H and ^{13}C NMR spectra

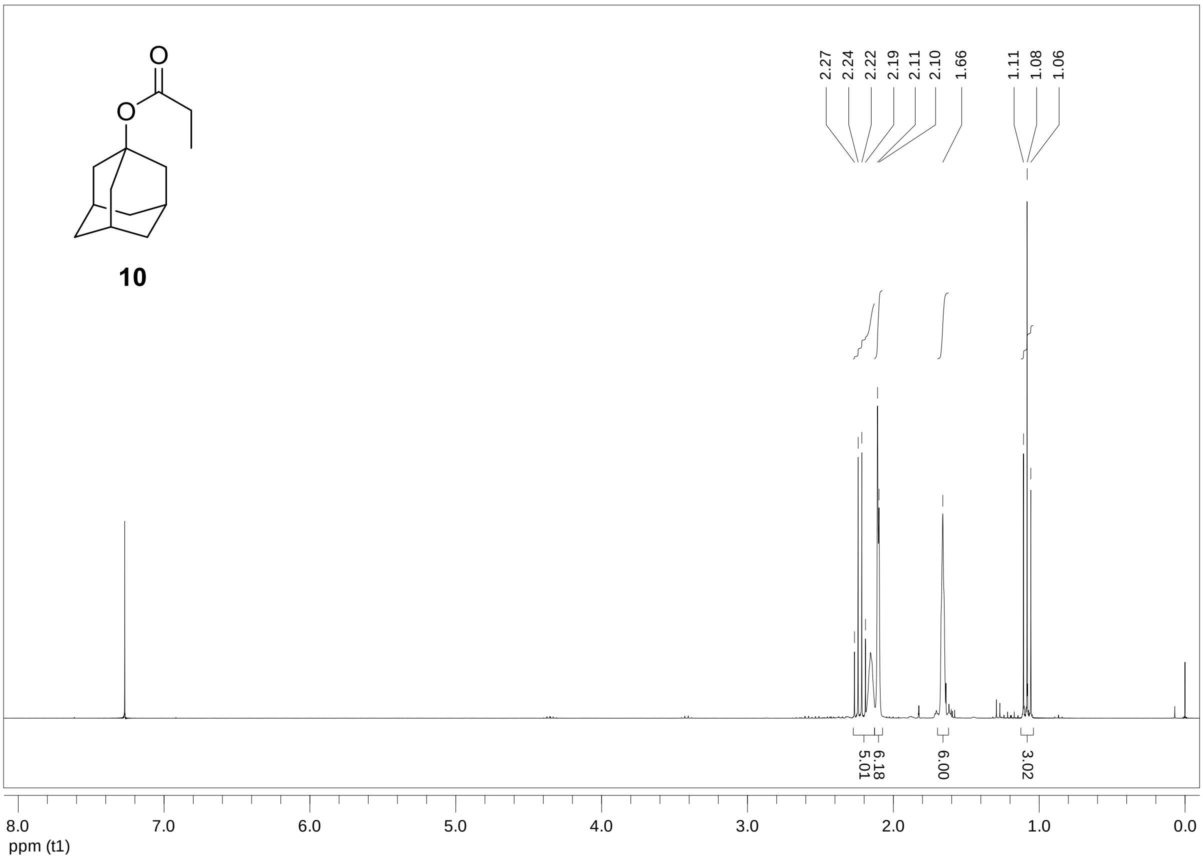

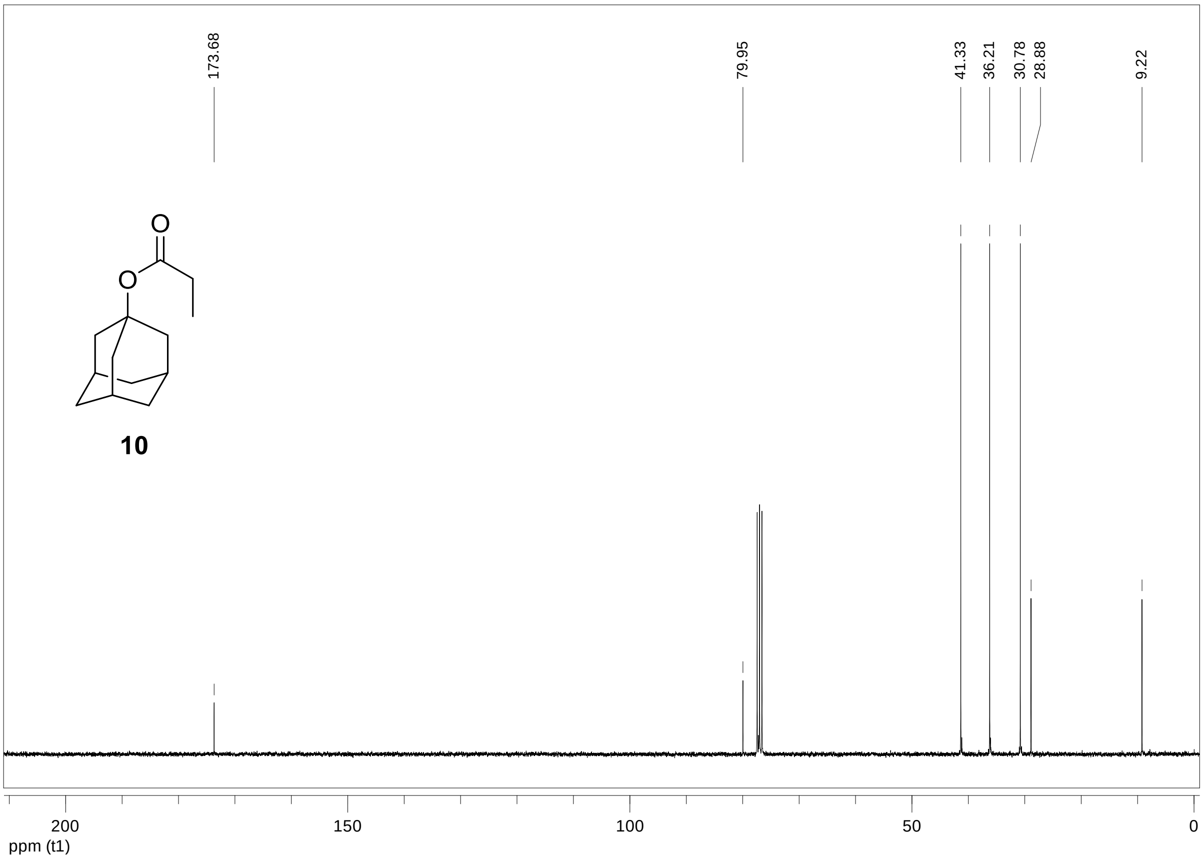

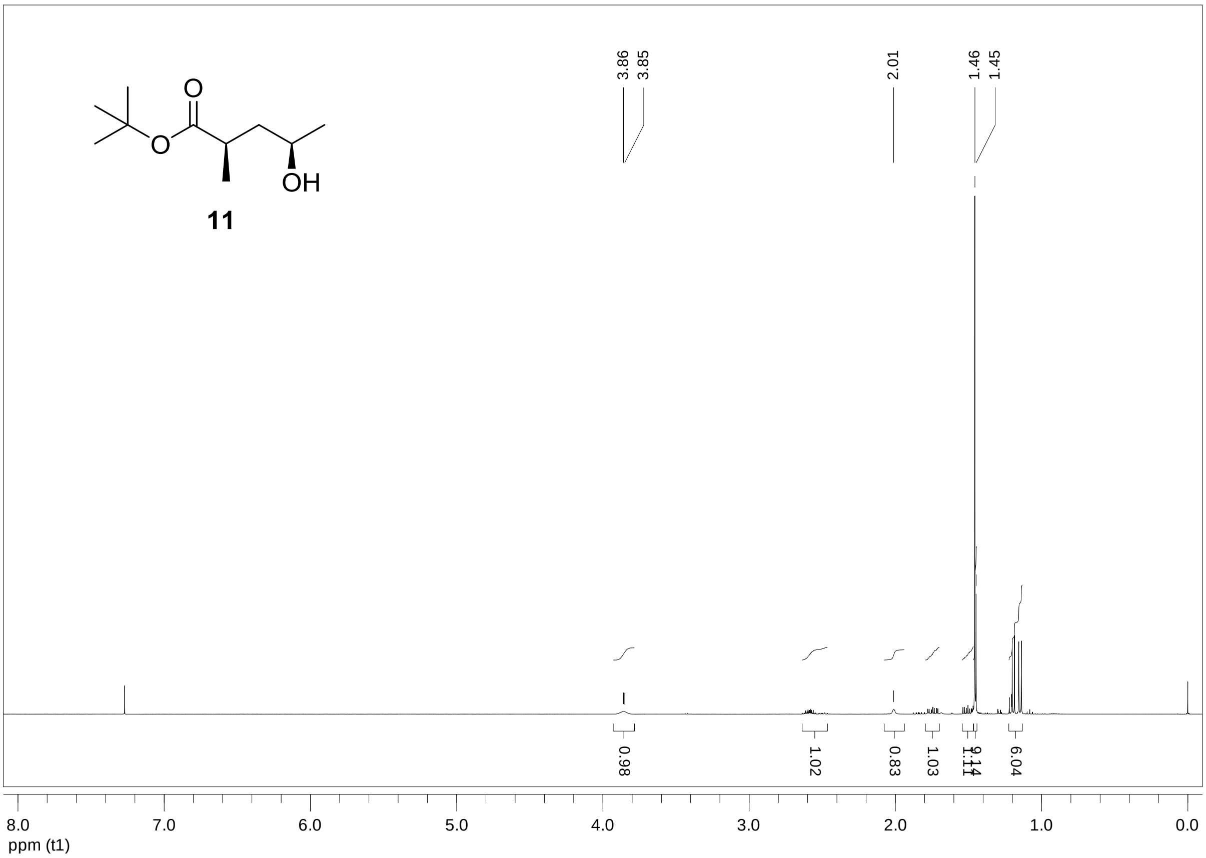

11
3.86
3.85
2.01
1.46
1.45
0.98
1.02
0.83
1.03
6.04
8.0
7.0
6.0
5.0
4.0
3.0
2.0
1.0
0.0
ppm (t1)

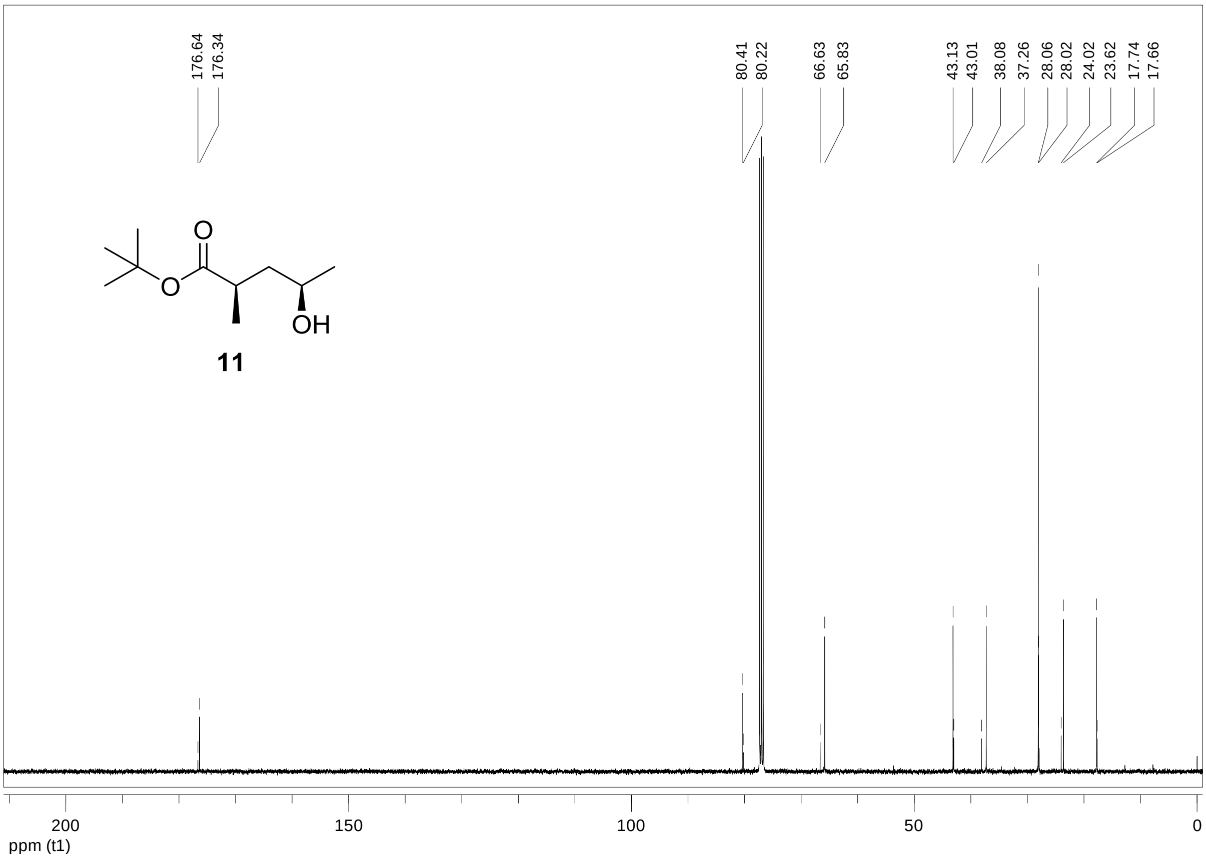

176.64
176.34
80.41
80.22
66.63
65.83
43.13
43.01
38.08
37.26
28.06
28.02
24.02
23.62
17.74
17.66
11
200
150
100
50
0
ppm (t1)

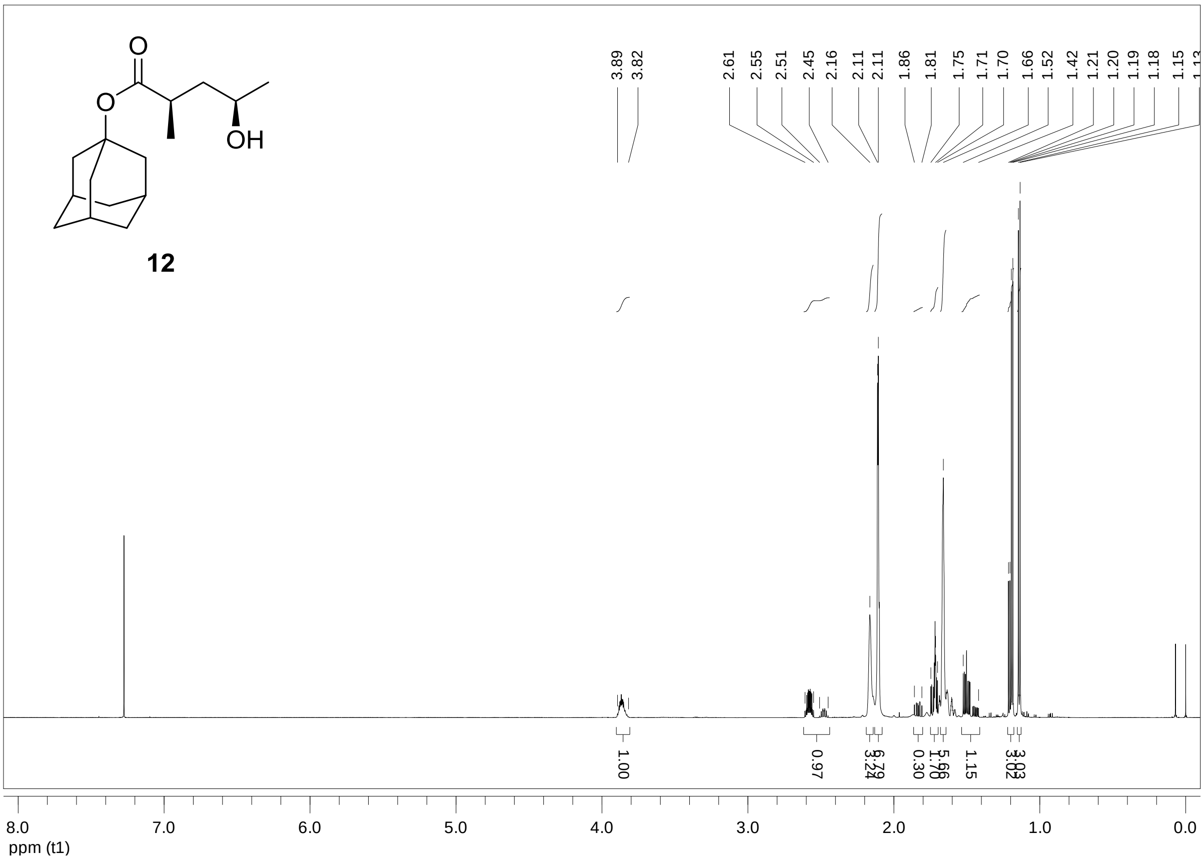
3.89
3.82
2.61
2.55
2.51
2.45
2.16
2.11
2.11
1.86
1.81
1.75
1.71
1.70
1.66
1.52
1.42
1.21
1.20
1.19
1.18
1.15
O
O
OH
12
1.00
0.97
0.30
1.15
8.0
7.0
6.0
5.0
4.0
3.0
2.0
1.0
0.0
ppm (t1)

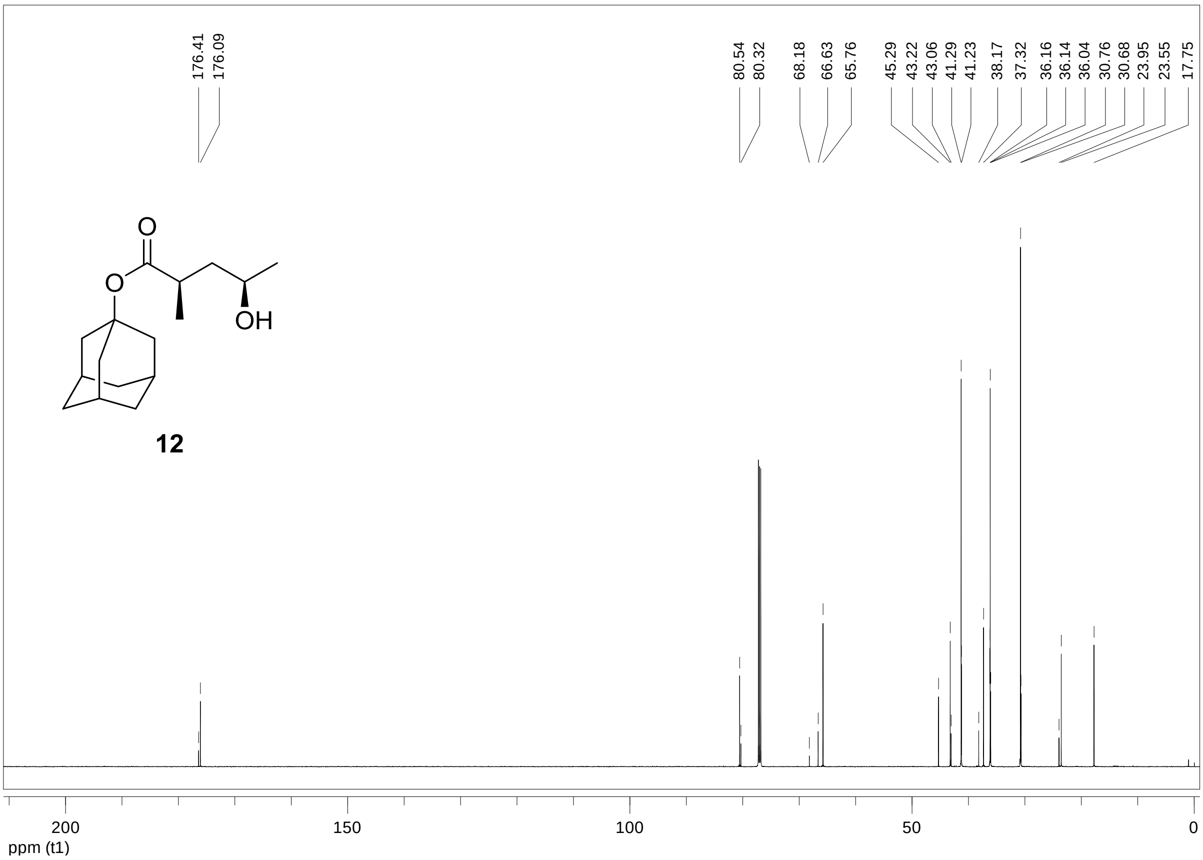
176.41
176.09
80.54
80.32
68.18
66.63
65.76
45.29
43.22
43.06
41.29
41.23
38.17
37.32
36.16
36.14
36.04
30.76
30.68
23.95
23.55
17.75
O
O
OH
12
200
150
100
50
0
ppm (t1)

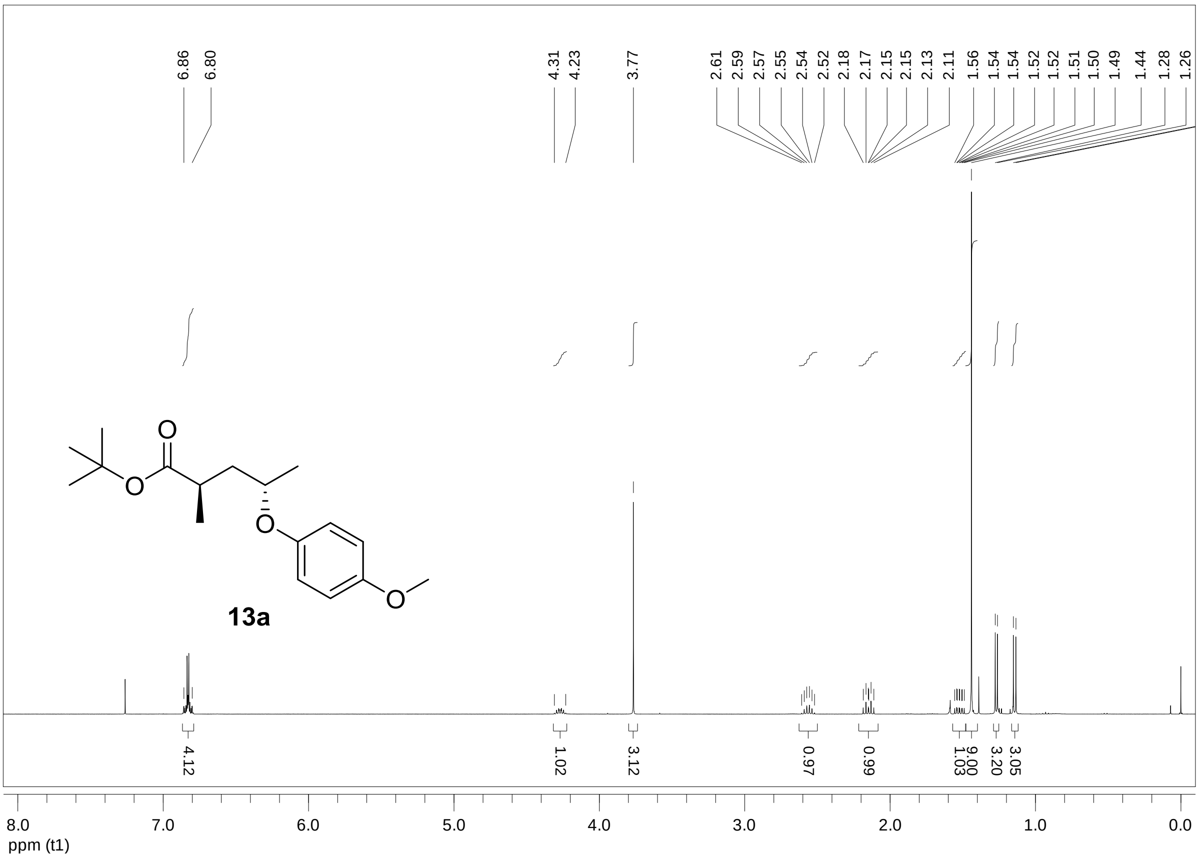
6.86
6.80
4.31
4.23
3.77
2.61
2.59
2.57
2.55
2.54
2.52
2.18
2.17
2.15
2.15
2.13
2.11
1.56
1.54
1.54
1.52
1.52
1.51
1.50
1.49
1.44
1.28
1.26
O
O
O
O
13a
4.12
1.02
3.12
0.97
0.99
1.03
9.00
3.20
3.05
8.0
7.0
6.0
5.0
4.0
3.0
2.0
1.0
0.0
ppm (t1)

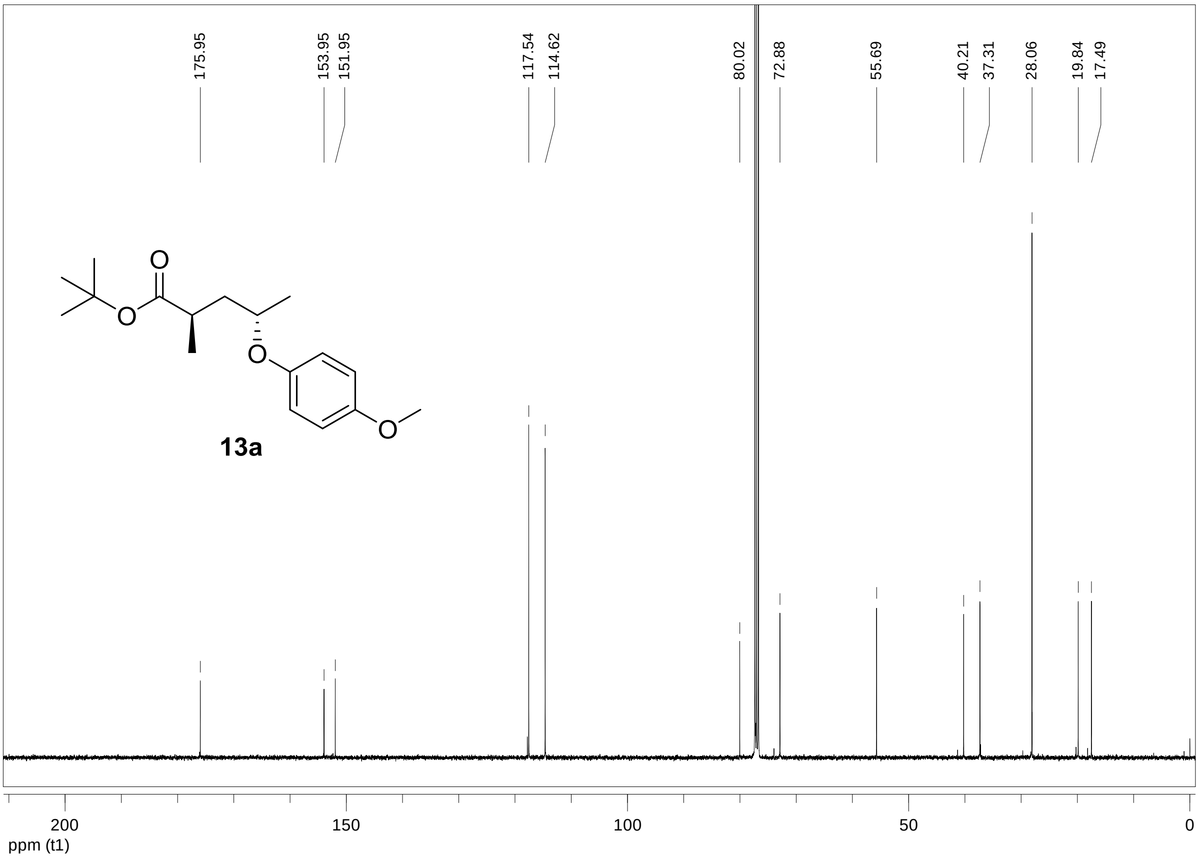
175.95
153.95
151.95
117.54
114.62
80.02
72.88
55.69
40.21
37.31
28.06
19.84
17.49
O
O
O
O
13a
200
150
100
50
0
ppm (t1)

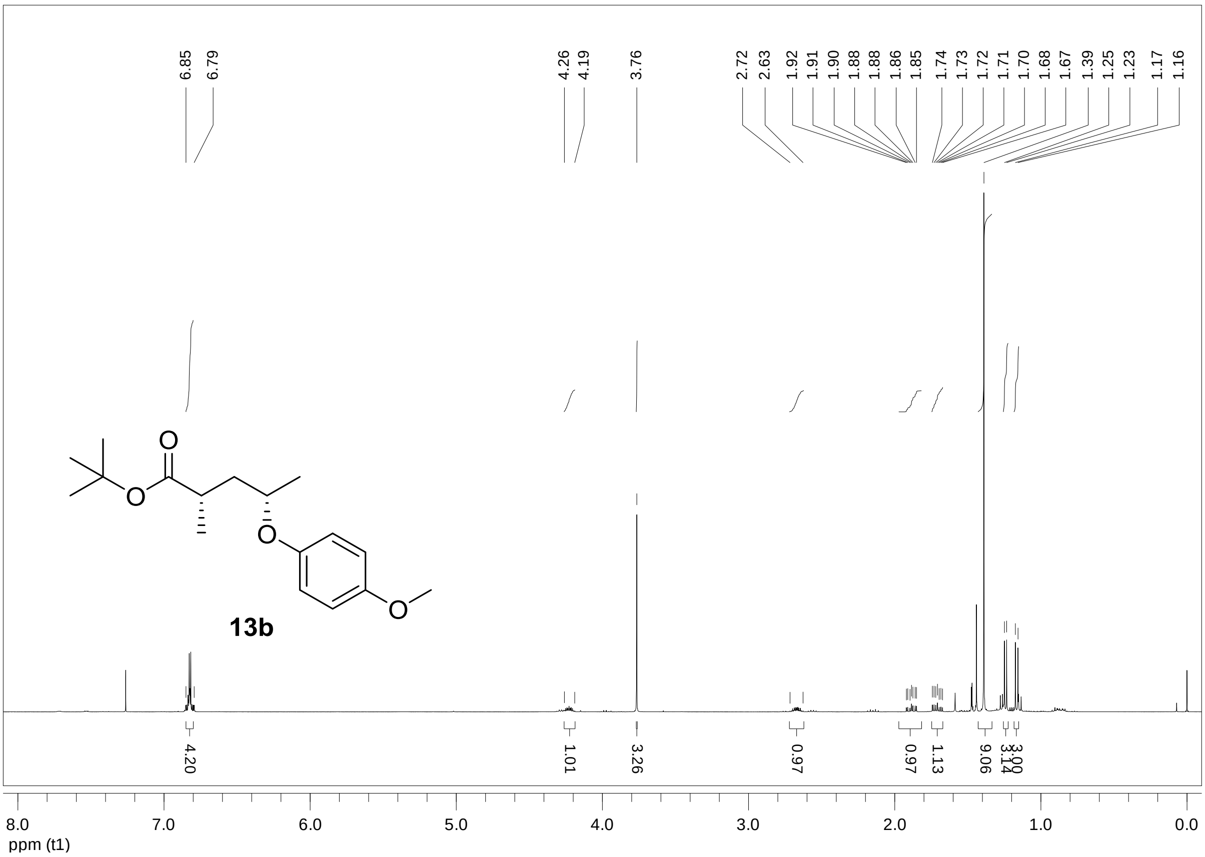
6.85
6.79
4.26
4.19
3.76
2.72
2.63
1.92
1.91
1.90
1.88
1.88
1.86
1.85
1.74
1.73
1.72
1.71
1.70
1.68
1.67
1.39
1.25
1.23
1.17
1.16
13b
4.20
1.01
3.26
0.97
0.97
1.13
9.06
3.14
3.00
8.0
7.0
6.0
5.0
4.0
3.0
2.0
1.0
0.0
ppm (t1)

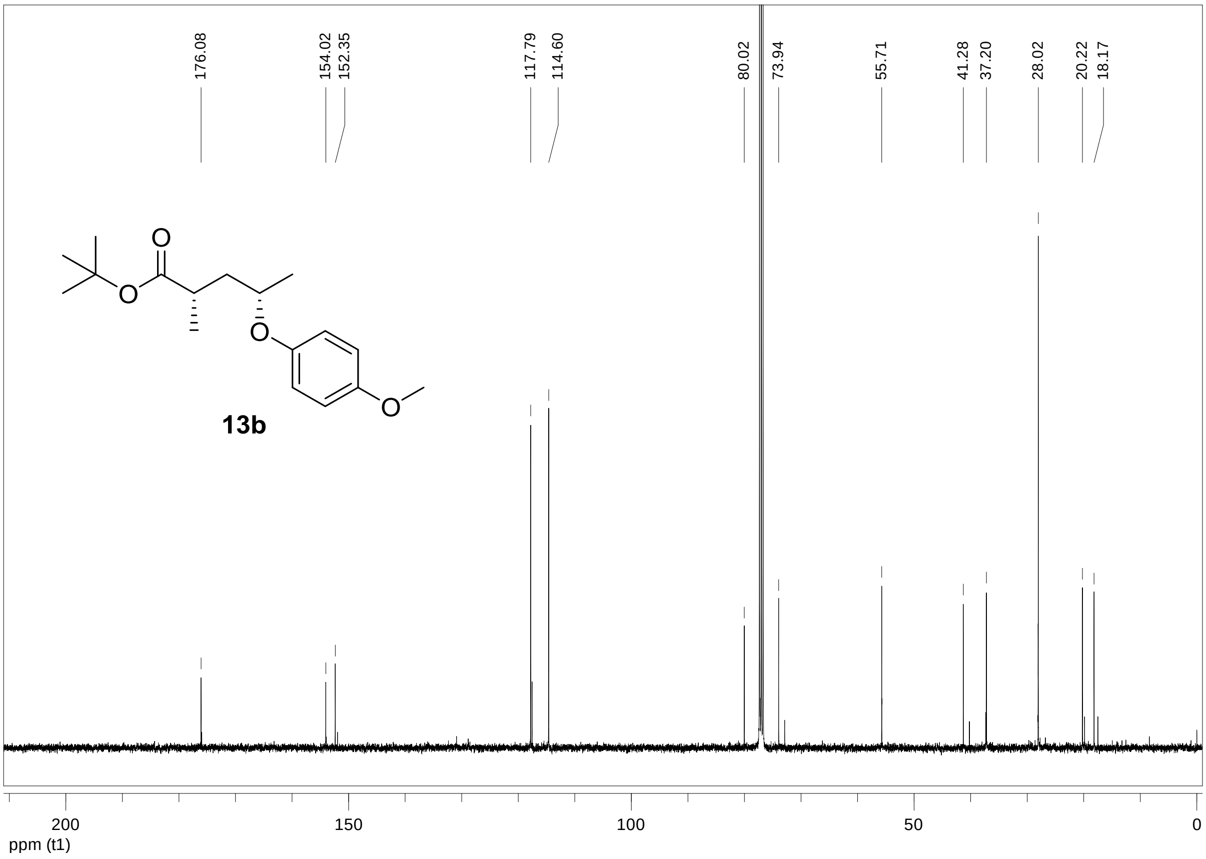
176.08
154.02
152.35
117.79
114.60
80.02
73.94
55.71
41.28
37.20
28.02
20.22
18.17
13b
200
150
100
50
0
ppm (t1)

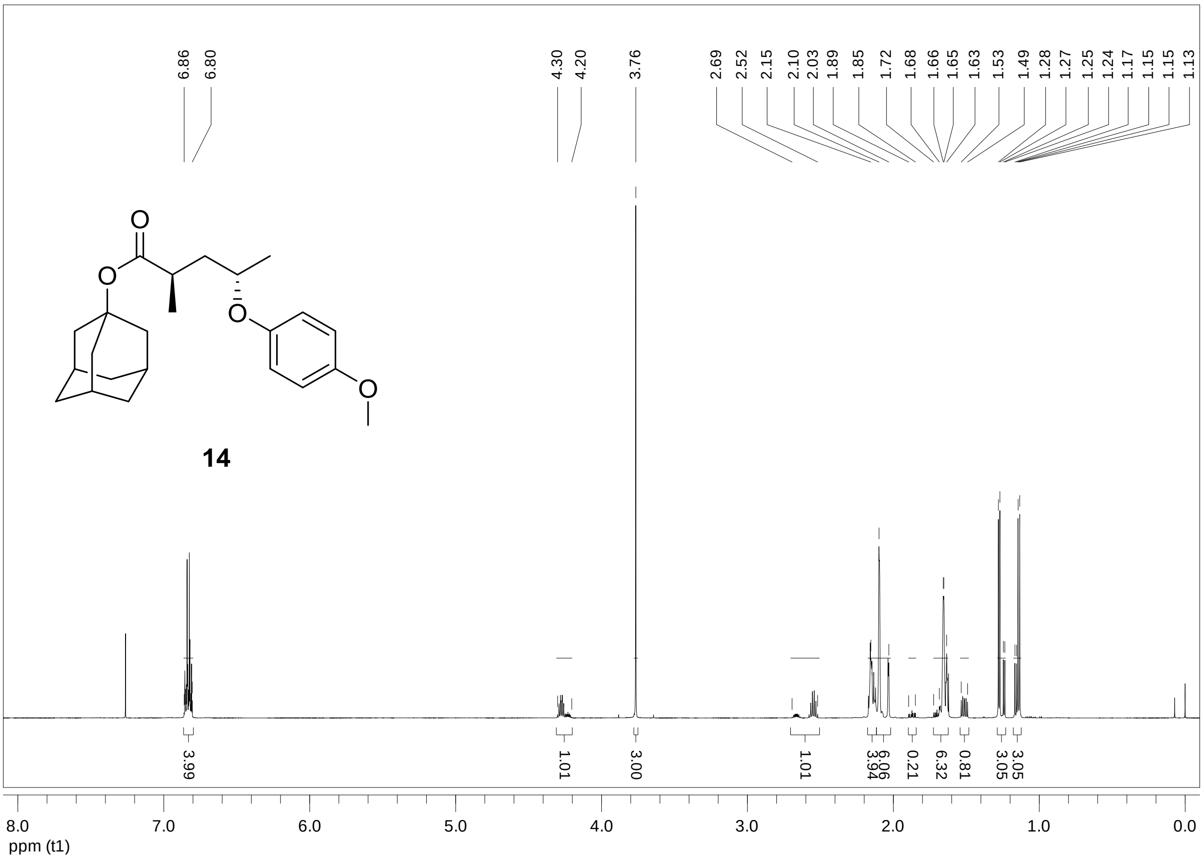
6.86
6.80
4.30
4.20
3.76
2.69
2.52
2.15
2.10
2.03
1.89
1.85
1.72
1.68
1.66
1.65
1.63
1.53
1.49
1.28
1.27
1.25
1.24
1.17
1.15
1.15
1.13
14
3.99
1.01
3.00
1.01
3.94
6.06
0.21
6.32
0.81
3.05
3.05
8.0
7.0
6.0
5.0
4.0
3.0
2.0
1.0
0.0
ppm (t1)

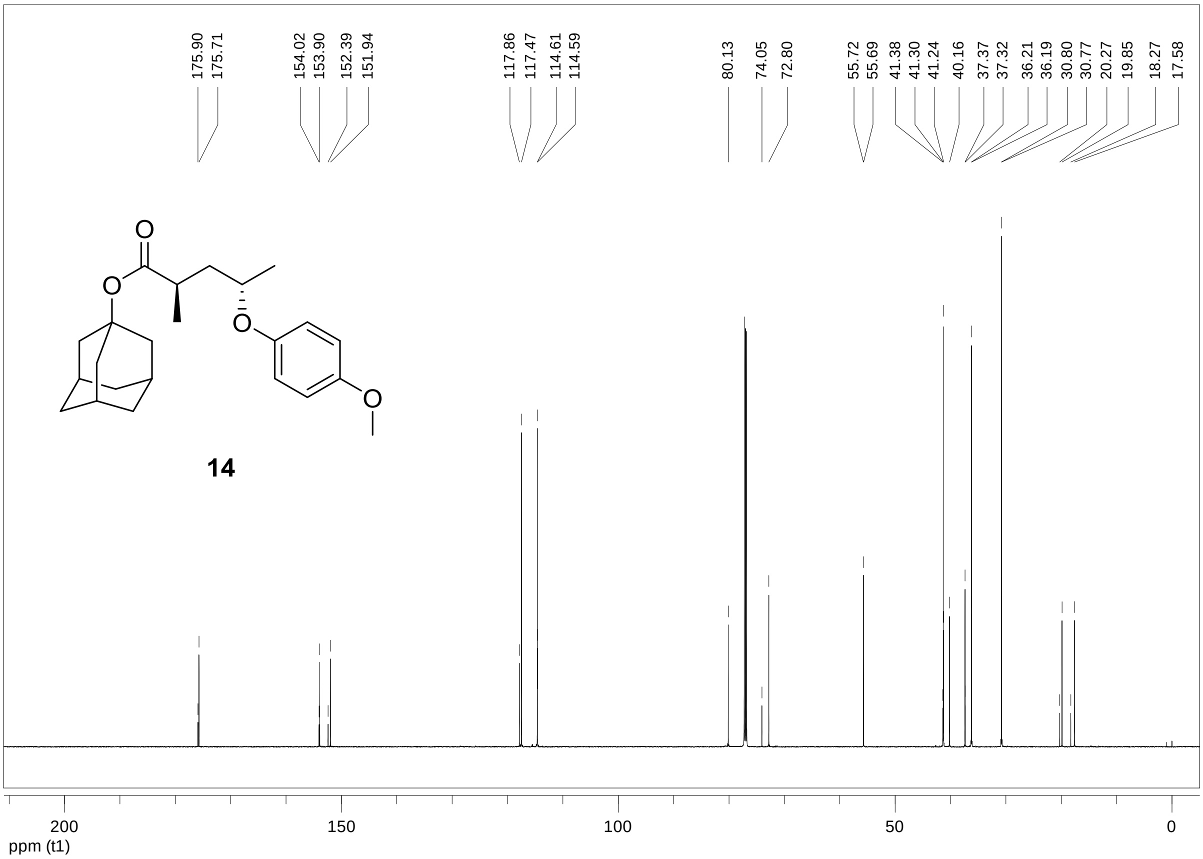
175.90
175.71
154.02
153.90
152.39
151.94
117.86
117.47
114.61
114.59
80.13
74.05
72.80
55.72
55.69
41.38
41.30
41.24
40.16
37.37
37.32
36.21
36.19
30.80
30.77
20.27
19.85
18.27
17.58
14
200
150
100
50
0
ppm (t1)

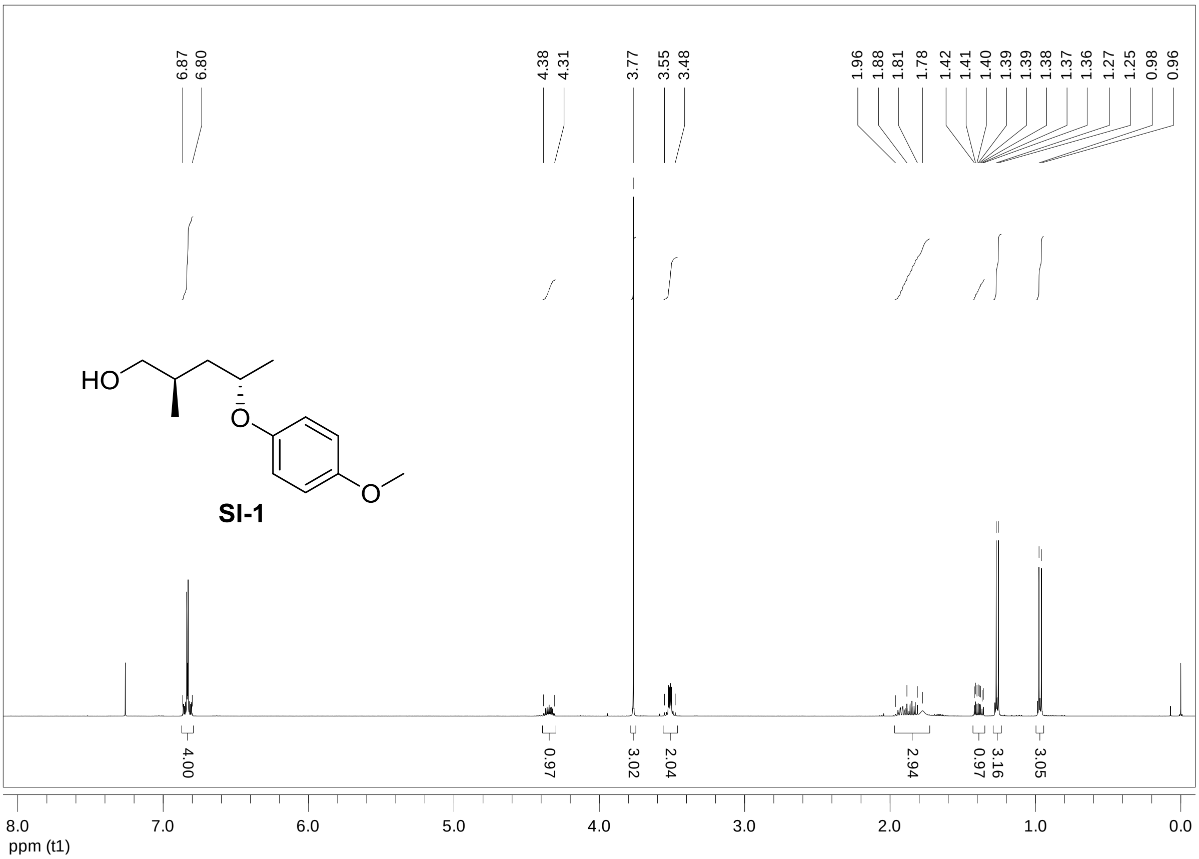
6.87
6.80
4.38
4.31
3.77
3.55
3.48
1.96
1.88
1.81
1.78
1.42
1.41
1.40
1.39
1.39
1.38
1.37
1.36
1.27
1.25
0.98
0.96
HO
O
O
SI-1
4.00
0.97
3.02
2.04
2.94
0.97
3.16
3.05
8.0
7.0
6.0
5.0
4.0
3.0
2.0
1.0
0.0
ppm (t1)

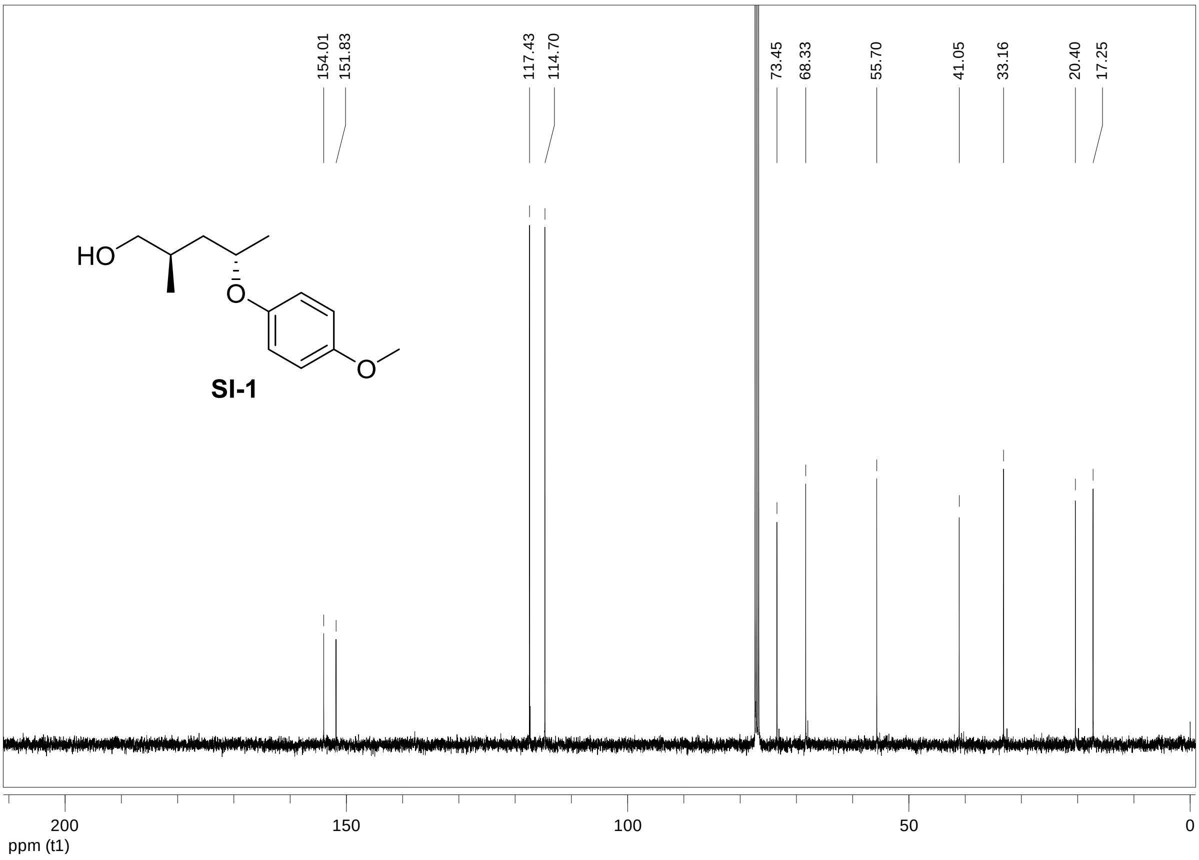
154.01
151.83
117.43
114.70
73.45
68.33
55.70
41.05
33.16
20.40
17.25
HO
O
O
SI-1
200
150
100
50
0
ppm (t1)

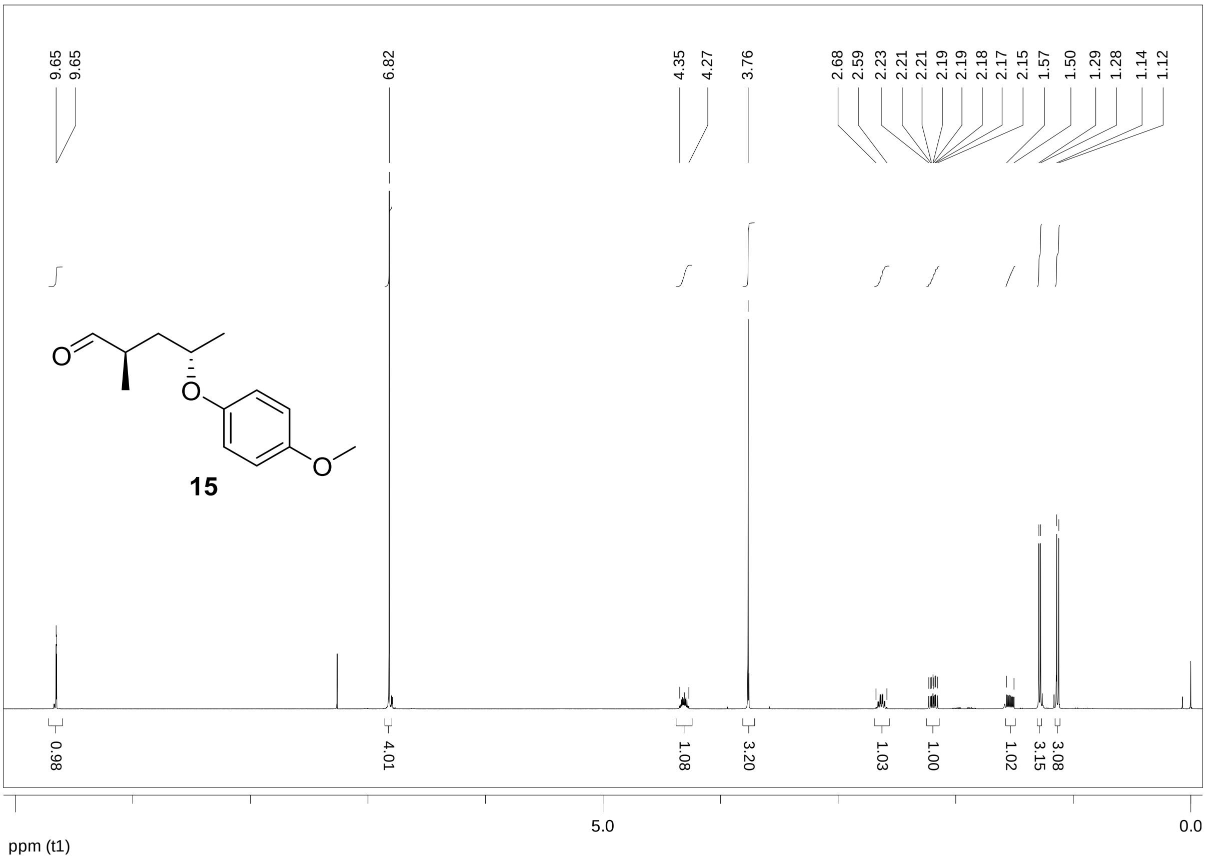
9.65 9.65 6.82 4.35 4.27 3.76 2.68 2.59 2.23 2.21 2.21 2.19 2.19 2.18 2.17 2.15 1.57 1.50 1.29 1.28 1.14 1.12
15
0.98 4.01 1.08 3.20 1.03 1.00 1.02 3.15 3.08
5.0
0.0
ppm (t1)

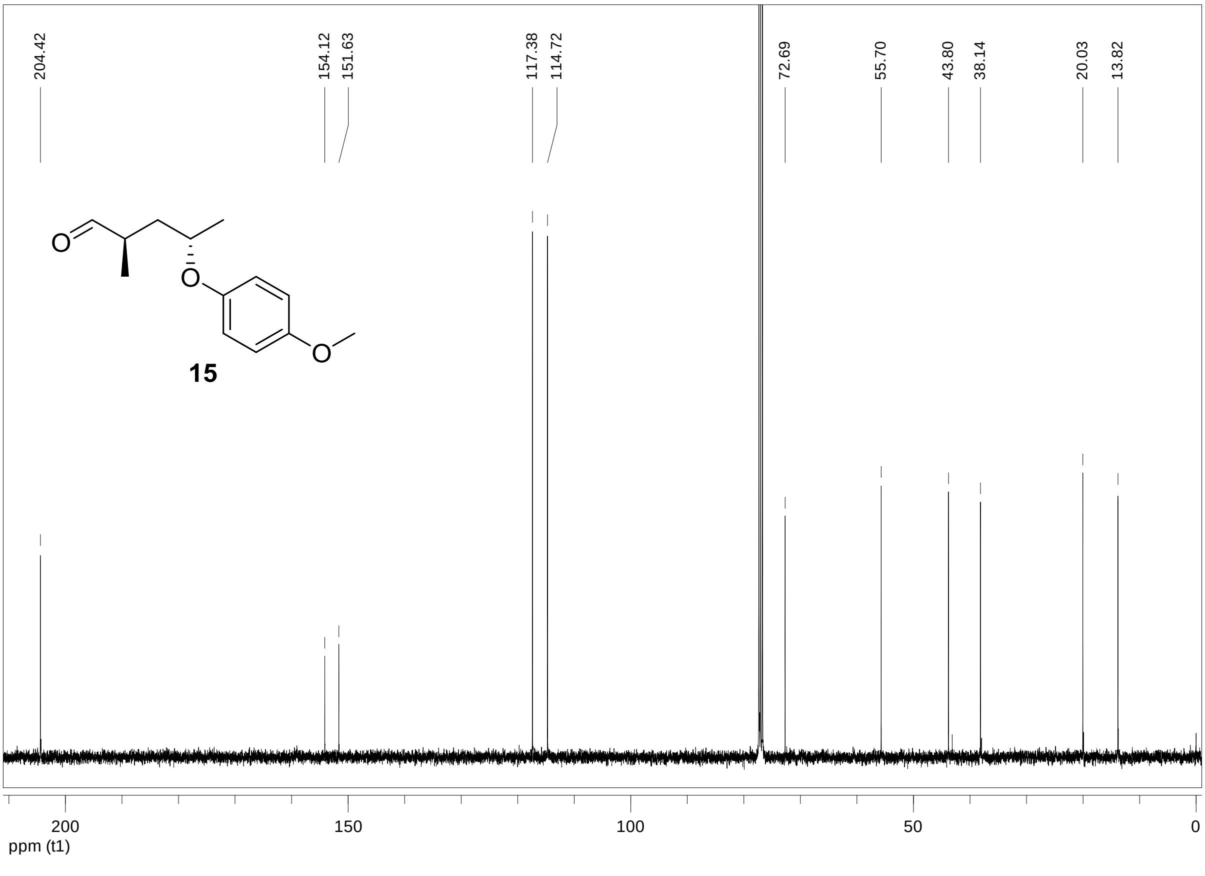
204.42 154.12 151.63 117.38 114.72 72.69 55.70 43.80 38.14 20.03 13.82
15
200 150 100 50 0
ppm (t1)

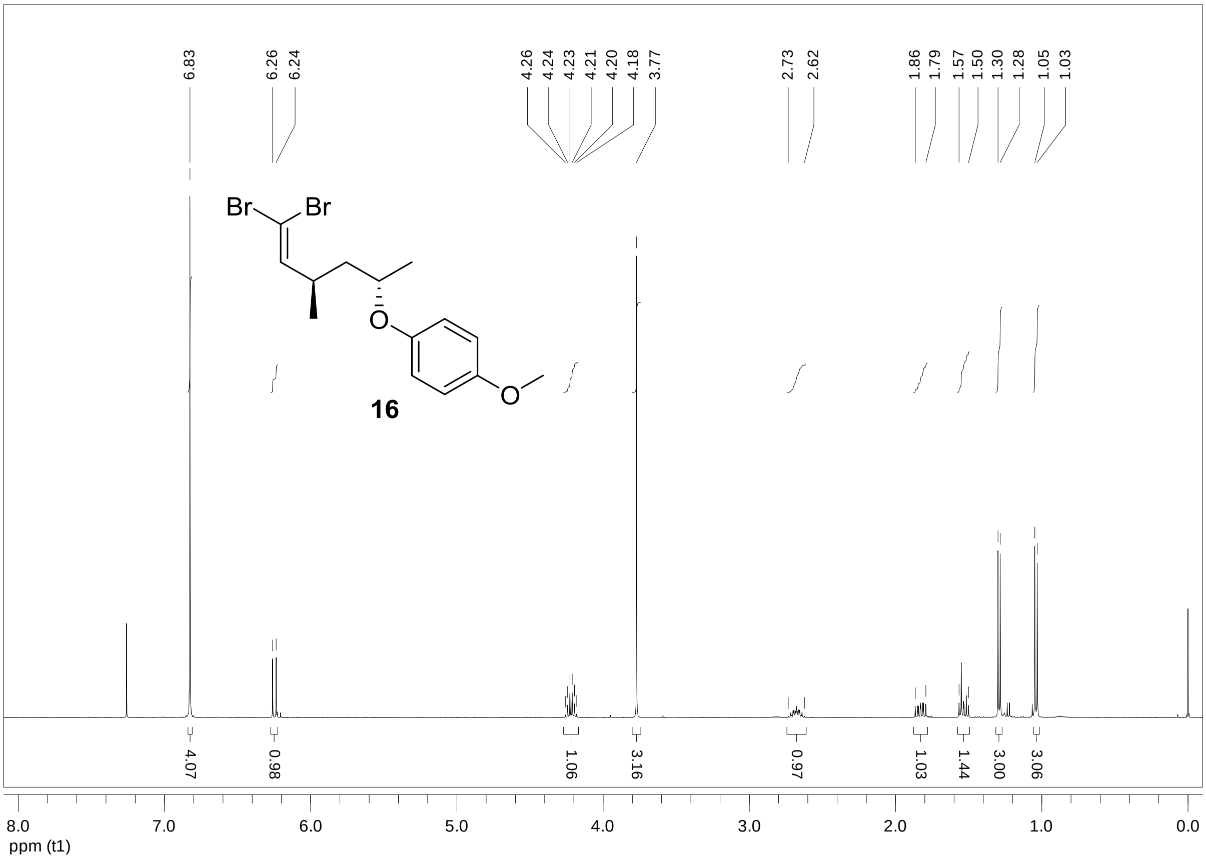
6.83
6.26
6.24
4.26
4.24
4.23
4.21
4.20
4.18
3.77
2.73
2.62
1.86
1.79
1.57
1.50
1.30
1.28
1.05
1.03
Br
Br
O
O
16
4.07
0.98
1.06
3.16
0.97
1.03
1.44
3.00
3.06
8.0
7.0
6.0
5.0
4.0
3.0
2.0
1.0
0.0
ppm (t1)

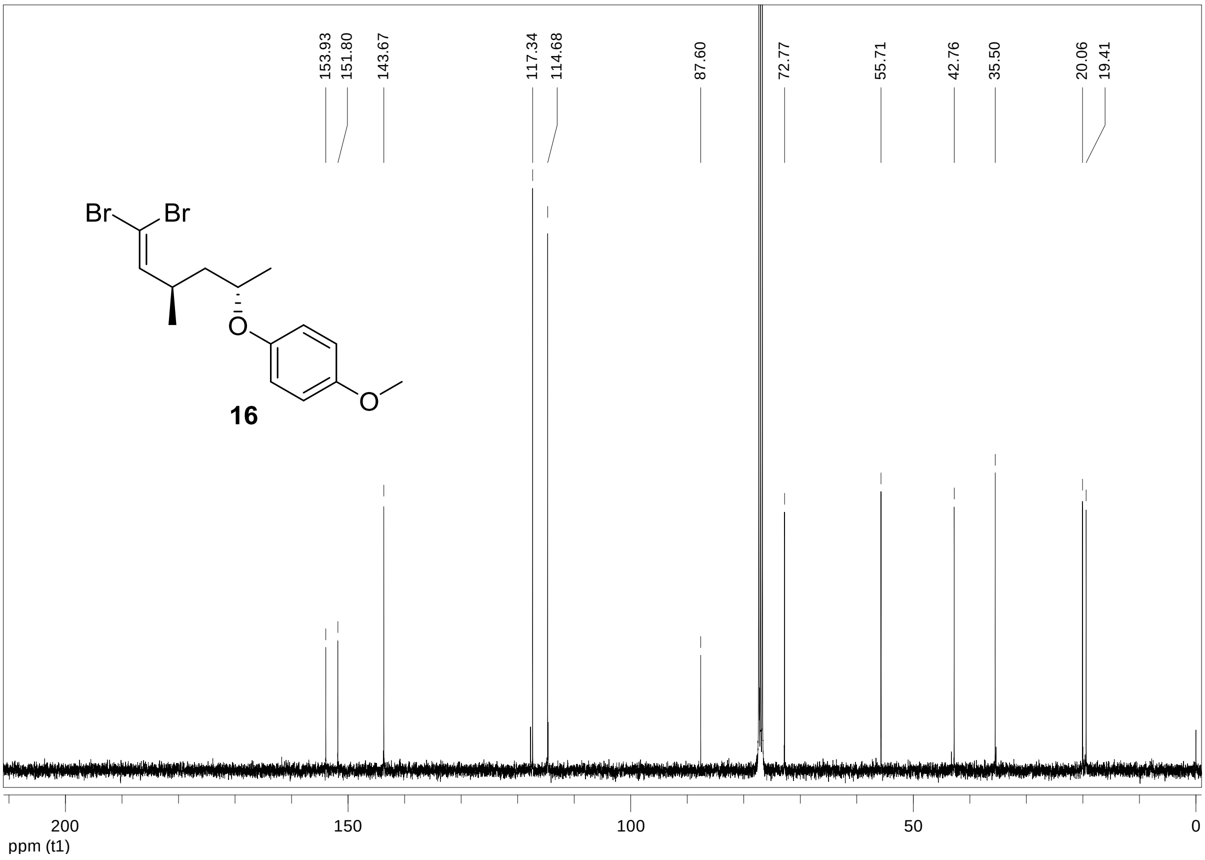
153.93
151.80
143.67
117.34
114.68
87.60
72.77
55.71
42.76
35.50
20.06
19.41
Br
Br
O
O
16
200
150
100
50
0
ppm (t1)

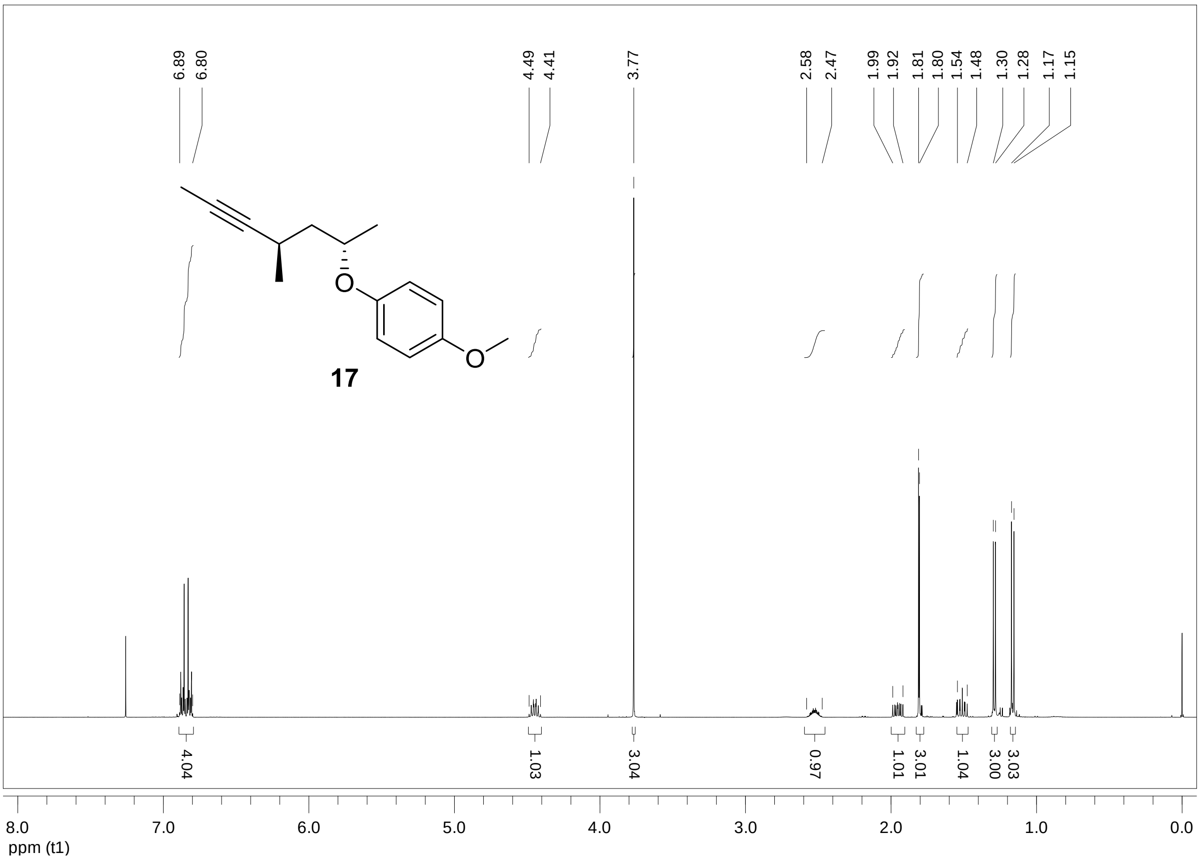
6.89
6.80
4.49
4.41
3.77
2.58
2.47
1.99
1.92
1.81
1.80
1.54
1.48
1.30
1.28
1.17
1.15
17
4.04
1.03
3.04
0.97
1.01
3.01
1.04
3.00
3.03
8.0
7.0
6.0
5.0
4.0
3.0
2.0
1.0
0.0
ppm (t1)

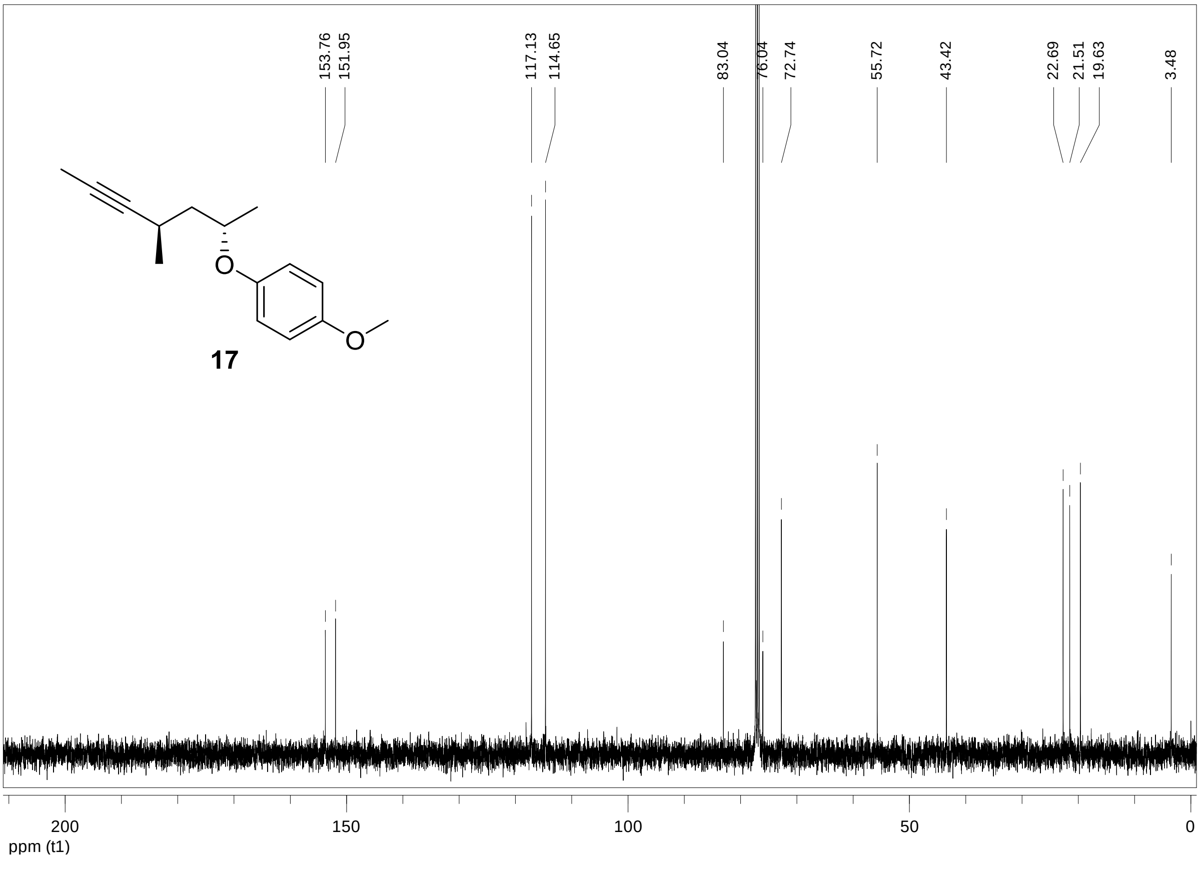
153.76
151.95
117.13
114.65
83.04
76.04
72.74
55.72
43.42
22.69
21.51
19.63
3.48
17
200
150
100
50
0
ppm (t1)

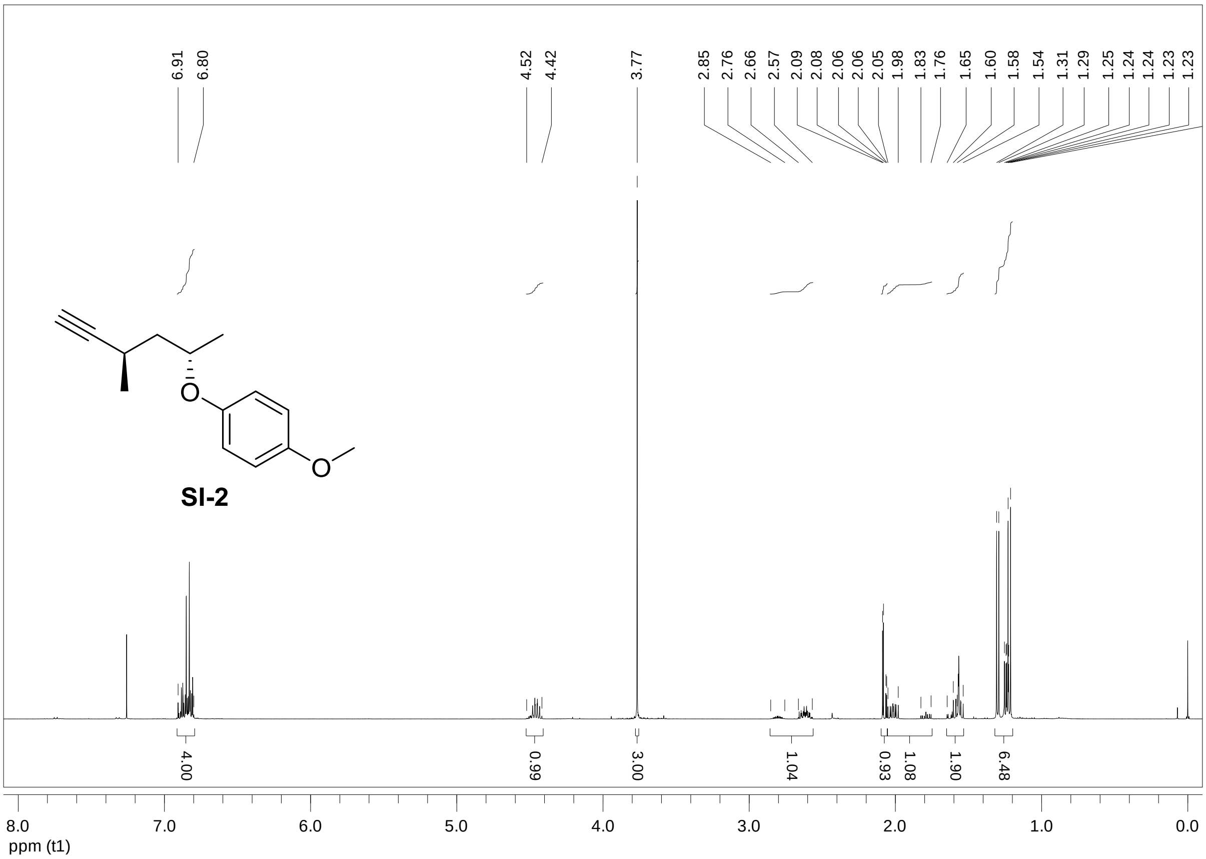
6.91 6.80 4.52 4.42 3.77 2.85 2.76 2.66 2.57 2.09 2.08 2.06 2.06 2.05 1.98 1.83 1.76 1.65 1.60 1.58 1.54 1.31 1.29 1.25 1.24 1.24 1.23 1.23
SI-2
4.00 0.99 3.00 1.04 0.93 1.08 1.90 6.48
8.0 7.0 6.0 5.0 4.0 3.0 2.0 1.0 0.0
ppm (t1)

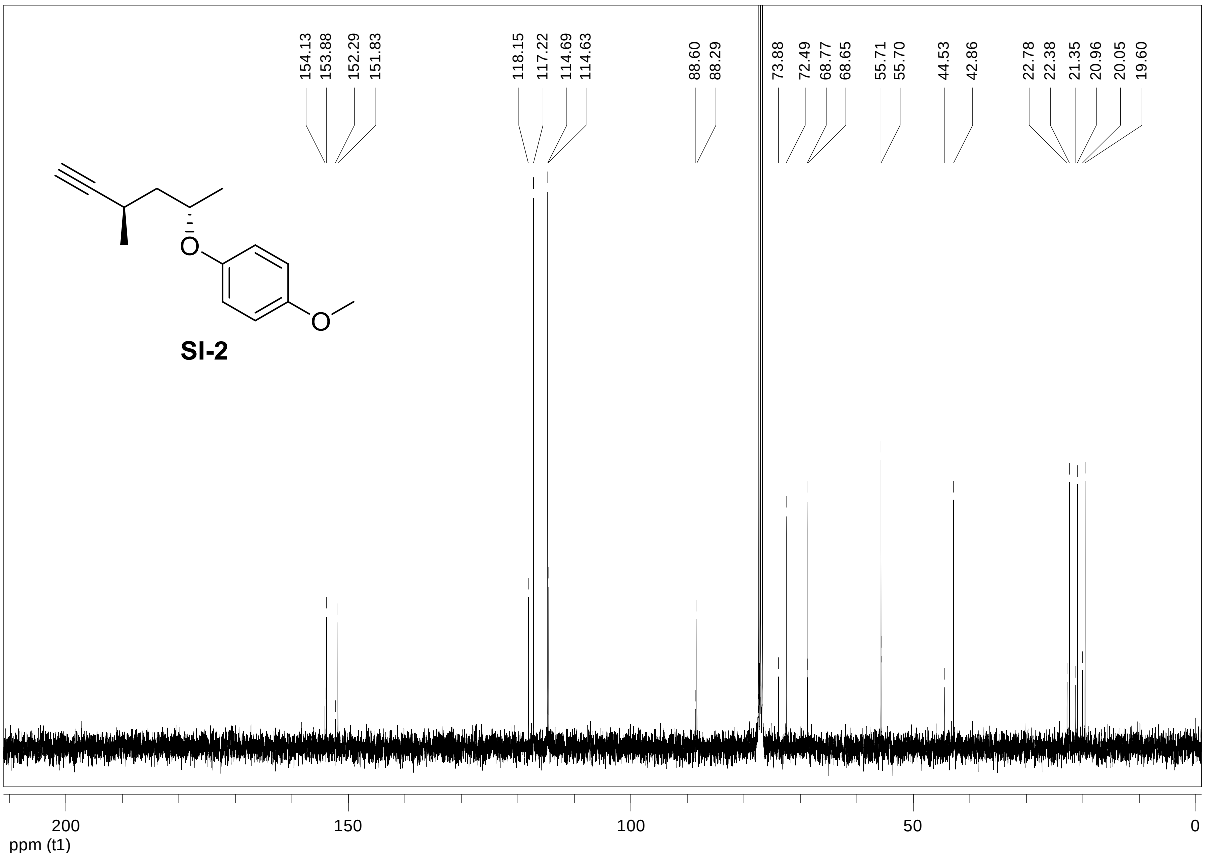
154.13 153.88 152.29 151.83 118.15 117.22 114.69 114.63 88.60 88.29 73.88 72.49 68.77 68.65 55.71 55.70 44.53 42.86 22.78 22.38 21.35 20.96 20.05 19.60
SI-2
200 150 100 50 0
ppm (t1)

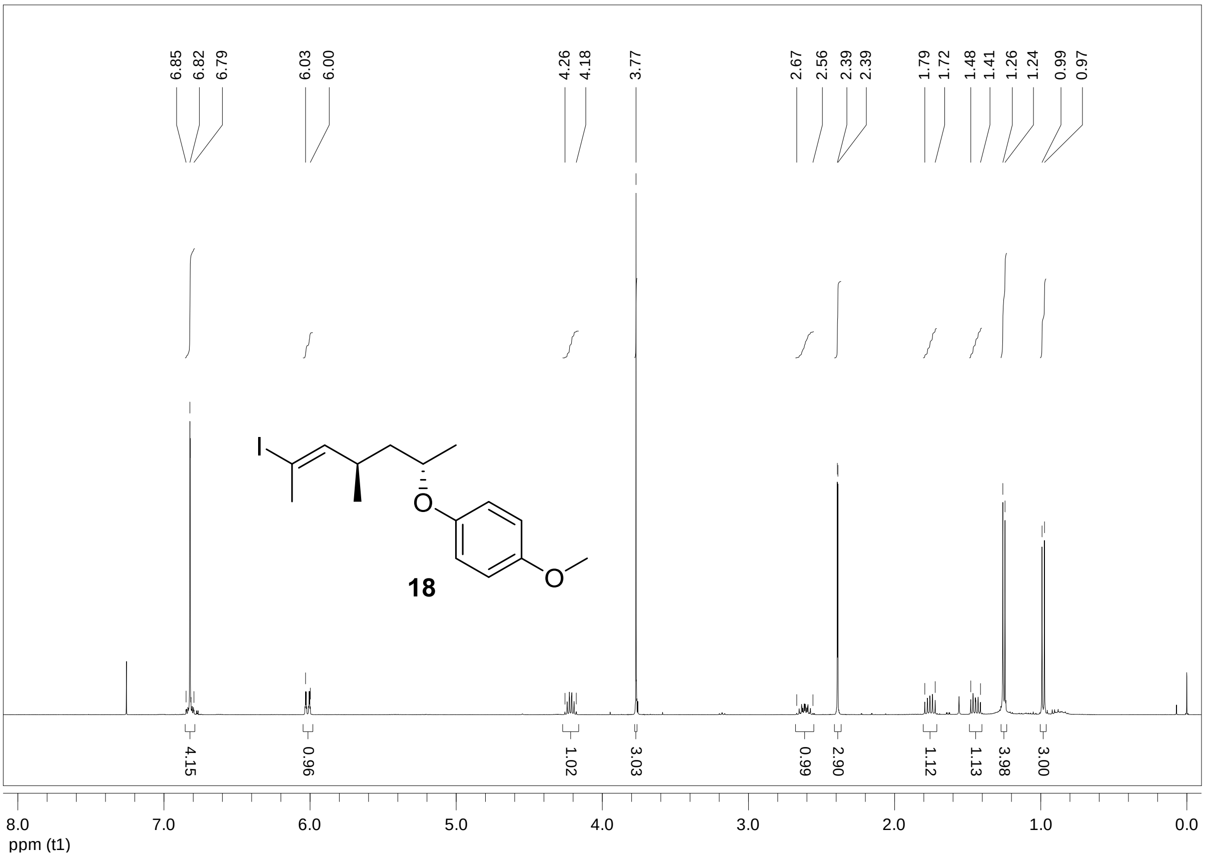
6.85
6.82
6.79
6.03
6.00
4.26
4.18
3.77
2.67
2.56
2.39
2.39
1.79
1.72
1.48
1.41
1.26
1.24
0.99
0.97
I
O
O
18
4.15
0.96
1.02
3.03
0.99
2.90
1.12
1.13
3.98
3.00
8.0
7.0
6.0
5.0
4.0
3.0
2.0
1.0
0.0
ppm (t1)

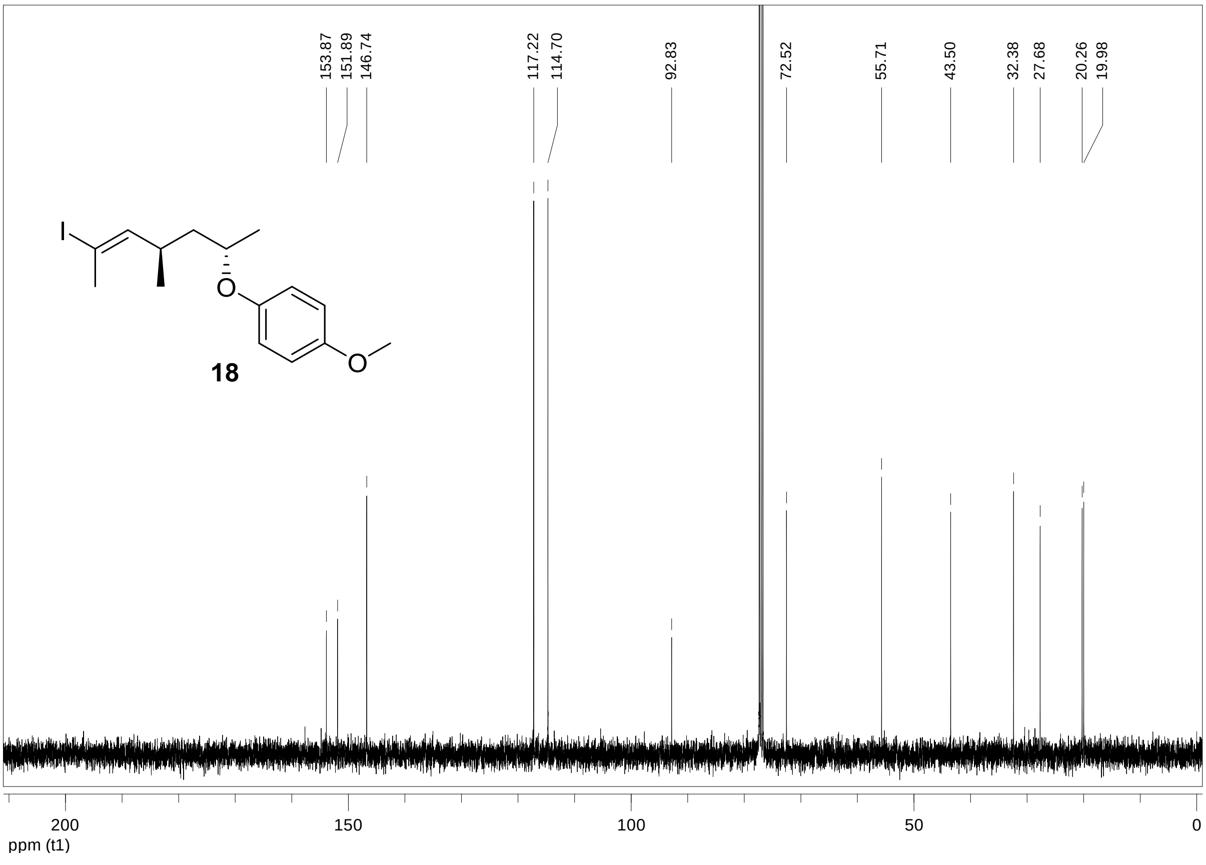
153.87
151.89
146.74
117.22
114.70
92.83
72.52
55.71
43.50
32.38
27.68
20.26
19.98
I
O
O
18
200
150
100
50
0
ppm (t1)

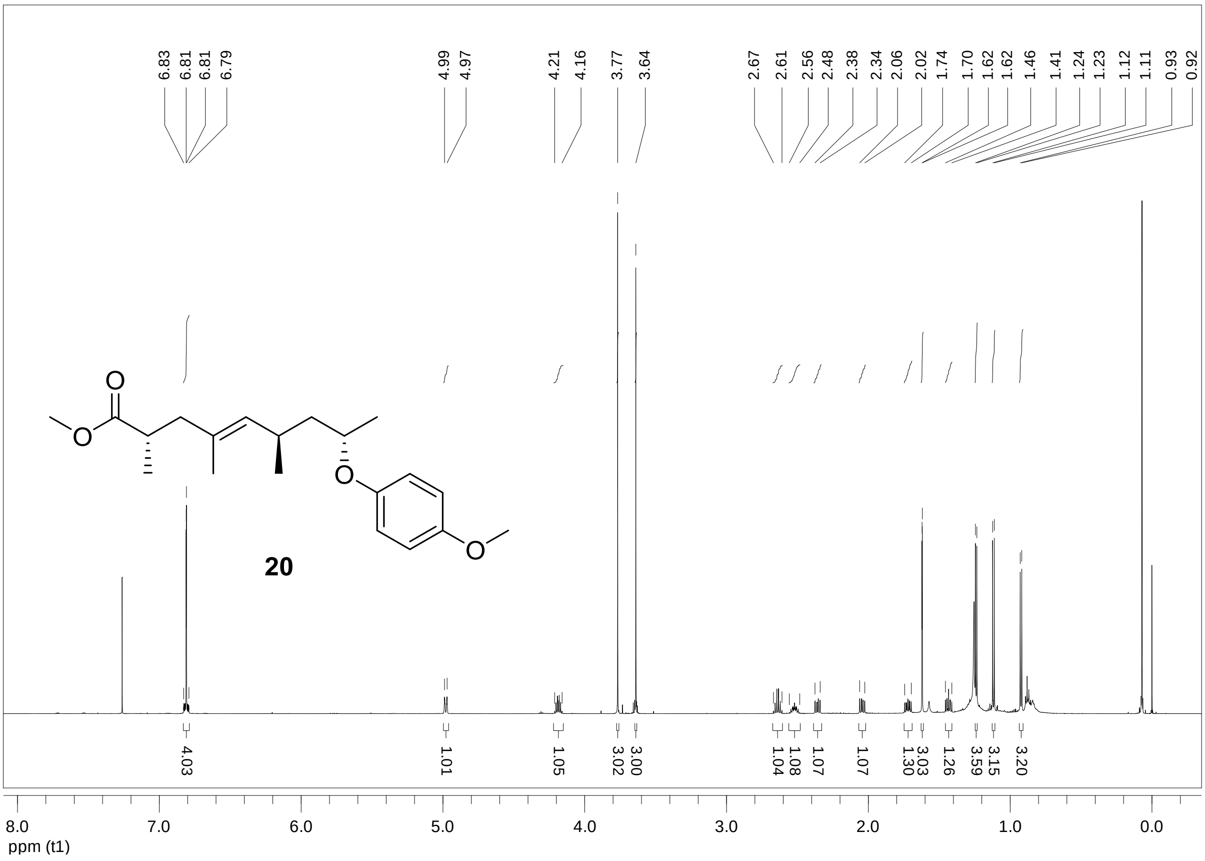
6.83 6.81 6.81 6.79 4.99 4.97 4.21 4.16 3.77 3.64 2.67 2.61 2.56 2.48 2.38 2.34 2.06 2.02 1.74 1.70 1.62 1.62 1.46 1.41 1.24 1.23 1.12 1.11 0.93 0.92
20
4.03 1.01 1.05 3.02 3.00 1.04 1.08 1.07 1.07 1.30 3.03 1.26 3.59 3.15 3.20
8.0 7.0 6.0 5.0 4.0 3.0 2.0 1.0 0.0
ppm (t1)

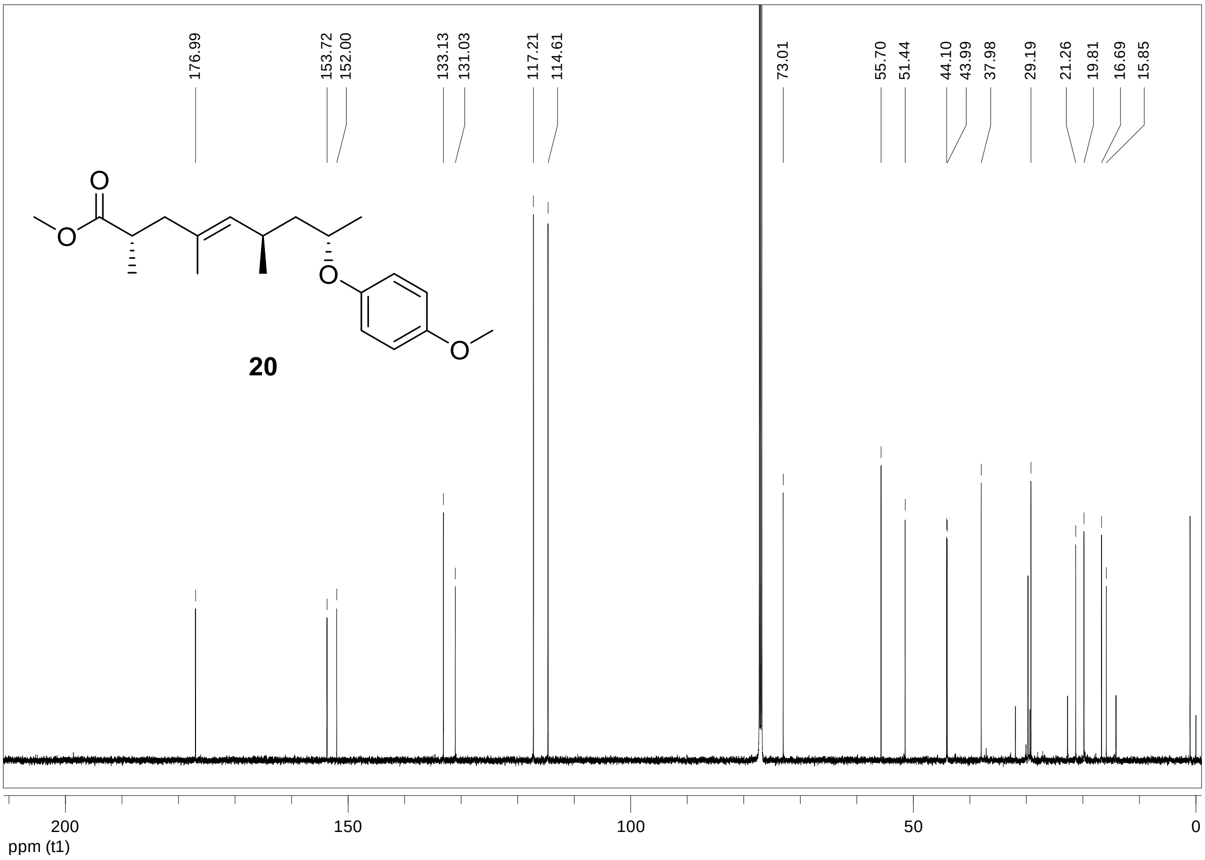
176.99 153.72 152.00 133.13 131.03 117.21 114.61 73.01 55.70 51.44 44.10 43.99 37.98 29.19 21.26 19.81 16.69 15.85
20
200 150 100 50 0
ppm (t1)

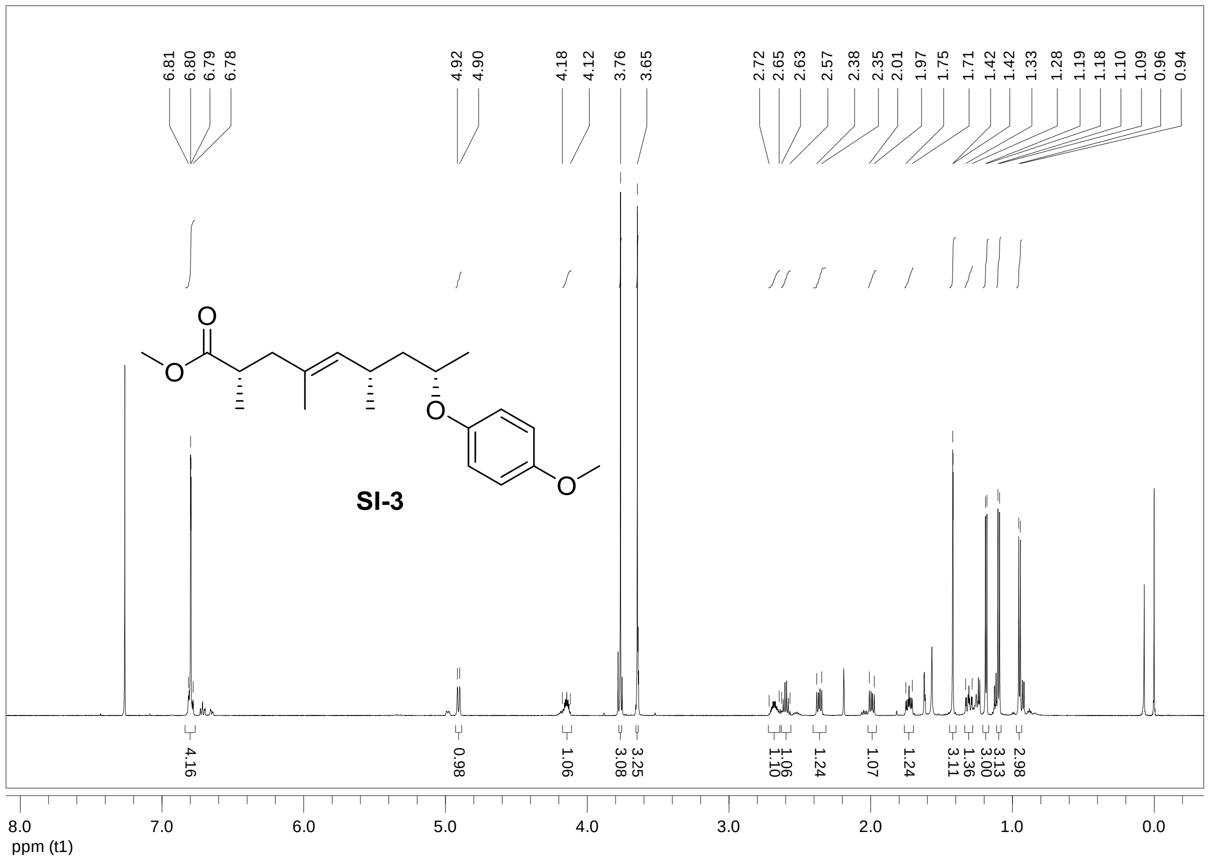
6.81
6.80
6.79
6.78
4.92
4.90
4.18
4.12
3.76
3.65
2.72
2.65
2.63
2.57
2.38
2.35
2.01
1.97
1.75
1.71
1.42
1.42
1.33
1.28
1.19
1.18
1.10
1.09
0.96
0.94
SI-3
4.16
0.98
1.06
3.08
3.25
1.10
1.06
1.24
1.07
1.24
3.11
1.36
3.00
3.13
2.98
ppm (t1)

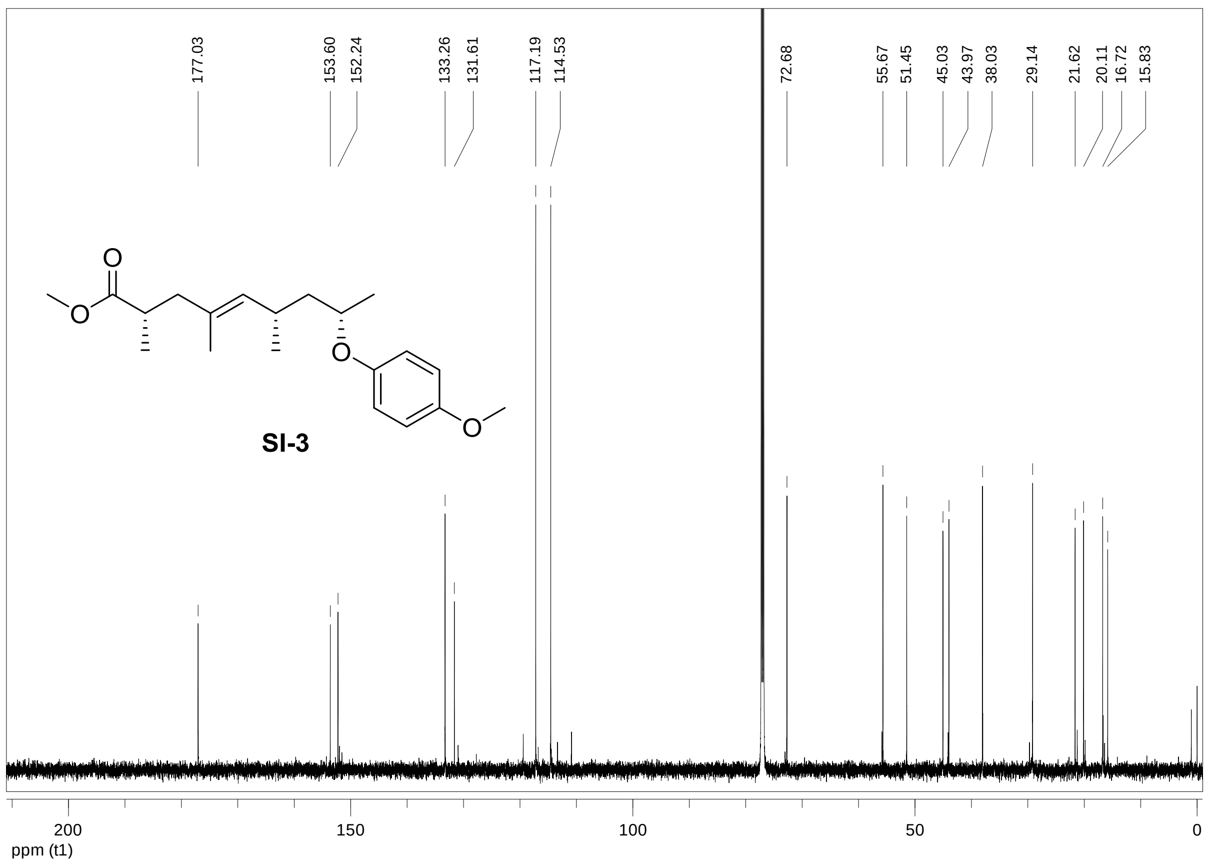
177.03
153.60
152.24
133.26
131.61
117.19
114.53
72.68
55.67
51.45
45.03
43.97
38.03
29.14
21.62
20.11
16.72
15.83
SI-3
ppm (t1)

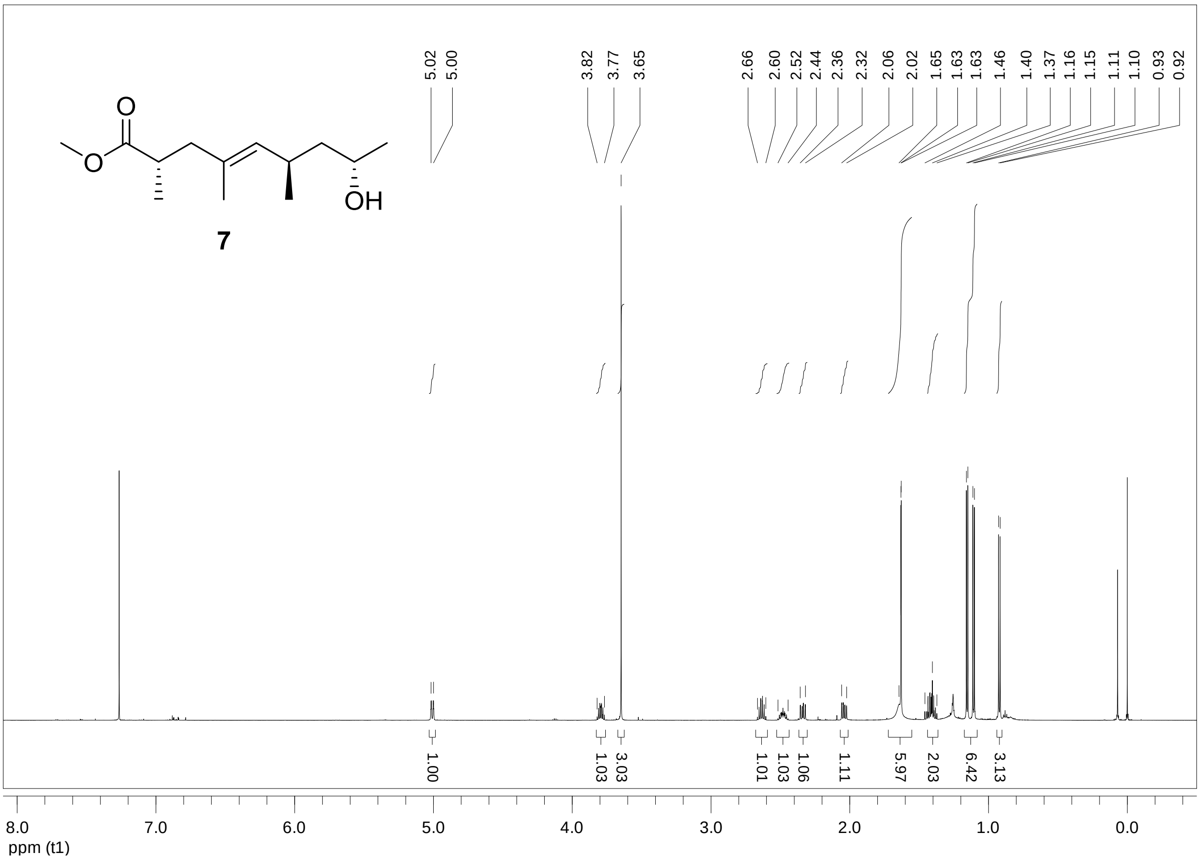
5.02
5.00
3.82
3.77
3.65
2.66
2.60
2.52
2.44
2.36
2.32
2.06
2.02
1.65
1.63
1.63
1.46
1.40
1.37
1.16
1.15
1.11
1.10
0.93
0.92
O
O
OH
7
1.00
1.03
3.03
1.01
1.03
1.06
1.11
5.97
2.03
6.42
3.13
8.0
7.0
6.0
5.0
4.0
3.0
2.0
1.0
0.0
ppm (t1)

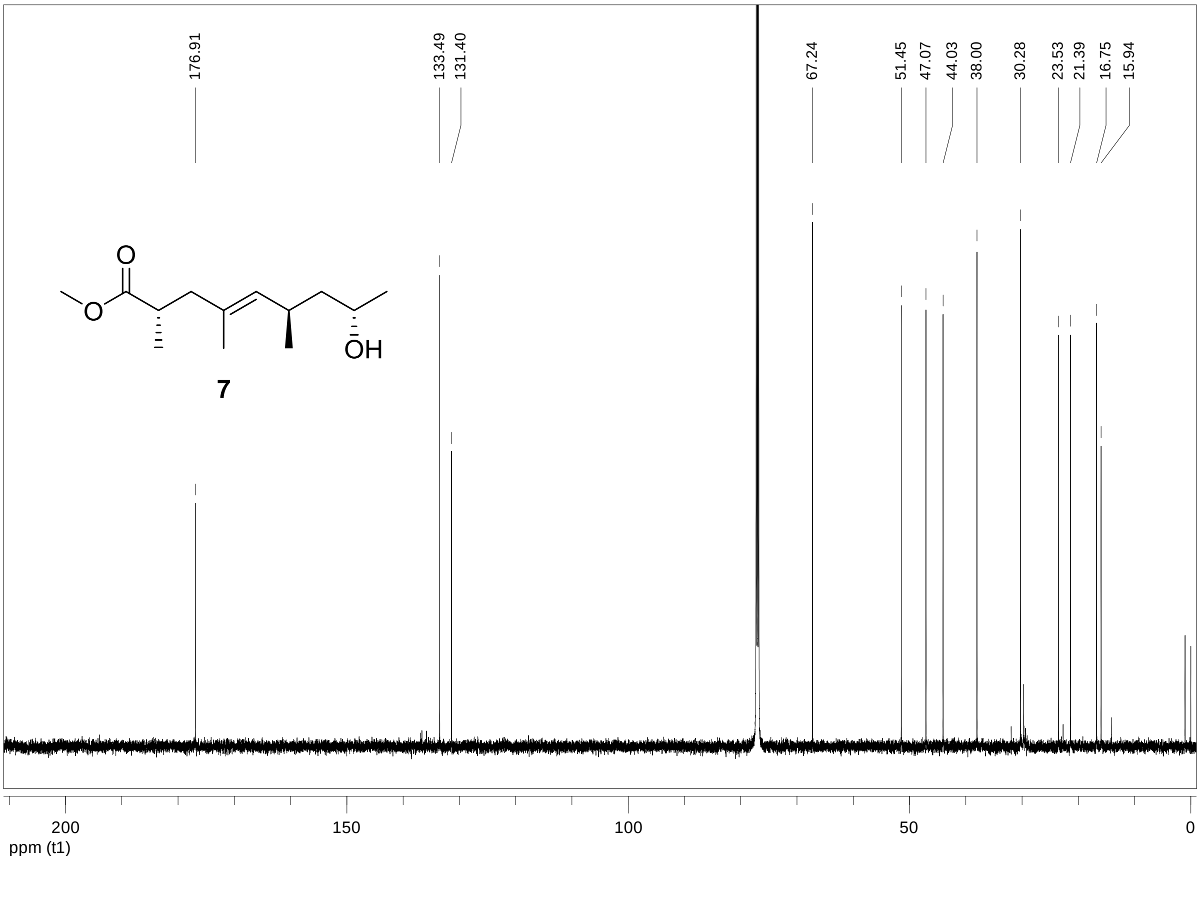
176.91
133.49
131.40
67.24
51.45
47.07
44.03
38.00
30.28
23.53
21.39
16.75
15.94
O
O
OH
7
200
150
100
50
0
ppm (t1)

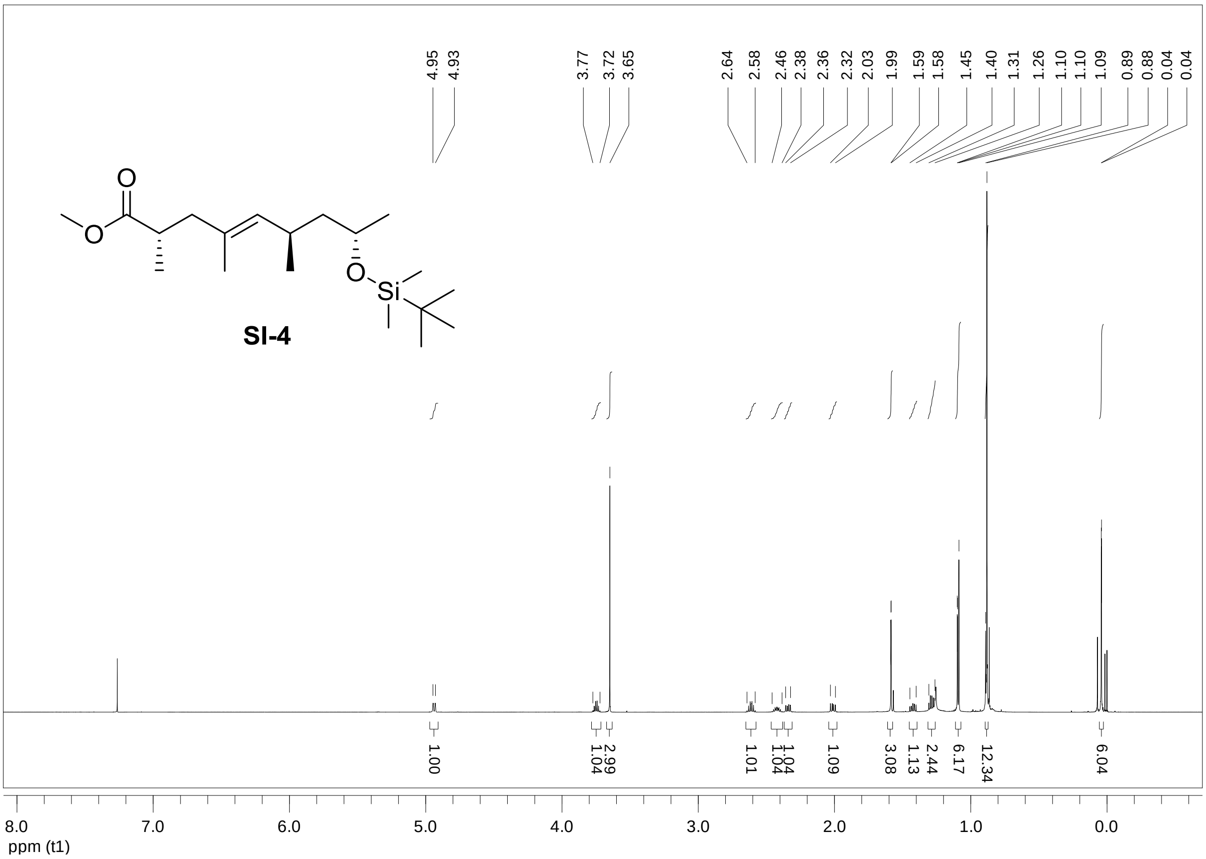
SI-4
ppm (t1)

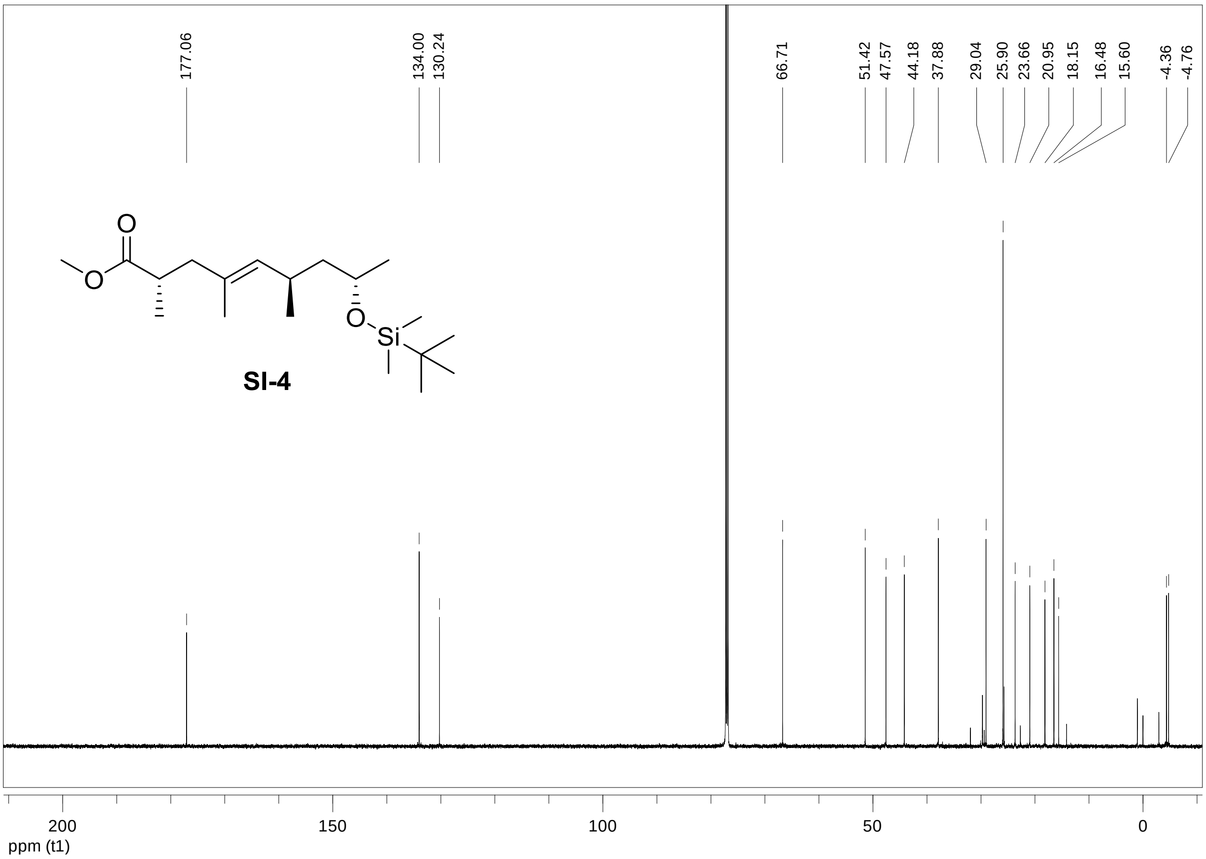
SI-4
ppm (t1)

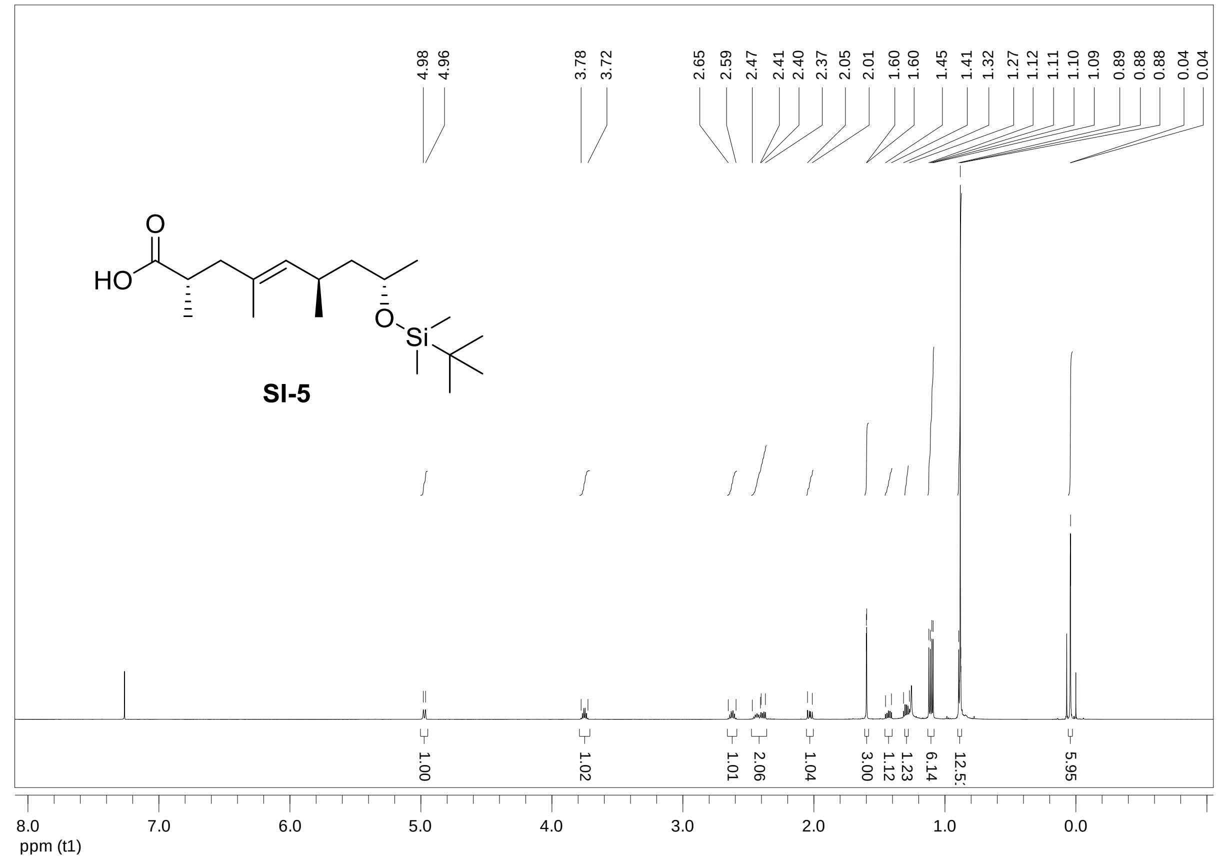
4.98 4.96 3.78 3.72 2.65 2.59 2.47 2.41 2.40 2.37 2.05 2.01 1.60 1.60 1.45 1.41 1.32 1.27 1.12 1.11 1.10 1.09 0.89 0.88 0.88 0.04 0.04
HO
O
O
Si
SI-5
1.00 1.02 1.01 2.06 1.04 3.00 1.12 1.23 6.14 12.5 5.95
8.0 7.0 6.0 5.0 4.0 3.0 2.0 1.0 0.0
ppm (t1)

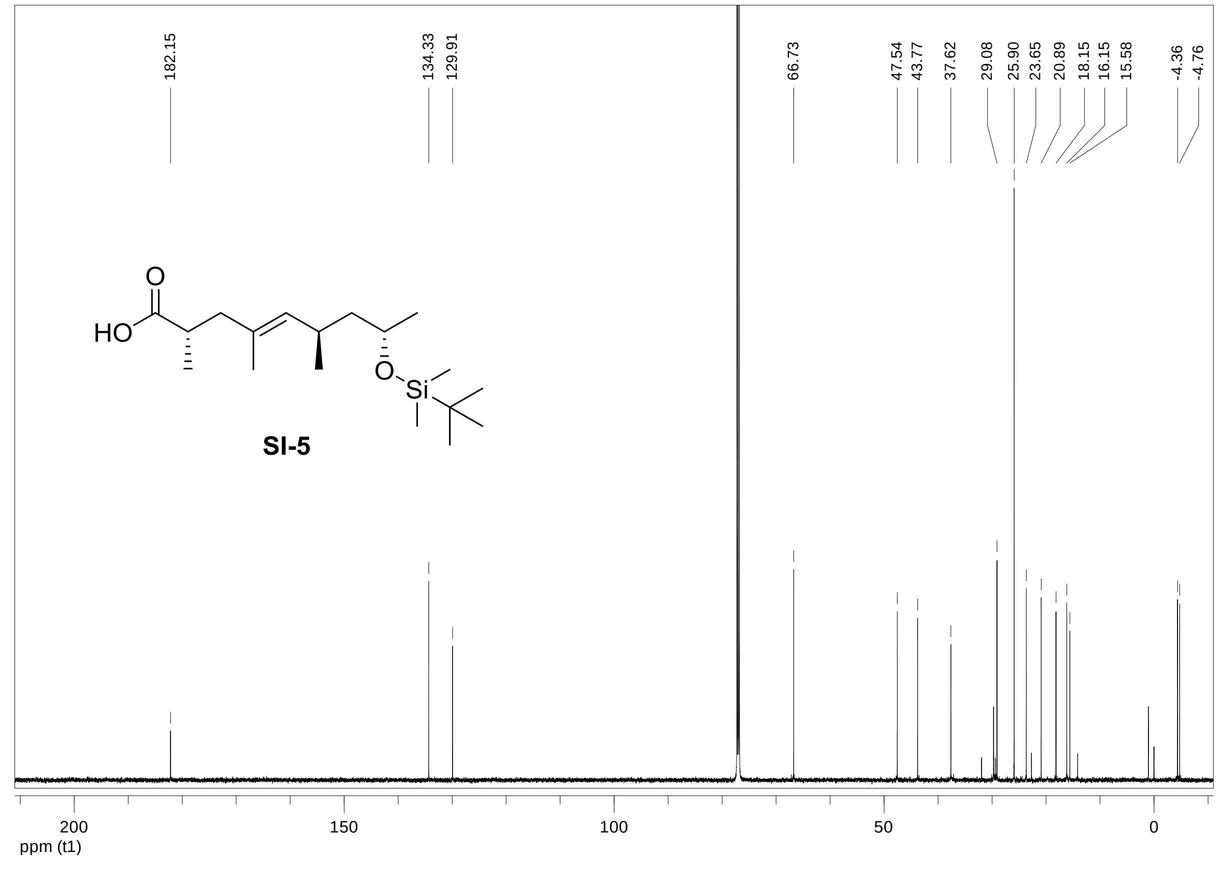
182.15 134.33 129.91 66.73 47.54 43.77 37.62 29.08 25.90 23.65 20.89 18.15 16.15 15.58 -4.36 -4.76
HO
O
O
Si
SI-5
200 150 100 50 0
ppm (t1)

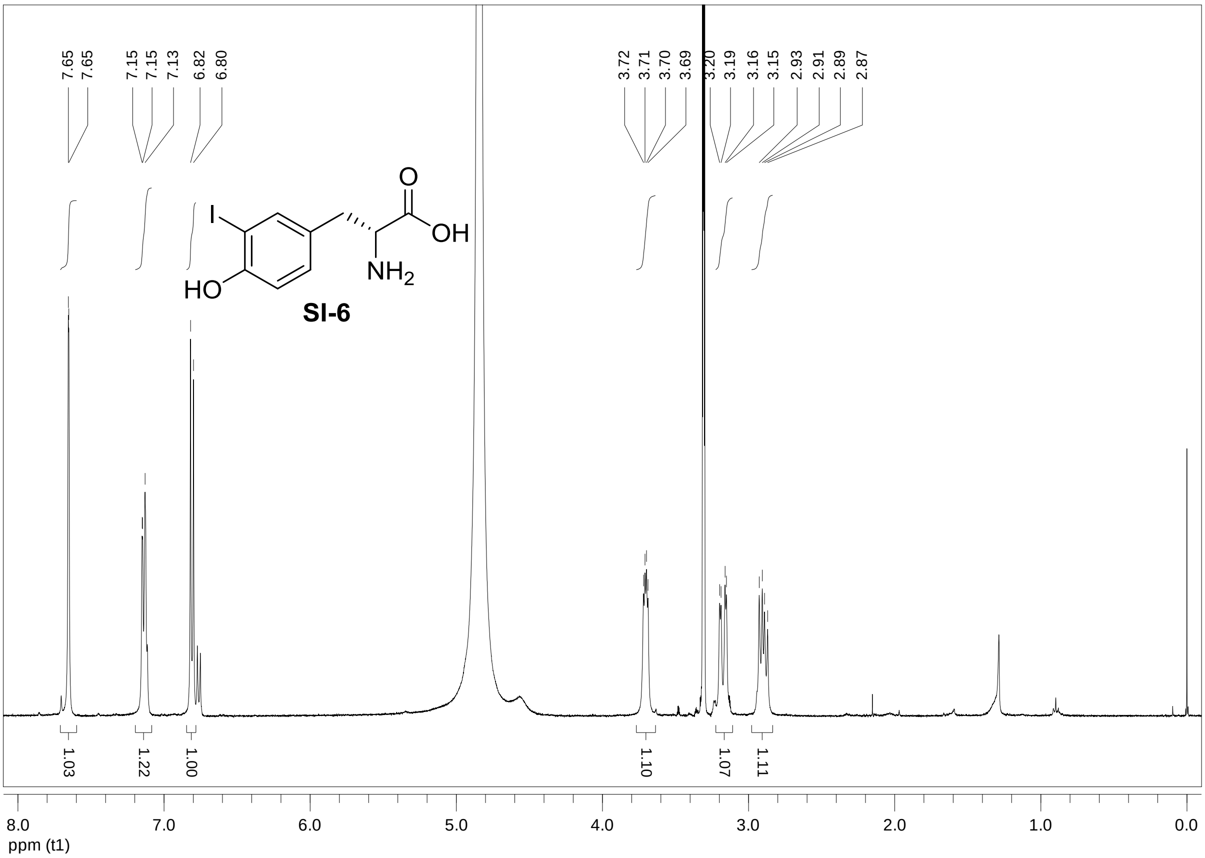
7.65
7.65
7.15
7.15
7.13
6.82
6.80
3.72
3.71
3.70
3.69
3.20
3.19
3.16
3.15
2.93
2.91
2.89
2.87
I
O
OH
NH2
HO
SI-6
1.03
1.22
1.00
1.10
1.07
1.11
8.0
7.0
6.0
5.0
4.0
3.0
2.0
1.0
0.0
ppm (t1)

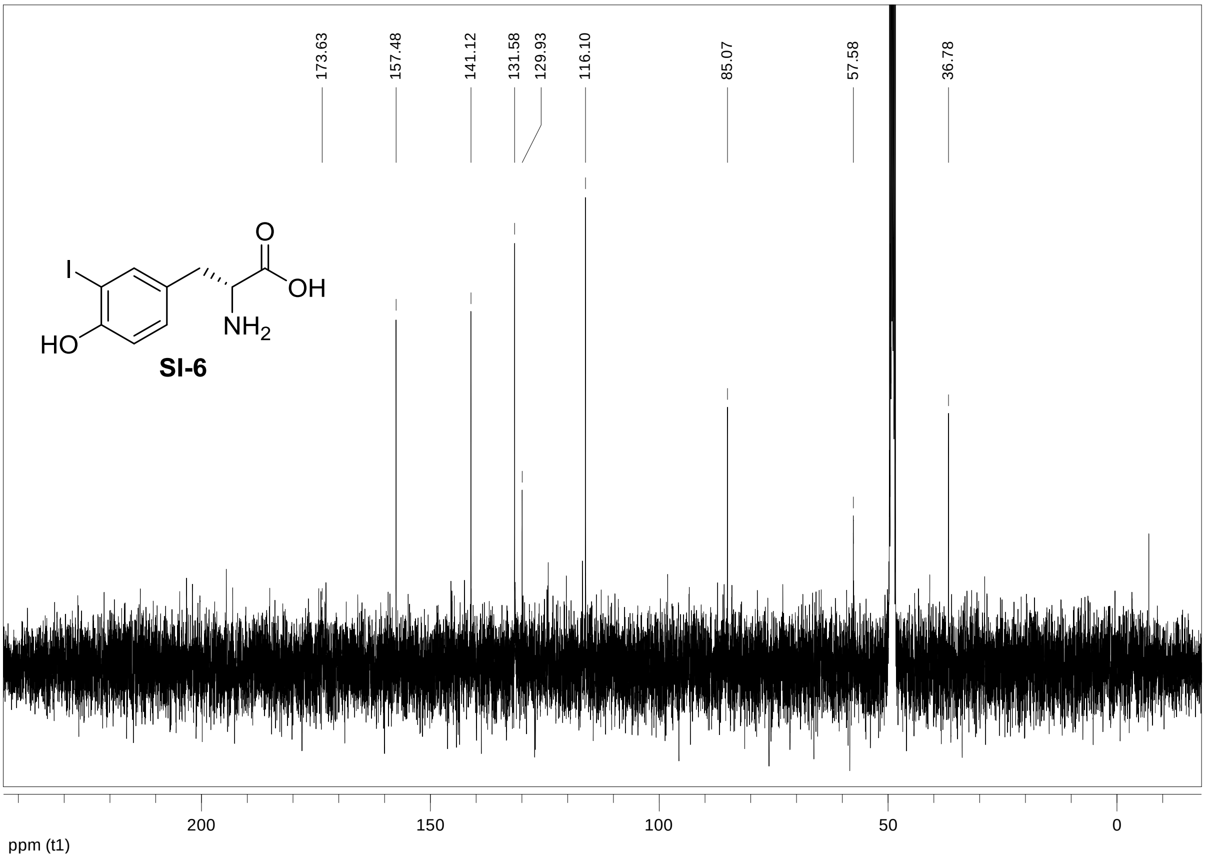
173.63
157.48
141.12
131.58
129.93
116.10
85.07
57.58
36.78
I
O
OH
NH2
HO
SI-6
200
150
100
50
0
ppm (t1)

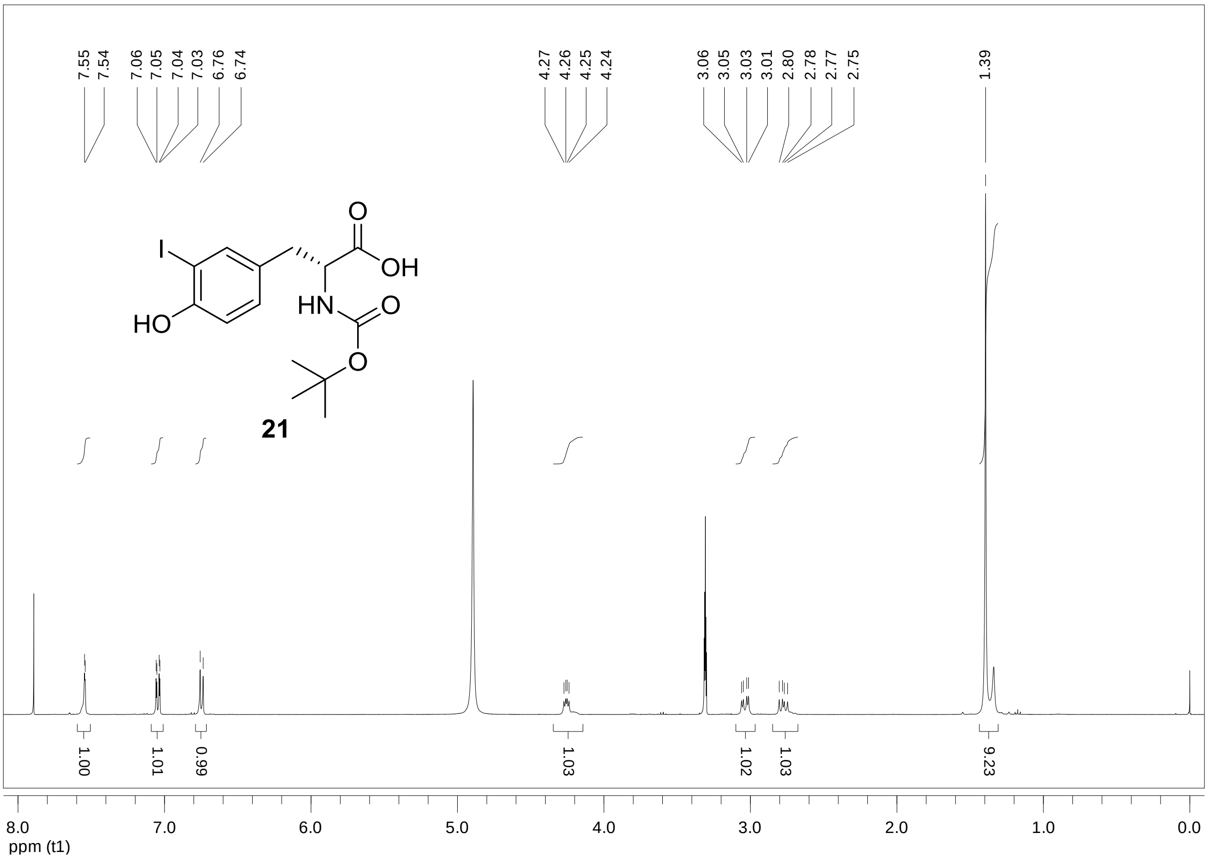
7.55
7.54
7.06
7.05
7.04
7.03
6.76
6.74
4.27
4.26
4.25
4.24
3.06
3.05
3.03
3.01
2.80
2.78
2.77
2.75
1.39
I
O
OH
HO
HN
O
O
21
1.00
1.01
0.99
1.03
1.02
1.03
9.23
8.0
7.0
6.0
5.0
4.0
3.0
2.0
1.0
0.0
ppm (t1)

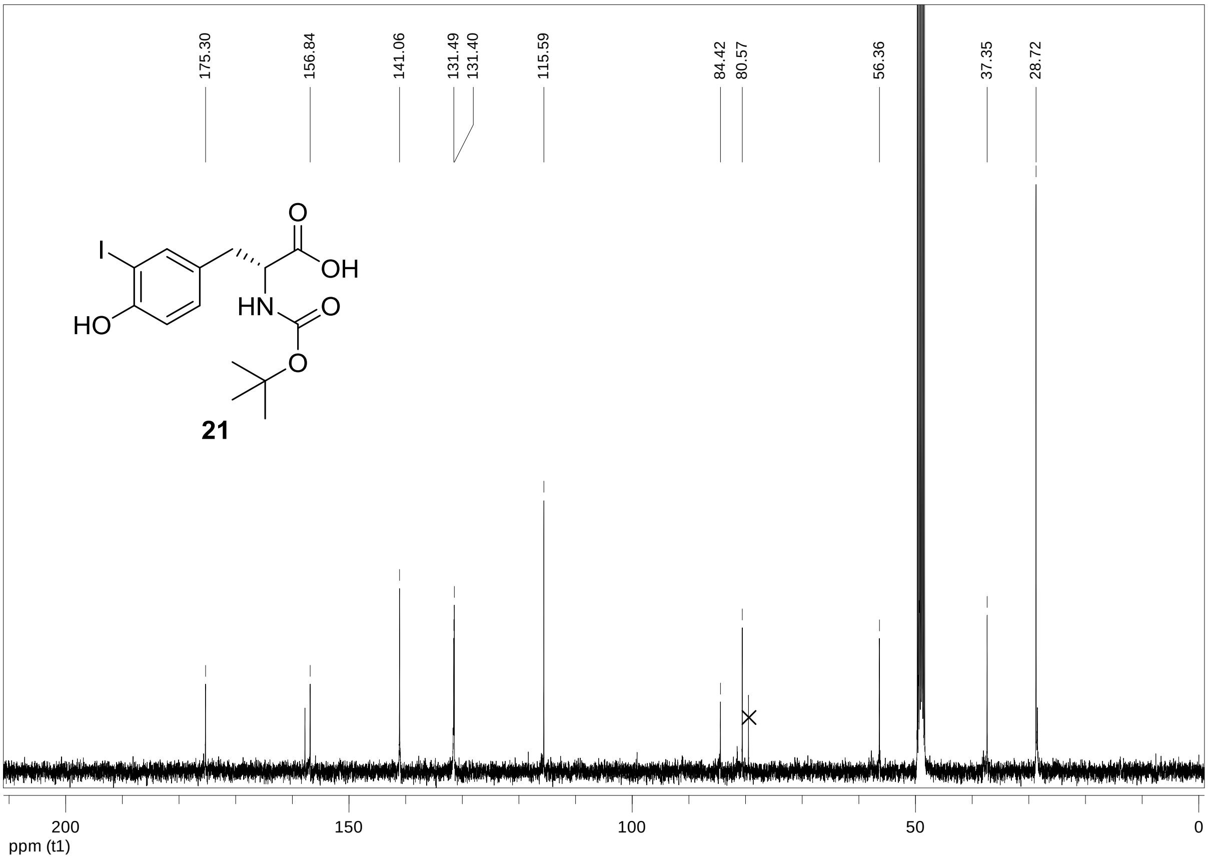
175.30
156.84
141.06
131.49
131.40
115.59
84.42
80.57
56.36
37.35
28.72
I
O
OH
HO
HN
O
O
21
200
150
100
50
0
ppm (t1)

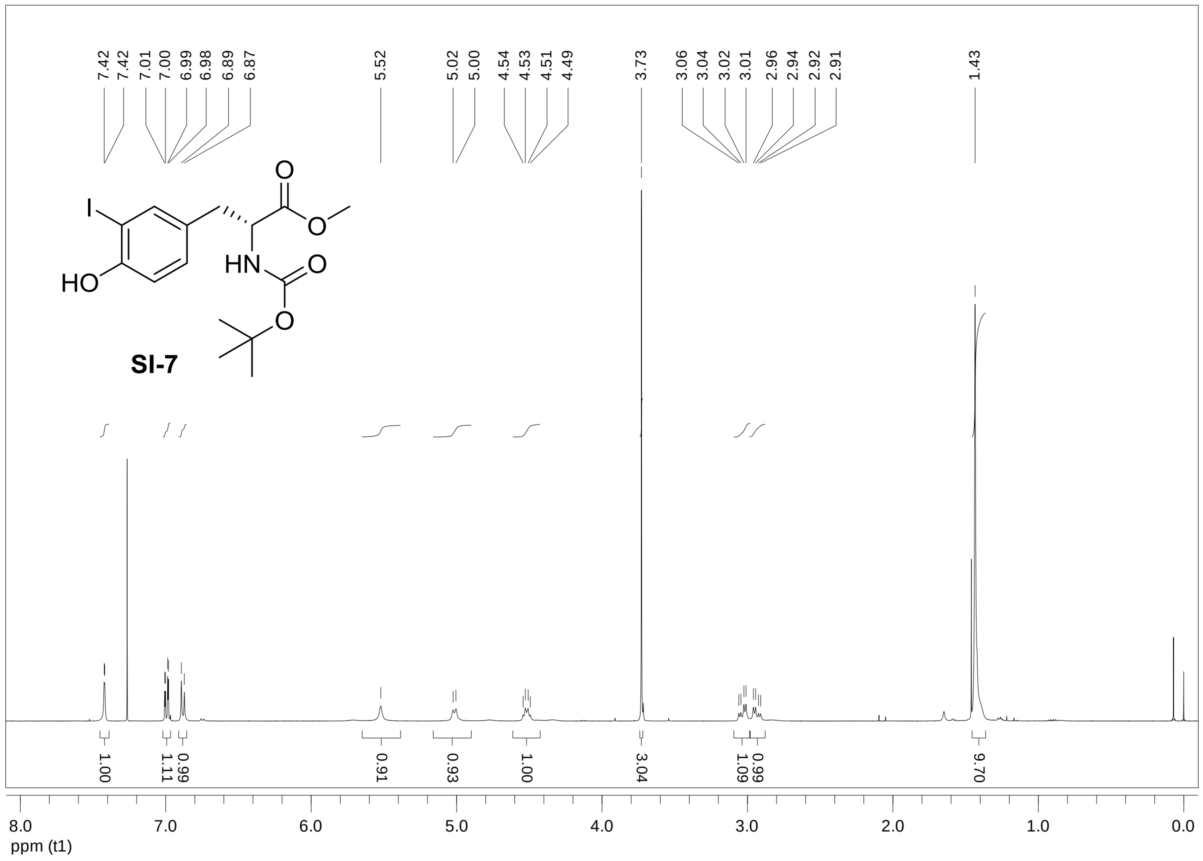
7.42
7.42
7.01
7.00
6.99
6.98
6.89
6.87
5.52
5.02
5.00
4.54
4.53
4.51
4.49
3.73
3.06
3.04
3.02
3.01
2.96
2.94
2.92
2.91
1.43
SI-7
1.00
1.11
0.99
0.91
0.93
1.00
3.04
1.09
0.99
9.70
8.0
7.0
6.0
5.0
4.0
3.0
2.0
1.0
0.0
ppm (t1)

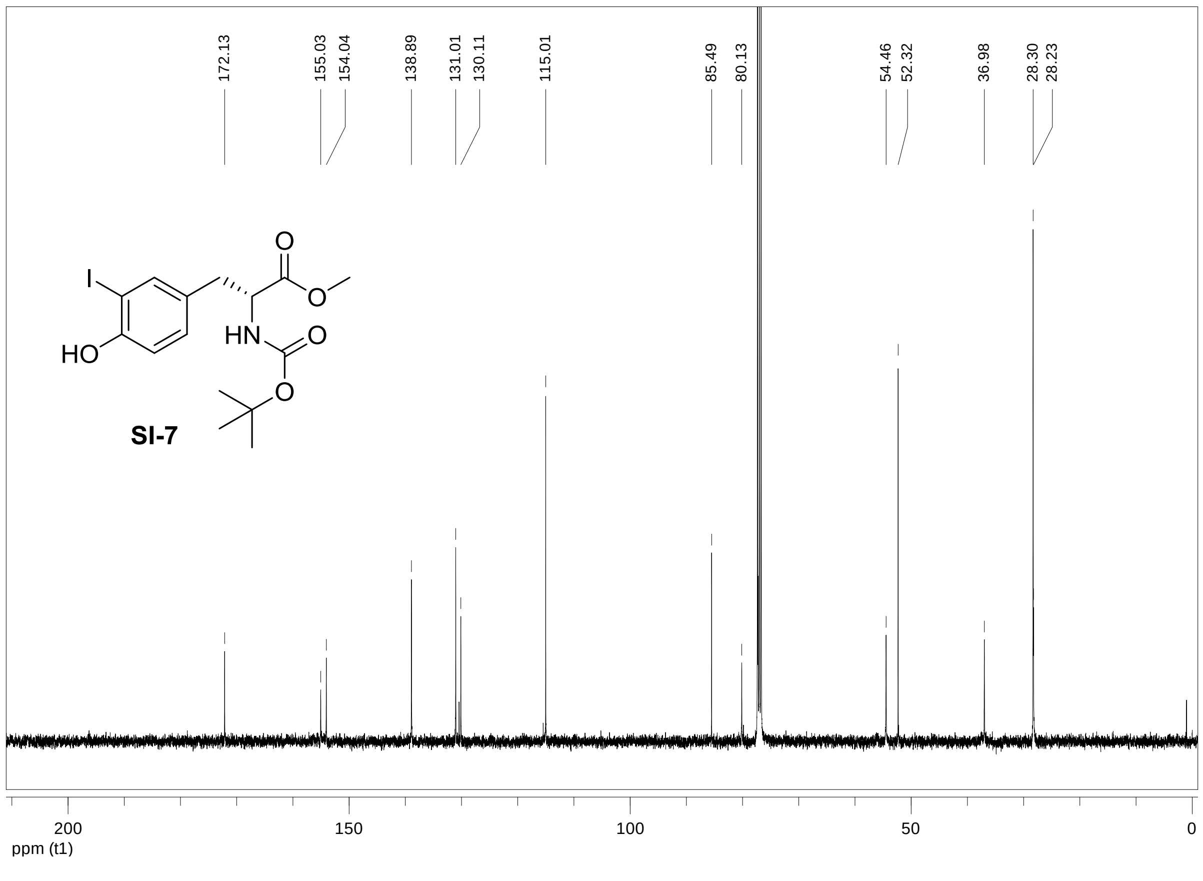
172.13
155.03
154.04
138.89
131.01
130.11
115.01
85.49
80.13
54.46
52.32
36.98
28.30
28.23
SI-7
200
150
100
50
0
ppm (t1)

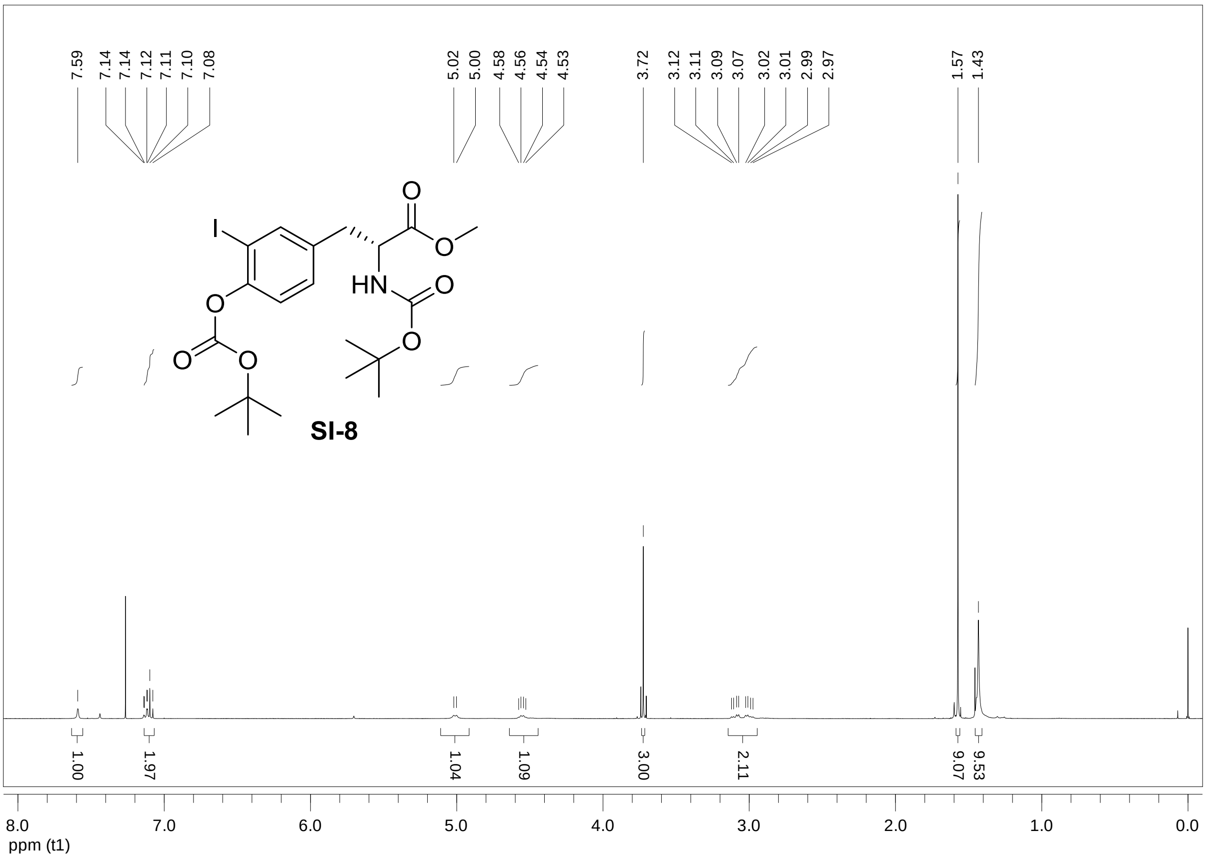
7.59
7.14
7.14
7.12
7.11
7.10
7.08
5.02
5.00
4.58
4.56
4.54
4.53
3.72
3.12
3.11
3.09
3.07
3.02
3.01
2.99
2.97
1.57
1.43
SI-8
1.00
1.97
1.04
1.09
3.00
2.11
9.07
9.53
8.0
7.0
6.0
5.0
4.0
3.0
2.0
1.0
0.0
ppm (t1)

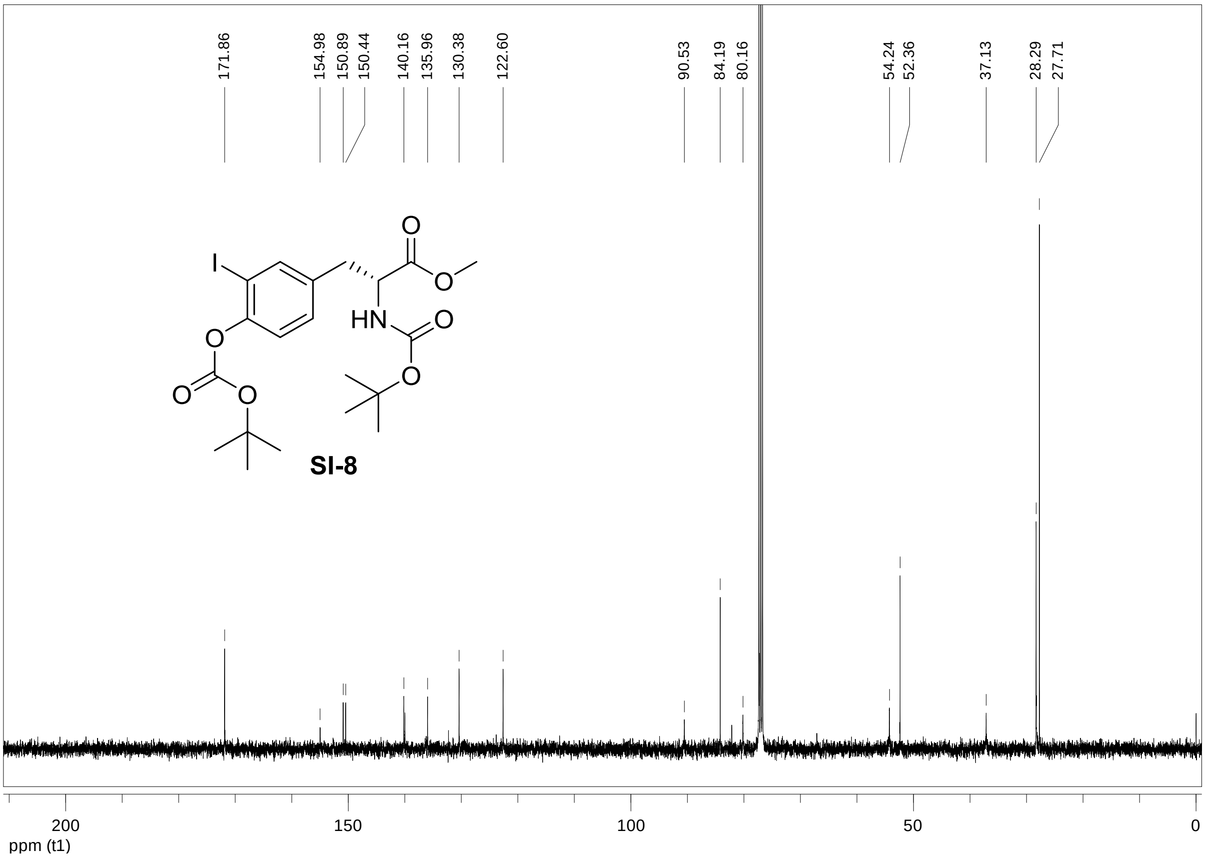
171.86
154.98
150.89
150.44
140.16
135.96
130.38
122.60
90.53
84.19
80.16
54.24
52.36
37.13
28.29
27.71
SI-8
200
150
100
50
0
ppm (t1)

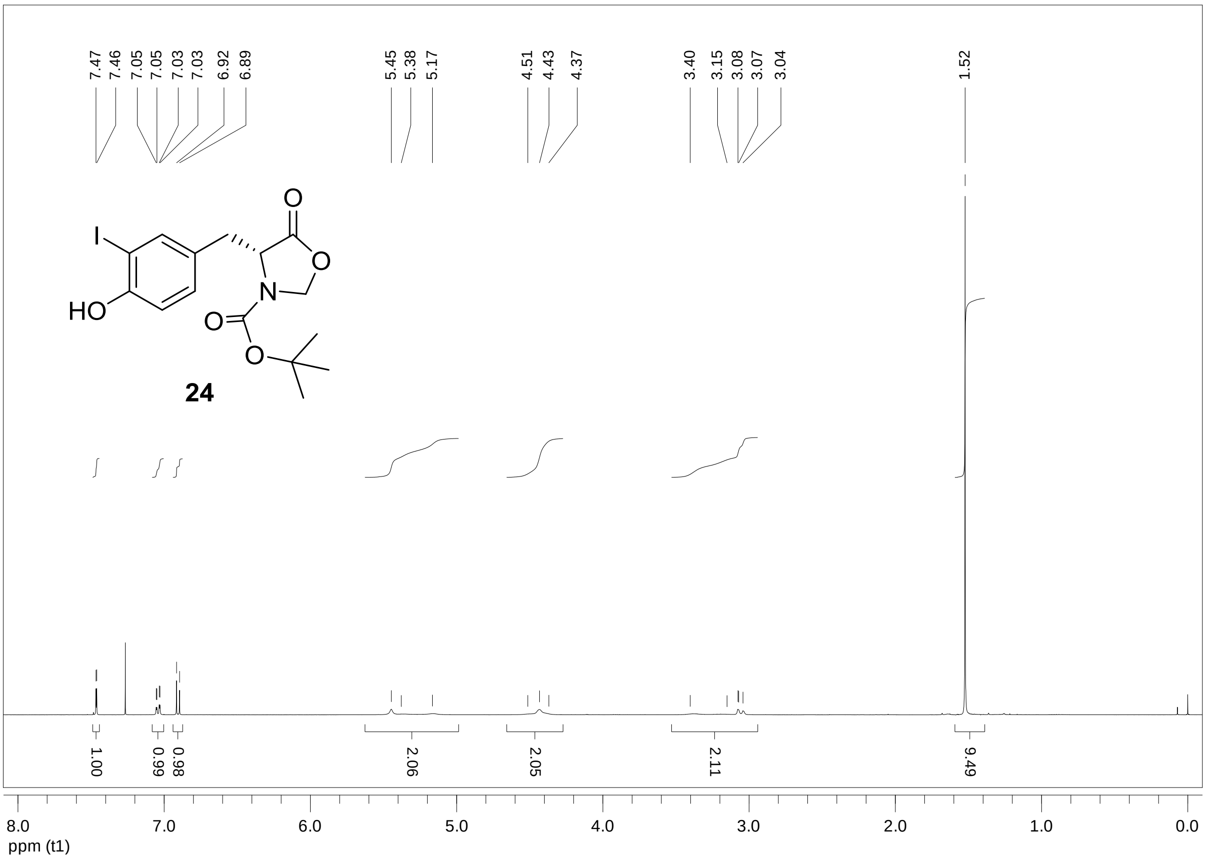
7.47
7.46
7.05
7.05
7.03
7.03
6.92
6.89
5.45
5.38
5.17
4.51
4.43
4.37
3.40
3.15
3.08
3.07
3.04
1.52
I
HO
O
O
N
O
O
24
1.00
0.99
0.98
2.06
2.05
2.11
9.49
8.0
7.0
6.0
5.0
4.0
3.0
2.0
1.0
0.0
ppm (t1)

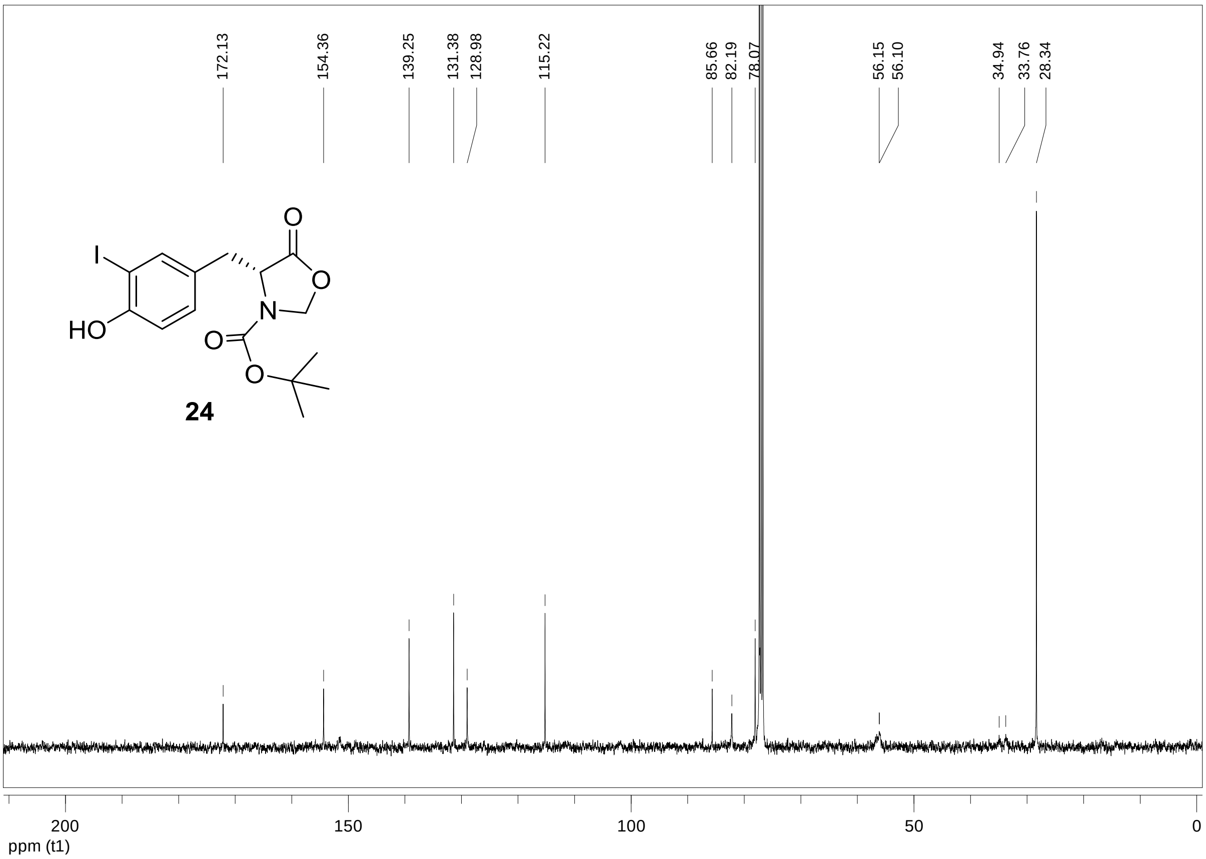
172.13
154.36
139.25
131.38
128.98
115.22
85.66
82.19
78.07
56.15
56.10
34.94
33.76
28.34
I
HO
O
O
N
O
O
24
200
150
100
50
0
ppm (t1)

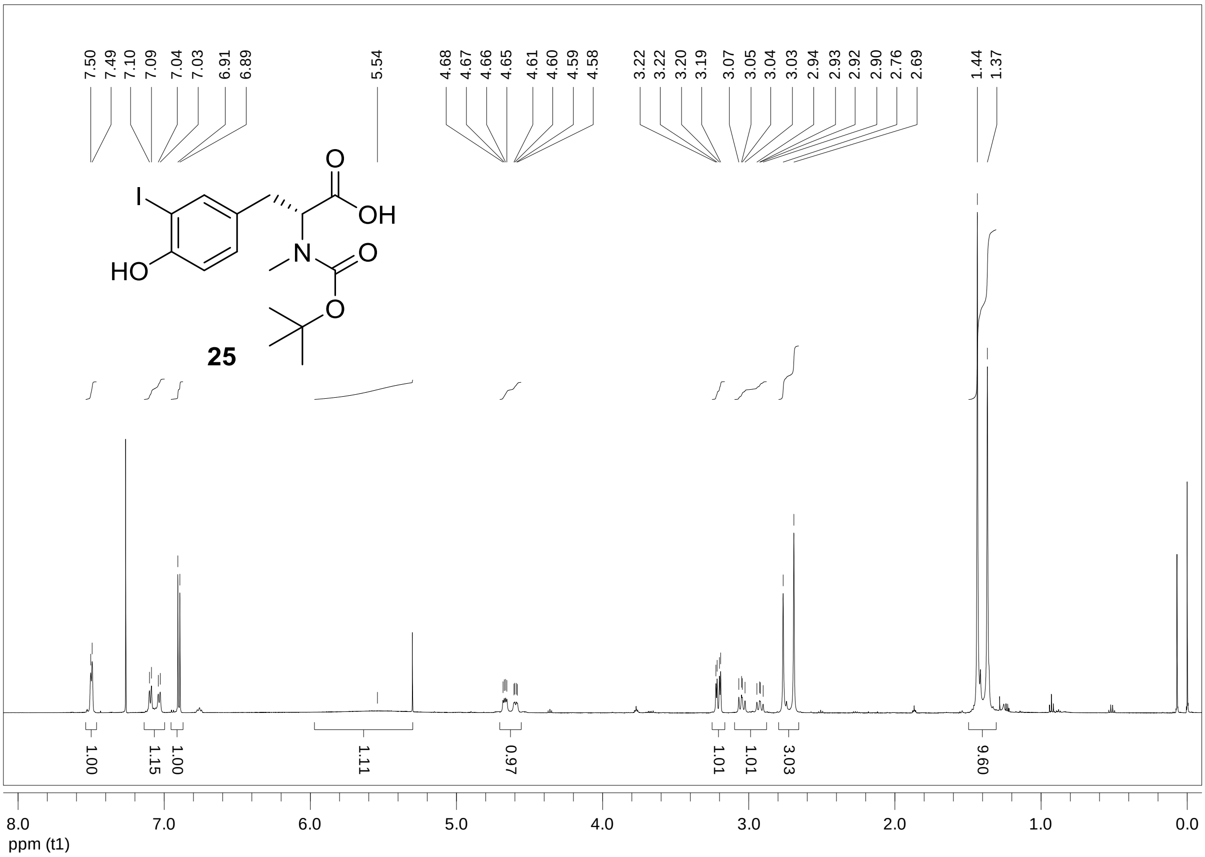
7.50
7.49
7.10
7.09
7.04
7.03
6.91
6.89
5.54
4.68
4.67
4.66
4.65
4.61
4.60
4.59
4.58
3.22
3.22
3.20
3.19
3.07
3.05
3.04
3.03
2.94
2.93
2.92
2.90
2.76
2.69
1.44
1.37
I
O
OH
N
O
HO
O
25
1.00
1.15
1.00
1.11
0.97
1.01
1.01
3.03
9.60
8.0
7.0
6.0
5.0
4.0
3.0
2.0
1.0
0.0
ppm (t1)

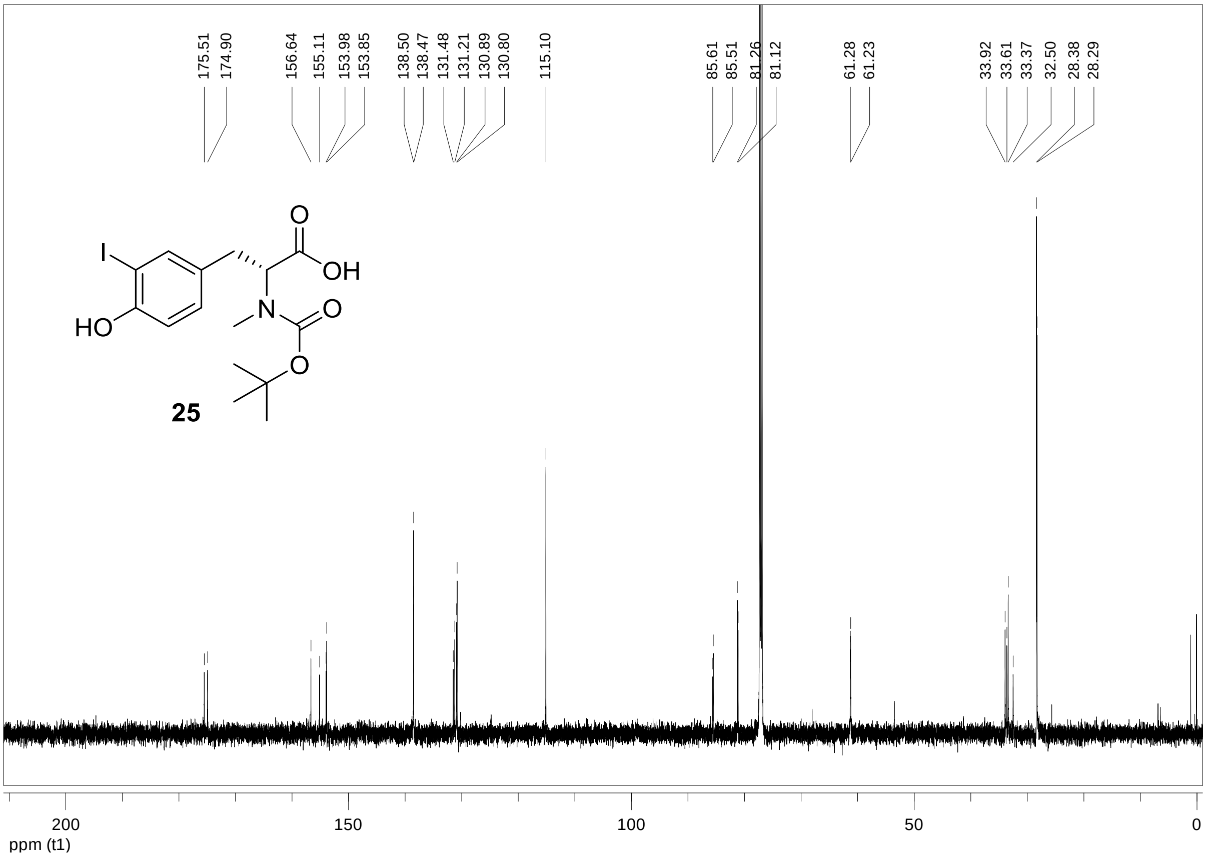
175.51
174.90
156.64
155.11
153.98
153.85
138.50
138.47
131.48
131.21
130.89
130.80
115.10
85.61
85.51
81.26
81.12
61.28
61.23
33.92
33.61
33.37
32.50
28.38
28.29
I
O
OH
N
O
HO
O
25
200
150
100
50
0
ppm (t1)

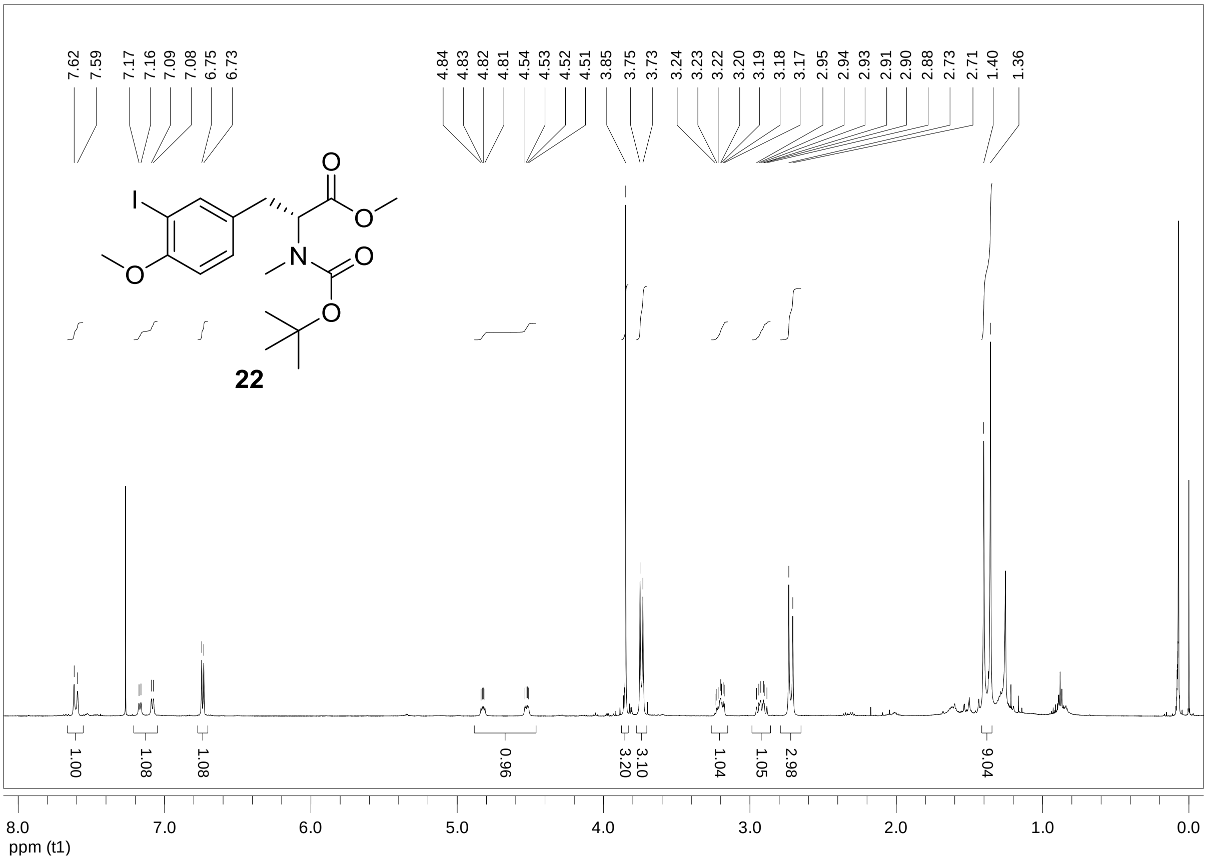
7.62 7.59 7.17 7.16 7.09 7.08 6.75 6.73
4.84 4.83 4.82 4.81 4.54 4.53 4.52 4.51 3.85 3.75 3.73 3.24 3.23 3.22 3.20 3.19 3.18 3.17 2.95 2.94 2.93 2.91 2.90 2.88 2.73 2.71 1.40 1.36
I
O
O
O
N
O
O
22
1.00 1.08 1.08 0.96 3.20 3.10 1.04 1.05 2.98 9.04
8.0 7.0 6.0 5.0 4.0 3.0 2.0 1.0 0.0
ppm (t1)

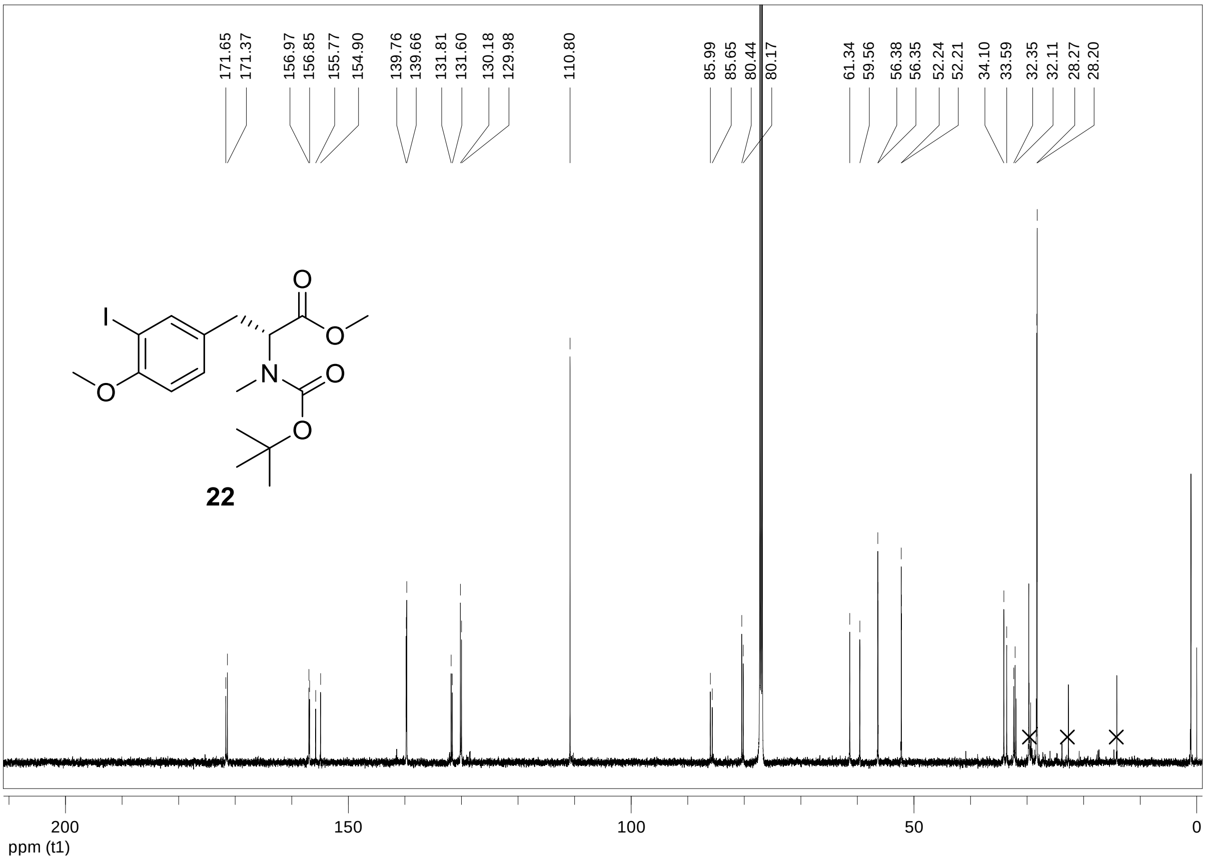
171.65 171.37 156.97 156.85 155.77 154.90 139.76 139.66 131.81 131.60 130.18 129.98 110.80
85.99 85.65 80.44 80.17 61.34 59.56 56.38 56.35 52.24 52.21 34.10 33.59 32.35 32.11 28.27 28.20
I
O
O
O
N
O
O
22
200 150 100 50 0
ppm (t1)

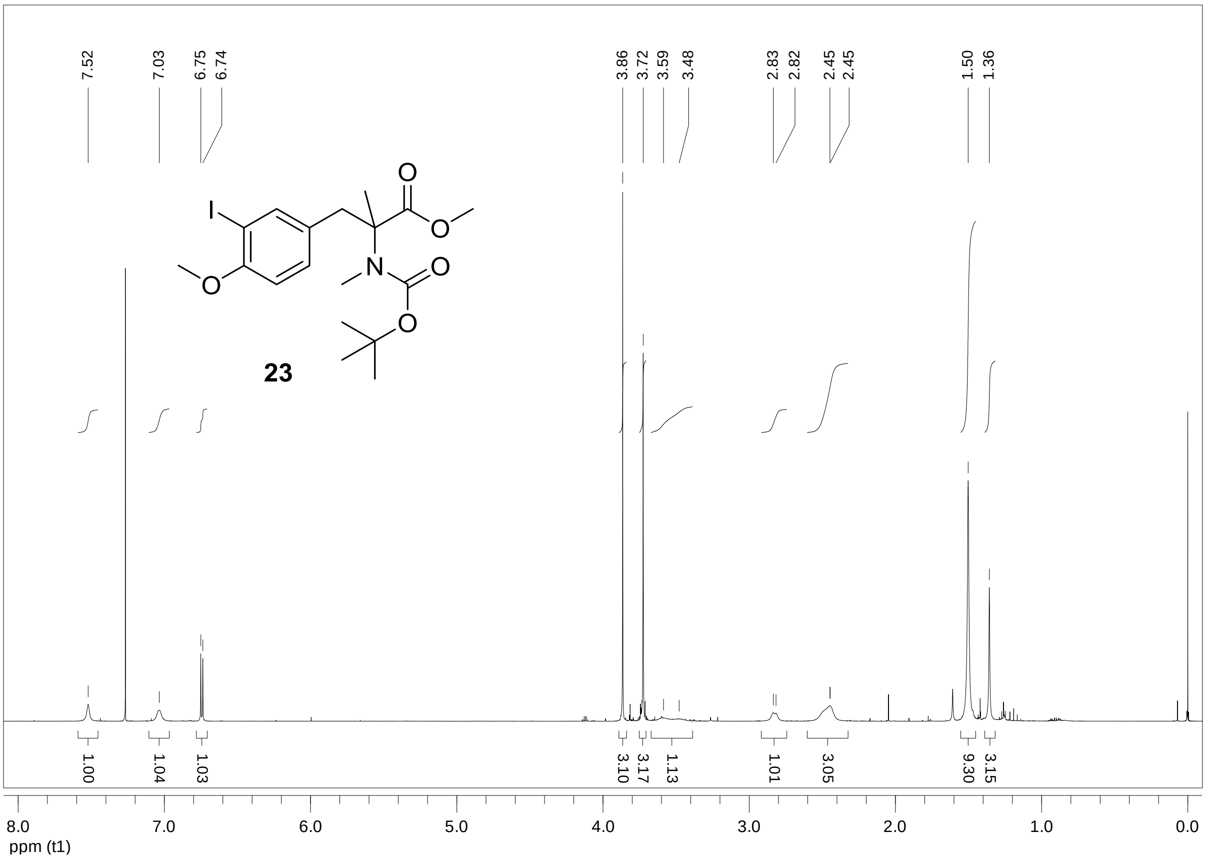
7.52
7.03
6.75
6.74
3.86
3.72
3.59
3.48
2.83
2.82
2.45
2.45
1.50
1.36
I
O
O
O
N
O
O
23
1.00
1.04
1.03
3.10
3.17
1.13
1.01
3.05
9.30
3.15
8.0
7.0
6.0
5.0
4.0
3.0
2.0
1.0
0.0
ppm (t1)

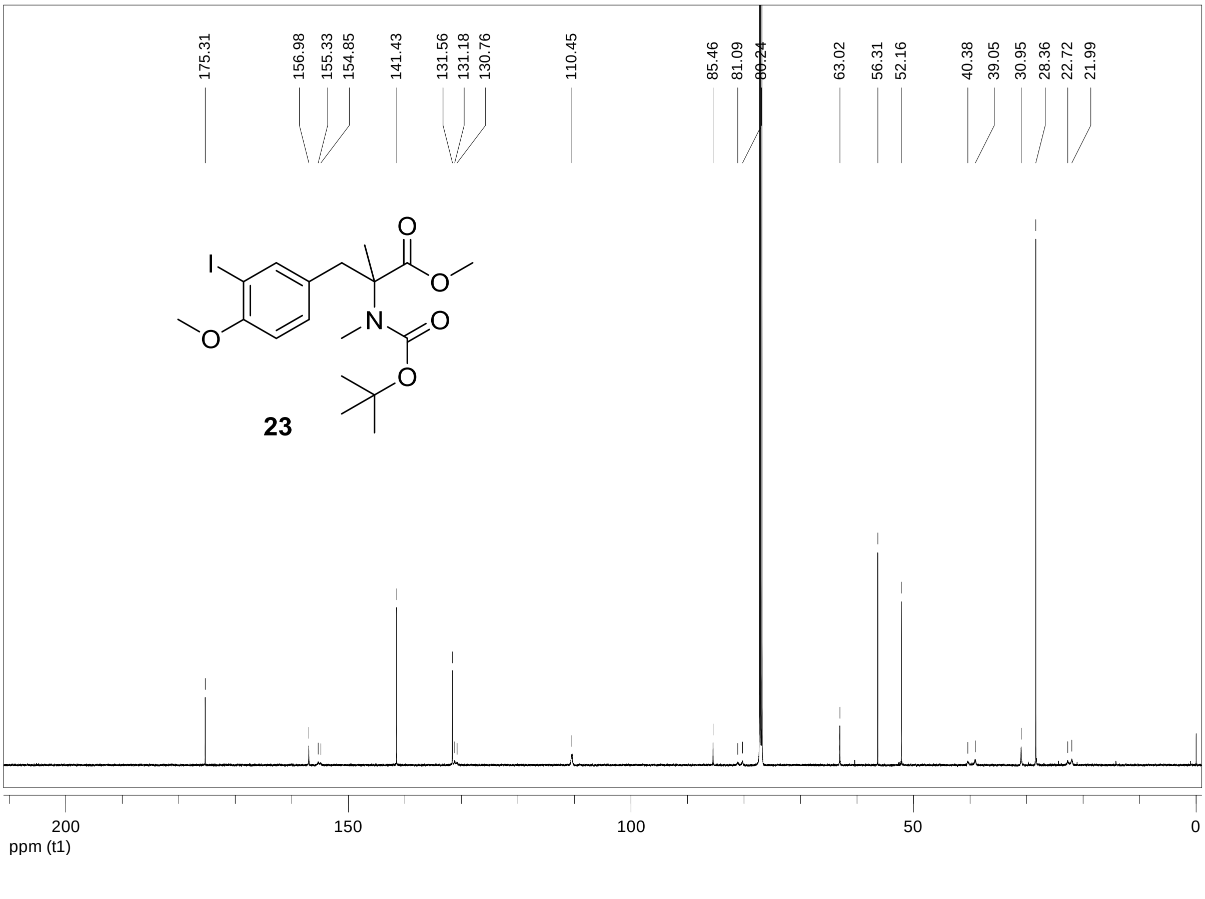
175.31
156.98
155.33
154.85
141.43
131.56
131.18
130.76
110.45
85.46
81.09
80.24
63.02
56.31
52.16
40.38
39.05
30.95
28.36
22.72
21.99
I
O
O
O
N
O
O
23
200
150
100
50
0
ppm (t1)

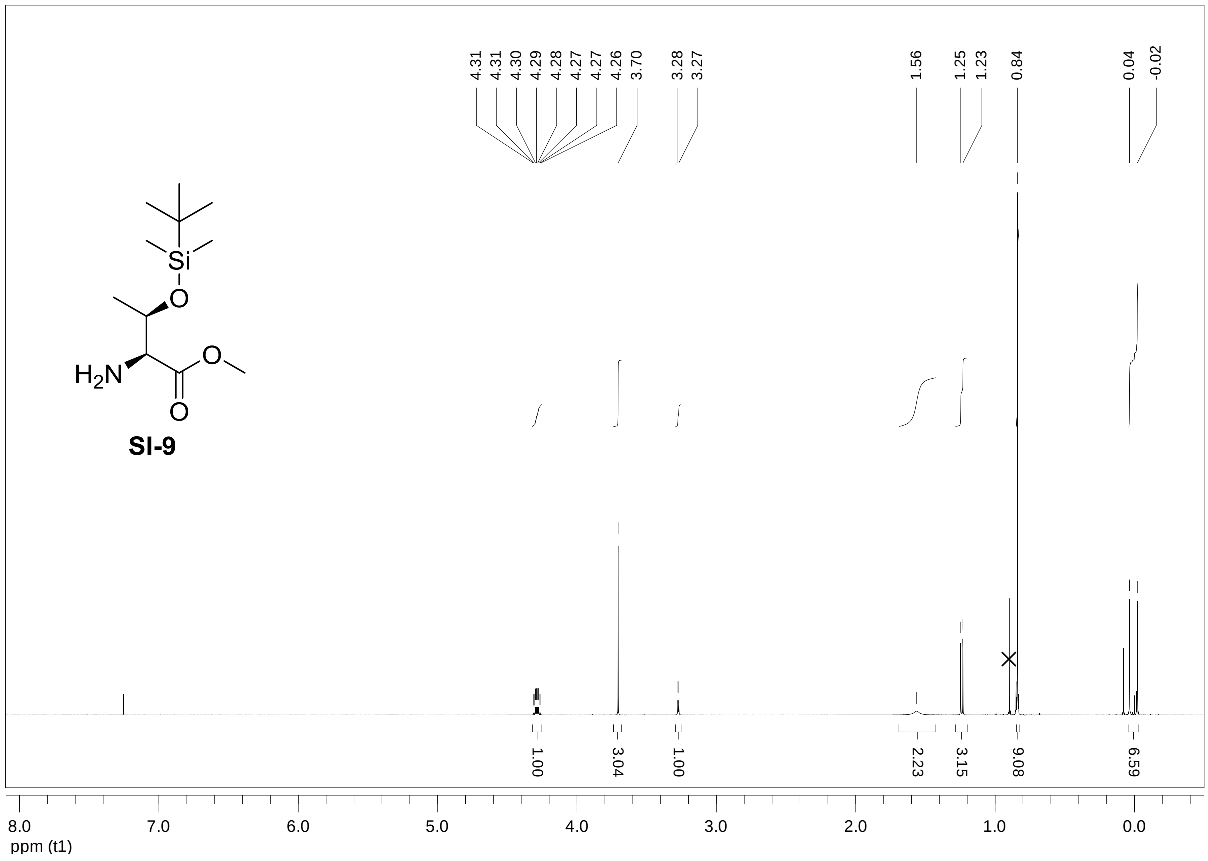

4.31 4.31 4.30 4.29 4.28 4.27 4.27 4.26 3.70 3.28 3.27 1.56 1.25 1.23 0.84 0.04 -0.02
H_2N
O
Si
SI-9
1.00 3.04 1.00 2.23 3.15 9.08 6.59
8.0 7.0 6.0 5.0 4.0 3.0 2.0 1.0 0.0
ppm (t1)

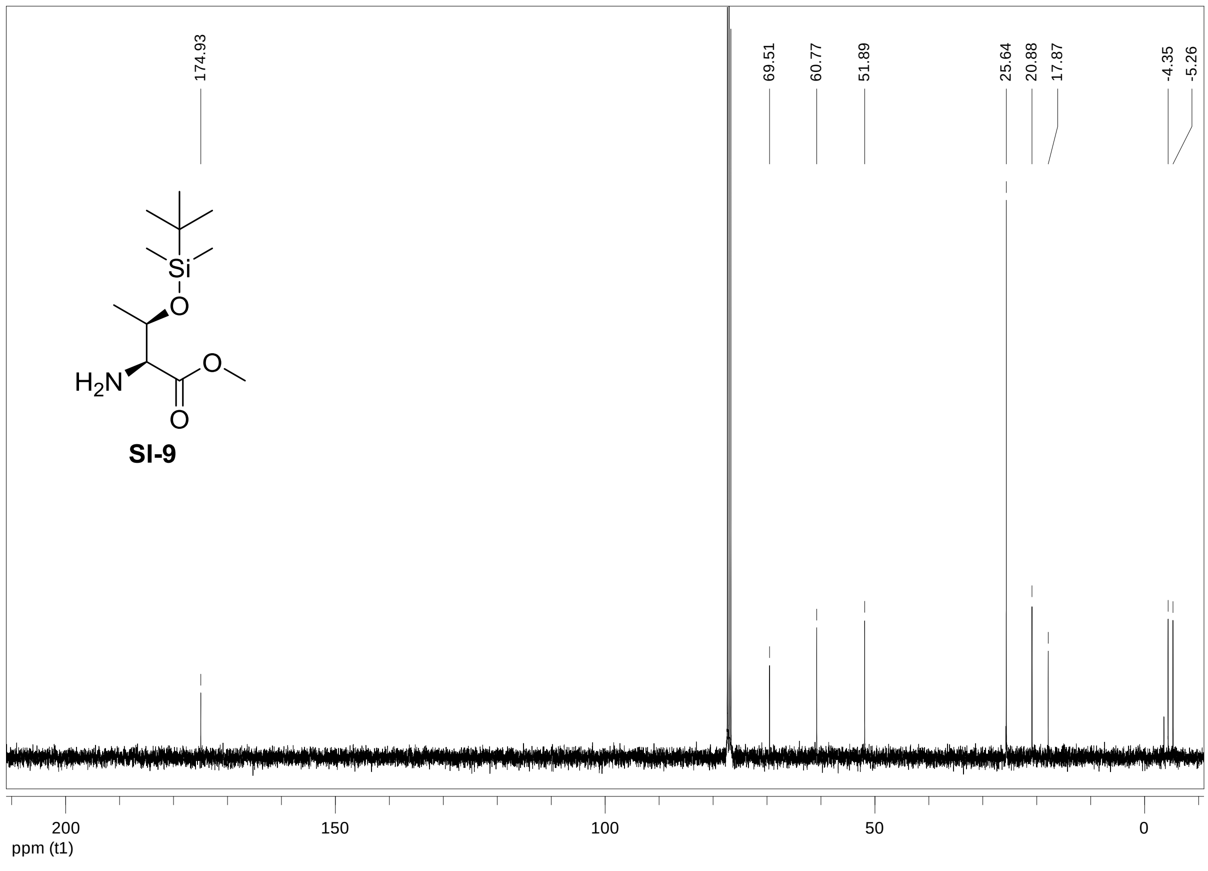

174.93 69.51 60.77 51.89 25.64 20.88 17.87 -4.35 -5.26
H_2N
O
Si
SI-9
200 150 100 50 0
ppm (t1)

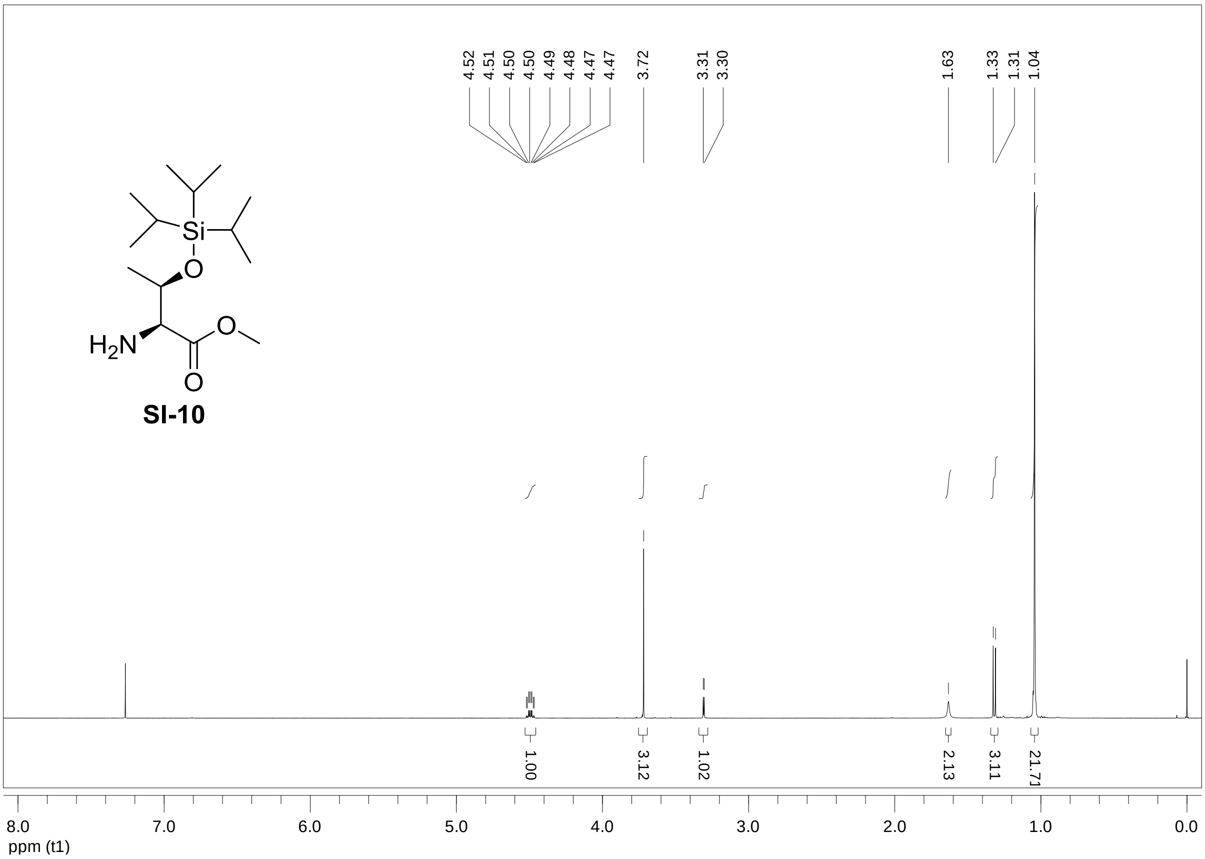
4.52 4.51 4.50 4.50 4.49 4.48 4.47 4.47 3.72 3.31 3.30 1.63 1.33 1.31 1.04
Si
O
H_2N
O
O
SI-10
1.00 3.12 1.02 2.13 3.11 21.71
8.0 7.0 6.0 5.0 4.0 3.0 2.0 1.0 0.0
ppm (t1)

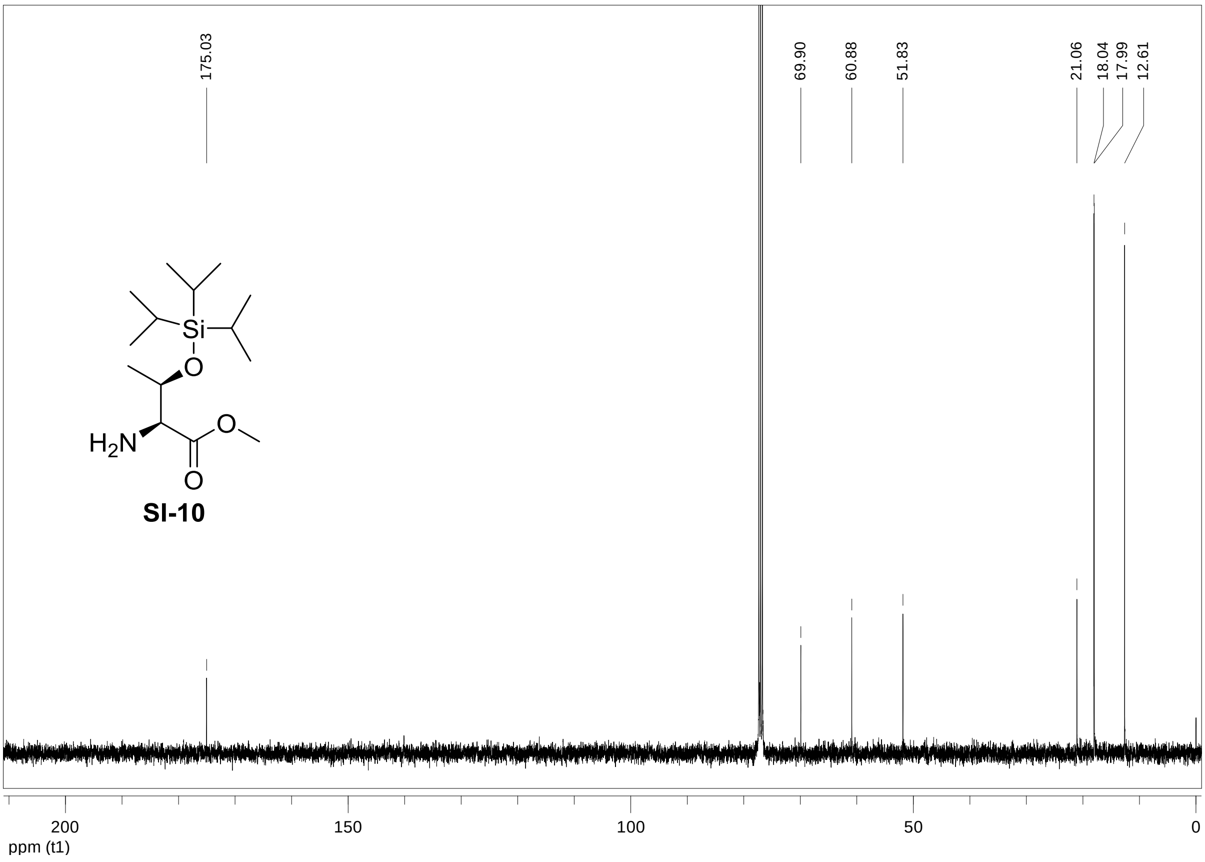
175.03 69.90 60.88 51.83 21.06 18.04 17.99 12.61
Si
O
H_2N
O
O
SI-10
200 150 100 50 0
ppm (t1)

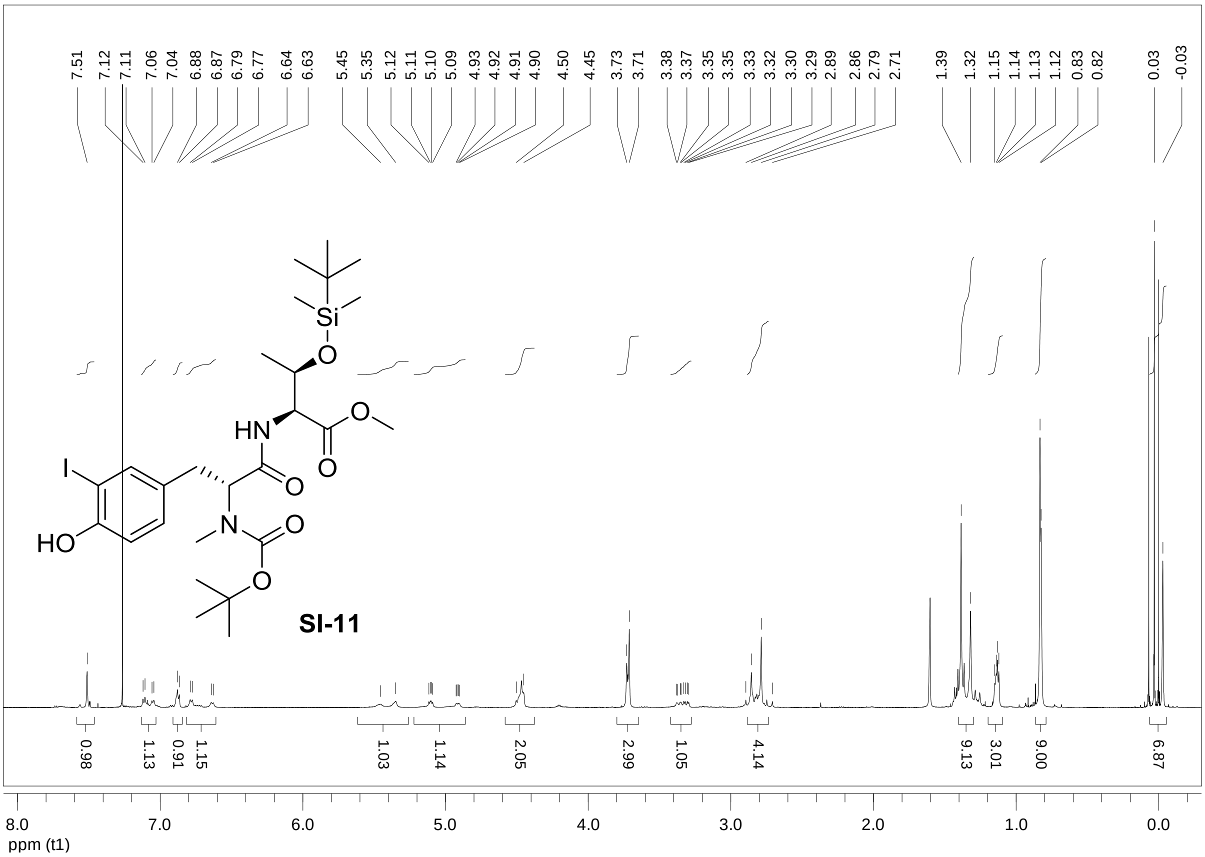
7.51
7.12
7.11
7.06
7.04
6.88
6.87
6.79
6.77
6.64
6.63
5.45
5.35
5.12
5.11
5.10
5.09
4.93
4.92
4.91
4.90
4.50
4.45
3.73
3.71
3.38
3.37
3.35
3.35
3.33
3.32
3.30
3.29
2.89
2.86
2.79
2.71
1.39
1.32
1.15
1.14
1.13
1.12
0.83
0.82
0.03
-0.03
SI-11
0.98
1.13
0.91
1.15
1.03
1.14
2.05
2.99
1.05
4.14
9.13
3.01
9.00
6.87
8.0
7.0
6.0
5.0
4.0
3.0
2.0
1.0
0.0
ppm (t1)

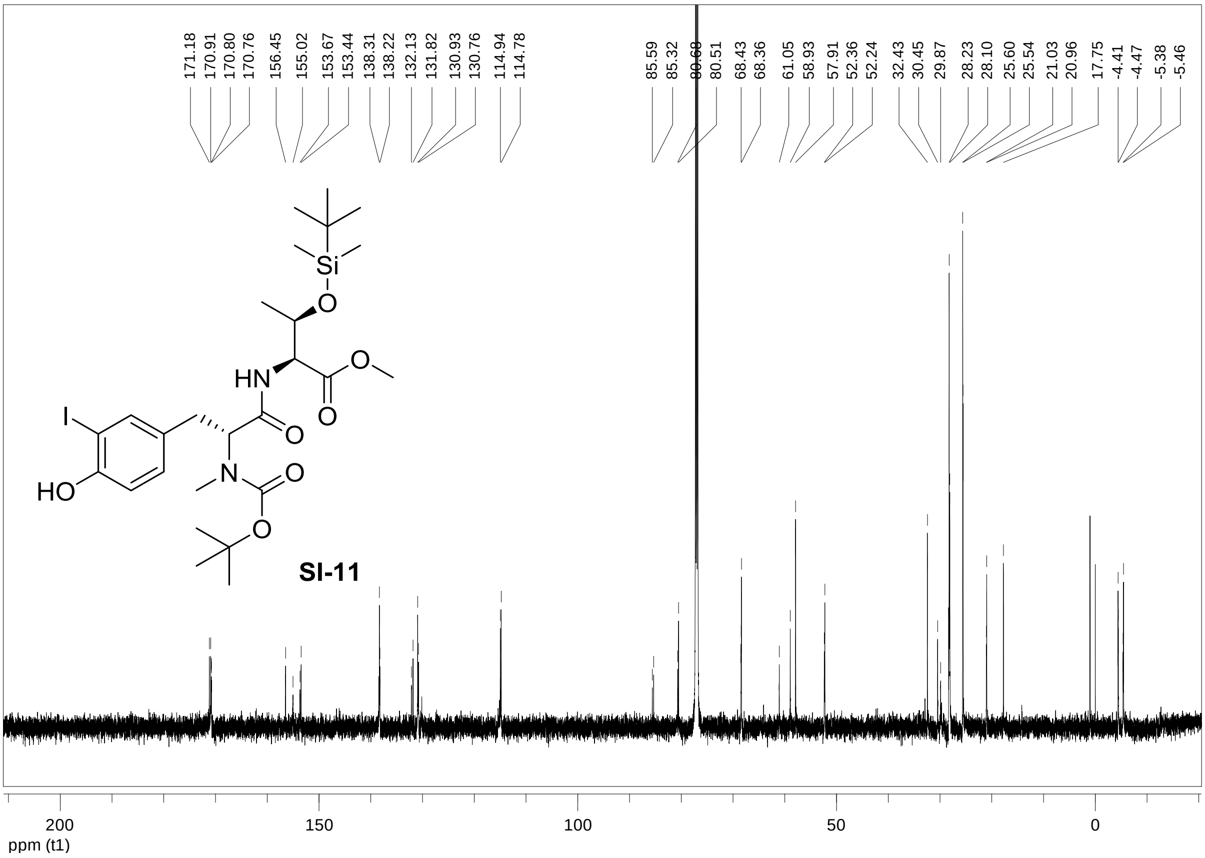
171.18
170.91
170.80
170.76
156.45
155.02
153.67
153.44
138.31
138.22
132.13
131.82
130.93
130.76
114.94
114.78
85.59
85.32
80.68
80.51
68.43
68.36
61.05
58.93
57.91
52.36
52.24
32.43
30.45
29.87
28.23
28.10
25.60
25.54
21.03
20.96
17.75
-4.41
-4.47
-5.38
-5.46
SI-11
200
150
100
50
0
ppm (t1)

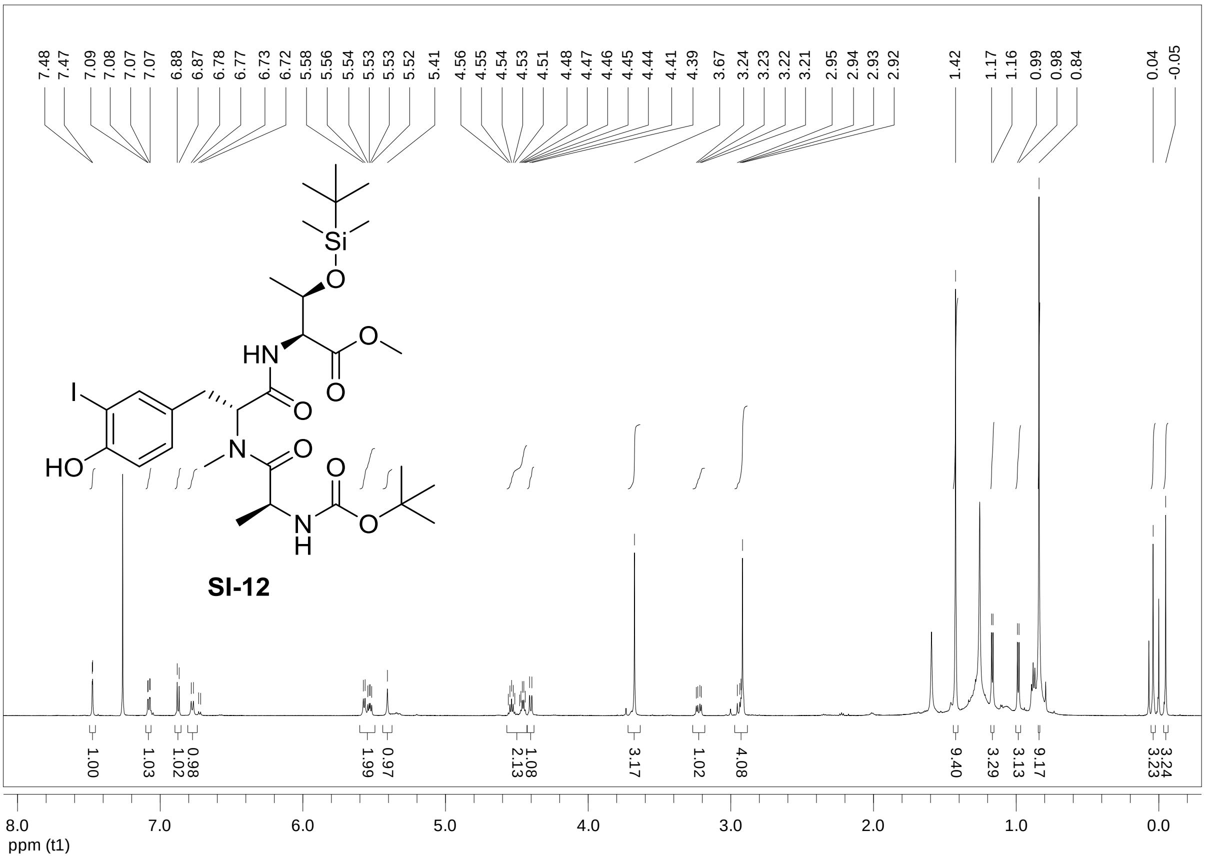

7.48
7.47
7.09
7.08
7.07
7.07
6.88
6.87
6.78
6.77
6.73
6.72
5.58
5.56
5.54
5.53
5.53
5.52
5.41
4.56
4.55
4.54
4.53
4.51
4.48
4.47
4.46
4.45
4.44
4.41
4.39
3.67
3.24
3.23
3.22
3.21
2.95
2.94
2.93
2.92
1.42
1.17
1.16
0.99
0.98
0.84
0.04
-0.05
SI-12
1.00
1.03
1.02
0.98
1.99
0.97
2.13
1.08
3.17
1.02
4.08
9.40
3.29
3.13
9.17
3.24
3.23
8.0
7.0
6.0
5.0
4.0
3.0
2.0
1.0
0.0
ppm (t1)

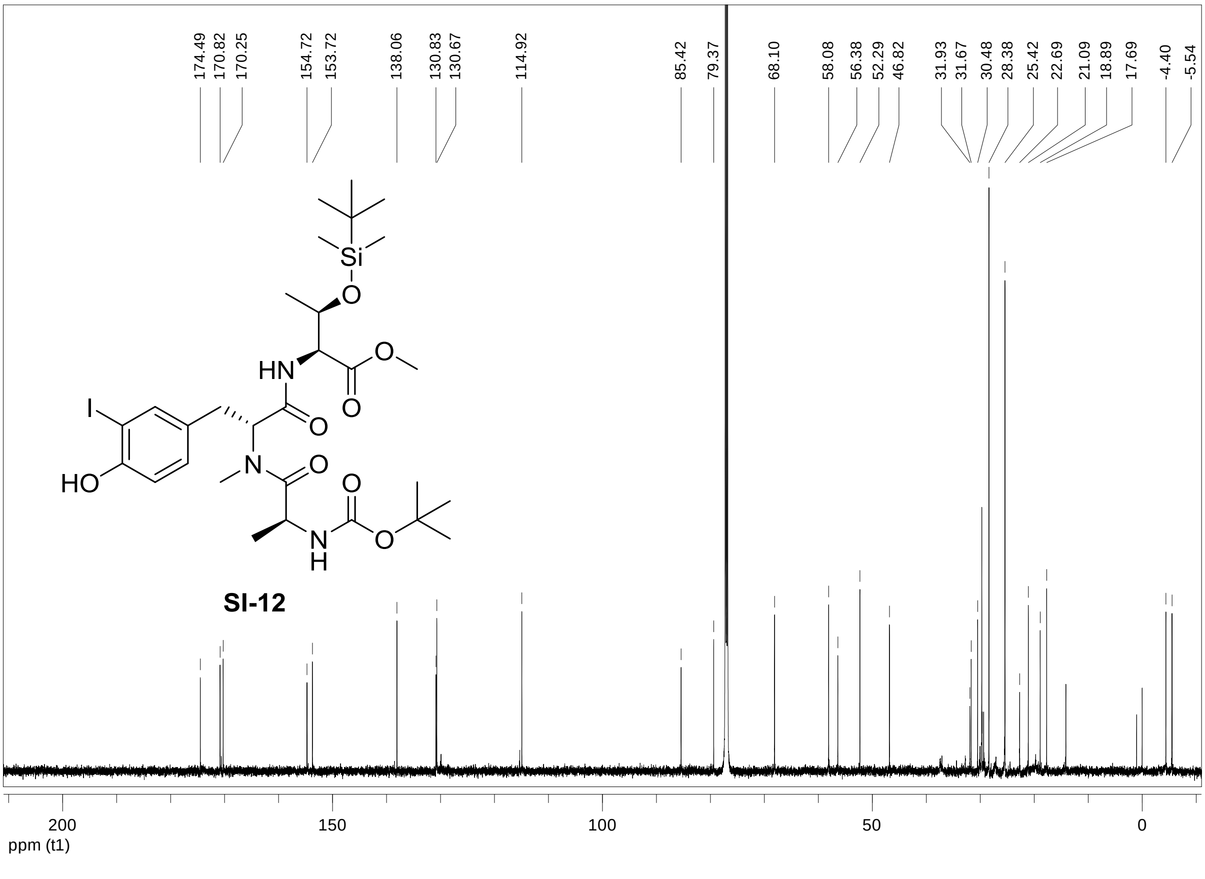

174.49
170.82
170.25
154.72
153.72
138.06
130.83
130.67
114.92
85.42
79.37
68.10
58.08
56.38
52.29
46.82
31.93
31.67
30.48
28.38
25.42
22.69
21.09
18.89
17.69
-4.40
-5.54
SI-12
200
150
100
50
0
ppm (t1)

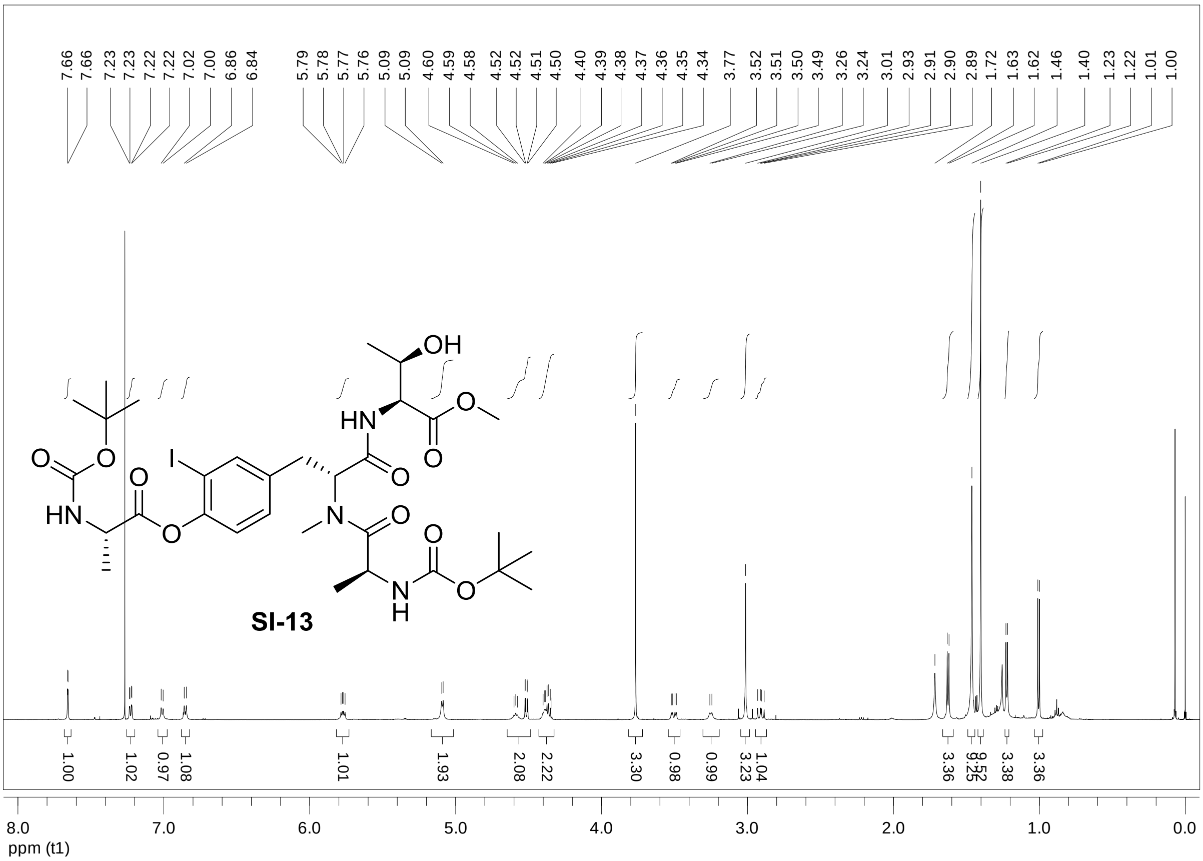
7.66 7.66 7.23 7.23 7.22 7.22 7.02 7.00 6.86 6.84 5.79 5.78 5.77 5.76 5.09 5.09 4.60 4.59 4.58 4.52 4.52 4.51 4.50 4.40 4.39 4.38 4.37 4.36 4.35 4.34 3.77 3.52 3.51 3.50 3.49 3.26 3.24 3.01 2.93 2.91 2.90 2.89 1.72 1.63 1.62 1.46 1.40 1.23 1.22 1.01 1.00
1.00 1.02 0.97 1.08 1.01 1.93 2.08 2.22 3.30 0.98 0.99 3.23 1.04 3.36 9.25 9.52 3.38 3.36
OH
HN
O
I
N
H
SI-13
8.0 7.0 6.0 5.0 4.0 3.0 2.0 1.0 0.0
ppm (t1)

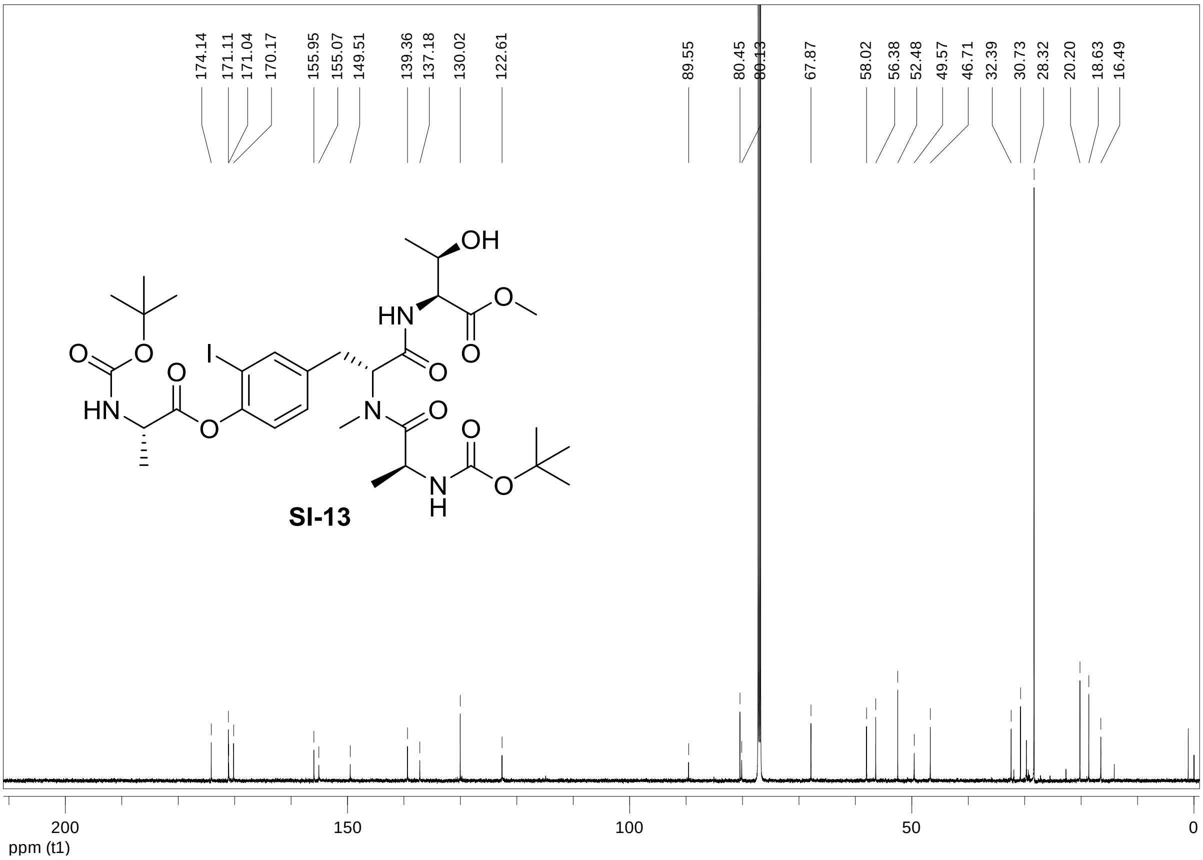
174.14 171.11 171.04 170.17 155.95 155.07 149.51 139.36 137.18 130.02 122.61 89.55 80.45 80.13 67.87 58.02 56.38 52.48 49.57 46.71 32.39 30.73 28.32 20.20 18.63 16.49
OH
HN
O
I
N
H
SI-13
200 150 100 50 0
ppm (t1)

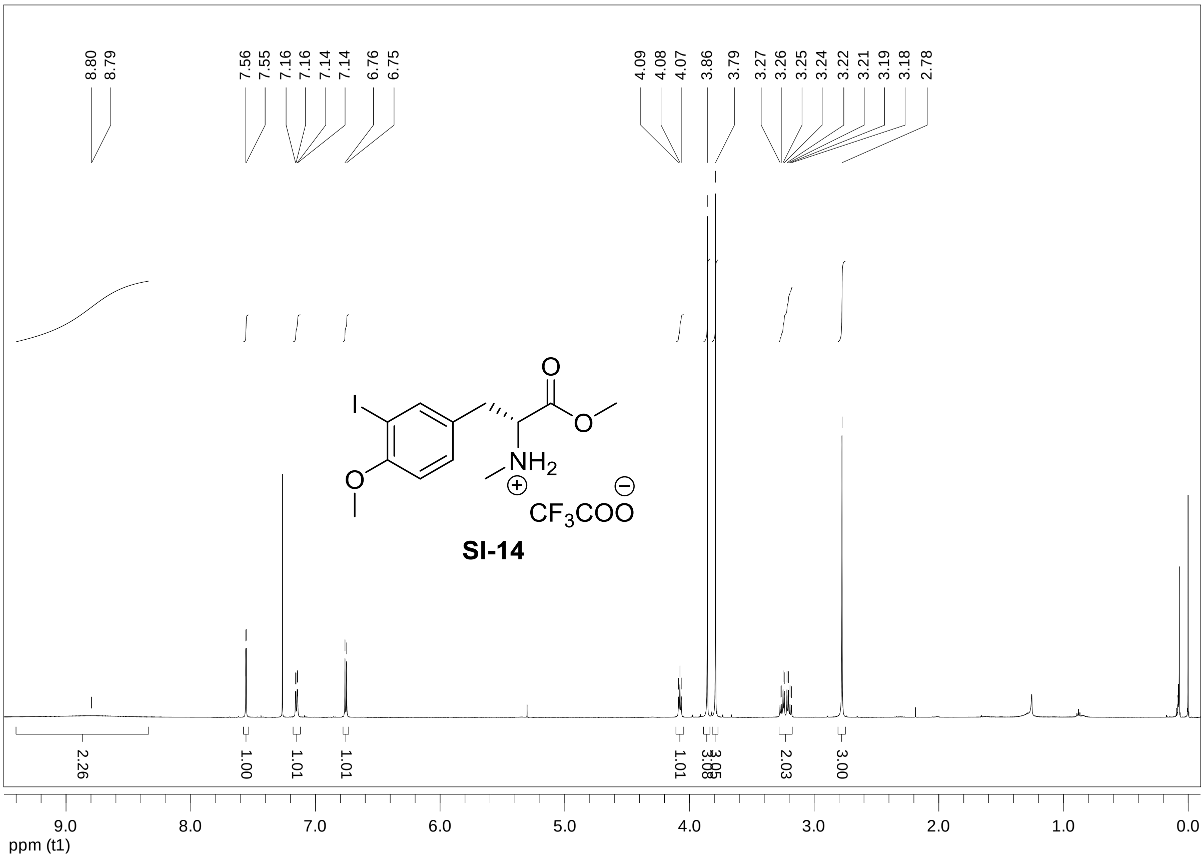
8.80
8.79
7.56
7.55
7.16
7.16
7.14
7.14
6.76
6.75
4.09
4.08
4.07
3.86
3.79
3.27
3.26
3.25
3.24
3.22
3.21
3.19
3.18
2.78
I
O
NH2
CF3COO
SI-14
2.26
1.00
1.01
1.01
1.01
2.03
3.00
9.0
8.0
7.0
6.0
5.0
4.0
3.0
2.0
1.0
0.0
ppm (t1)

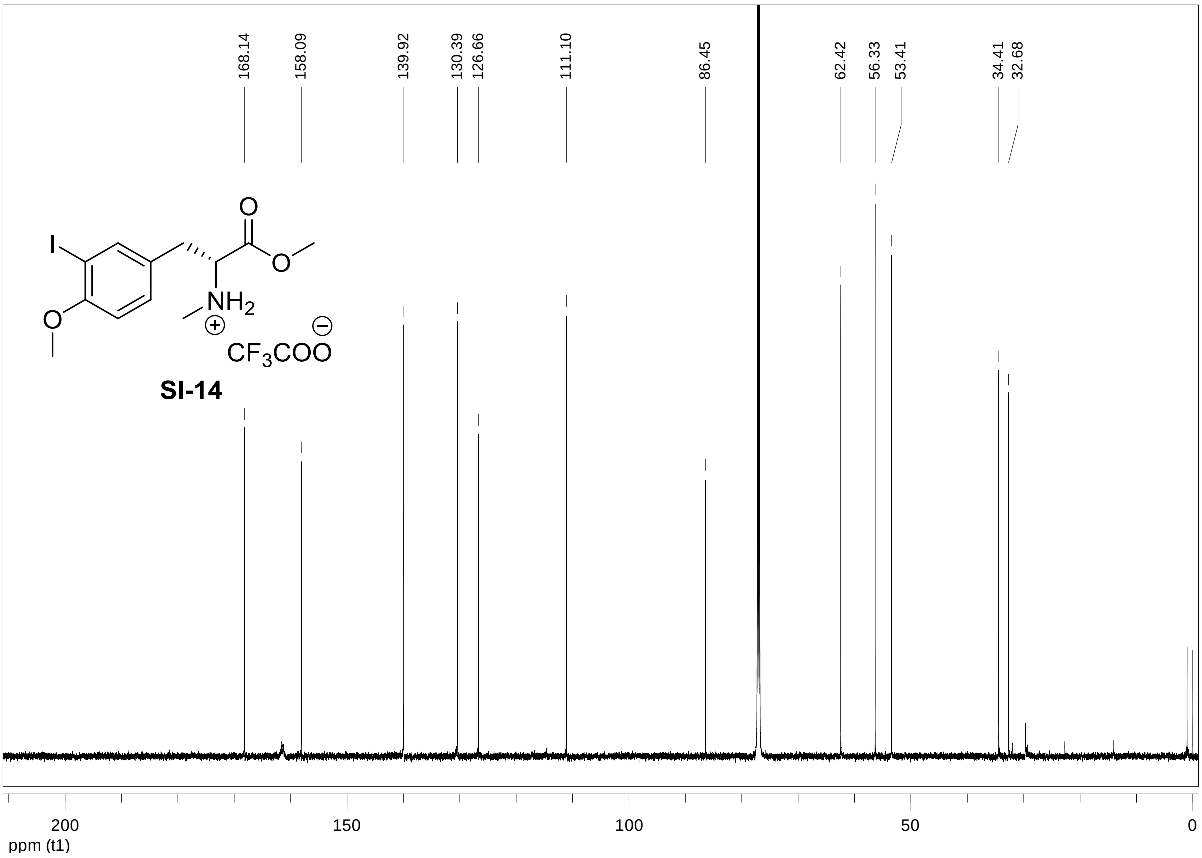
168.14
158.09
139.92
130.39
126.66
111.10
86.45
62.42
56.33
53.41
34.41
32.68
I
O
NH2
CF3COO
SI-14
200
150
100
50
0
ppm (t1)

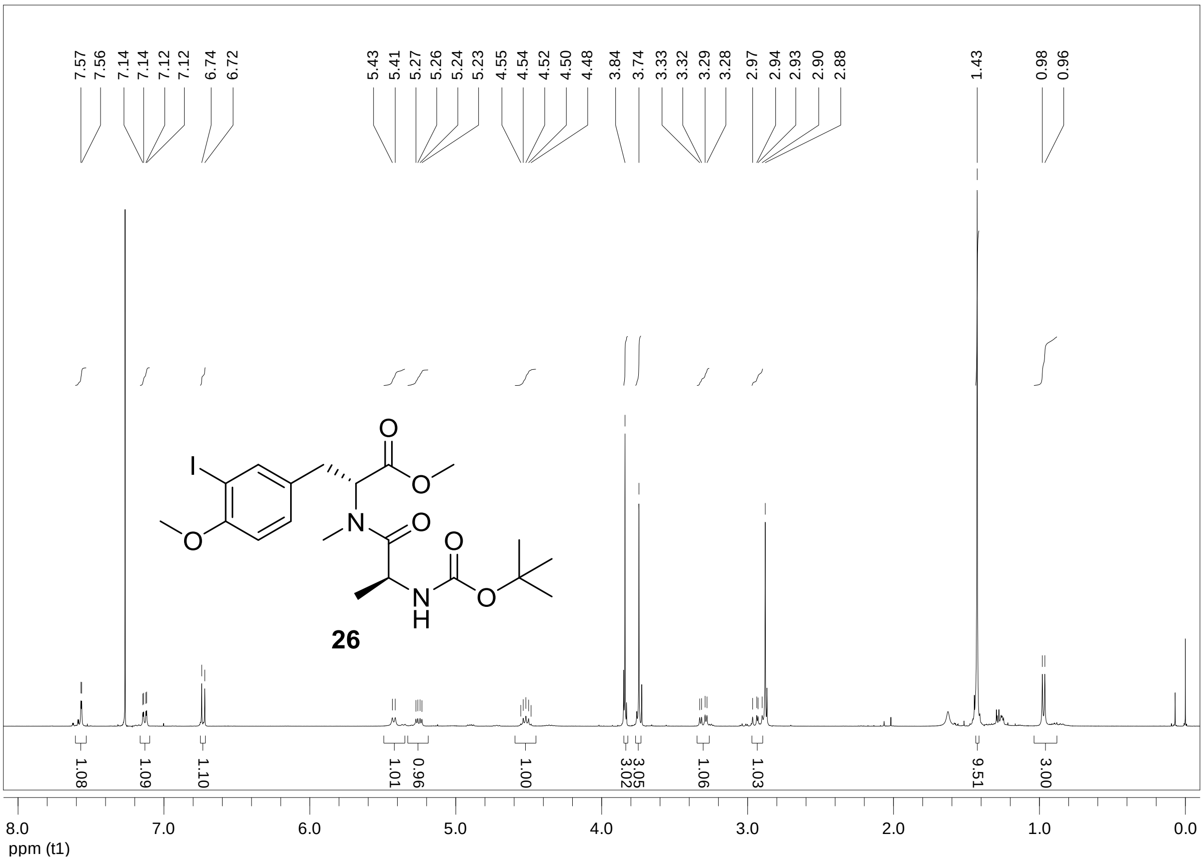

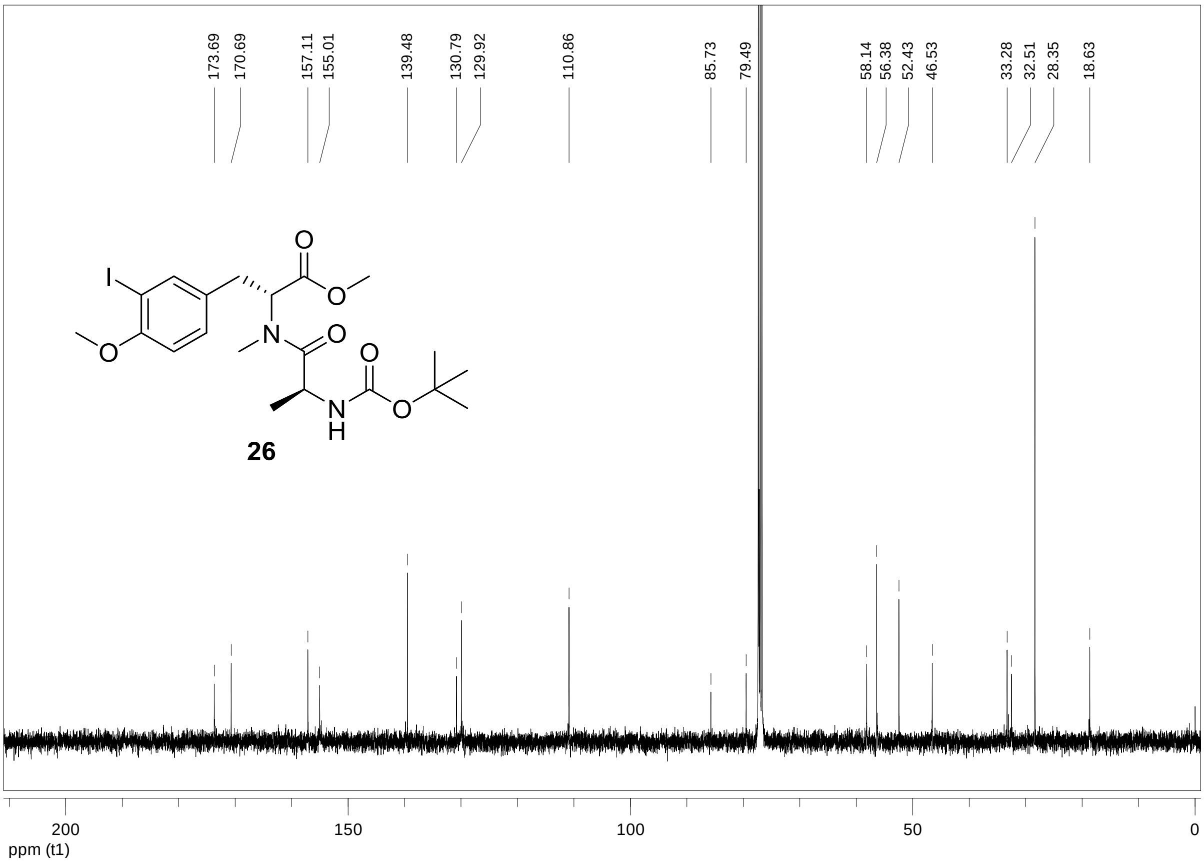

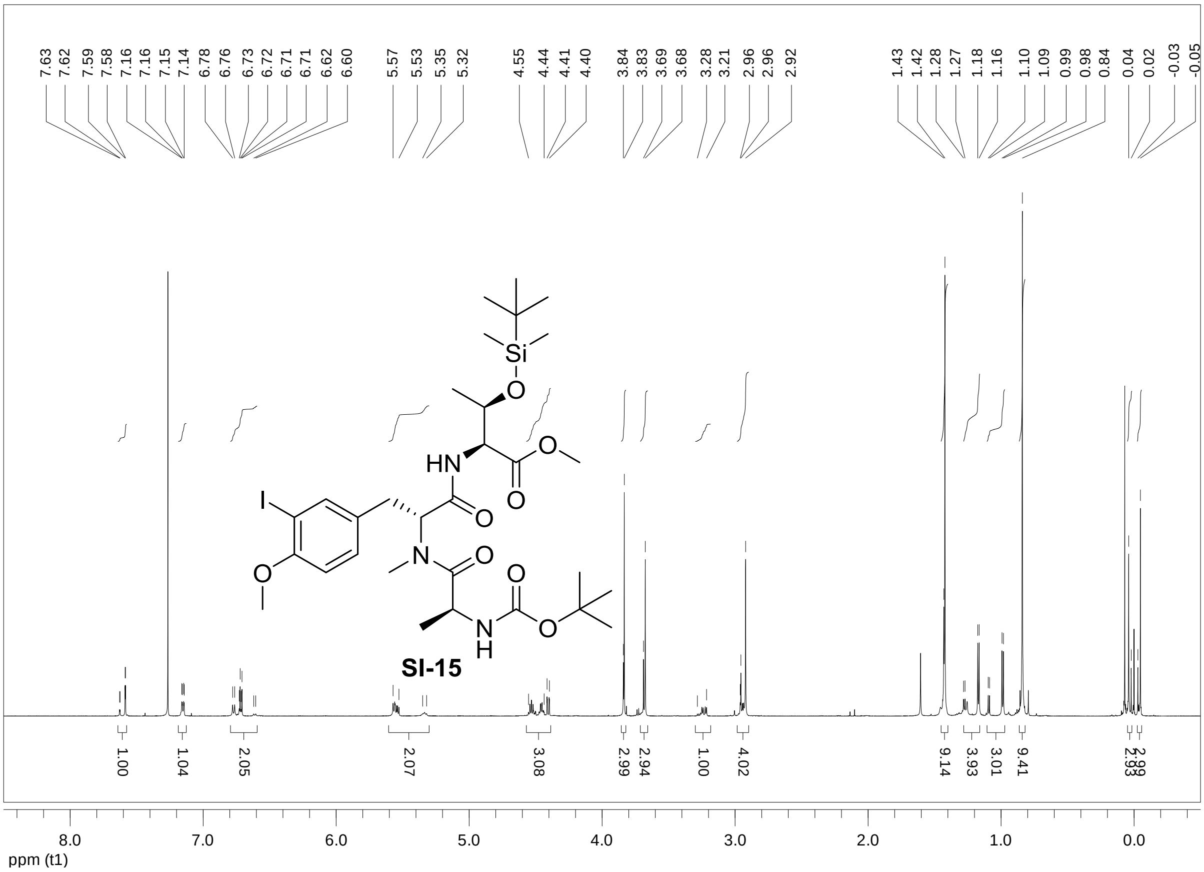

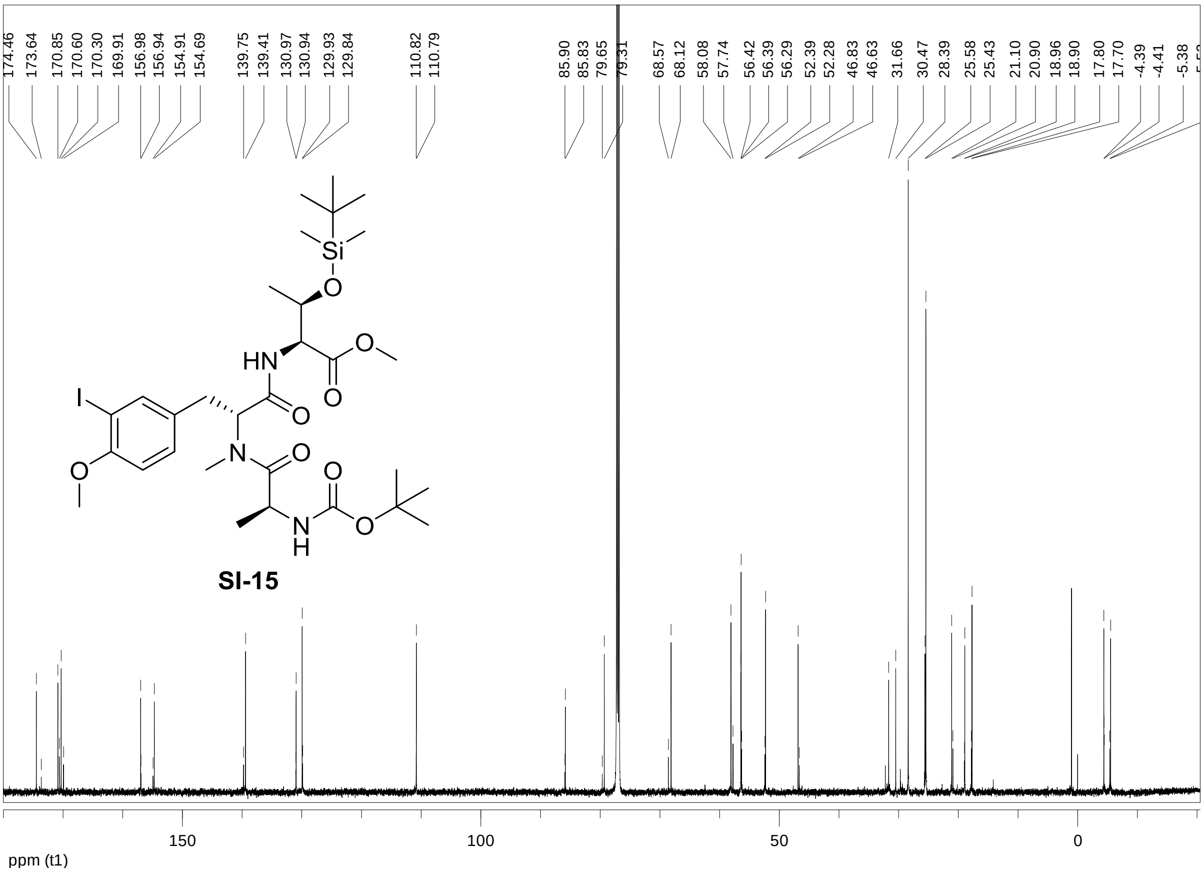

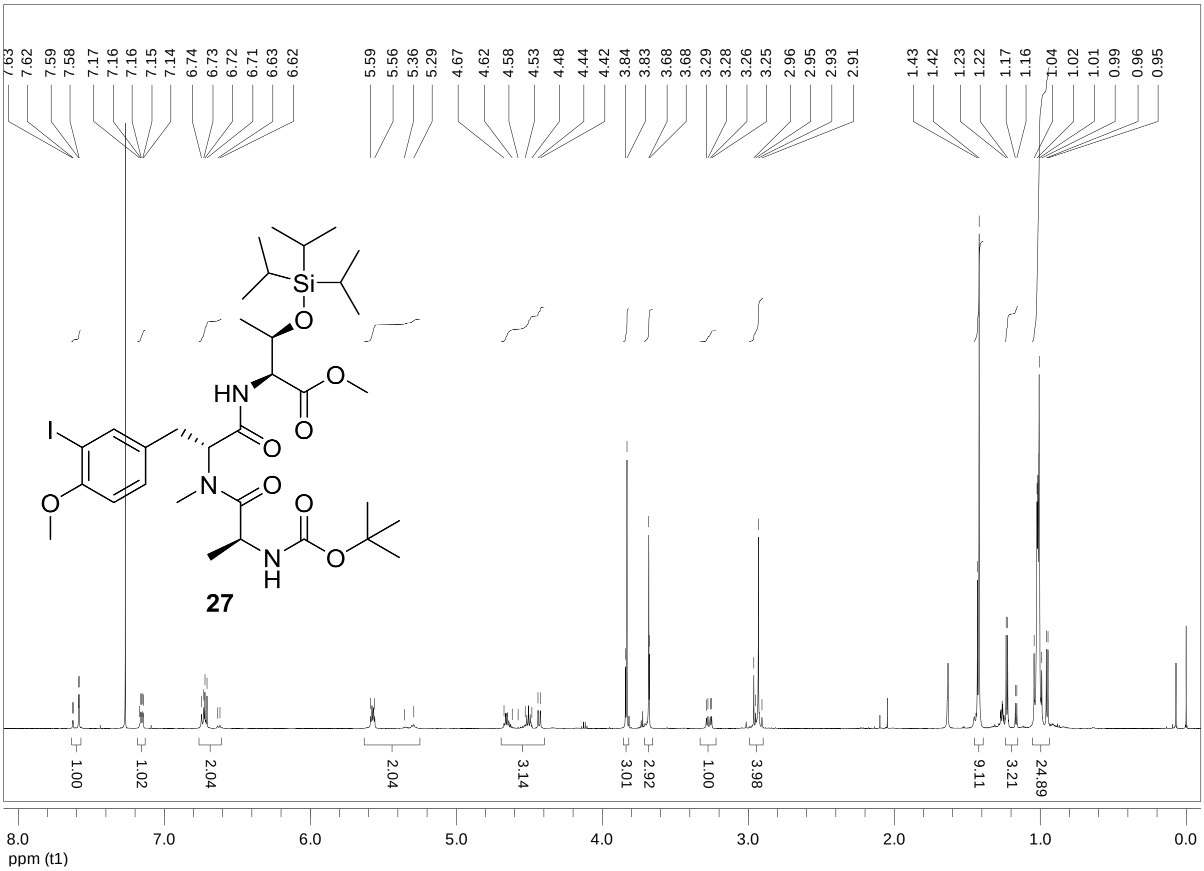
7.63 7.62 7.59 7.58 7.17 7.16 7.16 7.15 7.14 6.74 6.73 6.72 6.71 6.63 6.62 5.59 5.56 5.36 5.29 4.67 4.62 4.58 4.53 4.48 4.44 4.42 3.84 3.83 3.68 3.68 3.29 3.28 3.26 3.25 2.96 2.95 2.93 2.91 1.43 1.42 1.23 1.22 1.17 1.16 1.04 1.02 1.01 0.99 0.96 0.95
27
1.00 1.02 2.04 2.04 3.14 3.01 2.92 1.00 3.98 9.11 3.21 24.89
8.0 7.0 6.0 5.0 4.0 3.0 2.0 1.0 0.0
ppm (t1)

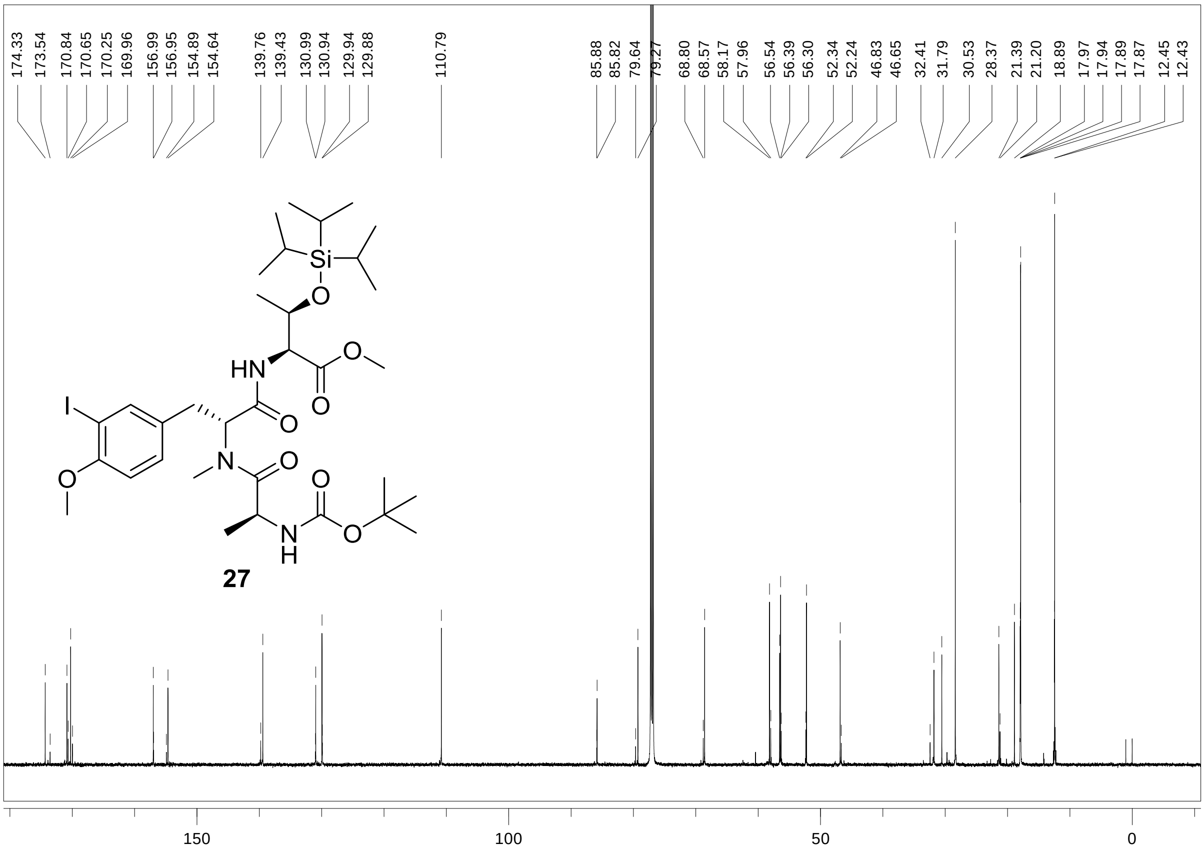
174.33 173.54 170.84 170.65 170.25 169.96 156.99 156.95 154.89 154.64 139.76 139.43 130.99 130.94 129.94 129.88 110.79 85.88 85.82 79.64 79.27 68.80 68.57 58.17 57.96 56.54 56.39 56.30 52.34 52.24 46.83 46.65 32.41 31.79 30.53 28.37 21.39 21.20 18.89 17.97 17.94 17.89 17.87 12.45 12.43
27
150 100 50 0
ppm (t1)

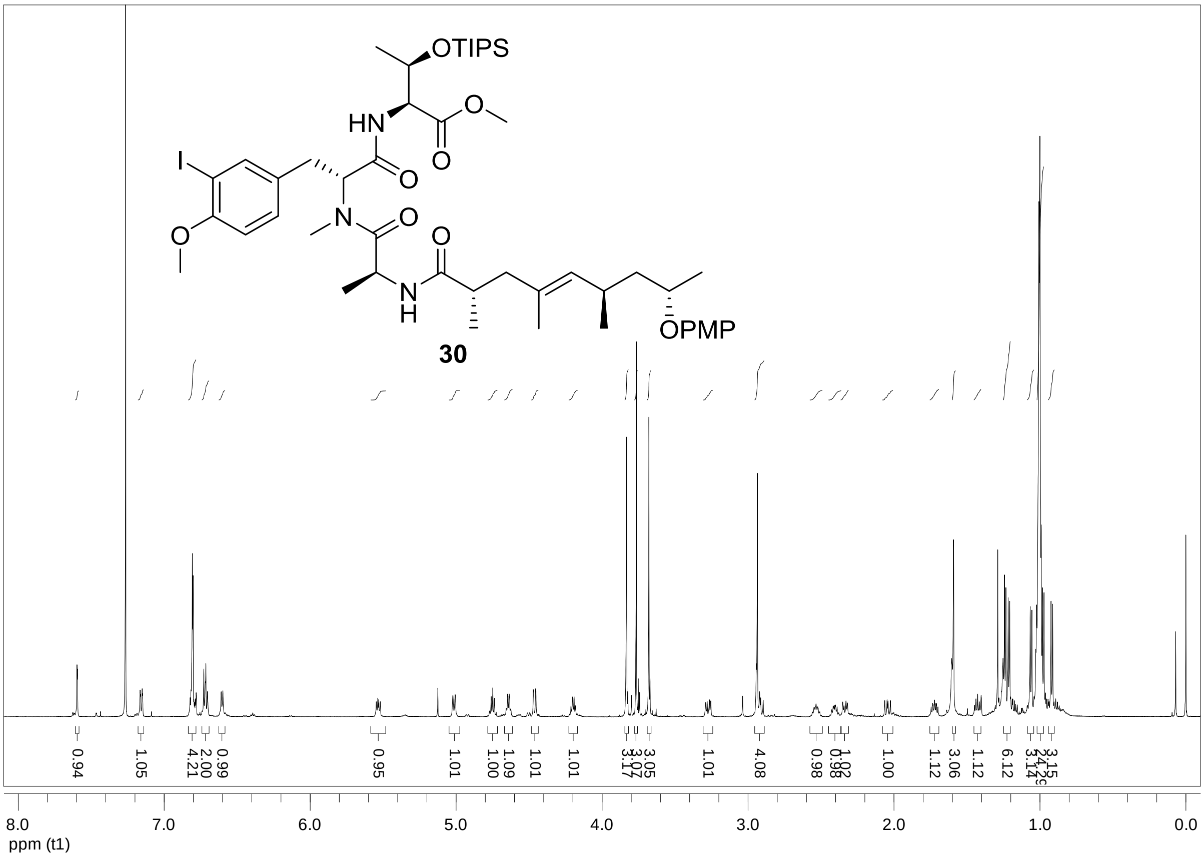

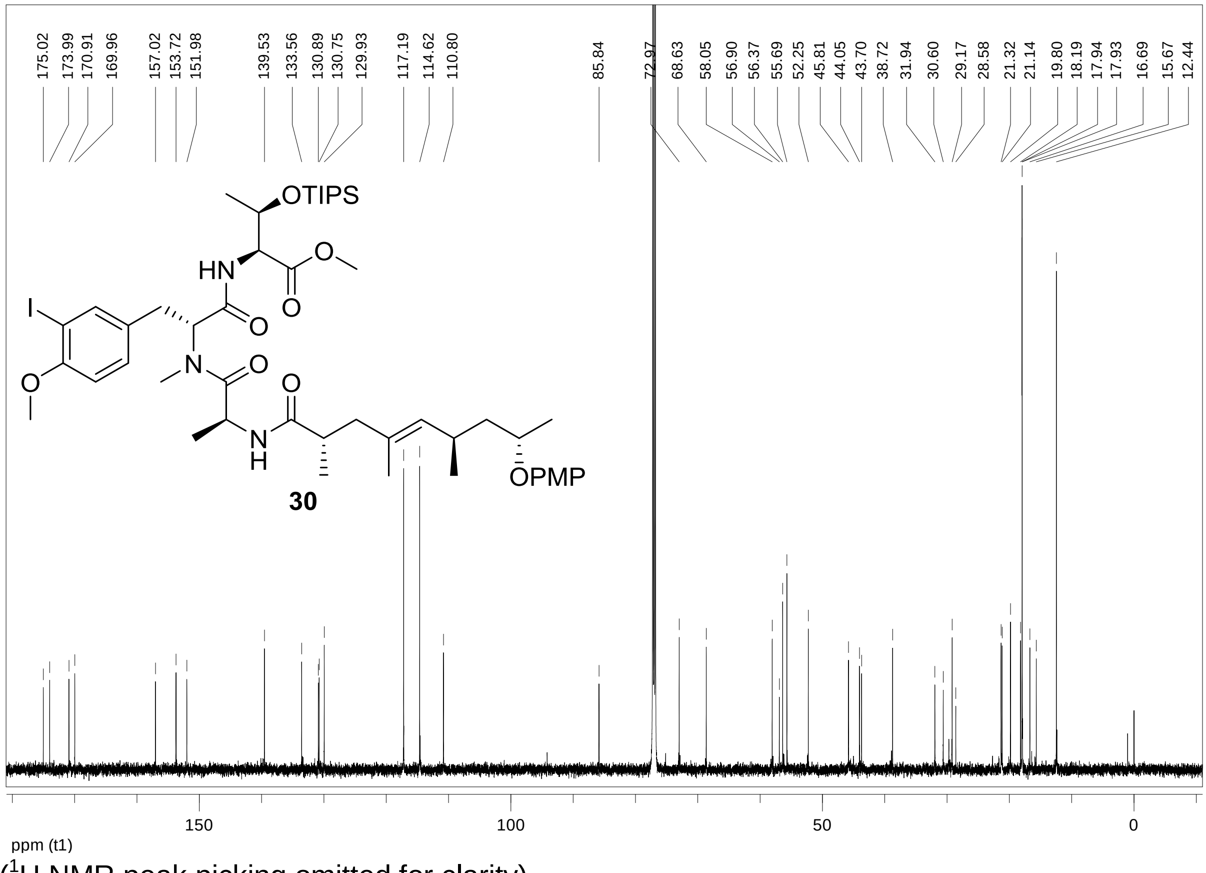

(^{1}H NMR peak picking omitted for clarity)

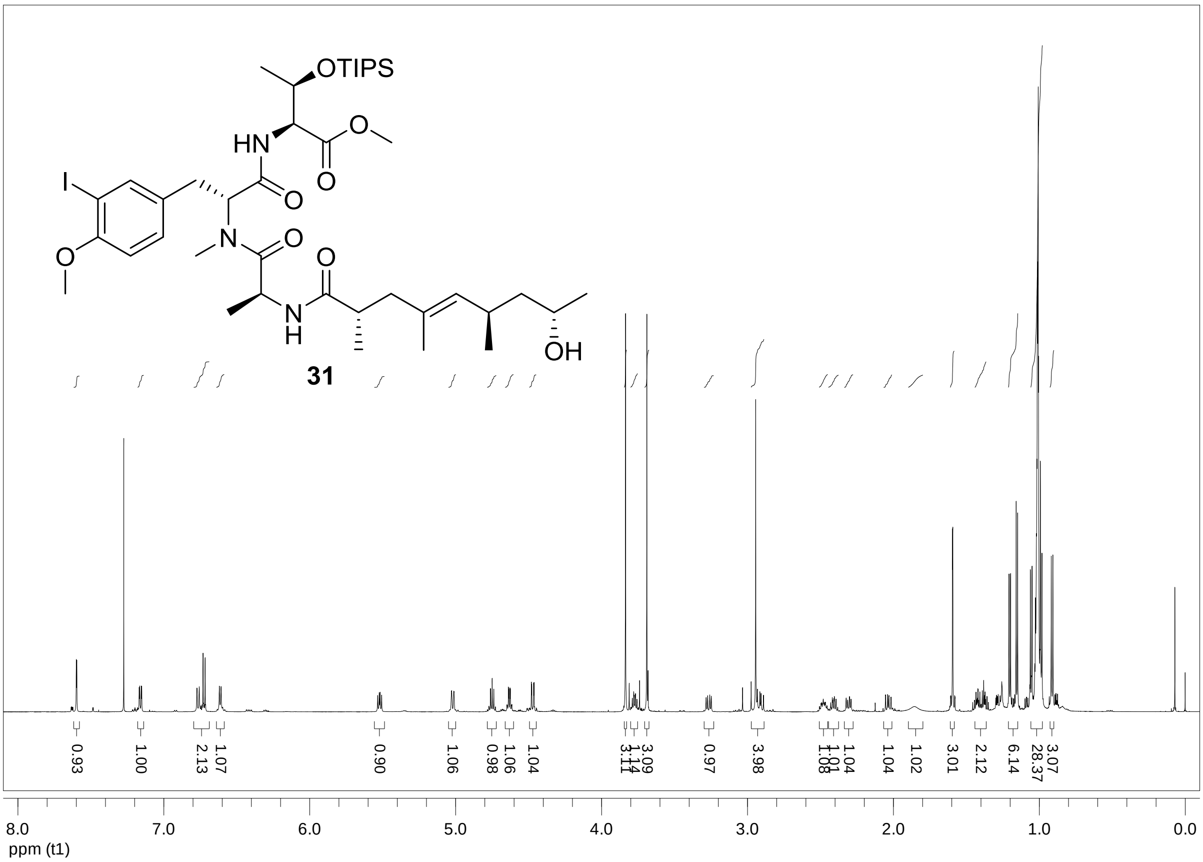

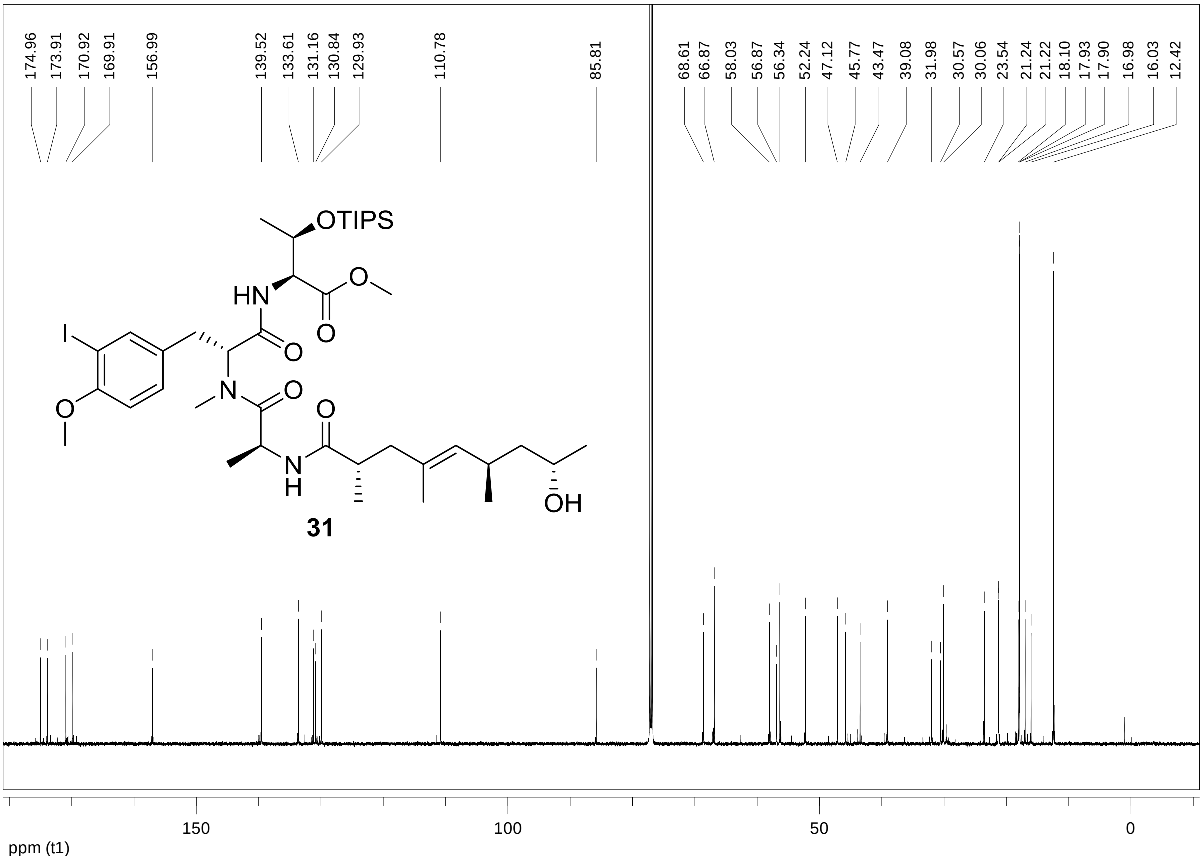

(¹H NMR peak picking omitted for clarity)
